# Revisiting recent Ostracod type material from Ishizaki with insights into species distribution and taxonomic reassessment

**DOI:** 10.1038/s41598-025-22250-5

**Published:** 2025-10-03

**Authors:** Gengo Tanaka, Sota Niiyama

**Affiliations:** 1 Aitsu Marine Station, Kumamoto University, 6061 Aitsu, Matsushima, Kami-Amakusa, Kumamoto , 861-6102 Japan; 2 Okinawa Prefectural Museum and Art Museum, 3-1-1Omoromachi, Naha City, Okinawa, 900-0006 Japan

**Keywords:** Biogeography, East Asia, Ishizaki, Japan, Neogene, Ostracoda, Animal migration, Biogeography, Biooceanography, Ecology, Ocean sciences

## Abstract

Ishizaki’s work is among the most significant taxonomic studies of recent Ostracods in Japan. However, limited access to scanning electron microscopes at that time led to several misidentifications, causing confusion in biogeographical interpretations, including the fossil record. In this study, we rephotographed Ishizaki’s type specimens under an electron microscope and compared them with illustrations from papers published over the past 70 years since Ishizaki’s work. This has enabled us to compile an updated synonym list and perform biogeographical assessments based on Titterton and Whatley’s definitions. Of the 47 Ishizaki specimens examined, 24 species either have the oldest fossil record or are exclusively found around Japan in both fossil and modern records. Four species, originally recorded on the coast of South China, including Taiwan, are now only found around Japan: *Sinoleberis tosaensis, Aurila tosaensis, Loxoconcha zamia*, and *Loxoconcha viva*. Additionally, 15 species exhibit a wider biogeographical distribution, ranging from the South China Sea, the Indochina Peninsula, the Philippines, and Indonesia to around Japan.

**Supplementary Information:**

The online version contains supplementary material available at 10.1038/s41598-025-22250-5.

## Introduction

**Fig. 1:**
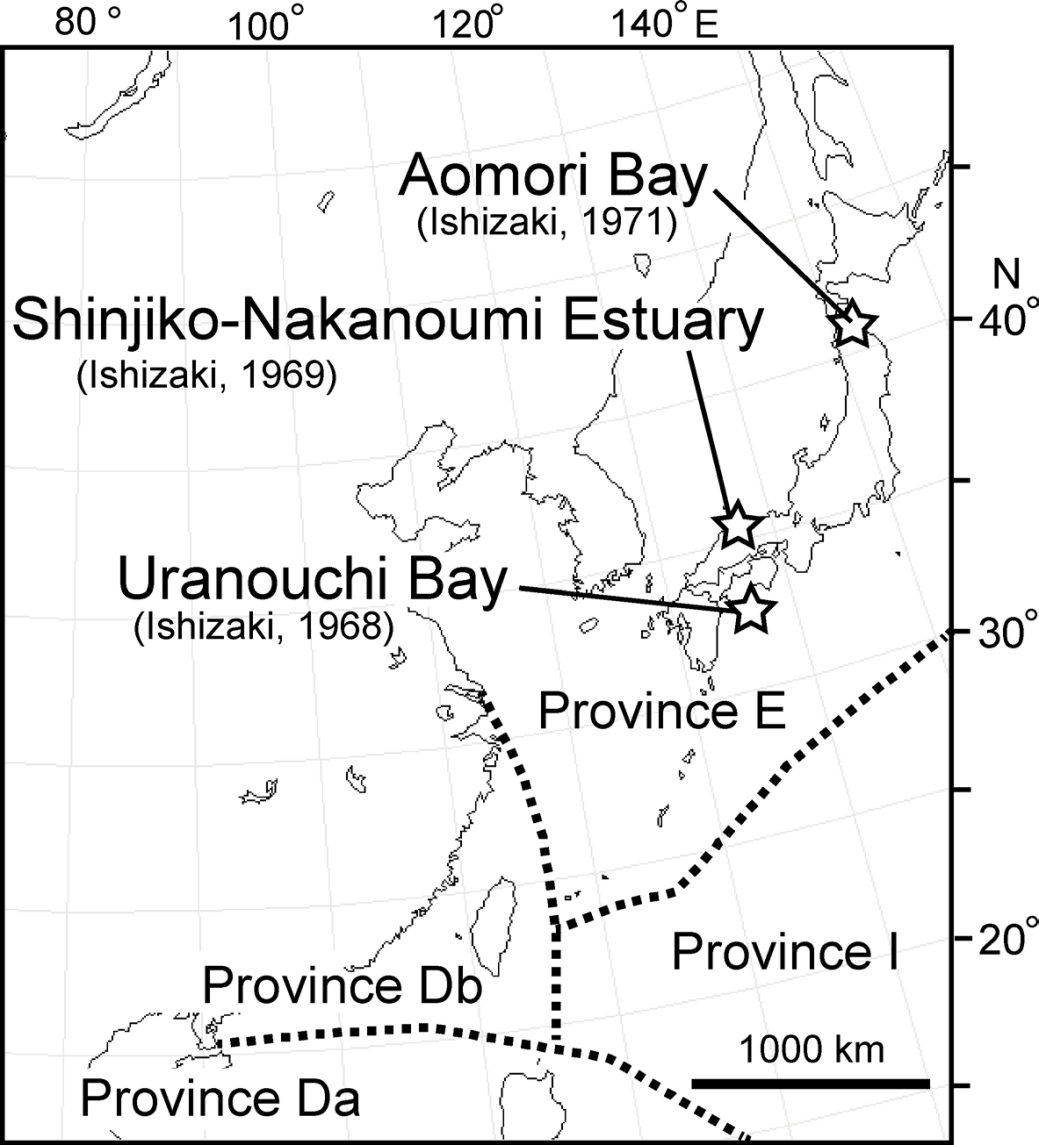
Location map from Ishizaki (1968–1971)^1–3^ and the Ostracod biogeographical provinces^4^.

One of the most important studies on Japanese Ostracods was conducted by Ishizaki during the late 1960s to early 1970s (Fig. 1): Uranouchi Bay^1^, Lake Shinji and Nakaumi Estuary^2^, and Aomori Bay^3^. His research has been crucial in clarifying the extant Ostracod fauna from Japan to Southeast Asia^4^ (Fig. 1) due to the wide biogeographical distribution of some species. Additionally, some species have been reported from the Miocene period around the eastern margin of the Eurasian Continent. However, the use of scanning electron microscopes was limited at that time. Although Ishizaki’s optical photographs were especially well taken, some misidentifications occurred. Hanai et al.^5^ created a checklist of Ostracod species described in Japan and adjacent areas, verifying it with Ikeya, Ishizaki, Sekiguchi, and Yajima. For this checklist, the Ostracod collections at the Natural History Museum in London (UK) and the Southeast Asian Ostracod collection at the University of Utrecht (NH) were checked by Ishizaki and Hanai, respectively. Hanai et al.^6^ compiled the Ostracod literature from Southeast Asia and published a checklist for the region, although their research was solely based on the collections at the University of Utrecht. Their effort significantly contributed to the classification of Ostracods around Japan. However, some taxonomic confusion arose after the 1980s. In this study, we reexamined the type specimens described by Ishizaki (1968–1971)^1–3^ from recent sediments, reviewed subsequent images by various authors up to 2024, and discussed the biogeographical significance of the species described by Ishizaki. We could not examine and discuss *Ambtonia obai* (Ishizaki, 1971)^3^ from Aomori Bay because we could not locate any type specimens, and the original article only included an external view of an optical photograph of a right valve and an illustration of the internal view. A biogeographical and taxonomic revision of *A. obai* was recently published^7^. Forty-six species belonging to twenty-six genera were figured and systematically checked before and after Ishizaki’s studies. These synonym lists, accumulated over a half century, provide the (paleo-) geographical distribution throughout the geological period and contribute to understanding the origin and migration of each species.

All species described by Ishizaki (1968–71)^1–3^ were obtained from surface sediments of inland bays. Inland bays are habitats for benthic Ostracods, extending into the land and bordered by deeper waters at the back. Consequently, these bays likely harbor many endemic species, forming unique biogeographical regions. This study focuses on bays with particularly high endemism around the Japanese archipelago, classified as “Province E”^4^, and considers the routes of introduction of the species based on their biogeography and fossil records.

## Material and methods

One of the authors, G. Tanaka, visited the Tohoku University Museum, which has taken over the Institute of Geology and Palaeontology, Tohoku University, Sendai (IGPS), where Ishizaki had previously registered specimens, and borrowed the type specimen of Ishizaki (1968–1971)^1–3^ stored under the care of Dr. Jun Nemoto. The specimens were carefully packed and transported to the Aitsu Marine Station of Kumamoto University. After cleaning each specimen with a fine brush and distilled water, it was placed on a SEM stub and observed under low vacuum at 15 kV with a TM-1000 electron microscope (Hitati) without coating, and images were captured. The specimens were then returned to their original slide, checked for any damage, and sent back to the Tohoku University Museum. The revised taxonomic information of each species is listed in the Supplementary Information (SI 1), and the dataset of (paleo) geographic record and microhabitat data of each species is provided in the Supplementary Information (SI 2). The following criteria were used to identify intraspecific variation. It is generally known that individuals growing in colder environments have larger body sizes than those growing in warmer environments^5^, and this is also observed in Ostracoda^6,7^. It is also known that individuals growing in warmer environments have better developed surface ornamentation than those growing in colder environments^8–10^. Therefore, differences in body size and the degree of development of surface ornamentation were considered to be intraspecific variation.

## Results

SEM images of the 47 species described by Ishizaki (1968–1971)^1–3^ are shown below (Table 1).

**Fig. 2:**
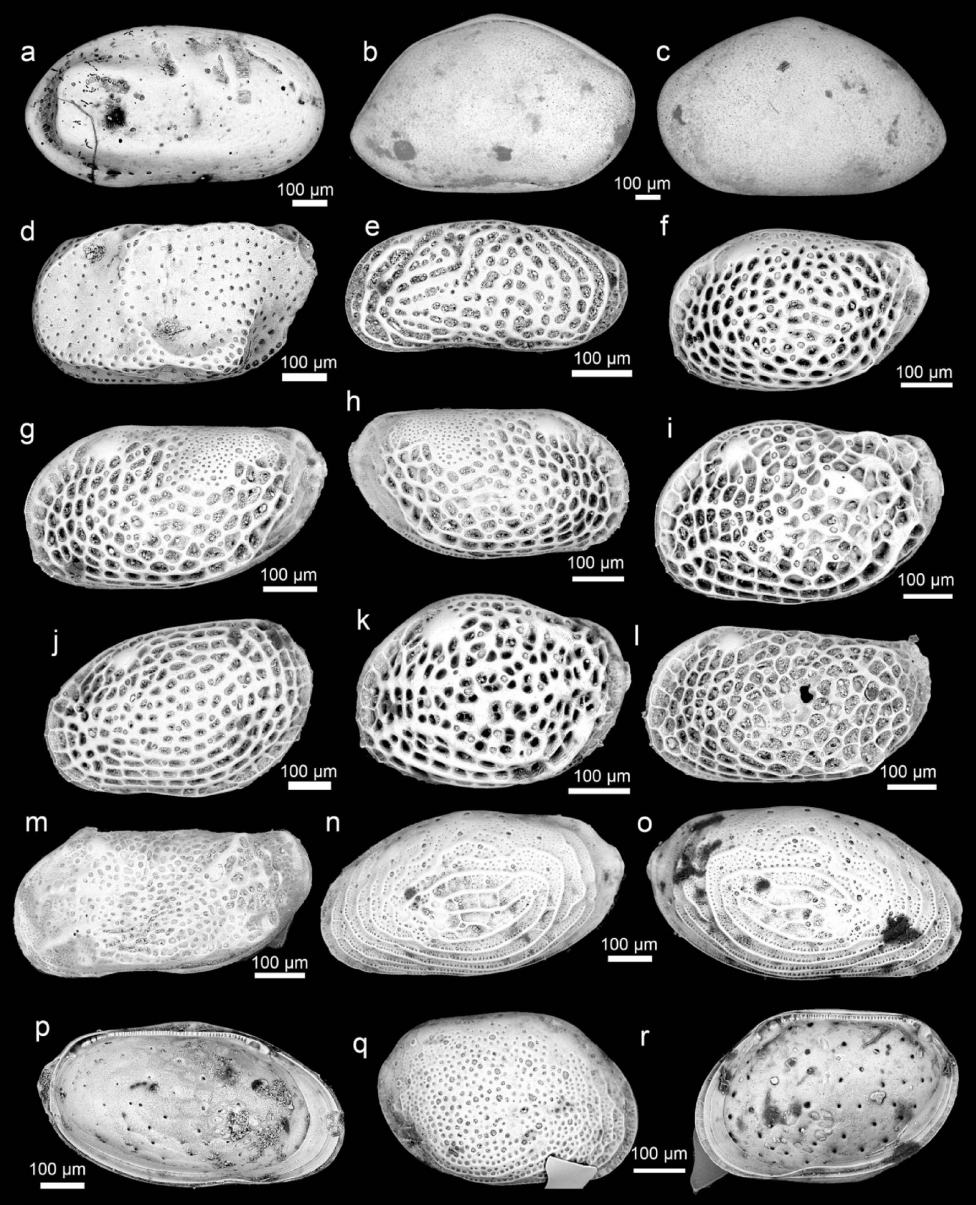
Ostracod type specimens from Ishizaki (1968–71); *Cytherellidae*, *Bairdiidae*, *Bythocytheridae*, *Leptocytheridae*, *Loxoconchidae*: (**a**) *Cytherelloidea munechikai* Ishizaki, 1968^1^ (Holotype, IGPS 90,324); (**b–c**) *Neonesidea mutsuensis* (Ishizaki, 1971)^3^ (Holotype, IGPS 90,334); (**d**) *Bythoceratina hanaii* Ishizaki, 1968^1^ (Holotype, IGPS 90,208); (**e**) *Tanella gracilis* Kingma, 1948^11^ (IGPS 90,247); (**f–h**) *Loxoconcha epeterseni* Ishizaki, 1981^12^ (Holotype, IGPS 90,266, Paratypes, IGPS 90,268); (**i**) *Loxoconcha hattorii* Ishizaki, 1971^3^ (Holotype, IGPS 91,556); (**j**) *Loxoconcha japonica* Ishizaki, 1968^1^ (Holotype, IGPS 90,260); (**k**) *Loxoconcha kattoi* Ishizaki, 1968^1^ (Holotype, IGPS 90,264); (**l**) *Loxoconcha kitanipponica* Ishizaki, 1971^3^ (Holotype, IGPS 91,559); (**m**) *Loxoconcha mutsuense* (Ishizaki, 1971)^3^ (Holotype, IGPS 91,571); (**n**–**p**) *Loxoconcha optima* Ishizaki, 1968^1^ (Holotype, IGPS 90,269); (**q**–**r**) *Loxoconcha pulchra* Ishizaki, 1968^1^ (Holotype, IGPS 90,270).

**Fig. 3:**
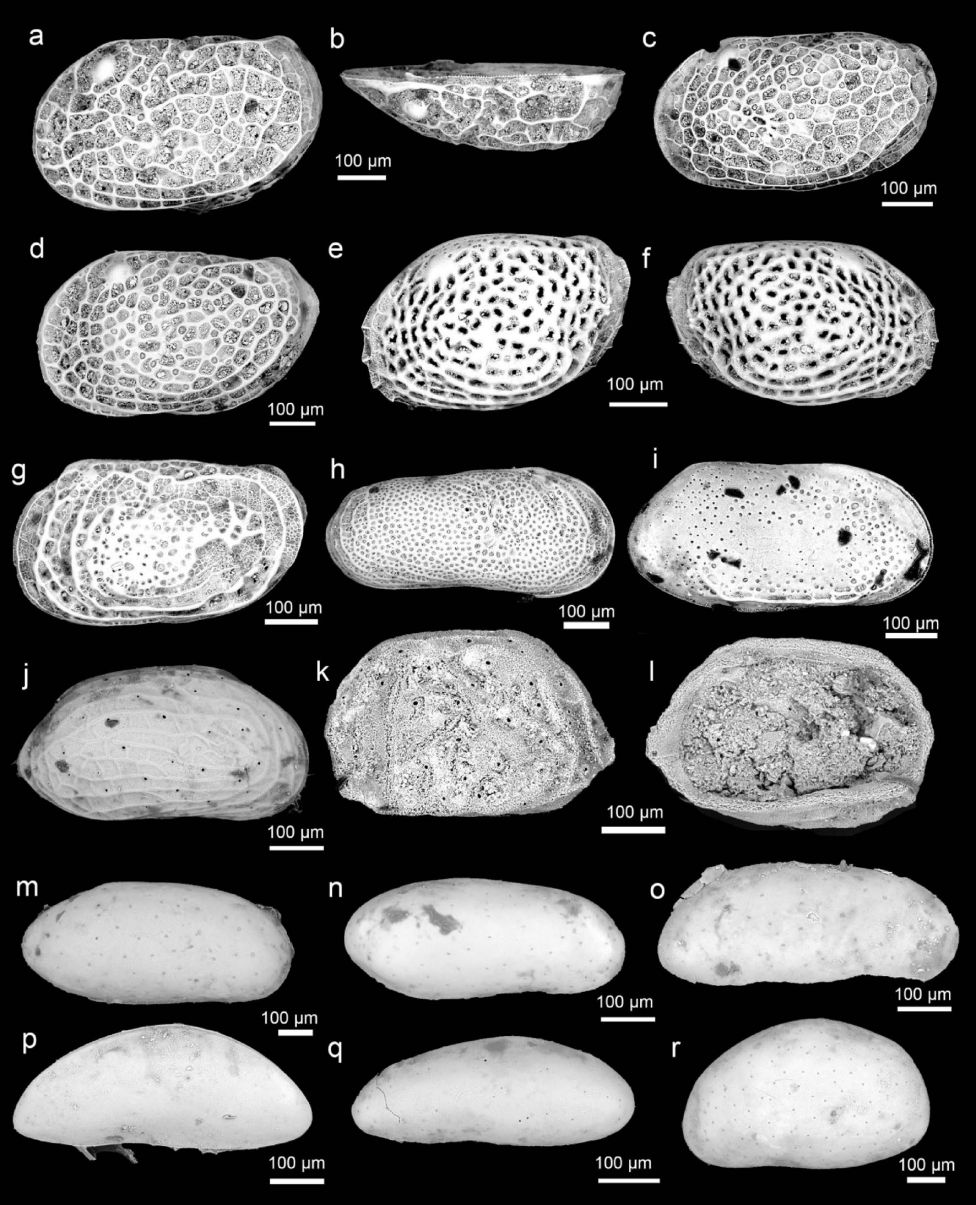
Ostracod type specimens from Ishizaki (1968–71); *Loxoconchidae*, *Cytheruridae*, *Paradoxostomatidae*, *Xestoleberididae*: (**a–b**) *Loxoconcha tosaensis* Ishizaki, 1968^1^ (Holotype, IGPS 90,272); (**c**) *Loxoconcha uranouchiensis* Ishizaki, 1968^1^ (Holotype, IGPS 90,276); (**d**) *Loxoconcha viva* Ishizaki, 1968^1^ (Holotype, IGPS 90,278); (**e–f**) *Loxoconcha zamia* Ishizaki, 1968^1^ (Holotype, IGPS 90,281); (**g**) *Miia uranouchiensis* Ishizaki, 1968^1^ (Holotype, IGPS 90,286); (**h**) *Cytheromorpha acupunctata* (Brady, 1880)^13^ (IGPS 90,290); (**i**) *Nipponocythere bicarinata* (Brady, 1880)^13^ (IGPS 91,567); (**j**) *Angulicytherura miii* (Ishizaki, 1969)^2^ (Holotype, IGPS 90,328); (**k–l**) *Howeina higashimeyaensis* Ishizaki, 1971 (Holotype, IGPS 90,350); (**m**) *Cytherois asamusiensis* Ishizaki, 1971^3^ (Holotype, IGPS 91,575); (**n**) *Cytherois nakanoumiensis* Ishizaki, 1969^2^ (Holotype, IGPS 87,018); (**o**) *Cytherois uranouchiensis* Ishizaki, 1968^1^ (Holotype, IGPS 90,295); (**p**) *Paracytherois mutsuensis* Ishizaki, 1971^3^ (Holotype, IGPS 91,579); (**q**) *Paracytherois tosaensis* Ishizaki, 1968^1^ (Holotype, IGPS 90,297); (**r**) *Xestoleberis hanaii* Ishizaki, 1968^1^ (Holotype, IGPS 90,316).

**Fig. 4:**
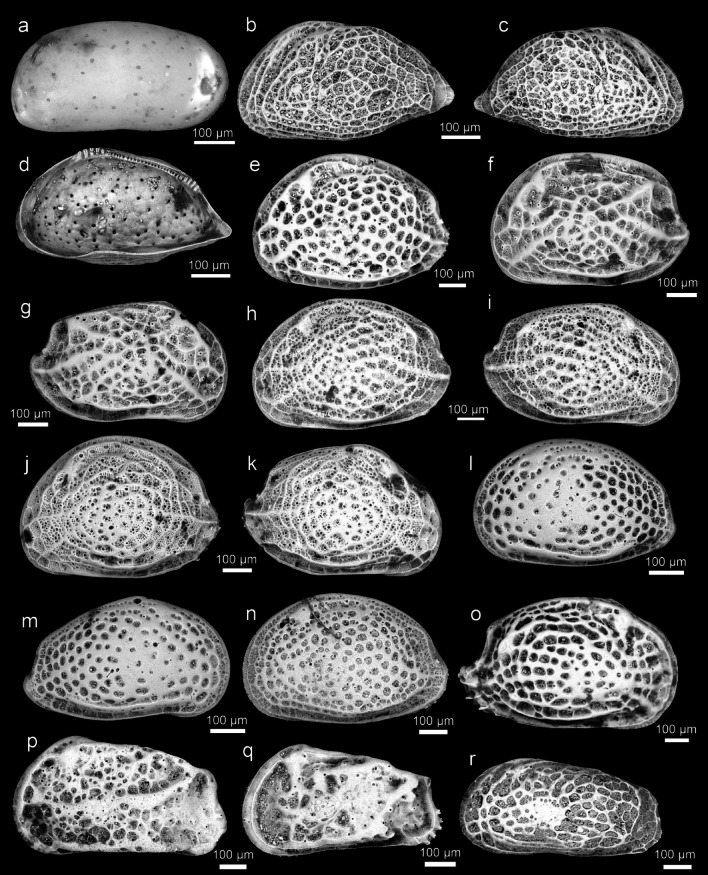
Ostracod type specimens from Ishizaki (1968–71); *Krithidae, Cytherideidae, Hemicytheridae*: (**a**) *Krithe japonica* Ishizaki, 1971^3^ (Holotype, IGPS 90,342); (**b–d**) *Perissocytheridea japonica* Ishizaki, 1968^1^ (Holotype, IGPS 90,204); (**e**) *Aurila cymba* (Brady, 1969)^14^ (Holotype, IGPS 90,231); (**f–g**) *Aurila hataii* Ishizaki, 1968^1^ (Holotype, IGPS 90,299); (**h–i**) *Aurila imotoi* Ishizaki, 1968^1^ (Holotype, IGPS 90,230); (**j–k**) *Aurila munechikai* Ishizaki, 1968^1^ (Holotype, IGPS 90,233); (**l–m**) *Aurila tosaensis* Ishizaki, 1968^1^ (Holotype, IGPS 90,234); (**n**) *Aurila uranouchiensis* Ishizaki, 1968^1^ (Holotype, IGPS 90,235); (**o**) *Pseudoaurila japonica* (Ishizaki, 1968)^1^ (Holotype, IGPS 90,240); (**p**) *Caudites japonicus* Ishizaki, 1971^3^ (Holotype, IGPS 91,541); (**q**) *Cornucoquimba tosaensis* (Ishizaki, 1968)^1^ (Holotype, IGPS 90,313); (**r**) *Finmarchinella uranipponica* Ishizaki, 1969^2^ (Holotype), IGPS 87,044).

**Fig. 5:**
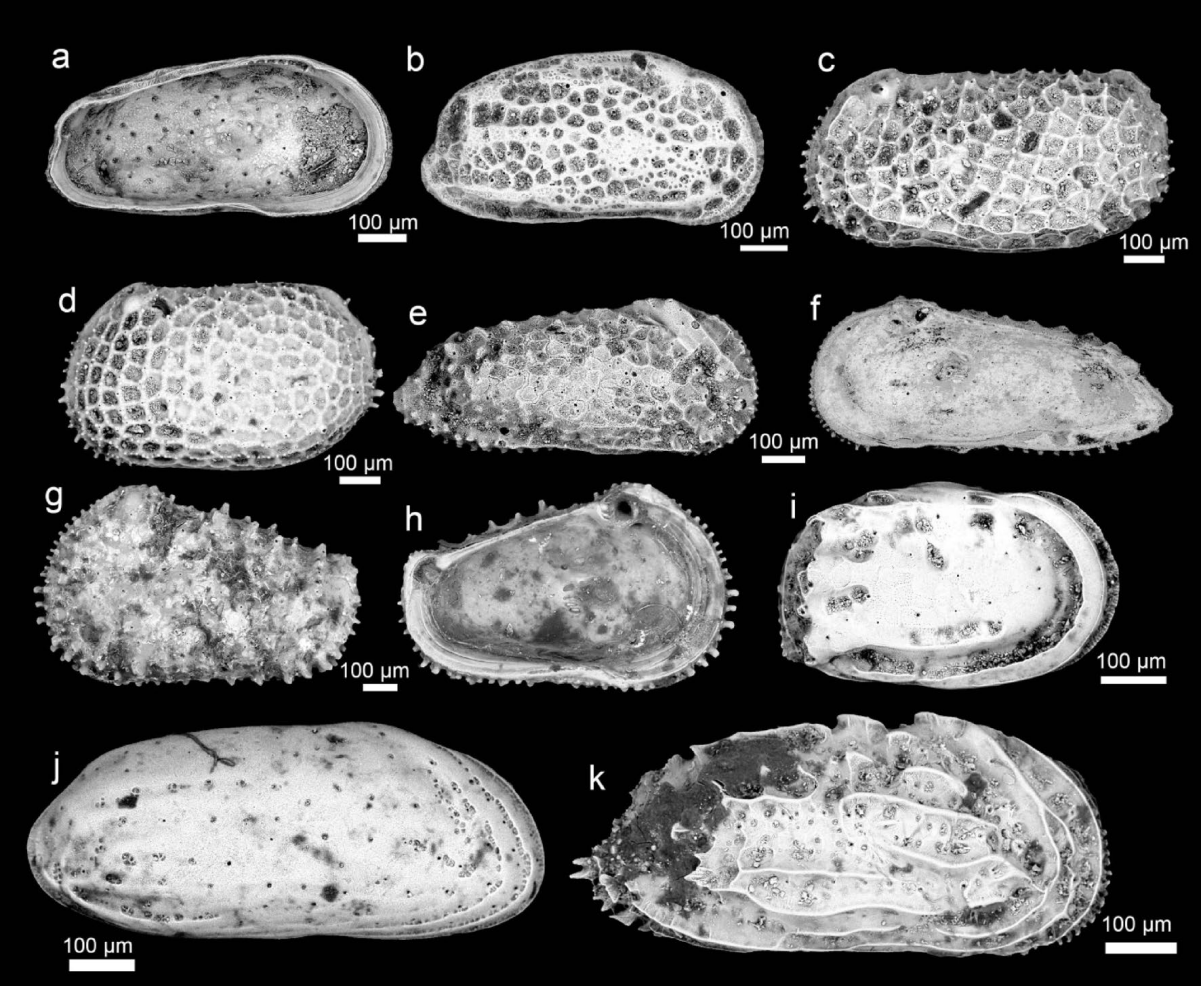
Ostracod type specimens from Ishizaki (1968–71); Hemicytheridae, Trachyleberididae: (**a**) *Finmarchinella uranipponica* Ishizaki, 1969^2^ (Holotype), IGPS 87,044); (**b**) *Urocythereis miii* Ishizaki, 1969^2^ (Holotype, IGPS 90,332); (**c**) *Pistocythereis bradyformis* (Ishizaki, 1968)^1^ (Holotype, IGPS 90,311); (**d**) *Pistocythereis bradyi* (Ishizaki, 1968)^1^ (Holotype, IGPS 90,312); (**e–f**) *Trachyleberis niitsumai* Ishizaki, 1971^3^ (Holotype, IGPS 91,705); (**g–h**) *Acanthocythereis mutsuensis* Ishizaki, 1971^3^ (Holotype, IGPS 91,708); (**i**) *Pacambocythere japonica* (Ishizaki, 1968)^1^ (Holotype, IGPS 90,307); (**j**) *Doratocythere tomokoae* (Ishizaki, 1968)^1^ (Holotype, IGPS 90,245); 5(**k**) *Sinoleberis tosaensis* (Ishizaki, 1968)^1^ (Holotype, IGPS 90,304).

1. *Cytherelloidea munechikai* Ishizaki, 1968^1^ Fig. 2a
2. *Neonesidea mutsuensis* (Ishizaki, 1971)^3^ Fig. 2b–c
3. *Bythoceratina hanaii* Ishizaki, 1968^1^ Fig. 2d
4. *Tanella gracilis* Kingma, 1948^11^ Fig. 2e
5. *Loxoconcha epeterseni* Ishizaki, 1981^12^ Fig. 2f–h
6. *Loxoconcha hattorii* Ishizaki, 1971^3^ Fig. 2i
7. *Loxoconcha japonica* Ishizaki, 1968^1^ Fig. 2j
8. *Loxoconcha kattoi* Ishizaki, 1968^1^ Fig. 2k
9. *Loxoconcha kitanipponica* Ishizaki, 1971^3^ Fig. 2l
10. *Loxoconcha mutsuense* (Ishizaki, 1971)^3^ Fig. 2m
11. *Loxoconcha optima* Ishizaki, 1968^1^ Fig. 2n–p
12. *Loxoconcha pulchra* Ishizaki, 1968^1^ Fig. 2q–r
13. *Loxoconcha tosaensis* Ishizaki, 1968^1^ Fig. 3a–b
14. *Loxoconcha uranouchiensis* Ishizaki, 1968^1^ Fig. 3c
15. *Loxoconcha viva* Ishizaki, 1968^1^ Fig. 3d
16. *Loxoconcha zamia* Ishizaki, 1968^1^ Fig. 3e–f
17. *Miia uranouchiensis* Ishizaki, 1968^1^ Fig. 3g
18. *Cytheromorpha acupunctata* (Brady, 1880) ^13^ Fig. 3h
19. *Nipponocythere bicarinata* (Brady, 1880)^13^ Fig. 3i
20. *Angulicytherura miii* (Ishizaki, 1969)^2^ Fig. 3j
21. *Howeina higashimeyaensis* Ishizaki, 1971^3^ Fig. 3k, l
22. *Cytherois asamusiensis* Ishizaki, 1971^3^ Fig. 3m
23. *Cytherois nakanoumiensis* Ishizaki, 1969^2^ Fig. 3n
24. *Cytherois uranouchiensis* Ishizaki, 1968^1^ Fig. 3o
25. *Paracytherois mutsuensis* Ishizaki, 1971^3^ Fig. 3p
26. *Paracytherois tosaensis* Ishizaki, 1968^1^ Fig. 3q
27. *Xestoleberis hanaii* Ishizaki, 1968^1^ Fig. 3r
28. *Krithe japonica* Ishizaki, 1971^3^ Fig. 4a
29. *Perissocytheridea japonica* Ishizaki, 1968^1^ Fig. 4b–d
30. *Aurila cymba* (Brady, 1969)^14^ Fig. 4e
31. *Aurila hataii* Ishizaki, 1968^1^ Fig. 4f, g
32. *Aurila imotoi* Ishizaki, 1968^1^ Fig. 4h, i
33. *Aurila munechikai* Ishizaki, 1968^1^ Fig. 4j, k
34. *Aurila tosaensis* Ishizaki, 1968^1^ Fig. 4l, m
35. *Aurila uranouchiensis* Ishizaki, 1968^1^ Fig. 4n
36. *Pseudoaurila japonica* (Ishizaki, 1968)^1^ Fig. 4o
37. *Caudites japonicus* Ishizaki, 1971^3^ Fig. 4p
38. *Cornucoquimba tosaensis* (Ishizaki, 1968)^1^ Fig. 4q
39. *Finmarchinella uranipponica* Ishizaki, 1969^2^ Fig. 4r, 5a
40. *Urocythereis miii* Ishizaki, 1969^2^ Fig. 5b
41. *Pistocythereis bradyformis* (Ishizaki, 1968)^1^ Fig. 5c
42. *Pistocythereis bradyi* (Ishizaki, 1968)^1^ Fig. 5d
43. *Trachyleberis niitsumai* Ishizaki, 1971^3^ Fig. 5e, f
44. *Acanthocythereis mutsuensis* Ishizaki, 1971^3^ Fig. 5g, h
45. *Pacambocythere japonica* (Ishizaki, 1968)^1^ Fig. 5i
46. *Doratocythere tomokoae* (Ishizaki, 1968)^1^ Fig. 5j
47. *Sinoleberis tosaensis* (Ishizaki, 1968)^1^ Fig. 5k

**Table 1:** Correspondence table between extant Ostracod species described by Ishizaki (1968–1971) and the species in this study.

Ishizaki’s taxonomic status	This study
*Cytherelloidea munechikai* Ishizaki, 1968	*Cytherelloidea munechikai* Ishizaki, 1968
*Bairdia (Neonesidea) mutsuensis* Ishizaki, 1971	*Neonesidea mutsuensis* (Ishizaki, 1971)
*Bythoceratina hanaii* Ishizaki, 1968	*Bythoceratina hanaii* Ishizaki, 1968
*Leptocythere ? tosaensis* Ishizaki, 1968	*Tanella gracilis* Kingma, 1948
*Loxoconcha laeta* Ishizaki, 1968	*Loxoconcha epeterseni* Ishizaki, 1981
*Loxoconcha hattorii* Ishizaki, 1971	*Loxoconcha hattorii* Ishizaki, 1971
*Loxoconchajaponica* Ishizaki, 1968	*Loxoconcha japonica* Ishizaki, 1968
*Loxoconcha kattoi* Ishizaki, 1968	*Loxoconcha kattoi* Ishizaki, 1968
*Loxoconcha kitanipponica* Ishizaki, 1971	*Loxoconcha kitanipponica* Ishizaki, 1971
*Loxocorniculum mutsuensis* Ishizaki, 1971	*Loxoconcha mutsuense* (Ishizaki, 1971)
*Loxoconcha optima* Ishizaki, 1968	*Loxoconcha optima* Ishizaki, 1968
*Loxoconcha pulchra* Ishizaki, 1968	*Loxoconcha pulchra* Ishizaki, 1968
*Loxoconcha tosaensis* Ishizaki, 1968	*Loxoconcha tosaensis* Ishizaki, 1968
*Loxoconcha uranouchiensis* Ishizaki, 1968	*Loxoconcha uranouchiensis* Ishizaki, 1968
*Loxoconcha viva* Ishizaki, 1968	*Loxoconcha viva* Ishizaki, 1968
*Loxoconcha zamia* Ishizaki, 1968	*Loxoconcha zamia* Ishizaki, 1968
*Miia uranouchiensis* Ishizaki, 1968	*Miia uranouchiensis* Ishizaki, 1968
*Cytheromorpha japonica* Ishizaki, 1968	*Cytheromorpha acupunctata* (Brady, 1880)
*Nipponocythere asamushiensis* Ishizaki, 1971	*Nipponocythere bicarinata* (Brady, 1880)
*Tetracytherura miii* Ishizaki, 1969	*Angulicytherura miii* (Ishizaki, 1969)
*Howeina higashimeyaensis* Ishizaki, 1971	*Howeina higashimeyaensis* Ishizaki, 1971
*Cytherois asamusiensis* Ishizaki, 1971	*Cytherois asamusiensis* Ishizaki, 1971
*Cytherois nakanoumiensis* Ishizaki, 1969	*Cytherois nakanoumiensis* Ishizaki, 1969
*Cytherois uranouchiensis* Ishizaki, 1968s	*Cytherois uranouchiensis* Ishizaki, 1968
*Paracytherois mutsuensis* Ishizaki, 1971	*Paracytherois mutsuensis* Ishizaki, 1971
*Paracytherois tosaensis* Ishizaki, 1968	*Paracytherois tosaensis* Ishizaki, 1968
*Xestoleberis hanaii* Ishizaki, 1968	*Xestoleberis hanaii* Ishizaki, 1968
*Krithe japonica* Ishizaki, 1971	*Krithe japonica* Ishizaki, 1971
*Perissocytheridea japonica* Ishizaki, 1968	*Perissocytheridea japonica* Ishizaki, 1968
*Aurila miii* Ishizaki, 1968	*Aurila cymba* (Brady, 1969)
*Aurila hataii* Ishizaki, 1968	*Aurila hataii* Ishizaki, 1968
*Aurila imotoi* Ishizaki, 1968	*Aurila imotoi* Ishizaki, 1968
*Aurila munechikai* Ishizaki, 1968	*Aurila munechikai* Ishizaki, 1968
*Aurila tosaensis* Ishizaki, 1968	*Aurila tosaensis* Ishizaki, 1968
*Aurila uranouchiensis* Ishizaki, 1968	*Aurila uranouchiensis* Ishizaki, 1968
*Pokornyella japonica* Ishizaki, 1968	*Pseudoaurila japonica* (Ishizaki, 1968)
*Caudites japonicus* Ishizaki, 1971SSS	*Caudites japonicus* Ishizaki, 1971
*Hermanites tosaensis* Ishizaki, 1968	*Cornucoquimba tosaensis* (Ishizaki, 1968)
*Finmarchinella uranipponica* Ishizaki, 1969	*Finmarchinella uranipponica* Ishizaki, 1969
*Urocythereis miii* Ishizaki, 1969	*Urocythereis miii* Ishizaki, 1969
*Echinocythereis bradyformis* Ishizaki, 1968	*Pistocythereis bradyformis* (Ishizaki, 1968)
*Echinocythereis bradyi* Ishizaki, 1968	*Pistocythereis bradyi* (Ishizaki, 1968)
*Trachyleberis niitsumai* Ishizaki, 1971	*Trachyleberis niitsumai* Ishizaki, 1971
*Acanthocythereis mutsuensis* Ishizaki, 1971	*Acanthocythereis mutsuensis* Ishizaki, 1971
*Ambocythere japonica* Ishizaki, 1968	*Pacambocythere japonica* (Ishizaki, 1968)
*Leguminocythereis tomokoae* Ishizaki, 1968	*Doratocythere tomokoae* (Ishizaki, 1968)
*Trachyleberis tosaensis* Ishizaki, 1968	*Sinoleberis tosaensis* (Ishizaki, 1968)

## Discussion

**Fig. 6:**
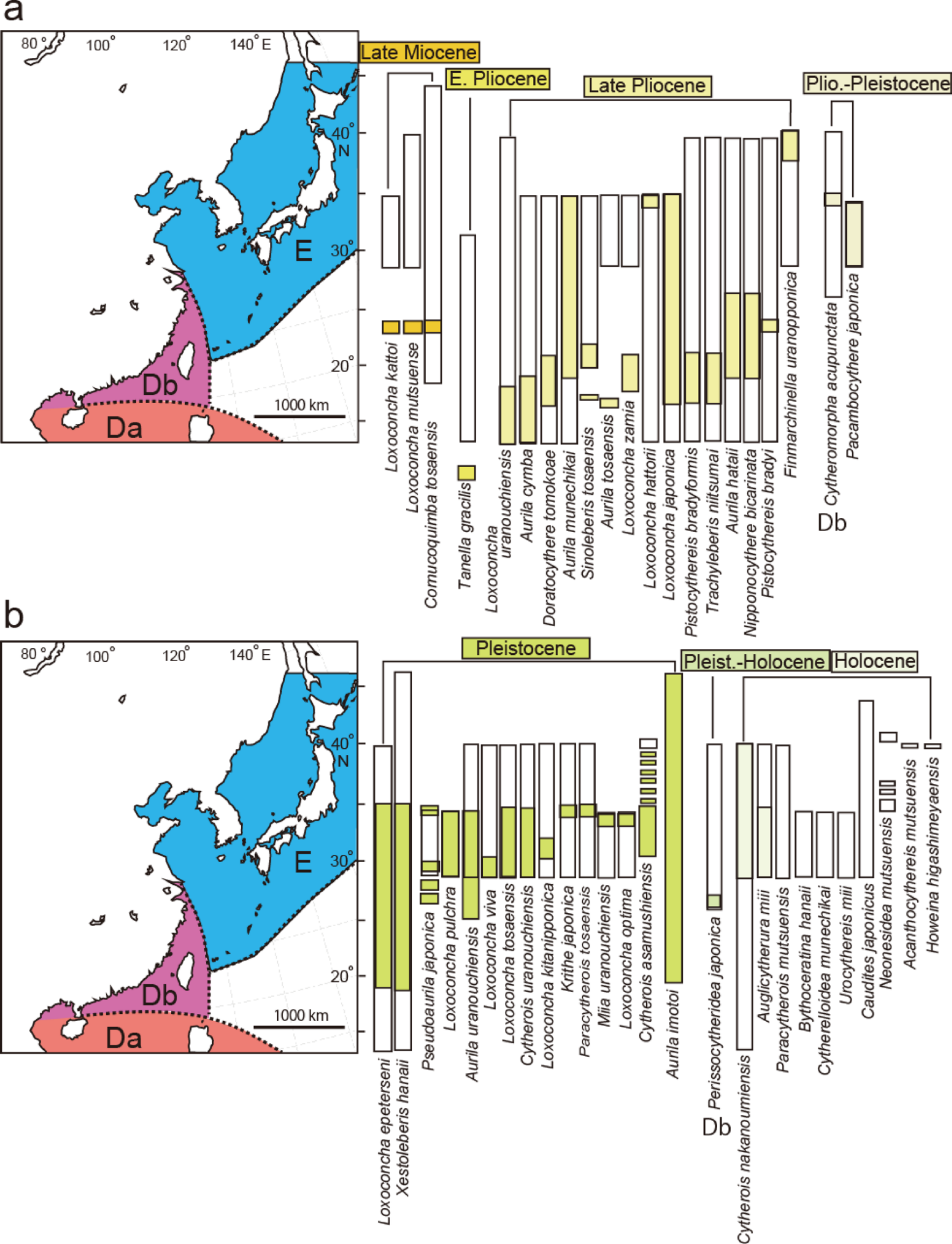
Location of the earliest fossil record (colour bars) and Recent geographic distribution (white bars) of each species described by Ishizaki (1968–71)^1–3^. (**a**) 21 species with the earliest fossil record from Late Miocene to Plio-Pleistocene. (**b**) 26 species with the earliest fossil record from Pleistocene to Holocene. E, Db, and Da refer to Provinces E, Db, and Da, respectively^4^.

**Fig. 7:**
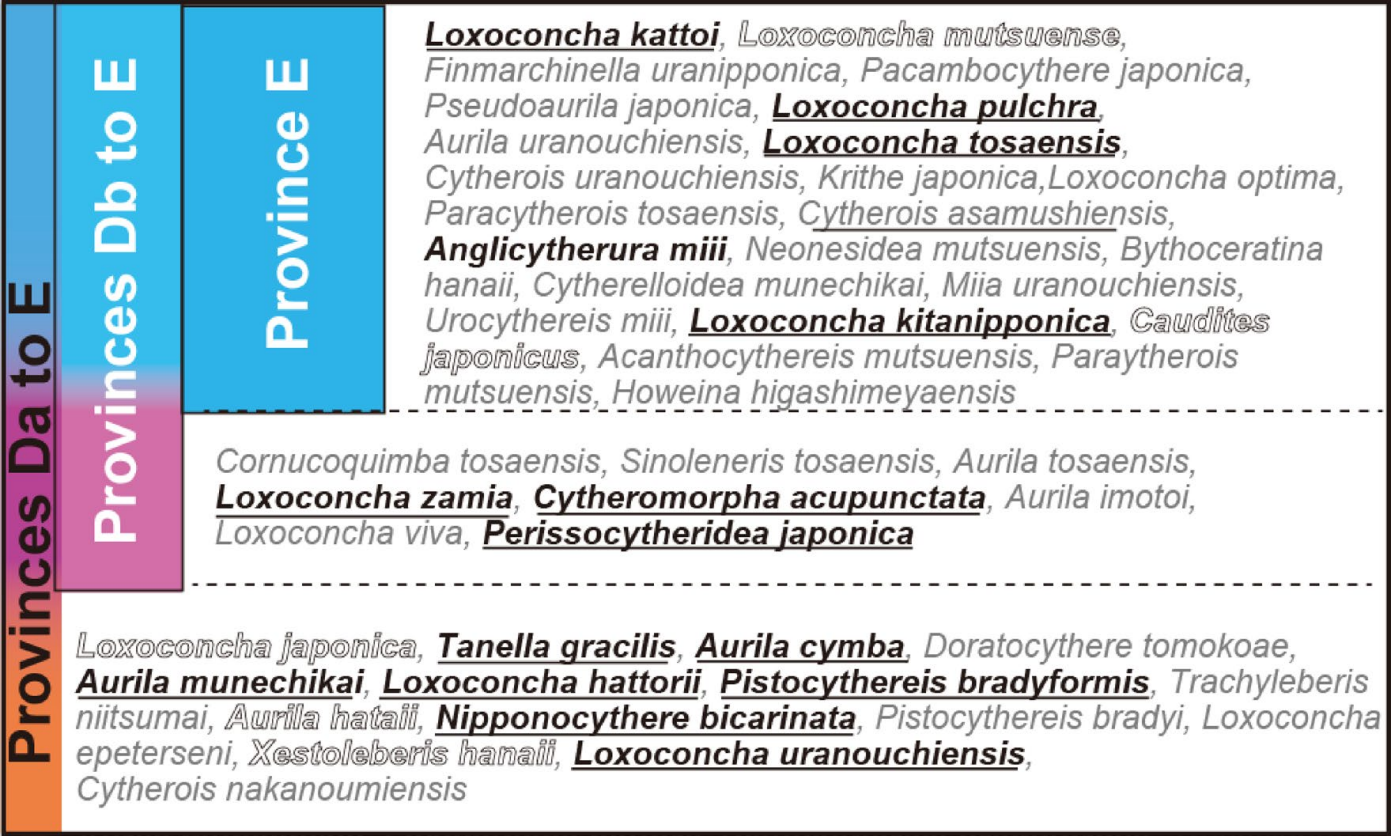
Biogeographical distribution of the Ostracod type specimens described by Ishizaki (1968–71)^1–3^ and their microhabitats. Species listed in white represent phytal species, those in black underlined are mud/sand creeping species, and species in gray have unknown microhabitats. The Province E, the Province Db, and the Province Da are defined according to previous study^4^.

Previous research^4^ studied 2599 species of Ostracods from the Cenozoic era onward in the Indo-Pacific region, identifying 13 zoogeographical provinces. They designated the area around Japan as Province E, the coast of South China, including Taiwan, as Province Db, and the South China Sea south of Hainan Island, the Indochina Peninsula, the Philippines, and Indonesia as Province Da (Fig. 6). Of the 47 Ishizaki specimens examined here, 24 species are endemic to Province E in both fossil and modern records (Figs. 6, 7, SI 2). Eight species have a broader distribution, ranging from Province E to Province Db (Figs. 6, 7). The remaining 15 species have an even wider distribution, from Province E to Province Da (Figs. 6, 7). Thus, approximately 50% of Ishizaki’s described species are characteristic of Province A, while the other 50% of the Ostracods have a broader biogeographical distribution extending southward.

**Fig. 8:**
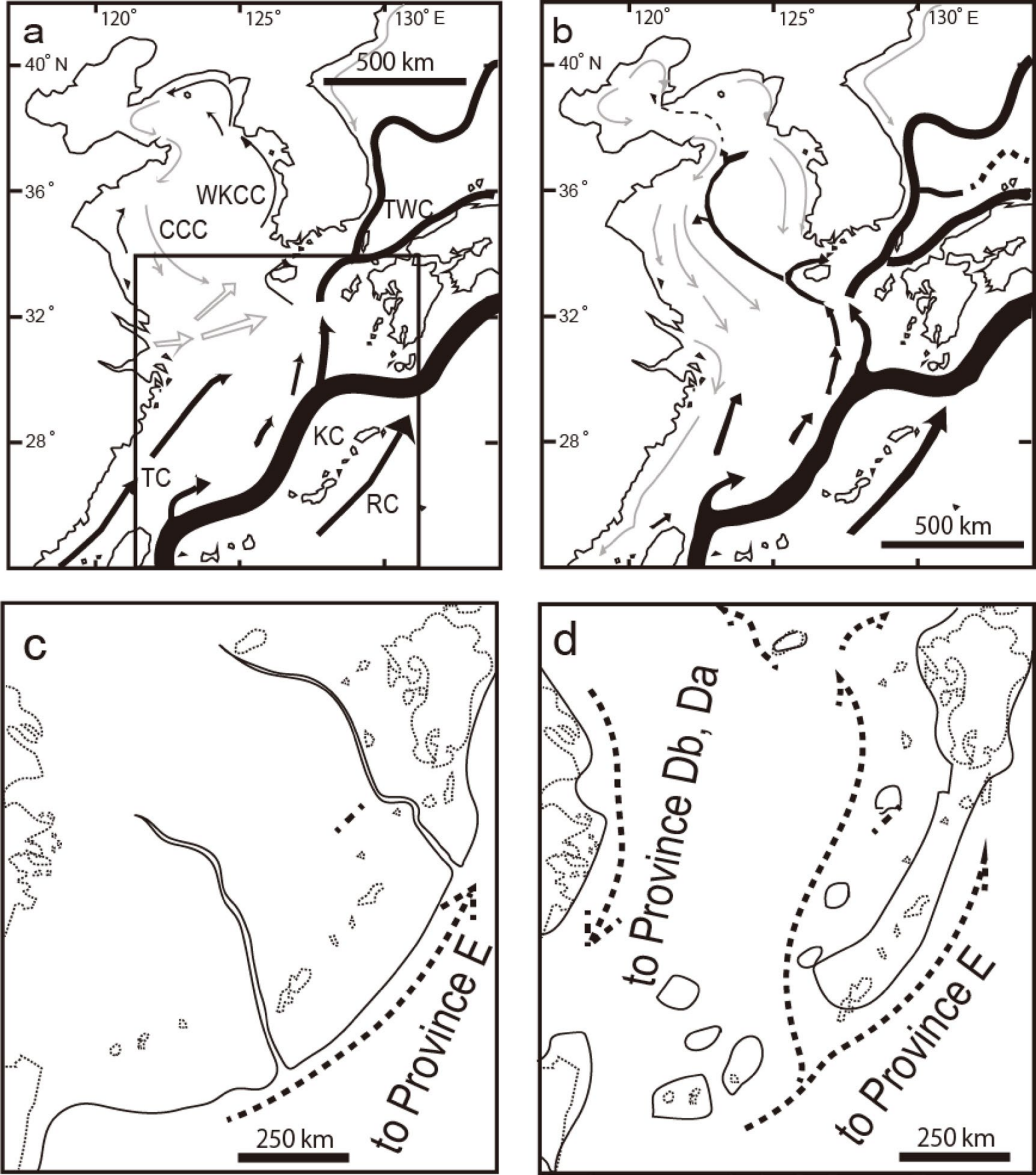
Recent distribution of oceanic and coastal currents, along with palaeogeography of East Asia. (**a**) Summer oceanic and coastal currents, (**b**) Winter oceanic and coastal currents after Guo et al.^25^; (**c**) Late Miocene palaeogeographical map and (**d**) Late Pliocene palaeogeographical map^26^, including estimated oceanic and coastal currents shown as broken lines; abbreviations are CCC = Chinese Coastal Current, KC = Kuroshio Current, RC = Ryukyu Current, TC = Taiwan Current, TWC = Tsushima Warm Current, WKCC = West Korea Coastal Current.

The oldest fossil record of three species, *Loxoconcha kattoi*, *Loxoconcha mutsuense*, and *Cornucoquimba tosaensis*, date back to the Late Miocene in the Ryukyu Islands. Two species, *L. kattoi* and *L. mutsuense*, migrated northward (Fig. 6a). Teeter^15^ suggested that the pan-oceanic dispersal of certain shallow-marine podocopid Ostracods occurs through transoceanic currents carried by drifting algae. East Asia features not only oceanic currents like the Kurosho Current, Tsushima Warm Current, Ryukyu Current, and Taiwan Current, but also coastal currents, such as the West Korean Coastal Current and the Chinese Coastal Current. These currents facilitate the easy dispersion of species around the coast of East Asia (Fig. 8a). Additionally, the West Korean Coastal Current and the Chinese Coastal Current reverse direction during the summer (Fig. 8a) and winter (Fig. 8b). For phytal species, the substrate they grow on is unstable and shows different ethology^7,16^. Furthermore, ocean currents carry away the substrate itself^15^. Compared to benthic species, which crawl or burrow on the surface of sand and mud^17^, they are thought to have a higher possibility of passive dispersal. Consequently, in the absence of physical or chemical barriers, coastal Ostracoda, both phytal and benthic species, can extend their distribution by utilizing longshore currents. The migration mechanisms are estimated to involve oceanic and coastal currents associated with floating algae for *L. mutsuense* or coastal currents combined with land bridges for *L. kattoi*, based on their current microhabitats^18^, based on their current microhabitats, namely on the leaves (eel glass and calcareous algae) and on the bottom sediment, respectively. (Figs. 7, 8b, c).

**Fig. 9:**
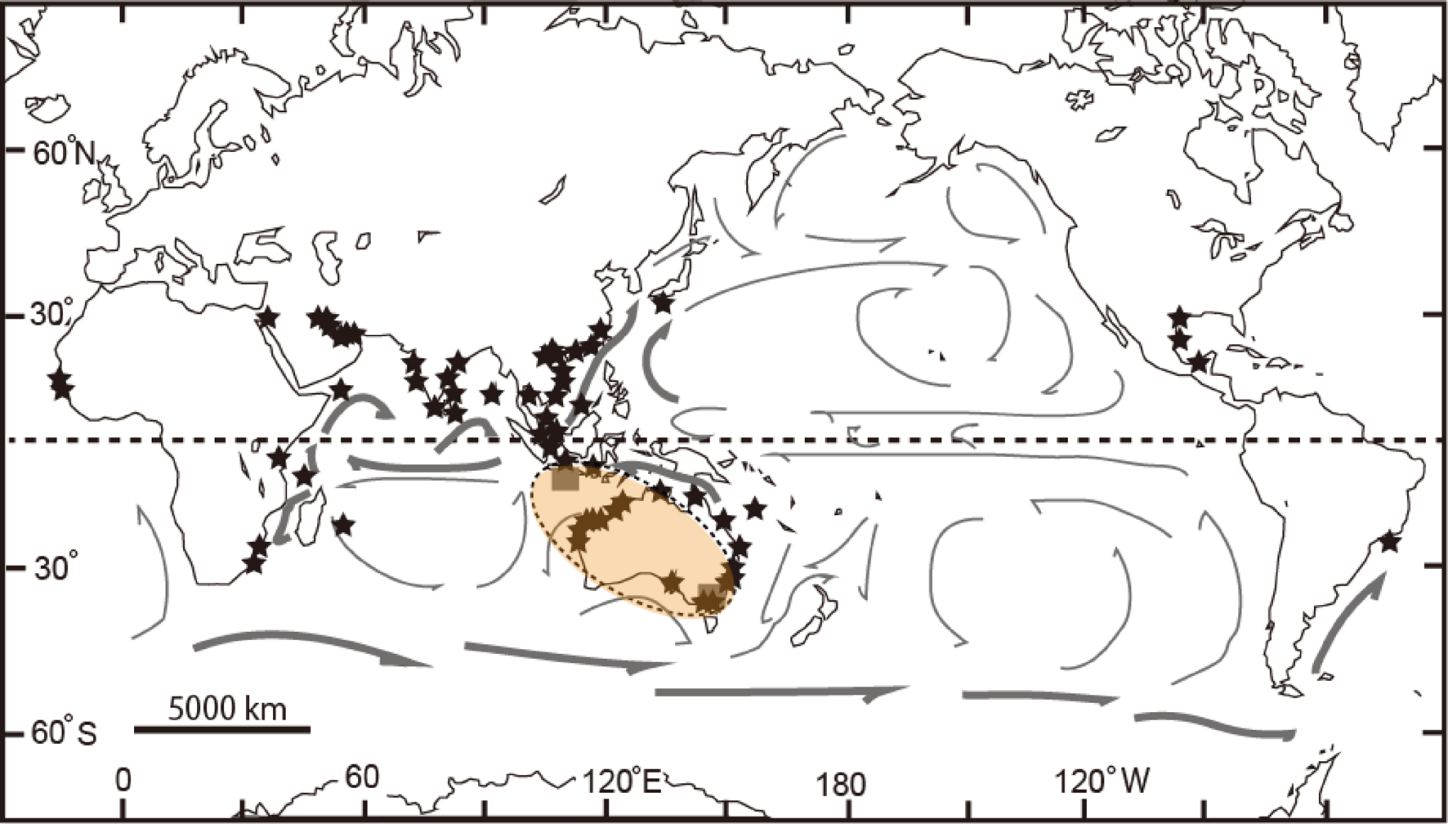
Biogeographical distribution of *Tanella gracilis* (black-filled star = recent, gray square = fossil) and oceanic currents^4^. The broken ellipse filled with light orange color indicates a possible origin inferred from fossil records. The bold oceanic current represents a likely dispersal route for *Tanella gracilis*.

From the Pliocene to the Pliocene–Pleistocene, 18 species have been identified as having the oldest fossil records (Fig. 6a). Of these, 12 species are estimated to have originated from the southern province, Province Da, Db, or the southern area of Province E (Fig. 6a). Among these, 10 species, including *Loxoconcha uranouchiensis*, *Aurila cymba*, *Loxoconcha zamia*, *Pistocythereis bradyformis*, *Nipponicythere bicarinata* are creepers on the bottom sediments^18^–^21^. This suggests their northward migration was facilitated by oceanic currents, such as the Taiwan Current, the Kuroshio Current, the Tsushima Warm Current and possibly by land bridge after the Middle Pliocene. Notably, *Loxoconcha uranouchiensis* is found in surface sediments around eelgrass^7^ (Fig. 7), indicating that its dispersal was possibly aided by oceanic current such as the China Coastal Current during the winter and/or glacial periods after the Pliocene (Figs. 8b, d). In contrast, *Aurila hataii* and *Loxoconcha japonica* are phytal species^16^, with their dispersal presumed to occur via oceanic and/or coastal currents carrying floating algae. An exception is *Tanella gracilis*, which has the oldest fossil records from southeast Australia and Indonesia and is now distributed worldwide from 30°S to 30°N (Fig. 9). However, living *Tanella gracilis* has been reported in mud to muddy fine sands^1^, suggesting its migration was facilitated by coastal currents and land bridges. Three species, *Loxoconcha hattorii*, *Finmarchinella uranipponica*, and *Cytheromorpha acupunctata*, are possibly of northern origin and later migrated southward (Fig. 6a). *Loxoconcha hattorii* and *Cytheromorpha acupunctata* are creeping on littoral and/or embayment bottom sediments^17,22^, and their passive dispersal by ocean currents is more difficult than that of phytal species. Therefore, their migration was likely accomplished by coastal or oceanic currents caused by a drop in sea level after the Late Pliocene (Fig. 6a).

Fifteen species appeared during the Pleistocene, with 12 of them restricted to Province E (Fig. 6b). Although the two species *Loxoconcha epeterseni* and *Xestoleberis hanaii* were more widely distributed toward Province Da, their origin lies in Province E, indicating a southward migration. *Xestoleberis hanaii* is commonly found on algae along rocky shores^23^ (Fig. 7), with its dispersal occurring via oceanic and coastal currents, suggesting a transoceanic distribution (Fig. 8b). During the Pleistocene-Holocene, the species *Perissocytheridea japonica* first appeared in Province Db and subsequently migrated to Province E. It inhabits beach sand in deep rock holes (> 50 cm)^24^, suggesting that its dispersal occurred via land bridges combined with coastal currents (Fig. 8a, b). During the Holocene, the 10 species emerged, with 9 species being restricted to Province E (Fig. 6b). *Cytherois nakanoumiensis* was found in Province Db. Although the microhabitat of the species is unknown, its biogeographical distribution and fossil records imply that its presence across the two provinces likely occurred over a period of 10,000 years. Research at the species-level (α taxonomy) of endemic species in Province Da and Province Db will help clarify the boundaries of Asian Ostracod biogeography.

## Conclusions

Of the 47 Ishizaki specimens examined, 24 species either have the oldest known fossil records or are found exclusively in Province E, both in fossil and modern contexts. Four species, *Sinoleberis tosaensis*, *Aurila tosaensis*, *Loxoconcha zamia*, and *Loxoconcha viva*, were originally recorded with their earliest fossils in Province Db but are now restricted to Province E. Fifteen species exhibit a broader biogeographical distribution, ranging from Province Da to Province E. Recent data on microhabitats, oceanic and coastal currents, palaeogeographical changes, and molecular phylogenetic analysis provide important insights into the formation of the Ostracod Province. These patterns significantly contribute to biodiversity studies by clarifying the historical dispersal of species, endemism, and regional diversification within marine Ostracods.

## Supplementary Information

Below is the link to the electronic supplementary material.

Supplementary Material 1

Supplementary Material 2

## Data Availability

All the illustrated specimens have been deposited in the Tohoku University Museum. All data supporting the findings of this study are available within the paper. This work is partially supported by the Ocean Shot Research Grant Program. This research does not fall under any national/international guidelines for sampling, care and experimental use of organisms for study.
