# Supplementary material for: Revisiting recent Ostracod type material from Ishizaki with insights into species distribution and taxonomic reassessment
Source: Sci Rep. 2025 Oct 3;15:34637. doi: 10.1038/s41598-025-22250-5 (PMC12494741; doi:10.1038/s41598-025-22250-5)
Supplement: Supplementary file 1 — Supplementary Material 1 [file 41598_2025_22250_MOESM1_ESM.docx]

**Supplementary Information 2** Recent and fossil records of type species described from Ishizaki (1968–1971). Each reference corresponds to the reference list of SI 1.

|  |  |  |  |  |  |  |  |  |  |  |  |  |  |  |
| --- | --- | --- | --- | --- | --- | --- | --- | --- | --- | --- | --- | --- | --- | --- |
|  | Species |  | Recent |  | Fossil |  |  |  |  |  |  |  |  | Microhabitat |
|  |  |  |  |  | L Miocene | E Pliocene |  |  | L Pliocene | Plio-Pleistocene | Pleistocene | Pleistocene-Holocene | Holocene |  |
|  | *Cytherelloidea munechikai* |  | SW Japan^1,8,9^ |  | - | - |  |  | - | - | - | - | - | - |
|  | *Neonesidea mutsuensis* |  | N Japan^3,8^, Peter the Great Bay^16^ |  | - | - |  |  | - | - | N Japan^13^ | - | Sea of Japan side C Japan^14^ | - |
|  | *Bythoceratina hanaii* |  | SW to central Japan^1,8,19,27,39^ |  | - | - |  |  | - | C Japan^33^ | N Ryukyu^37^, Sea of Japan side C Japan^33^ | - | - | - |
|  |  |  |  |  |  |  |  |  |  |  |  |  |  |  |
|  | *Tanella gracilis* |  | Septtiba Bay in Brazil^66,78^, Gulf of Mexico^48,51^, West Indies^80^, East Africa^51,52,55,75^, western Africa^56,76^, South Africa, Red Sea^50,54,98,102,114,128,137,140^, Persian Gulf^63,97,112^, Andaman Island^49,70,103,126^, coast of India^57,58,73,79,82,85,86,88-90,93-95,101,105,109-111,115,117,118,121-123^, Sri Lanka^119^, Thailand^,99,104,120,136^, Malacca Strait^68,74^, Malaysia^107,127^, Vietnam^108,132,133^, Java^87^, Bari^100^, Indonesia^51,116,124^, New Caledonia^84^, Australia^61,62,69,77,83^, South China^106^, Hainan Island^71^, China Sea^21,26,30,32,72^, Hong Kong^65^, SW Japan^1,8^ |  | - | S Australia^96^, Sumatra^45,60^ |  |  | Taiwan^106^ | - | New South Wales^64^ | Kalimantan^92^, Fujian^25^ | Bays of Texas^59^, S Iraq^91^, E India^53^, South Autsralia^81^, New Caledonia^84^, Hong Kong^34, 65,125^ | mud to muddy fine sand^133^ |
|  | *Loxoconcha epeterseni* |  | Hong Kong^158^, East China Sea^143^, Ulleung Basin^145^, SW &N Japan^1,8,12,27,40,144,151,153^ |  | - | - |  |  | - | - | Taiwan^17^, N Ryukyu^37^, Cheju Island^29^, C Honshu^33,152^ | Okinawa Trough^28^ | Hong Kong^34,65^ SW to N Japan^147,150,154,157^ | - |
|  | *Loxoconcha hattorii* |  | South to Middle China^28^, Shanghai^160^, Hubei^160^, Guangxi^160^, Hebei^161^, Matsu Islands^17^, Penghu Island^17^, Taiwan^17^, S Korea, SW to N Japan^3,8,12,36,41,153173,165^ |  | - | - |  |  | C Japan^134^ | - | Cheju Island^29^, SW & C Japan^13,33,36,162^ | - | Hong Kong^34,146^, SW & NE Japan^36,150,166,168^ | littoral sediment^167^ |
|  | *Loxoconcha japonica* |  | N vietnam^133^, Guangxi^72^, Penghu Islands^17^, South Korea^188^, SW & C Japan^1,8,27,36,41,144,151,153,169,170,173,178-185,188,189,193^ |  | - | S Taiwan^17^ |  |  | C Japan^164^ | Taiwan^17,176,177^, C Japan^33,188^ | Taiwan^17,23,172,174^, Okinawa Trough^28^, Ryukyu^186^, Cheju Island^29^, SW & C Honshu^188,198,203^ | Taiwan^17^, Okinawa Trough^28^, C & N Japan^188^ | Hong Kong^146^, SW & C Japan^14,36,154,188,199,200^ | Sea grass^178^ |
|  | *Loxoconcha kattoi* |  | SW Japan^188,27,35,36,39,40,151,153,183^ |  | Okinawa^196^ | Taiwan^17^, Okinawa^196^ |  |  | SW Japan^205^ | Taiwan^17,176,177^, C Japan^33,188^ | Cheju Island^29^, C Japan^152,204^ | - | SW & C Japan^14,199^ | Bottom^153^ |
|  | *Loxoconcha kitanipponica* |  | SW & N Japan^3,9^ |  | - | - |  |  | - | - | Kyushu, Japan^203^ | - | - | Bottom^153^ |
|  | *Loxoconcha mutsuense* |  | South Korea^167^, SW & N Japan^3,8,12,35,39,40,153,173,180,182,206^ |  | Okinawa^196^ | - |  |  | Okinawa^196^ | C Japan^33^ | S Taiwan^17^, Okinawa Trough^28^, Cheju Island^29^, N Ryukyu^37^, C Japan^207,21219^ | C Japan^209^ | S Taiwan^17^ | Phytal^153^ |
|  | *Loxoconcha optima* |  | SW & C Japan^1,8,12,40,144,188^ |  | - | - |  |  | - | - | C Japan^204,218,220^ | - | - | - |
|  | *Loxoconcha pulchra* |  | South Korea^239^, SW & C Japan^1,2,12,35,151,153,188,227,240^ |  | - | - |  |  | - | - | SW & C Japan^203,222,226^ | - | SW & C Japan^14,166,228,230,237,238^ | Bottom^153^ |
|  | *Loxoconcha tosaensis* |  | South Korea^167^, Tsushima^200^, SW & N Japan^1,3,8,35,36,39,151,153,163,212,242,244,246,248^ |  | - | - |  |  | - | - | SW & C Japan^152,162,198,203,218,241^ | - | Tsushima^200^, SW & N Japan^14,216,230,232,237,242,243,245,247,249,250^ | Bottom^153^ |
|  | *Loxoconcha uranouchiensis* |  | N Vietnam^133^, Guangxi^72^, Okinawa^256^, SW to N Japan^1-3,8,27,36,40,41,149,151,153,173,178-183,188,200,212,248,255,257,258^, Peter the Great Bay^156^ |  | - | - |  |  | Leizhou Peninsula^106^ | - | SW & C Japan^13,152,162,203,226,264^ | - | Tsushima, SW to N Japan^150,154,166,168,230,232,250,261,264^ | Bottom^153^ |
|  | *Loxoconcha viva* |  | SW & N Japan^1,3,8,151,248^ |  | - | - |  |  | - | - | - | Jiangsu^20^ | Okinawa Trough^28^, SW & C Japan^147,154,222,230,238,243,260^ | - |
|  | *Loxoconcha zamia* |  | SW Japan^1,8,36,153,183^ |  | - | - |  |  | Taiwan^17^ | - | N Ryukyu^37^ | - | SW Japan^14^ | Bottom^153^ |
|  | *Miia uranouchiensis* |  | SW Japan^1,8,12,151^ |  | - | - |  |  | - | - | C. Japan^12^ | - | - | - |
|  | *Cytheromorpha acupunctata* |  | Shanghai^158,160^, Hubei^160^, Hebei^161^, Shangdong^160^, South Korea^271^, Tsushima^200^, SW to N Japan^1-3,12,35,39-41,144,149,151,163,170,206,212,227,234,240,242,244,246,254,248,257,268-270,272,277,284^, Peter the Great Bay^16^ |  | - | - |  |  | - | C Japan^33^ | Cheju Island^29,31^, SW & C Japan^152,162,198,203,213,219,220,222,226,241,278,285^ | Jiangsu^20^, Hong Kong^34^, Bohai Sea^276^ | Okinawa Trough^28^, Ulleung Basin^280^, Tsushima^200^, SW to N Japan^42,147,150,154,157,166,168,207,230,238,242,243,245,247,250,260,261,264,275,281,282^ | Sand^278^ |
|  | *Nipponocythere bicarinata* |  | South China Sea^148^, Hong Kong^158^, East China Sea^287^, South Korea^7,271^, Tsushima^200^, Okinawa^186^, SW to N Japan^35,36,39,40,149,151,163,170,242,246,248,257,268,284^ |  | - | - |  |  | Taiwan^17^, Okinawa^196^ | - | Okinawa Trough, SW & C Japan^162,213,218,219,222,260,286,288^ | Bohai Sea^276^ | Hong Kong^146^, Ullung Basin^280^, Tsushima^200^, SW to N Japan^147,150,154,237,242,247,260,264^ | littoral <1m^289^ |
|  | *Anglicytherura miii* |  | SW to N Japan^2,3,8,41,212,227,234,240,281^ |  | - | - |  |  | - | - | - | - | SW Japan^14,166,230^ | Bottom sediment^227^ |
|  | *Howeina* *higashimeyaensis* |  | N Japan^3^ |  | - | - |  |  | - | - | - | - | - | - |
|  | *Cytherois asamushiensis* |  | N Japan^3,8,149^, Peter the Great Bay^156^ |  | - | - |  |  | - | - | Cheju Island^29^, C Japan^204^ | - | - | - |
|  | *Cytherois nakanoumiensis* |  | South China Sea^21^, SW & N Japan^2,3,8,41,151,163^ |  | - | - |  |  | - | - | - | - | SW & N Japan^42,247^ | - |
|  | *Cytherois uranouchiensis* |  | SW to N Japan^1-3,8,41,151,163,244^ |  | - | - |  |  | - | - | SW Japan^222^ | - | SW & N Japan^42,168,247^ | - |
|  | *Paracytherois mutsuensis* |  | SW & N Japan^3,8,151^ |  | - | - |  |  | - | - | - | - | - | - |
|  | *Paracytherois tosaensis* |  | SW to N Japan^1-3,8,257^ |  | - | - |  |  | - | - | S Taiwan^17^, C Japan^308^ | - | Okinawa Trough^28^ | - |
|  | *Xestoleberis hanaii* |  | E Malay Peninsula^324,326^, S Vietnam^133,202^, South China Sea^26,32^, SW to N Japan^1,3,8,12,27,36,41,144,149,151,180,182,183,206,248,258,312,313,319,320^, Kuril Islands^311^ |  | - | - |  |  | - | - | S Taiwan^17^, Cheju Island^29^, C & N Japan^13,152,155,203,218,220,308^ | Okinawa Trough^28^ | Hong Kong^146^, Tsushima Straits^168,200^, SW to N Japan^14,150,157,166,199,216,249,250^ | Algae of Rocky shore^313^ |
|  | *Krithe japonica* |  | Tsushima^200^, SW & N Japan^3,8,149,151,163,254^ |  | - | - |  |  | - | - | Cheju Island^29,31^, C Japan^218,219,222,241^ | - | Tsushima^200^, SW & C Japan^243,230,245,264^ | - |
|  | *Perissocytheridea japonica* |  | Shangha^160^i, Hebei^161^, Yellow Sea^160^, SW & C Japan^1,8,173,234,334^ |  | - | - |  |  | - | - | - | Jiangsu^20^ | C Japan^238^ | Beach sand^334^ |
|  | *Aurila cymba* |  | Vietnam^133^, South China Sea^21,32^, Hong Kong^170,151,268,269,315^, Yellow Sea^160^, Bohai Sea^273^, SW to N Japan^1,3,8,35,39,151,212,244,246,248^ |  | - | - |  |  | South China Sea^221^ | - | Taiwan^17^, Cheju Island^29^, SW to N Japan^13,152,162,198,203,222,226,266,308,340^ | Hong Kong^34^, Jiangsu^20^, Fujian^21^, Zhejiang^21^, Bohai Sea^276^ | Hong Kong^34,146^, Bohai Sea^276^, SW & C Japan^150,199,216,261,266^ | Sandy and rocky shores^339^ |
|  | *Aurila hataii* |  | N Vietnam^133^, South China Sea^21,32^, Hainan Island^71^, Guangxi^72^, SW to N Japan^1,3,8,12,27,36,173,149,183,258,339^, Kunashiri Island |  | - | - |  |  | Taiwan^17^, Okinawa^196^ | - | Taiwan^17^, Cheju Island^29,31^, SW & C Japan^203,288, 296^ | Hong Kong^20,34^ | Hong Kong^20,34,146^, SW Japan^14^ | Intertidal zone of rocky shore^173^ |
|  | *Aurila imotoi* |  | N Taiwan^17^, SW & C Japan^1,8^ |  | - | - |  |  | - | - | S Taiwan^17^, Cheju Island^31^ | - | Hong Kong^125^, N Japan^168^ | - |
|  | *Aurila munechikai* |  | South China Sea^21^, SW & C Japan^1,8,181,182^ |  | - | - |  |  | S Taiwan^17^, N Japan^208^ | - | S Taiwan^17^, Cheju Island^29^ | - | SW & C Japan^14,150^ | Phytal^180^ |
|  | *Aurila tosaensis* |  | SW & C Japan^1,8,12,35,40,183^ |  | - | - |  |  | Leizhou Peninsula^22^ | - | Cheju Island^29,31^ | - | N Japan^168^ | - |
|  | *Aurila uranouchiensis* |  | SW & N Japan^1,3,8^ |  | - | - |  |  | - | - | N Ryukyu^37^, SW Japan^162^ | - | - | - |
|  | *Pseudoaurila japonica* |  | SW Japan^1,8,39,183^ |  | - | - |  |  | - | - | N Ryukyu^37^, Cheju Island^29^, C Japan^342^ | - | SW Japan^14^ | - |
|  | *Caudites japonicus* |  | Ulleung Basin^145^, SW Japan to Hokkaido^3,8,333,348^ |  | - | - |  |  | - | SW Japan^33^ | - | - | - | Intertidal zone of rocky shore^333^ |
|  |  |  |  |  |  |  |  |  |  |  |  |  |  |  |
|  | *Cornucoquimba tosaensis* |  | N Taiwan^17^, Ulleung Basin^280,145^, Tsushima Straits^349^, SW Japan to Hokkaido^1-3,8,35,36,40,41,151,173,246,333^ |  | Okinawa^196^ | - |  |  | Leizhou Peninsula^22^, N Taiwan^17^, Okinawa^196^ | C Japan^33^ | Cheju Island^29,31^, SW & C Japan^162,218,222,340,345^ | - | S Taiwan^17^, Jiangsu^23,346^, SW & C Japan^14,199,261,282,345^ | - |
|  | *Finmarchinella uranipponica* |  | Ulleung Basin^280^, SW to N Japan^2,3,8,36,149,151,257^, Peter the Great Bay^156^ |  | - | - |  |  | N Japan^353^ | - | Cheju Island^29,31^, C & N Japan^208,213^ | - | SW & N Japan^14,247^ | - |
|  |  |  |  |  |  |  |  |  |  |  |  |  |  |  |
|  | *Urocythereis miii* |  | SW Japan^2,8,212^ |  | - | - |  |  | - | - | - | - | - | - |
|  | *Pistocythereis bradyformis* |  | E Malay Peninsula^362^, Vietnam^108^, Sunda Shelf^74^, Hong Kong^65^, South China Sea^21,26,30,32,148,363^, Penghu Islands^17^, SW Taiwan^17^, Hebei^161^, Bohai Sea^273,360^, East China Sea^148,160,287,357^, SW to N Japan^1,3,8,35,36,39,41,151,169,170,212,246,248,333^ |  | - | - |  |  | Leizhou Peninsula^22^, Taiwan^17^ | Taiwan^17^, C Japan^33^ | Taiwan^17^, Cheju Island^29,31^, SW & C Japan^162163,218,219,222,226,280,285,286,308^ | Hong Kong^34^, Okinawa Trough^28^, Fujiang^25,221^ | Hong Kong^34,125,178^, SW to N Japan^14,147,150,154,168,237,243,261,264,275^ | Subtidal zone of muddy shore^333^ |
|  | *Pistocythereis bradyi* |  | W Malay Peninsula^107,368s,369,372^, Malacca Strait^67,68^, Sunda Shelf^74^, South China Sea^21,26,32,314,359^, Hong Kong^65,158^, S Ryukyu^253,254^, Shandong^273^, SW to N Japan^1-3,8,35,36,39,40,151,183,212,242,244,246,248,257,358^, |  | - | - |  |  | Okinawa^196^ | Taiwan^17^, Okinawa^186^,^365^ | Taiwan^17^, Okinawa^186,365^, SW & C Japan^162,203,222,226,285,308,367^ | Hong Kong^34^, Bohai Sea^276,371^ | Laizhou Bay^371^, Tsushima Straits^200^, SW to N Japan^42,147,150,154,157,168,199,216,230,237,238,242,243,247,249,264^ | - |
|  | *Trachyleberis niitsumai* |  | South China Sea^21,32,148,359^, Hebei^161^, S Ryukyu, East China Sea^148,287^, Yellow Sea^148,374^, Tsushima Straits^349^, SW & N Japan^3,8,35,41,246,257,272,333,376^ |  | - | - |  |  | Leizhou Peninsula^22^, Taiwan^17^ | Taiwan^17,175^, Kyushu^377^ | Taiwan^17,23,174,176^, Okinawa Trough^28,31^, Cheju Island^29^, SW & C Japan^203,218,285^ | Jiangsu^20^, Fujiang^25^, Bohai Sea | Bohai Sea^276,338^, Kyushu, SW & C Japan^14,150,157,228,243,245,250,261,282,345^ | - |
|  | *Acanthocythereis mutsuensis* |  | N Japan^3,8^ |  | - | - |  |  | - | - | - | - | - | - |
|  | *Pacambocythere japonica* |  | SW & C Japan^1,8,35,36,41^ |  | - | - |  |  | - | SW Japan^33^ | Taiwan^23^ | - | C Japan^36^ | - |
|  | *Doratocythere tomokoae* |  | South China Sea^32,221^, Yellow Sea^160^, SW & C Japan^1,8,12,35,36,257^ |  | - | - |  |  | Leizhou Peninsula^22^, Taiwan^17^ | Taiwan^17,175,176^ | Taiwan^17,23,174^, C Japan^286^ | East China Sea^347^ | SW Japan^14,216^ | - |
|  | *Sinoleberis tosaensis* |  | Penghu Islands^17^, W & N Taiwan^17,382^, SW & C Japan^1,8,40,258^ |  | - | - |  |  | Taiwan^17,383^ | Taiwan^17,283^ | Taiwan^17,23,176^, Cheju Island^29,31^, C Japan^204,218,286^ | - | - | - |
|  |  |  |  |  |  |  |  |  |  |  |  |  |  |  |
|  |  |  |  |  |  |  |  |  |  |  |  |  |  |  |
