# Supplementary material for: Revisiting recent Ostracod type material from Ishizaki with insights into species distribution and taxonomic reassessment
Source: Sci Rep. 2025 Oct 3;15:34637. doi: 10.1038/s41598-025-22250-5 (PMC12494741; doi:10.1038/s41598-025-22250-5)
Supplement: Supplementary file 2 — Supplementary Material 2 [file 41598_2025_22250_MOESM2_ESM.docx]

**Supplementary Information 1** Synonym list of type specimens described by Ishizaki (1968–1971)^1-3^. An asterisk (*) shows the type specimen, (v.) is a valid specimen, (v.p.) includes a partly valid specimen, and (non.) means a non-valid specimen identified by the authors from each reference.

Phylum: Arthropoda

Class: Ostracoda Latreille, 1802^4^

Order: Platycopa Sars, 1866^5^

Family: Cytherellidae Sars, 1866^5^

Genus: *Cytherelloidea* Alexander, 1929^6^

Type Species: *Cytherella williamsoniana* Jones, 1849^7^

1. *Cytherelloidea munechikai* Ishizaki, 1968^1^

v*. 1968 *Cytherelloidea munechikai* Ishizaki, p. 443, pl. 8, figs. 1-3^1^.

v. 1977 *Cytherelloidea munechikai* Ishizaki; Hanai et al., p. 15^8^.

v. 2003 *Cytherelloidea munechikai* Ishizaki; Yamaguchi and Endo, p. 28, fig. 4l^9^.

**Type:** Right valve (Holotype), IGPS 90324

**Occurrence:** *Recent*: Shikoku, Southwest Honshu, Japan. *No fossil record*.

Order: Podocopida Sars, 1866^5^

Family: Bairdiidae Sars, 1866^5^

Genus: *Neonesidea* Maddocks, 1969^10^

Type Species: *Triebelina schulzi* Hartmann, 1964^11^

**2.** *Neonesidea mutsuensis* (Ishizaki, 1971)^3^

v*. 1971 *Bairdia (Neonesidea) mutsuensis* Ishizaki, p. 77, pl. 11, fig. 9, pl. 2, figs. 6-8^3^.

v. 1977 *Neonesidea mutsuensis* (Ishizaki); Hanai et al., p. 18^8^.

non. 1985 *Neonesidea mutsuensis* (Ishizaki); Ikeya et al., pl. 1, fig. 1^12^.

v. 1985 *Neonesidea mutsuensis* (Ishizaki); Ishizaki and Matoba, pl. 5, fig. 7^13^.

v.1993 *Neonesidea mutsuensis* (Ishizaki); Kamiya and Nakagawa., p. 123, pl. 1, fig. 3^14^.

v. 2006 *Neonesidea mutsuensis* (Ishizaki); Schornikov and Zanina, p.214, table 2^15^.

2006 *Neonesidea mutsuensis* (Ishizaki); Schornikov, p.33^16^.

non. 2008 *Neonesidea mutsuensis* (Ishizaki); Hu and Tao, p.57-58, pl. 23, figs. 3.4, pl. 32, figs. 2, 7, 10,16, pl. 125, figs. 5, 12, pl. 183, figs. 10, 14, 16, pl. 193, figs. 2, 3, 8, 10, pl. 205, figs. 12, 13, pl. 220, figs. 2, 3, 5^17^.

**Type:** Carapace (Holotype), IGPS 90334

**Remarks:** *Neonesidea mutsuensis*, as reported by Ikeya et al.^12^ from the Omaezaki coast in Central Japan, differs from the type specimen by having a widely arched dorsal margin, an elongated lateral outline, and less sharped caudal process. *Neonesidea mutsuensis*, as illustrated by Hu and Tao^17^ from the Late Pliocene Tungshiao Formation in Northwest Taiwan, the Pleistocene Hengchun Limestone in Southern Taiwan, recent sediments from Northeast Taiwan, the recent sediments from Penghu Islands and Kinmen Island in West Taiwan, differs from the type specimen by having a lower height, an elongated posterior process, and a longer dorsal margin.

**Occurrence:**

*Recent*: Aomori Bay, Northern Honshu, Japan; Peter the Great Bay, Russia.

*Fossil*: Pleistocene Northern Honshu, Japan; Holocene Japan Sea of Central Japan.

Family: Bythocytheridae Sars, 1866^5^

Genus: *Bythoceratina* Hornibrook, 1952^18^

Type Species: *Bythoceratina mestayerae* Hornibrook, 1952^18^

**3.** *Bythoceratina hanaii* Ishizaki, 1968^1^

v*. 1968 *Bythoceratina hanaii* Ishizaki, p. 17, pl. 1, fig. 3, pl. 3, figs. 9, 10^1^.

v. 1977 *Bythoceratina hanaii* Ishizaki; Hanai et al., p. 53^8^.

v. 1981 *Bythoceratina hanaii* Ishizaki; Schornikov, p. 90-91, pl. 1, figs. 1, 2, pl. 3, figs. 9, 10, text-figs. 26A, 26B^19^.

non.1982 *Bythoceratina hanaii* Ishizaki; Hou et al., p. 159-160, pl. 71, figs. 21, 22, text-fig. 23^20^.

1982 *Bythoceratina hanaii* Ishizaki; Cai, p. 5, pl. 2, figs. 6, 7^21^.

non. 1983 *Bythoceratina hanaii* Ishizaki; Gou et al., p. 20, pl. 3, fig. 25^22^.

1985 *Bythoceratina hanaii* Ishizaki; Ikeya et al., pl. 5, fig. 8^12^.

non. 1986 *Bythoceratina hanaii* Ishizaki; Hu, p. 143, pl. 26, figs. 1-12, 14^23^.

non. 1987 *Bythoceratina hanaii* Ishizaki; Cai and Chen, pl. 2, fig. 8^24^.

non.1987 *Bythoceratina hanaii* Ishizaki; Zheng, p. 195, pl. 2, figs. 15, 18, 19^25^.

non.1988 *Bythoceratina hanaii* Ishizaki; Cai, pl. 4, fig. 20^26^.

v. 1988 *Bythoceratina hanaii* Ishizaki; Ikeya and Shiozaki, p. 135, pl. 2, fig. 8^27^.

non.1988 *Bythoceratina hanaii* Ishizaki; Ruan and Hao, p. 258, pl. 41, figs. 17-19^28^.

non. 1990 *Bythoceratina hanaii* Ishizaki; Lee, p. 310-311, pl. 26, figs. 5, 6^29^.

non.1991 *Bythoceratina hanaii* Ishizaki; Cai, p. 102, pl. 2, fig. 17-19^30^.

non. 1992 *Bythoceratina hanaii* Ishizaki; Lee and Paik, p. 153, pl. 4, fig. 14^31^.

non. 1994 *Bythoceratina hanaii* Ishizaki; Zheng et al., pl. 47, figs. 9, 10^32^.

v. 1996 *Bythoceratina hanaii* Ishizaki; Ozawa, p.108, pl. 2, fig. 4^33^.

non. 1998 *Bythoceratina hanaii* Ishizaki; Cao, pl. 2, fig. 8^34^.

non. 1998 *Bythoceratina hanaii* Ishizaki; Yamane, p. 39, pl. 2, fig. 7^35^.

2001 *Bythoceratina hanaii* Ishizaki; Kamiya et al., p. 95, fig. 14.9^36^.

v. 2004 *Bythoceratina hanaii* Ishizaki; Irizuki, p. 75, pl. 2, fig. 7^37^.

2004 *Bythoceratina hanaii* Ishizaki; Yamauchi, p. 73^38^.

v. 2006 *Bythoceratina hanaii* Ishizaki; Irizuki et al., p. 24, fig. 7.8^39^.

non. 2012 *Bythoceratina hanaii* Ishizaki; Tanaka et al., p. 9, pl. 1, fig. 5^40^.

non. 2014 *Bythoceratina hanaii* Ishizaki; Masuma and Yamada, p. 5, fig. 3.7^41^.

non. 2015 *Bythoceratina hanaii* Ishizaki; Yamada et al., p. 56, fig. 3.3^42^.

**Type:** Left valve (Holotype), IGPS 90208.

**Remarks:** *Bythoceratina hanaii*, as illustrated by Cai^21^ from the South China Sea, is not this species, as it features a protruding anterior margin, a longer ala, and a straight dorsal margin. *Bythoceratina hanaii*, as depicted by Hou et al.^20^ from the Quaternary of Jiangsu, China, differs from the type specimen in having an antero-ventrally arched anterior margin, a posteriorly elongated caudal process, and a straight dorsal margin. *Bythoceratina hanaii,* reported by Gou et al.^22^ from the Pliocene strata of the Leizhou Peninsula, South China, differs from the type species by having an acutely arched anterior margin, a sulcate anterior area, and a straight antero-ventral ridge. Hu^23^ illustrated this species from the Pleistocene Tungshao Formation in Taiwan. However, these specimens differ from Ishizaki’s type by having a sinuate anterior marginal carinal ridge, an antero-ventrally arched anterior margin, and a long dorsal carinal ridge. Cai and Chen^24^, Cai^26,30^, and Zheng et al.^32^ reported this species from the recent South China Sea and Nansha Islands. However, their species displays an acutely arched anterior margin, a smooth surface, and an ala parallel to the ventral margin. Zheng^25^ identified this species from Quaternary sediment along the coast of Fujian, China; however, their species has a widely arched dorsal carinal ridge, a shorter anterior carinal ridge, and an ala positioned one-third of the way from the venter. Ruan and Hao^28^ identified this species from Upper Pleistocene and Holocene cores around the Ryukyus, Japan; however, their species differs from the type specimen in having a longer ala, a curved carinal ridge at the anterior area, and a widely arched dorsal margin. Lee^29^ and Lee and Paik^31^ noted that their specimens differ from the type specimen in having an ala positioned more dorsally, an antero-ventrally arched anterior margin, and an inflated area in front of the middle sulcus. *Bythoceratina hanaii,* as figured by Cao^34^*,* belongs to *Coquimba* instead of *Bythoceratina*. Tanaka et al.^40^ reported this species from Suruga Bay, Central Honshu, Japan; however, their specimen differs in having a robust carinal ridge, a shallower central sulcus, and a straight dorsal margin. The specimen figured by Yamane^35^ from the Recent sediment of Hiuchi-nada, Seto Inland Sea, Southwest Japan, displays an undulate carinal ridge along the anterior margin, a more dorsally located ala, and an anterior margin protruding antero-ventrally. *Bythoceratina hanaii*, as reported by Masuma & Yamada^41^ and Yamada et al^42^ from Kumihama Bay and Holocene borehole cores from Nakaumi in Southwest Japan, respectively, is not identified as this species due to its inflated ventral area. Hu and Tao^17^ established a new genus, *Bubuloceratina*, with the genotype *B. bella* (Hu, 1977)^43^ from the Pleistocene Toukoshan Formation in Miaori, Northwestern Taiwan. *Bythoceratina hanaii* Ishizaki, 1968^1^ (except for fig. 9) was synonymized with *B. bella*. However, *B. bella* differs from *B. hanaii* in having a more undulated dorsal margin, a narrowly arched anterior margin, and less prominent ala.

**Occurrence:**

*Recent*: East China Sea near Goto Islands, Kyushu, Japan; Kyushu, Shikoku, Southwest and Central Japan.

*Fossil*: Plio-Pleistocene Central Honshu, Japan; Early Pleistocene Northern Ryukyu, Tanegashima Island, and Japan Seaside of Central Honshu, Japan.

Family: Leptocytheridae Hanai, 1957^44^

Genus: *Tanella* Kingma, 1948^45^

Type Species: *Tanella gracilis* Kingma, 1948^45^

**4.** *Tanella gracilis* Kingma, 1948^45^

v*. 1948 *Tanella gracilis* Kingma, p. 88-89, pl. 10, fig. 7^45^.

1955 *Tanella gracilis* Kingma; Howe, p. 181^46^.

v. 1963 *Tanella gracilis* Kingma; Morkhoven, p. 117, Figs. 172, 173^47^.

v. 1966 *Tanella gracilis* Kingma; Morales, pl. 7, figs. 1a-1c^48^.

v. 1968 *Leptocythere* ? *tosaensis* Ishizaki, p. 26-27, pl. 1, fig. 12, pl. 5, figs. 19, 20^1^.

1970 *Tanella gracilis* Kingma; Guha, p. 209^49^.

v. 1971 *Tanella* cf. *gracilis* Kingma; Bate, p. 247, pl. 1, figs. 1.ll, 2.ll, p. 249, pl. 2, figs. 1.ll, 3.ll^50^.

v. 1973 *Tanella gracilis* Kingma; Teeter, pl. 2, figs. l-p^51^.

v. 1974 *Tanella africana*; Hartmann, p. 267-268, p. 401, pl. 33, figs. 243-151, p. 402, pl. 34, figs. 252-256, pl. 150, fig. 3^52^.

v. 1976 *Tanella gracilis* Kingma; Jain, pl. 2, figs. g-I^53^.

v. 1976 *Tanella seminis* Bonaduce, Masoli and Pugliese, pl. 4, figs. 1-5^54^.

v. 1977 *Callistocythere tosaensis* (Ishizaki); Hanai et al., p. 34^8^.

v. 1977 *Tanella* aff. *gracilis* Kingm; Paik, p. 43, pl. 2, figs. 35-37^55^.

v. 1978 *Tanella gracilis* Kingma; Hartmann-Schröder and Hartmann, p. 172, figs. 108-113^56^.

v. 1978 *Tanella gracilis* Kingma; Jain, p. 93, pl. 2, figs. 2.J2-2., J4^57^.

v. 1979 *Tanella indica* Annapurna and Rama Sarma, p. 149-150, figs. 1, 2^58^.

v. 1979 *Tanella* sp., Garbett and Maddocks, p. 865, spl. 1, figs. 9, 10^59^.

v. 1979 *Tanella gracilis* Kingma; Keij, pl. 1, figs. 7, 8^60^.

v. 1980 *Tanella gracilis* Kingma; Hartmann, p. 178, fig. 47, pl. 5, figs. 11-16^61^.

v. 1981 *Tanella* cf. *gracilis* Kingma; Hartmann, p. 134, figs. 4, 5, pl. 3, figs. 7-14^62^.

v. 1982 *Jainella karwarensis* Bhatia and Kumar; Al-Abdul- Razzaq et al., p. 67, fig. 7^63^.

v. 1982 *Tanella gracilis* Kingma; Cai, pl. 4, fig. 6^21^.

v. 1984 *Tanella gracilis gracilis* Kingma; McKenzie and Pickett, p. 237, figs. 4N^64^.

v. 1984 *Tanella gracilis minor*, McKenzie and Pickett, p. 237, figs. 4M, p. 240, figs. 6J-6L^64^.

v. 1985 *Tanella gracilis* Kingma; Li, pl. 1, fig. 7^65^.

v. 1987 *Tanella optima* Chen; Zheng, pl. 5, figs. 16, 17^25^.

v. 1988 *Tanella gracilis* Kingma; Cai, pl. 1, fig. 15^26^.

v. 1988 *Tanella* sp., Dias-Brito et al., p. 479, pl. 1, fig. 13^66^.

v. 1988 *Tanella gracilis* Kingma; Whatley and Zhao, p. 6, pl. 6, figs. 5, 6^67^.

v. 1988 *Tanella gracilis* Kingma; Zhao and Whatley, p. 165, pl. 1, fig. 13^68^.

v. 1989 *Tanella gracilis darwini* Howe and McKenzie, p. 21, fig. 93^69^.

1989 *Tanella gracilis* Kingma; Howe and McKenzie, p. 31^69^.

v. 1989 *Tanella gracilis* Kingma; Whatley and Keeler, p. 79, pl. 3, fig. 17, p. 81, pl. 4, figs. 1-3^70^.

v. 1990 *Tanella gracilis darwini* Gou, p. 26, pl. 3, fig. 36^71^.

v. 1990 *Leptocythere*? *tosaensis* Ishizaki; Ruan, p. 133, pl. 1, fig. 22^72^.

v. 1991 *Tanella gracilis* Kingma; Cai, p. 116, pl. 4, fig. 22^30^.

v. 1991 *Tanella gracilis* Kingma; Sreenivas et al., pl. 1, fig. 7^73^.

v. 1992 *Tanella gracilis* Kingma; Mostafawi, p. 141, pl. 2, fig. 40^74^.

v. 1993 *Tanella gracilis* Kingma; Jellinek, p. 179, pl. 7, figs. 145–150^75^.

v. 1993 *Tanella gracilis* Kingma; Witte, p. 59, pl. 4, figs. 13–15^76^.

v. 1993 *Tanella gracilis gracilis* Yassini et al., p. 389, pl. 3, figs. 48-52^77^.

v. 1993 *Tanella gracilis carpentariaensis* Yassini et al., p. 388, pl. 3, figs. 53-55^77^.

v. 1994 *Tanella gracilis* Kingma; Coimbra et al., p.92, 94, pl. 2, figs. 5-13^78^.

1994 *Tanella gracilis* Kingma; Vaidya and Mannikeri, Table 1^79^.

v. 1994 *Tanella gracilis* Kingma; Zheng et al., pl. 59, fig. 3^32^.

v. 1995 *Tanella gracilis* Kingma; Babinot and Kouyoumontzakis, p. 23, pl. 1, fig. 12^80^.

1995 *Tanella gracilis* Kingma; Bell et al., Table 1^81^.

1995 *Tanella gracilis* Kingma; Shyam Sunder et al., p. 473^82^.

v. 1995 *Tanella gracilis* Kingma; Yassini and Jones, p. 334, figs. 239, 240^83^.

1996 *Tanella gracilis* Kingma; Babinot and Degaugue-Michalski, p. 361^84^.

1996 *Tanella gracilis* Kingma; Hussain and Rajeshwara, p. 44^85^.

1996 *Tanella gracilis* Kingma; Hussain et al., p. 79-80^86^.

v. 1997 *Tanella gracilis* Kingma; Dewi, p. 68, fig. 147^87^.

v. 1997 *Tanella gracilis* Kingma; Kumar and Hussain, pl. 1, fig. 7^88^.

v. 1997 *Tanella gracilis* Kingma; Naidu et al., pl. 5, fig. 5^89^.

v. 1998 *Tanella gracilis* Kingma; Hussain, p. 6, pl. 1, fig. 18^90^.

v. 1998 *Tanella gracilis* Kingma; Cao, pl. 5, fig. 1^34^.

v. 1999 *Tanella gracilis* Kingma; Al-Jumaily and Al-Sheikhly, p. 229, fig. 16^91^.

v. 1999 *Tanella gracilis* Kingma; Carbonel et al., p. 129, pl. 4, fig. 9^92^.

v. 2000 *Tanella gracilis* Kingma; Hussain and Mohan, pl. 1, fig. 10^93^.

v. 2001 *Tanella gracilis* Kingma; Mohan et al., p. 3, pl. 1, fig. 18^94^.

v. 2002 *Tanella gracilis* Kingma; Sridhar et al., p.23, pl. 2, fig. 2^95^.

v. 2002 *Tanella gracilis* Kingma; Warne, fig. 2F^96^.

v. 2003 *Tanella gracilis* Kingma; Mostafawi, p. 57, fig. 12^97^.

v. 2004 *Tanella gracilis* Kingma; Helal and Abd El-Wahab, p. 84, pl. 1, fig. 6^98^.

v. 2004 *Tanella gracilis* Kingma; Montenegro et al. p. 233, pl. 2, fig. 10^99^.

v. 2005 *Tanella ochracea* (Brady); Mostafawi et al., p. 131, pl. 2, fig. 1^100^.

v. 2006 *Tanella gracilis* Kingma; Bhandari and Singh, p. 662, pl. 2, fig. 8^101^.

v. 2006 *Tanella gracilis* Kingma; Helal and Abd El-Wahab, pl. 2, fig. 8^102^.

v. 2006 *Tanella gracilis* Kingma; Hussain et al., figs. 6.5, 6.15^103^.

2006 *Tanella gracilis* Kingma; Pugliese et al., Table 1^104^.

v. 2007 *Tanella gracilis* Kingma; Gopalakrishna et al., pl. 1, fig. 14^105^.

v. 2007 *Tanella gracilis* Kingma; Hou and Gou, pl. 148, figs. 4–7^106^.

v. 2007 *Tanella gracilis* Kingma; Nor Faiz et al., figs. 2.10, 2.11^107^.

v. 2009 *Tanella gracilis* Kingma; Tanaka et al., p. 377, pl. 1, fig. 3^108^.

v. 2010 *Tanella gracilis* Kingma; Elumalai et al., p. 16-18, pl. 2, fig. 1^109^.

v. 2010 *Tanellsa gracilis* Kingma; Ganesan and Hussain, p. 109, fig. 3c^110^.

v. 2010 *Tanella gracilis* Kingma; Hussain et al., p. 517, fig. 2i^111^.

2010 *Tanella gracilis* Kingma; Mostafawi et al., p. 265, appendix 1^112^.

2011 *Tanella gracilis* Kingma; Tanaka et al., appendices 1-3^113^.

2012 *Tanella gracilis* Kingma; Helal and Abd El-Wahab, fig. 5^102^.

v. 2012 *Tanella gracilis* Kingma; Mohammed and Keyser, p. 293, figs. 7, 97–100^114^.

v. 2013 *Tanella gracilis* Kingma; Baskar et al., p. 203, fig. 3.5^115^.

2013 *Tanella gracilis* Kingma; Fauzielly et al., table 2^116^.

v. 2013 *Tanella gracilis* Kingma; Hussain et al., p. 203, fig. 3e^117^.

v. 2013 *Tanella gracilis* Kingma; Hussain et al., p. 216, pl. 1, fig. 10^118^.

v. 2014 *Tanella gracilis* Kingma; Iwatani et al., text-fig. 4.19^119^.

v. 2014 *Tanella gracilis* Kingma; Yamada et al., fig. 4I^120^.

v. 2015 *Tanella gracilis* Kingma; Mahalakshmi and Hussain, p. 273, pl. 1^121^.

v. 2015 *Tanella gracilis* Kingma; Mohammed Nishath et al., pl. 1^122^.

v. 2016 *Tanella gracilis* Kingma; Hussain et al., fig. 4, table 3^123^.

2017 *Tanella gracilis* Kingma; Dewi et al., p. 5, table 1^124^.

v. 2017 *Tanella gracilis* Kingma; Hong et al., p. 58, fig. 8.38^125^.

v. 2017 *Tanella gracilis* Kingma; Hussain, p. 153, pl. 1, fig. 7s^126^.

v. 2017 *Tanella gracilis* Kingma; Noraswana et al., p. 87, fig. 2f^127^.

v. 2018 *Tanella gracilis* Kingma; Yousef, p. 66-67, figs. 6e, 6f^128^.

2018 *Tanella gracilis* Kingma; Wang et al., appendix 1^129^.

2018 *Tanella gracilis* Kingma; Hussain et al., table 2^130^.

2019 *Tanella gracilis* Kingma; Mishra et al., p. 118, table 4^131^.

v. 2019 *Tanella gracilis* s.l. Kingma; Niiyama et al., p. 696, fig. 2.8^132^.

v. 2019 *Tanella gracilis* s.l. Kingma; Niiyama et al., p. 104, fig. 3.13^133^.

2019 *Tanella gracilis* Kingma; Sridhar et al., tables 1, 4^134^.

2020 *Tanella gracilis* Kingma; Rajkumar et al., p. 5, table 2^135^.

v. 2021 *Tanella gracilis* Kingma; Forel, p.8, fig. 3T^136^.

v. 2021 *Mediocytherideis (Sylvestra) seminis* (Bonaduce et al., 1976); Keyser and Mohammed, p. 8, pl. 3, figs. 45, 46^137^.

2021 *Tanella gracilis* Kingma; Tan et al., supplement, table S5^138^.

2022 *Tanella gracilis* Kingma; Radhakrishnan et al., p. 232, table 1^139^.

v. 2022 *Tanella gracilis* Kingma;Yousef, fig. 6.37^140^.

**Type:** Left and right valves, Mineralogisch-Geologisch Institut of the State University of Utrecht (D.31987–8) by Kingma^45^.

**Remarks:** The new species *Leptocythere*? *tosaensis*, proposed by Ishizaki^1^, has been determined to be a junior synonym of *Tanella gracilis* Kingma^45^, as evidenced by the SEM image of the lectotype specimen illustrated by Keij^60^. This species was reported from the Early Pliocene Serula Formation in North Sumatra, Indonesia. Similarly, *Tanella indica* Annapurna and Rama Sarma^58^, is also a junior synonym of *T*. *gracilis* due to its ornamental patterns and shell outline. *Tanella gracilis darwini* Howe and McKenzie^69^, from northwest Australia, was initially classified as a subspecies based solely on shell size. However, since its shell outline and ornamentation match those of the type species, it has been included in *Tanella gracilis*. *Tanella gracilis carpentariaensis* Yassini et al.^77^, from northwestern Australia, was categorized as a subspecies due to the lack of ornamentation and postero-ventral swelling. Since the lack of ornamentation results from poorly developed calcification and the postero-ventral swelling is observed in female specimens, this subspecies has also been included in *Tanella gracilis*. Hussain et al.^86^ reported that *T. gracilis* inhabits water temperatures of 31.8°C–33.5°C, salinities of 33.7–34.9, and dissolved oxygen levels greater than 6.1 g/l in the southern part of India.

**Occurrence:**

*Recent*: Sepetiba Bay, Brazil; Laguna de Terminos, Gulf of Mexico; Saint Lucia, South Africa; Costa Do Sol, Lourenco Marques, Mozambique; Reunion Island, East Africa; Kenia East Africa; Mayotte Island; Senegal to Gambia; Abu Dhabi Lagoon, Persian Gulf; Red Sea, Egypt; Kuwait Bay; Al-Hudeida, Northern Socotra Island, Yemen; Strait of Hormuz and Gulf of Oman, Northern Arabian Sea; Derby, Broome, Port Hedland, Port Samson, Dampier, Exmouth, Carnarvon, and the Western, Northern, Northeastern, and Eden to Heron Islands of Eastern Australia; Andaman Islands; Northwestern Gulf of Thailand, Thailand; West, Southeastern, and East coast of India; Trincomalee Bay, Sri Lanka; Malacca Straits; coast of Malaysia; Southern Vietnam; Java Sea, Bari, Indonesia; Sunda Shelf; Chesterfield Islands, Northern New Caledonia; Spratly Islands, South China Sea; Hainan Island, Gaode and Weizhou Islands of Guangxi, China; South China Sea; Hong Kong, China; Shikoku, Japan.

*Fossil*: Early Pliocene of Southeast Australia, Sumatra; Late Pliocene of Taiwan; Late Pleistocene of New South Wales, Eastern Australia; Quaternary borehole core from Kalimantan, Indonesia; Quaternary borehole cores from Fujian, China; Holocene from Bays of Texas; Holocene from Southern Iraq; Chilka Lake, Eastern India; Holocene sediments from New Caledonia; Holocene core from South Australia; Quaternary borehole core from Hong Kong, China.

Family: Loxoconchidae Sars, 1926^141^

Genus: *Loxoconcha* Sars, 1866^5^

Type Species: *Cythere rhomboidea* Fischer, 1855^142^

**5.** *Loxoconcha epeterseni* Ishizaki, 1981^143^

v. 1961 *Loxoconcha* sp., Hanai, p. 371, text-fig. 12, fig. 3a, b^144^.

v*. 1968 *Loxoconcha laeta* Ishizaki, p. 29-30, pl. 1, fig. 14, pl. 6, figs. 3, 4^1^.

v. 1968 *Loxoconcha modesta* Ishizaki, p. 30, pl. 1, fig. 15, pl. 8, figs.11, 12^1^.

v. 1977 *Loxoconcha (Loxoconcha)* *laeta* Ishizaki; Hanai et al., p. 61-62^8^.

v. 1981 *Loxoconcha epeterseni* Ishizaki, p. 65^143^.

v. 1981 *Loxoconcha tosamodesta* Ishizaki, p. 65^143^.

non. 1982 *Loxoconcha modesta* Ishizaki; Cai, p. 7, pl. 2, figs. 31, 32^21^.

v. 1985 *Loxoconcha modesta* Ishizaki; Ikeya et al., pl. 8, figs. 1-3^12^.

non. 1986 *Loxoconcha (Loxoconcha)* *laeta* Ishizaki; Cheong et al., p. 48, pl. 3, fig. 18^145^.

non. 1987 *Loxoconcha hattorii* Ishizaki; Wang and Zhang, p. 290, pl. 1, fig. 19^146^.

v. 1987 *Loxoconcha laeta* Ishizaki; Ikeya et al., p. 63, fig. 2^147^.

v. 1988 *Loxoconcha modesta* Ishizaki; Ikeya and Shiozaki, p. 139, pl. 4, fig. 3^27^.

v. 1988 *Loxoconcha laeta* Ishizaki; Ikeya and Shiozaki, p. 139, pl. 4, fig. 5^27^.

v. 1988 *Loxoconcha kattoi* Ishizaki; Ruan and Hao, p. 323-333, pl. 57, figs. 14-16^28^.

v.p. 1990 *Loxoconcha laeta* Ishizaki; Lee, p. 359-360, pl. 33, figs. 1, 2^29^.

v.p. 1990 *Loxoconcha laeta* Ishizaki; Lee, p. 359-360, pl. 33, figs. 3, 4^29^.

non. 1990 *Loxoconcha kattoi* Ishizaki; Ruan, p. 134, pl. 2, fig. 6^72^.

non. 1990 *Loxoconcha hattorii* Ishizaki; Zhao and Wang, pl. 1, fig. 24^148^.

v. 1991 *Loxoconcha viva* Ishizaki; Ikeya and Itoh, p. 139, fig. 18B^149^.

non. 1993 *Loxoconcha uranouchiensis* Ishizaki; Kamiya and Nakagawa, p. 133, pl. 6, fig. 8^14^.

non. 1994 *Loxoconcha modesta* Ishizaki; Zheng et al., pl. 53, figs. 4, 5^32^.

v. 1998 *Loxoconcha tosamodesta* Ishizaki; Cao, pl. 3, figs. 1-4^34^.

v. 1998 *Loxoconcha tosamodesta* Ishizaki; Irizuki et al., p. 8, fig. 3.7^150^.

v. 1998 *Loxoconcha viva* Ishizaki; Irizuki et al., p. 8, fig. 3.9^150^.

v. 2001 *Loxoconcha epeterseni* Ishizaki; Yasuhara and Irizuki, p. 83, pl. 6, figs. 5-9^151^.

non 2001 *Loxoconcha epeterseni* Ishizaki; Kamiya et al., 101, fig. 17.6^36^.

v. 2002 *Loxoconcha epeterseni* Ishizaki; Irizuki et al., p. 39, pl. 1, figs. 13, 14^152^.

v. 2004 *Loxoconcha epeterseni* Ishizaki; Irizuki, p. 77, pl. 3, fig. 1^37^.

2004 *Loxoconcha tosamodesta* Ishizaki; Yamauchi, p. 73^38^.

v. 2005 *Loxoconcha epeterseni* Ishizaki; Ishii et al., p. 83-85, 88, 92-93, fig. 1M, 3, 7, 8, 9, tables 1, 2, 4^153^.

non. 2006 *Loxoconcha modesta* Ishizaki; Schornikov, p. 44^16^.

non. 2006 *Loxoconcha modesta* Ishizaki; Schornikov and Zanina, p.217, table 2^15^.

v. 2007 *Loxoconcha viva* Ishizaki; Sasaki et al., p. 524, fig. 5.23^154^.

non. 2008 *Loxoconcha (Palmoconcha) modesta* Ishizaki; Hu and Tao, p. 460, pl. 122, figs. 5, 6^17^.

v. 2008 *Loxoconcha (Argoconcha) pendulosa* Hu and Tao, p. 472, pl. 31, figs. 5, 9, 15, pl. 49, figs, 10, 13, 17, 22^17^.

non. 2010 *Loxoconcha epeterseni* Ishizaki; Ozawa and Domitsu, p. 6, fig. 5.1^155^.

v. 2012 *Loxoconcha epeterseni* Ishizaki; Tanaka et al., p. 12^40^.

non. 2012 *Loxoconcha modesta* Ishizaki; Tanaka et al., p. 12, pl. 2, fig. 9^40^.

non. 2014 *Loxoconcha tosamodesta* Ishizaki; Schornikov and Zanina, p.35, pl. 6, figs. 5, 6^156^.

v. 2018 *Loxoconcha viva* Ishizaki; Irizuki et al., p. 643, fig. 5.6^157^.

v. 2019 *Loxoconcha epeterseni* Ishizaki; Hong, et al., p. 596, fig. 5^158^.

**Type:** Left valve (Holotype), IGPS 90266, Carapace (Paratype), IGPS 90268.

**Remarks:** Ishizaki^1^ illustrated the internal line drawing of a holotype, while the present re-examination reveals the external view of the specimen. Hong et al. ^158^ proposed that *L. epeterseni* and *L. tosamodesta* are synonyms. Based on SEM observations, we concur with Hong et al.^158^ conclusion. We therefore designate *Loxoconcha epeterseni* Ishizaki, 1981^143^, as the valid specific name and *Loxoconcha tosamodesta* Ishizaki, 1981^143^, as a synonym, in accordance with ICZN Article 24.2.1^159^. The specimen illustrated by Kamiya et al.^36^ is not *L. epeterseni*, as it features a broadly arched anterior margin, a protruded ventral carina extending toward the postero-ventral region, and a slightly concave dorsal margin. Ishii et al.^153^ documented the ontogenetic changes in the number of pore canals for this species and noted its fossil record from the Quaternary onward. Cheong et al.^145^ identified this species from the Ulleung Basin, Korea, as *L. tosamodesta* based on carapace outline, fine pit distributed in the postero-dorsal area, and a straight dorsal margin. Ruan and Hao^28^ identified *Loxoconcha kattoi* from a late Pleistocene to Holocene drill core collected in the Okinawa Trough, Japan. However, we have reclassified it as *L*. *epeterseni* due to its short postero-dorsal ridge, recessed dorsal ornamentation, and three concentric ridges along the ventral to dorsal margin. The specimen of *Loxoconcha kattoi* illustrated by Ruan^72^ from the coast of Gaode and Weizhou Islands of Guangxi, China, is identified as *L. epeterseni* based on its surface ornamentation. Hu and Tao^17^ reported this species from Taiwan; however, their specimen is not *L. epeterseni* due to its inflated postero-ventral area, prominent postero-ventral carina, and smooth central area. Ozawa and Domitsu^155^ identified *L. epeterseni* from the Early Pleistocene Hamada Formation in Northern Honshu, Japan, but it differs from the type specimen by having an antero-ventral arched anterior margin, a posterior projection in the postero-ventral area, and a straight shot carinal ridge in the mid-posterior area. Schornikov and Zanina^156^ reported this species from Peter the Great Bay, but their specimen differs from the type specimen in having a dorsally directed fossa, a small flat area along the posterior margin, and two carinal ridges running parallel to the ventral and posterior margins.

**Occurrence:**

*Recent*: Hong Kong; East China Sea; Ulleung Basin, Korea; Shikoku, Southwest Japan; Central Honshu; Sendai Bay of Northern Japan.

*Fossil*: Pleistocene, Cheju Island, Korea; Early Pleistocene, Northern Ryukyu, Japan; Middle Pleistocene, Central Honshu, Japan; Late Pleistocene, Taiwan; Late Pleistocene, Central Honshu, Japan; Late Pleistocene to Holocene, Okinawa Trough, Japan; Holocene, borehole cores from Hong Kong, China; Kyushu, the Seto Inland Sea, Southwest to Northern Honshu, Japan.

**6.** *Loxoconcha hattorii* Ishizaki, 1971^3^

v*. 1971 *Loxoconcha hattorii* Ishizaki, p. 86, pl. 5, figs. 5, 9, 10, pl. 7, fig. 7^3^.

v. 1977 *Loxoconcha (Loxoconcha) hattorii* Ishizaki; Hanai et al., p. 60^8^.

1982 *Loxoconcha hattorii* Ishizaki; Cai, p. 7, pl. 2, figs. 27, 28^21^.

v. 1985 *Loxoconcha hattorii* Ishizaki; Ikeya et al., pl. 7, figs. 13-18^12^.

v. 1985 *Loxoconcha hattorii* Ishizaki; Ishizaki and Matoba, pl. 4, figs. 14, 15^13^.

v. 1985 *Loxoconcha hattorii* Ishizaki; Zhao, pl. 2, fig. 18^160^.

v.p. 1987 *Loxoconcha hattorii* Ishizaki; Wang and Zhang, p. 290, pl. 1, fig. 18^146^.

non. 1987 *Loxoconcha hattorii* Ishizaki; Wang and Zhang, p. 290, pl. 1, fig. 19^146^.

1987 *Loxoconcha hattorii* Ishizaki; Zheng, p. 199, pl. 5, figs. 28, 29^25^.

v. 1988 *Loxoconcha hattorii* Ishizaki; Ruan and Hao, p. 326, pl. 57, fig. 27^28^.

v. 1989 *Loxoconcha hattorii* Ishizaki; Ruan, pl. 1, fig. 21^161^.

v. 1990 *Loxoconcha hattorii* Ishizaki; Lee, p. 357, pl. 34, fig. 1^29^.

non. 1990 *Loxoconcha kattoi* Ishizaki; Ruan, p. 134, pl. 2, fig. 6^72^.

v. 1996 *Loxoconcha hattorii* Ishizaki; Ozawa, p. 112, pl. 6, fig. 4^33^.

v. 2001 *Loxoconcha hattorii* Ishizaki; Kamiya et al., 97, fig. 15.15^36^.

2004 *Loxoconcha hattorii* Ishizaki; Yamauchi, p. 73^38^.

v. 2005 *Loxoconcha hattorii* Ishizaki; Irizuki et al., p. 42, fig. 4.13^162^.

v. 2005 *Loxoconcha hattorii* Ishizaki; Ishii et al., p. 83-85, 88, 92-93, fig. 1J, 3, 7, 8, 9, tables 1, 2, 4^153^.

v. 2008 *Loxoconcha (Argoconcha) hattorii* Ishizaki; Hu and Tao, p. 466, pl. 202, figs. 4, 10, 12, 19, pl. 215, fig. 5, pl. 225, figs. 17, 18^17^.

v. 2009 *Loxoconcha hattorii* Ishizaki; Irizuki et al., p. 5, fig. 3.18^163^.

v. 2014 *Loxoconcha hattorii* Ishizaki; Masuma and Yamada, p. 5, fig. 6.9^41^.

v. 2014 *Loxoconcha hattorii* Ishizaki; Goto et al., p. 78, fig. 6.9^164^.

v. 2016 *Loxoconcha hattorii* Ishizaki; Yamaguchi et al., p. 298, fig. 1^165^.

v. 2018 *Loxoconcha hattorii* Ishizaki; Yamada et al., p. 337, fig. 5.4^166^.

non. 2019 *Loxoconcha bizenensis* Okubo; Yoo and Karanovic, p. 117, 118, fig. 1^167^.

v. 2019 *Loxoconcha hattorii* Ishizaki; Irizuki et al., p. 267, fig. 5.9^168^.

**Type:** Female left valve (Holotype), IGPS 91556.

**Remarks:** Ishizaki presented an SEM image of the holotype’s inner view and an optical photograph of its external view. Ishizaki’s (1971)^3^ paratype (IGPS 91557) was a male specimen. Ishii et al.^153^ documented the ontogenetic changes in the number of pore canals in this species and noted its fossil record from the Late Pliocene onward. You and Karanovic^167^ reported *Loxoconcha bizenensis* off the coast of Korea, including photographs of the shell, antenna, antennae, and male copulatory apparatus. However, we identified the specimen as *L. hattorii* based on the reticulation structure and shell outline.

**Occurrence:**

*Recent*: Shelf locations include south to middle China; the coast near Shanghai, Hubei, Guangxi, in China; the Matsu Islands, Penghu Islands, the coast of Taiwan; the Seto Inland Sea in Japan; and the Sea of Japan side of Southwest, Central, and Northern Honshu, Japan.

*Fossil*: The Late Pliocene of Central Honshu, Japan; the Middle Pleistocene of Southwest Honshu, Japan; the Late Pleistocene of Central Honshu, Japan; the Pleistocene of Cheju Island, Korea; Holocene borehole core from Hong Kong, China; and from the Sea of Japan side of Southwest Honshu and the Pacific side of Northeast Honshu, Japan.

**7.** *Loxoconcha japonica* Ishizaki, 1968^1^

v. 1913 *Loxoconcha impressa* (Baird); Kajiyama, p. 9, pl. 1, figs. 50, 51^169^.

v. 1959 *Loxoconcha rhomboidea* (Fischer); Hanai, p. 431, 432^170^.

v. 1961 *Loxoconcha* sp. Hanai, p. 371, text-fig. 12, fig. 4a, b^144^.

v*. 1968 *Loxoconcha japonica* Ishizaki, p. 28-29, pl. 2, fig. 1, pl. 6, figs. 10-12^1^.

v. 1971 *Loxoconcha* sp. Igo and Ikeya, p. 204, fig. 13^171^.

v. 1977 *Loxoconcha (Loxoconcha) japonica* Ishizaki; Hanai et al., p. 61^8^.

v. 1979 *Loxoconcha japonica* Ishizaki; Hu, p. 69-70, pl. 2, figs. 32-37, text-fig. 8^172^.

v. 1980 *Loxoconcha japonica* Ishizaki; Okubo, p. 416-418, figs.12, 13, 18a-d^173^.

v. 1981 *Loxoconcha japonica* Ishizaki; Hu, p. 77, pl. 3, figs. 1-4, 8, text-fig. 14^174^.

1982 *Loxoconcha japonica* Ishizaki; Cai, p. 7, pl. 2, fig. 21^21^.

v.p. 1983 *Loxoconcha japonica* Ishizaki; Hu, p. 156-157, pl. 2, fig. 3^175^.

non. 1983 *Loxoconcha japonica* Ishizaki; Hu, pl. 2, figs. 4, 6, 7^175^.

v. 1984 *Loxoconcha japonica* Ishizaki; Hu, p. 116, pl. 4, figs. 24-26^176^.

v.p. 1986 *Loxoconcha japonica* Ishizaki; Hu and Tao, p. 62, pl. 3, figs. 22, 26^177^.

v. 1986 *Loxoconcha japonica* Ishizaki; Hu, p. 162, pl. 4, figs. 22, 28, 30, 31^23^.

v. 1987 *Loxoconcha sinensis* (Brady); Wang and Zhang, p. 290, pl. 1, fig. 20^146^.

v. 1987 *Loxoconcha uranouchiensis* Ishizaki; Wang and Zhang, p. 290, pl. 1, fig. 23^146^.

v. 1988 *Loxoconcha japonica* Ishizaki; Ikeya and Shiozaki, p. 139, pl. 4, figs. 1a, 1b^27^.

v. 1988 *Loxoconcha japonica* Ishizaki; Kamiya, p. 319, 321-324, 326, 327, 329-331, pl. 1, figs.1-6, text-figs. 4-6, 8, 10, 11, 13-15, table 1^178^.

v. 1988 *Loxoconcha japonica* Ishizaki; Kamiya, p. 339-342, 345, pl. 1, figs. 9-16, text-figs. 1, 2, 4, table 1^179^.

v. 1988 *Loxoconcha japonica* Ishizaki; Kamiya, p. 307, 308, 310, 311, 313-316, pl. 1, figs. 1-7, text-figs. 4, 5.1, 5.2, 7, 8, 10-13^180^.

v. 1988 *Loxoconcha japonica* Ishizaki; Ruan and Hao, p. 323, pl. 57, figs. 11-13^28^.

v. 1989 *Loxoconcha japonica* Ishizaki; Kamiya, p. 39-41, 43-45, pl. 1, figs. 1, 2, 5-8, 13, 14, 17, pl. 2, figs. 1-3, 10, figures. 1-8^181^.

v. 1989 *Loxoconcha japonica* Ishizaki; Kamiya, p. 78, 79, 81-83, figs. 3, 4.1, 4.2, 6, 7, 9, 11, 12^182^.

v. 1990 *Loxoconcha japonica* Ishizaki; Lee, p. 358, pl. 34, figs. 3, 4^29^.

v. 1990 *Loxoconcha japonica* Ishizaki; Ruan, p. 134, pl. 2, fig. 3^72^.

non. 1991 *Loxoconcha japonica* Ishizaki; Cai, p. 122, pl. 7, fig. 12^30^.

v. 1992 *Loxoconcha japonica* Ishizaki; Ikeya and Suzuki, p. 129, pl. 5, fig. 9^183^.

v. 1992 *Loxoconcha japonica* Ishizaki; Kamiya and Hazel, p. 160-163, 166, figs, 1-4, 6, pl. 1, table 1^184^.

v. 1992 *Loxoconcha japonica* Ishizaki; Nohara and Oshiro, p.336, fig. 2.6^185^.

v. 1993 *Loxoconcha japonica* Ishizaki; Kamiya and Nakagawa, p. 131, pl. 5, figs, 11, 12^14^.

non. 1994 *Loxoconcha japonica* Ishizaki; Zheng et al., pl. 53, fig, 13^32^.

v. 1996 *Loxoconcha japonica* Ishizaki; Ozawa, p. 112, pl. 6, fig. 5^33^.

v. 1998 *Loxoconcha japonica* Ishizaki; Cao, pl. 3, figs. 15, 17, 18, 19^34^.

v. 1998 *Loxoconcha japonica* Ishizaki; Irizuki et al., p. 8, fig. 3.5^150^.

v. 1998 *Loxoconcha japonica* Ishizaki; Yamane, p. 47, pl. 6, fig. 2^35^.

v. 2001 *Loxoconcha japonica* Ishizaki; Kamiya et al., 101, fig. 17.9^36^.

v. 2001 *Loxoconcha* cf. *japonica* Ishizaki; Tabuki, p. 33, fig. 10.1^186^.

v. 2001 *Loxoconcha japonica* Ishizaki; Yasuhara and Irizuki: p. 83, pl. 6, fig. 12^151^.

v. 2002 *Loxoconcha japonica* Ishizaki; Tanaka, p. 26, figs. 1c, 1d^187^.

v. 2002 *Loxoconcha japonica* Ishizaki; Tanaka and Ikeya, p.266, 269-279, figs.1, 3.1-3.3, 3.6, 5A, 5a, 7A, 8, 9.1, 9.2, 10-14, table 2^188^.

v. 2003 *Loxoconcha japonica* Ishizaki; Smith and Kamiya, p. 31-51, figs. 1-15, table 1^189^.

v. 2004 *Loxoconcha japonica* Ishizaki; Horne et al., p. 277, 278, 285, 286, figs. 20, 21, 25, 26^190^.

2004 *Loxoconcha japonica* Ishizaki; Yamauchi, p. 73^38^.

v. 2005 *Loxoconcha japonica* Ishizaki; Ishii et al., p. 83-85, 87-93, fig. 1A, 2-9, tables 1, 2, 4^153^.

v. 2005 *Loxoconcha japonica* Ishizaki; Smith and Kamiya, p. 224, 226, 228, figs. 6-8, tables 2, 3^191^.

v. 2005 *Loxoconcha japonica* Ishizaki; Smith and Tsukagoshi, p.168, fig. 9^192^.

v. 2006 *Loxoconcha japonica* Ishizaki; Irizuki et al., p. 26, fig. 8.2^39^.

v. 2007 *Loxoconcha japonica* Ishizaki; Hou and Gou, pl. 155, figs. 13, 14^106^.

v. 2006 *Loxoconcha japonica* Ishizaki; Kamiya et al., p. 110, fig. 1^193^.

v. 2007 *Loxoconcha japonica* Ishizaki; Sasaki et al., p. 524, fig, 5.18^154^.

v. 2007 *Loxoconcha japonica* Ishizaki; Tanaka, p. 125, fig, 2A^194^.

v. 2008 *Loxoconcha japonica* Ishizaki; Hu and Tao, p. 455-456, pl. 73, fig. 5, pl. 122, fig. 2, pl. 131, pl. 151, figs. 13, 14, pl. 180, fig. 13, pl. 202, fig. 13^17^.

non. 2008 *Loxoconcha (Loxoconcha) japonica* Ishizaki; Hu and Tao, pl. 73, fig. 2, 12, 24, pl. 131, fig. 7, pl. 150, fig. 7, pl. 151, figs. 1, 2, 12, 22, pl. 155, fig. 5, pl. 180, figs. 5, 15, 19, pl. 202, fig. 3^17^.

v. 2009 *Loxoconcha japonica* Ishizaki; Savatenalinton and Martens, p. 275, fig, 9D^195^.

non. 2009 *Loxoconcha japonica* Ishizaki; Tanaka and Nomura, p. 62, fig, 4.3^196^.

v. 2010 *Loxoconcha japonica* Ishizaki; Morishita et al., p. 539, fig, 11^197^.

v. 2011 *Loxoconcha japonica* Ishizaki; Kawano et al., p. 5, fig, 3.9^198^.

2012 *Loxoconcha japonica* Ishizaki; Tanaka et al., p. 11-12^40^.

v. 2012 *Loxoconcha japonica* Ishizaki; Tanaka et al., p. 117, pl. 1, fig. 11^199^.

v. 2013 *Loxoconcha japonica* Ishizaki; Kawano et al., p. 96, fig. 5.7^200^.

v. 2014 *Loxoconcha hattorii* Ishizaki; Goto et al., p. 78, fig. 6.9^164^.

v. 2014 *Loxoconcha japonica* Ishizaki; Masuma and Yamada, p. 5, fig. 3.24^41^.

v. 2017 *Loxoconcha japonica* Ishizaki; Tsukagoshi, p. 20, figs. 8.1, 8.2, 9-11^201^.

v. 2019 *Loxoconcha japonica* Ishizaki; Yoo and Karanovic, p. 118-119, fig. 2^167^.

v. 2019 *Loxoconcha* sp. 18 Le and Tsukagoshi, p. 19, fig. 4.2^202^.

v. 2019 *Loxoconcha* sp. 20 Le and Tsukagoshi, p. 19, fig. 4.4^202^.

v. 2019 *Loxoconcha japonica* Ishizaki; Niiyama et al., p. 105, fig. 4.5^133^.

v. 2019 *Loxoconcha japonica* Ishizaki; Tanaka et al., p. 10, fig. 2.19-20, p. 11, fig. 3.1, 3.2^203^.

**Type:** Male left valve (Holotype), IGPS 90260.

**Remarks:** Ishizaki^1^ provided an internal line drawing of the holotype (pl. 2, fig. 1) and an optical photograph (pl. 6, fig. 11). The present reexamination reveals detailed view of the holotype’s exterior. Okubo^173^ illustrated the male soft parts of this species. Kamiya^178^ noted that this species inhabits Zostera leaves, and its population density increases from March to June. He also observed that the female body length increases during low temperatures and decreases during high temperatures; this size pattern is preserved in subfossil assemblages. Additionally, the juvenile ratio is low during summer. Kamiya^179^ reported a higher female-to-male ratio, indicating high male mortality. Kamiya^180^ found that copulation lasts less than three seconds. Kamiya^181^ illustrated the shape and distribution of twisted and smooth bristles on this species’ shell. Kamiya and Hazel^184^ observed that smooth bristles consistently increase throughout each molting stage, while twisted bristle increase little after the A-4 stages. Tanaka and Ikeya^188^ reviewed the genus *Loxoconcha* and identified four species in the *Loxoconcha japonica* species group, also examining the fossil and recent paleo-biogeographical distribution of the group. According to Tanaka and Ikeya^188^, *L. japonica* has its oldest fossil record from the Early Pliocene Maja Formation (revised to Late Miocene by Tanaka and Nomura^196^) on Kume Island, Okinawa, with later occurrences from Taiwan to Northern Honshu, Japan. Smith and Kamiya^189^ documented ontogenetic changes from A-8 instar to adults and illustrated the development of appendages with SEM images. Ishii et al.^153^ showed the ontogenetic change in the number of pore canals and noted its fossil record from the Late Pliocene onward. Smith and Kamiya^191^ demonstrated that the ontogenetic development of appendages and the copulatory organ after the A-4 stage is similar to the adult stage. Morishita et al. ^197^ mapped the distribution of Mg, Ca, and Sr in this species valve. Wang and Zhang^146^ identified juveniles of *L. japonica* as *Loxoconcha sinensis* and *L. uranouchiensis* from Holocene borehole cores in Hong Kong, China. Hu^175^ included *Loxoconcha shanhaiensis* Hu, 1981^174^ in his illustrations. Cai^30^ reported this species from the Nansha Islands but noted differences from type specimens, such as the lack of a flat area along the posterior margin, finer reticulation in the posterior area, and a shorter dorsal margin. Zheng et al.^32^ identified a specimen as *L*. *shanhaiensis* based on its lateral profile and reticulation pattern. Specimens identified as *L. japonica* by Hu and Tao^17^ were classified as *Loxoconcha shanhaiensis* Hu, 1981^174^ or *L*. *tumulosa* (Hu, 1979) ^172^ due to the long and low shell characteristic of *L*. *shanhaiensis* and the postero-dorsal ridge characteristic of *L*. *tumulosa*. *Loxoconcha japonica* figured by Tanaka and Nomura^196^ from the Middle Pliocene Aka Formation differs from the type species in its fine reticulation, irregular reticulation in the mid-dorsal area, and widely arched anterior margin. *Loxoconcha* sp. 18 and L. sp. 20 reported by Le and Tsukagoshi^202^ from Northern Vietnam are male and female specimens of *Loxoconcha japonica*.

**Occurrence:**

*Recent*: Co To Islands, Northern Vietnam; Guangxi, China; Penghu Islands, Taiwan; Coast of South Korea; Kyushu, Shikoku, Seto Inland Sea, Sea of Japan side of Southwest Honshu and Central Honshu, Japan.

*Fossil*: Pliocene southern Taiwan; Late Pliocene Central Honshu on the Sea of Japan side; Plio-Pleistocene Taiwan; Plio-Pleistocene Central Honshu, Japan; Late Pliocene or Early Pleistocene Southern Taiwan; Pleistocene Taiwan; Early Pleistocene Okinawa, Japan; Pleistocene Cheju Island, Korea; Middle Pleistocene Kyushu, Japan; Late Pleistocene Okinawa, Japan; Pleistocene to Holocene Taiwan; Okinawa Trough, Japan; Central and Northern Honshu, Japan; Holocene borehole core from Central Japan; Quaternary borehole cores from Hong Kong, China; Southwest and Central Honshu, Japan.

**8.** *Loxoconcha kattoi* Ishizaki, 1968^1^

v*. 1968 *Loxoconcha kattoi* Ishizaki, p. 29-30, pl. 1, fig. 13, pl. 6, figs. 14, 15^1^.

v. 1977 *Loxoconcha (Loxoconcha) kattoi* Ishizaki; Hanai et al., p. 61^8^.

v. 1985 *Loxoconcha kattoi* Ishizaki; Ikeya et al., pl. 7, figs. 11, 12^12^.

v. 1988 *Loxoconcha zamia* Ishizaki; Ikeya and Shiozaki, p. 139, pl. 4, fig. 2^27^.

non. 1988 *Loxoconcha kattoi* Ishizaki; Ruan and Hao, p. 323-333, pl. 57, figs. 14-16^28^.

v. 1990 *Loxoconcha kattoi* Ishizaki; Lee, p. 358-359, pl. 36, fig. 11^29^.

v. 1992 *Loxoconcha kattoi* Ishizaki; Ikeya and Suzuki, p. 129, pl. 5, fig. 10^183^.

v. 1993 *Loxoconcha kattoi* Ishizaki; Kamiya and Nakagawa, p. 133, pl. 6, fig. 1^14^.

non. 1994 *Loxoconcha kattoi* Ishizaki; Zheng et al., pl. 53, fig. 14^32^.

v. 1995 *Loxoconcha kattoi* Ishizaki; Ozawa et al., p. 31, pl. 1, fig. 13^204^.

v. 1998 *Loxoconcha kattoi* Ishizaki; Yamane, p. 47, pl. 6, fig. 3^35^.

v. 2001 *Loxoconcha kattoi* Ishizaki; Yasuhara and Irizuki: p. 83, pl. 6, fig. 13^151^.

v. 2001 *Loxoconcha kattoi* Ishizaki; Kamiya et al., 101, fig. 17.13^36^.

v. 2002 *Loxoconcha kattoi* Ishizaki; Irizuki et al., p. 39, pl. 1, fig. 15^152^.

2004 *Loxoconcha kattoi* Ishizaki; Yamauchi, p. 73^38^.

v. 2006 *Loxoconcha kattoi* Ishizaki; Ishii et al., p. 83-85, 88, 92, 93, fig. 8.3^153^.

v. 2006 *Loxoconcha kattoi* Ishizaki; Irizuki et al., p. 25, fig. 1C, 378,

tables 1, 2, 4^39^.

v. 2008 *Loxoconcha (Argoconcha) kattoi* Ishizaki; Hu and Tao, p. 468-469, pl. 31, fig. 1, pl. 150, figs. 8, 11, 13-16, 18, 20-22^17^.

v. 2008 *Loxoconcha kattoi* Ishizaki; Iwatani and Irizuki, p. 68, fig. 6.19^205^.

v. 2009 *Loxoconcha kattoi* Ishizaki; Tanaka and Nomura, p. 62, fig. 4.4^196^.

v. 2012 *Loxoconcha kattoi* Ishizaki; Tanaka et al., p. 12, pl. 2, fig. 7^40^.

v. 2012 *Loxoconcha kattoi* Ishizaki; Tanaka et al., p. 117, pl. 1, fig. 13^199^.

**Type:** Left valve (Holotype), IGPS 90264.

**Remarks:** Ishii et al.^153^ documented the ontogenetic change in the number of pore canals and noted its fossil record from the Late Pliocene onward. Zheng et al.^32^ reported this species from the South China Sea; however, it differs from the type specimen due to its acutely arched anterior margin, absence of carinal ridges, and a postero-dorsally protruded caudal process.

**Occurrence:**

*Recent*: Kyushu, Shikoku, and the Sea of Japan side of Southwest Honshu, and Central Honshu, Japan.

*Fossil*: Late Miocene, Okinawa, Japan; Middle Pliocene Okinawa, Japan; Early Pliocene Taiwan; Late Pliocene Kyushu, Japan; Plio-Pleistocene Taiwan; Pleistocene Cheju Island, Korea; Middle Pleistocene Central Honshu, Japan; Holocene Southwestern and Central Honshu, Japan.

9. *Loxoconcha kitanipponica* Ishizaki, 1971^3^

v*. 1971 *Loxoconcha kitanipponica* Ishizaki, p. 87, pl. 5, fig. 4, pl. 6, figs. 11, 12, pl. 7, fig. 10^3^.

v. 1977 *Loxoconcha (Loxoconcha) kitanipponica* Ishizaki; Hanai et al., p. 61^8^.

non. 2005 *Loxoconcha kattoi* Ishizaki; Ishii et al., p. 83-85, 88, 92, 93, fig. 1C, 3, 7-9,

tables 1, 2, 4^153^.

v. 2006 *Loxoconcha kitanipponica* Ishizaki; Irizuki et al., p. 25, fig. 8.4^39^.

non. 2011 *Loxoconcha kitanipponica* Ishizaki; Kawano et al., p. 5, fig. 3.10^198^.

v. 2019 *Loxoconcha kitanipponica* Ishizaki; Tanaka et al., p. 11, fig. 3.3-5^203^.

**Type:** Male left valve (Holotype), IGPS 91559.

**Remarks:** Ishizaki presented an SEM image of the holotype’s inner view and an optical photograph of its external view. He also showed that the right valve of the paratype (IGPS 91560) is male. Ishii et al.^153^ documented the ontogenetic change in the number of pore canals and noted its fossil record from the Late Quaternary onward. Kawano et al.^198^ discovered a juvenile specimen from the Middle Pleistocene Ogushi Formation, Amakusa, Kyushu, Japan. However, we concluded that their specimen is juvenile *L. harimensis* based on the lateral outline and the direction of the postero-ventral spine.

**Occurrence:**

*Recent*: Kyushu and Northern Honshu, Japan; Kyushu.

*Fossil*: Middle Pleistocene Kyushu, Japan.

**10.** *Loxoconcha mutsuense* (Ishizaki, 1971)^3^

v*. 1971 *Loxocorniculum mutsuensis* Ishizaki, p. 89-90, pl. 5, fig. 11, pl. 6, figs. 3, 6, 7, pl. 7, fig. 5^3^.

v. 1977 *Loxocorniculum mutsuense* Ishizaki; Hanai et al., p. 65^8^.

v. 1980 *Loxocorniculum mutsuense* Ishizaki; Okubo, p. 424-425, figs. 17, 20^173^.

v. 1985 *Loxocorniculum mutsuense* Ishizaki; Ikeya et al., pl. 8, figs. 4^12^.

v. 1988 *Loxocorniculum mutsuense* Ishizaki; Kamiya, p. 307, text-fig. 4^180^.

v.p. 1988 *Loxocorniculum mutsuensis* Ishizaki; Ruan and Hao, p. 330, pl. 59, figs. 2, 4, 5^28^.

v. 1989 *Loxocorniculum mutsuense* Ishizaki; Kamiya, p. 78, 79, figs. 3, 4.5^182^.

v. 1990 *Loxocorniculum mutsuense* Ishizaki; Lee, p.362-363, pl. 34, figs. 5, 6^29^.

v. 1996 *Loxocorniculum mutsuense* Ishizaki; Ozawa, p.113, pl. 7, fig. 1^33^.

v. 1998 *Loxocorniculum mutsuense* Ishizaki; Yamane, p. 49, pl. 7, fig. 3^35^.

v. 2003 *Loxocorniculum mutsuense* Ishizaki; Yamaguchi, p. 135, fig. 1I^206^.

v. 2004 *Loxocorniculum mutsuense* Ishizaki; Irizuki, p. 77, pl. 3, fig. 6^37^.

v. 2005 *Loxocorniculum mutsuense* Ishizaki; Ishii et al., p. 83, 84, 86, 87, 88, 92, 93, figs. 1Q, 2, 3, 4, 7-9, tables 1, 2, 4^153^.

v. 2006 *Loxocorniculum mutsuense* Ishizaki; Irizuki et al., p.25, fig. 8.9^39^.

2006 *Loxocorniculum mutsuense* Ishizaki; Schornikov and Zanina, p. 217, table 2^15^.

v. 2007 *Loxocorniculum mutsuense* Ishizaki; Tanaka, p.125, fig. 2C^194^.

2006 *Loxocorniculum mutsuense* Ishizaki; Schornikov, p.44^16^.

v. 2008 *Loxocorniculum mutsuense* Ishizaki; Hu and Tao, p. 447, pl. 107, figs. 1, 3, pl. 123, fig. 1, pl. 181, figs. 3, 4, 7, 10, text-fig. 264^17^.

v. 2008 *Loxocorniculum mutsuense* Ishizaki; Ozawa and Ishii, p. 247, fig. 9^207^.

v. 2009 *Loxocorniculum mutsuense* Ishizaki; Tanaka and Nomura, p. 62, fig. 4.8^196^.

v. 2010 *Loxoconcha mutsuense* (Ishizaki); Ozawa, p. 84, fig. 12^208^.

v.p. 2010 *Loxocorniculum mutsuense* Ishizaki; Ozawa, p. 33, pl. 3, fig. 11^208^.

v. 2012 *Loxocorniculum mutsuense* Ishizaki; Tanaka et al., p. 13, pl. 2, fig. 10^40^.

v. 2013 *Loxoconcha mutsuense* Ishizaki; Ozawa, p. 65, 68, figs. 14, 18^209^.

v. 2017 *Loxocorniculum mutsuense* Ishizaki; Tsukagoshi, p. 20, fig. 5^201^.

v. 2019 *Loxocorniculum mutsuense* Ishizaki; Yoo and Karanovic, p. 120, 123, fig. 6^167^.

**Type:** Male left valve (Holotype), IGPS 91571.

**Remarks:** Ishizaki presented a SEM of the inner view of the holotype (pl. 7, fig. 5) and an optical photograph of the external view (pl. 6, fig. 7), as well as an optical photograph of an external view of the male. In 1971, Ishizaki provided the optical micrograph of the male right valve (pl. 6, fig. 6, paratype, IGPS 91572) and the SEM image of the female right valve (pl. 6, fig. 3) of the paratype (IGPS 91573). Benson and Coleman^210^ established the genus *Loxocorniculum* with *Loxoconcha fischeri* (Brady, 1869)^211^ as the type species, characterized by moderate and coarse reticulation with a hornlike protuberance on the postero-dorsal area. This reticulation is also frequently found in the genus *Loxoconcha*. Additionally, in this study, the hornlike protuberance on the postero-dorsal area was observed in many species of the genus *Loxoconcha*, such as *L. epeterseni*, *L. hattorii*, *L. kattoi*, *L. kitanipponica*, *L. tosaensis*, and *L. zamia*. Given the vague diagnosis of the genus *Loxocorniculum*, which cannot be distinguished from the genus *Loxoconcha*, we opted not to use the genus *Loxocorniculum* in this study. Okubo^173^ described the soft parts of this species, and Kamiya^180^ illustrated that this species lives on eelgrass. Yamaguchi^203^ analyzed the 18S rDNA of this species from Tsukumo Bay, Southwest Honshu, Japan. Ishii et al.^153^ documented the ontogenetic change in the number of pore canals and noted its fossil record from the Late Pliocene onward. The specimens illustrated by Hu and Tao^17^ from the Late Pleistocene to Holocene in southern Taiwan possess two nodes in the mid-posterior area, a feature not found in the type specimen described by Ishizaki^3^ from Northern Honshu, Japan. One of the authors (G.T.) collected the Recent species from the coast of Taiwan in 1999, dissected the soft parts, and compared them with the male copulatory organ from Honshu, Japan, as illustrated by Okubo^173^. These male copulatory organs were morphologically identical, suggesting that the development of the two nodes in the mid-posterior area represents intraspecific variation.

**Occurrence:**

*Recent*: The coast of South Korea; Kyushu, Seto Inland Sea, and Central and Northern Honshu, Japan.

*Fossil*: Late Miocene Okinawa, Japan; Middle Pliocene Okinawa, Japan; Plio-Pleistocene Sea of Japan, Central Honshu, Japan; Pleistocene Cheju Island, Korea; Early Pleistocene Northern Ryukyu and Central Honshu, Japan; Middle Pleistocene Sado Island, Central Japan; Late Pleistocene Southern Taiwan; Late Pleistocene Okinawa Trough, Japan; Quaternary Central Japan; Holocene Southern Taiwan.

**11.** *Loxoconcha optima* Ishizaki, 1968^1^ **Fig. 2n–p**

v. 1961 *Loxoconcha* sp. Hanai, p. 371, text-fig. 12, fig. 5a, b^144^.

v*. 1968 *Loxoconcha optima* Ishizaki, p. 30-31, pl. 2, fig. 2, pl. 6, figs. 8, 9^1^.

v. 1977 *Loxoconcha (Loxoconcha)* *optima* Ishizaki; Hanai et al., p. 62^8^.

v. 1985 *Loxoconcha (Loxoconcha)* *optima* Ishizaki; Ikeya et al., pl. 7, figs. 1-5^12^.

non. 1985 *Loxoconcha* *optima* Ishizaki; Ishizaki and Matoba, pl. 4, fig. 18^13^.

non. 1988 *Loxoconcha* *optima* Ishizaki; Ruan and Hao, p. 324, pl. 57, figs. 18, 19^28^.

non. 1988 *Loxoconcha* *tarda* Guan; Ruan and Hao, p. 325-326, pl. 57, figs. 25, 26^28^.

non. 1989 *Loxoconcha* *optima* Ishizaki; Ruan, pl. 1, fig. 23^161^.

non. 1990 *Loxoconcha optima* Ishizaki; Lee, p. 329, fig. 7H^29^.

non. 1990 *Loxoconcha optima* Ishizaki; Takayasu et al., pl. 1, fig. 12^212^.

non. 1990 *Loxoconcha optima* Ishizaki; Yajima and Lord, p. 157, figs. 4.14, 4.15^213^.

non. 1992 *Loxoconcha optima* Ishizaki; Huh and Paik, p. 283, pl. 2, fig. 17^214^.

non. 1992 *Loxoconcha optima* Ishizaki; Ikeya and Suzuki, p. 131, pl. 6, fig. 2^183^.

non. 1993 *Loxoconcha optima* Ishizaki; Ishizaki et al., p. 329, fig. 7H^215^.

non. 1993 *Loxoconcha optima* Ishizaki; Kamiya and Nakagawa, p. 133, pl. 6, figs. 2, 3^14^.

non. 1994 *Loxoconcha* *tarda* Guan; Zheng et al., pl. 53, figs. 6, 7^32^.

v. 1995 *Loxoconcha* *optima* Ishizaki; Ozawa et al., p. 31, pl. 1, fig. 17^204^.

non. 1996 *Loxoconcha optima* Ishizaki; Ozawa, p. 113, pl. 6, fig. 7^33^.

non. 2001 *Loxoconcha optima* Ishizaki; Kamiya et al., 103, fig. 18.2^36^.

v. 2002 *Loxoconcha optima* Ishizaki; Tanaka and Ikeya, p.266, fig.1^188^.

2004 *Loxoconcha optima* Ishizaki; Yamauchi, p. 73^38^.

non. 2005 *Loxoconcha optima* Ishizaki; Ishida and Takayasu, p. 76, fig. 2.8^216^.

non. 2005 *Loxoconcha optima* Ishizaki; Ishii et al., p. 83, fig. 1E^153^.

non. 2005 *Loxoconcha optima* Ishizaki; Ozawa and Kamiya, p. 257, pl. 1, fig. 1^217^.

non. 2006 *Loxoconcha optima* Ishizaki; Irizuki et al., p. 25, fig. 8.5^39^.

v. 2007 *Loxoconcha optima* Ishizaki; Tanaka, p. 125, fig. 2B^194^.

non. 2008 *Loxoconcha (Salixiconcha) optima* Ishizaki; Hu and Tao, p. 487, pl. 215, figs. 8,12, text-fig. 283^17^.

v. 2009 *Loxoconcha optima* Ishizaki; Ozawa, p. 239, fig. 4.5^218^.

v. 2011 *Loxoconcha optima* Ishizaki; Irizuki et al., p. 42, fig. 4.13^219^.

v. 2012 *Loxoconcha optima* Ishizaki; Tanaka et al., p. 12, pl. 2, fig. 5^40^.

non. 2014 *Loxoconcha optima* Ishizaki; Masuma and Yamada, p. 6, fig. 4.1^41^.

v. 2022 *Loxoconcha optima* Ishizaki; Kaneko et al., p. 65, pl. 3, fig. 6^220^.

**Type:** Male detached carapace (Holotype), IGPS 90269.

**Remarks:** Ishizaki^1^ provided an internal line drawing of the holotype (pl. 2, fig. 2) and optical photographs of the left and right valves of the same specimen (pl. 6, figs. 8, 9). The juvenile specimen depicted by Ishizaki and Matoba^13^ from the Middle Pleistocene Anden Formation, northern Japan, is not identified as *L. optima* due to its broadly arched anterior margin, short caudal process, and straight dorsal margin. Ruan and Hao^28^ reported this species from Late Pleistocene to Holocene borehole cores from the Okinawa Trough, Japan; however, it differs in having a smooth surface, a broadly arched anterior margin, and a straight dorsal margin. Ruan also reported this species from the coast of Hubei, China, but it again differs due to its smooth surface, broadly arched anterior margin, and straight dorsal margin. The specimen illustrated by Yajima and Lord^213^ from the Middle Pleistocene Yabu Formation, Central Honshu, Japan, is not *L. optima*, as it exhibits postro-ventrally inclined central reticulation. *Loxoconcha optima* illustrated by Huh and Paik^214^ from the Miocene Chunbuk Conglomerate, Pohang Basin, Korea, differs from the type species in its broadly arched anterior margin, lack of carinal ridges in the central area, and sinuate ventral margin. The specimen illustrated by Ishizaki et al.^215^ from the Early Pleistocene Omma Formation does not match the type specimen due to its widely arched anterior margin, widely arched dorsal margin, and underdeveloped central short ridges that are slightly arched toward the dorsal direction. Illustrations of *Loxoconcha optima* Lee^29^, Ikeya and Suzuki^183^, Kamiya and Nakagawa^14^, Takayasu et al.^212^, Ozawa^33^, Kamiya et al.^36^, Ishida and Takayasu^216^, Ishii et al.^153^, Ozawa and Kamiya^217^, Irizuki et al.^39^, and Masuma & Yamada^41^ depict specimens that do not correspond to *L. optima* due to its widely arched anterior margin, absence of a caudal process, and a widely arched dorsal margin. The juvenile specimens illustrated by Hu and Tao^17^ are not *L. optima* because they have a broadly arched anterior margin, a rounder lateral outline, and a longer ventral margin.

**Occurrence:**

*Recent*: Shikoku, Southwest Japan; and the Pacific side of Central Honshu, Japan.

*Fossil*: Middle Pleistocene from Central Honshu, Japan.

**12.** *Loxoconcha pulchra* Ishizaki, 1968^1^ **Fig. 2q–r**

v*. 1968 *Loxoconcha pulchra* Ishizaki, p. 31, pl. 1, fig. 16, pl. 7, figs.19, 20^1^.

v. 1977 *Loxoconcha (Loxoconcha)* *pulchra* Ishizaki; Hanai et al., p. 62^8^.

1981 *Loxoconcha pulchra* Ishizaki; Gou et al., p. 166, pl. 81, fsigs. 5, 6^221^.

v. 1984 *Loxoconcha pulchra* Ishizaki; Ishizaki, p. 43, pl. 2, fig. 12^222^.

v. 1985 *Loxoconcha pulchra* Ishizaki; Ikeya et al., pl. 7, figs. 1-5^12^.

non. 1988 *Loxoconcha pulchrsa* Ishizaki; Yajima, p. 1076, pl. 1, fig. 3^223^.

v. 1993 *Loxoconcha pulchra* Ishizaki; Kamiya and Nakagawa, p. 133, pl. 6, figs. 4, 5^14^.

non. 1994 *Loxoconcha pulchra* Ishizaki; Irizuki and Matsubara, pl. 1, fig. 18^224^.

non. 1994 *Loxoconcha pulchra* Ishizaki; Tsukagoshi et al., p. 49, 50, 57, fig. 2-28^225^.

v. 1998 *Loxoconcha pulchra* Ishizaki; Yamane, p. 47, pl. 6, fig. 5^35^.

v. 2000 *Loxoconcha pulchra* Ishizaki; Irizuki and Hosoyama, p. 12, fig. 3.16^226^.

v. 2001 *Loxoconcha pulchra* Ishizaki; Yasuhara and Irizuki: p. 83, pl. 6, fig. 14^151^.

v. 2002 *Loxoconcha pulchra* Ishizaki; Nakao and Tsukagoshi, p. 99-100, figs. 17, 18^227^.

v. 2002 *Loxoconcha pulchra* Ishizaki; Tanaka and Ikeya, p. 266, fig. 1^188^.

v. 2002 *Loxoconcha pulchra* Ishizaki; Yasuhara et al., p. 635, fig. 3.9^228^.

non. 2004 *Loxoconcha pulchra* Ishizaki; Irizuki et al., p. 141, pl. 8, figs. 11-13^229^.

v. 2004 *Loxoconcha pulchra* Ishizaki; Yasuhara et al., p. 31, fig. 13o, p. 33, fig. 15o^230^.

v. 2005 *Loxoconcha pulchra* Ishizaki; Ishii et al., p. 83-84, 86, 88, 89, 92, 93, fig. 1F, 3, 4, 7-9, tables 1, 2, 4^153^.

v. 2007 *Loxoconcha pulchra* Ishizaki; Yamada, p. 52, figs. 5L, 5M, 6D^231^.

v. 2007 *Loxoconcha pulchra* Ishizaki; Yamada, p. 205-209, figs. 3b, 3c, 5a, 5b, 6a, 6b, 10^232^.

v. 2008 *Loxoconcha pulchra* Ishizaki; Yamada, p. 42, 44, 47, 50-53, 55, figs. 1F, 4B, 8, 11-15, 17^233^.

v. 2008 *Loxoconcha pulchra* Ishizaki; Nakao and Tsukagoshi, p.275, pl. 7, figs. A-D^234^.

v. 2009 *Loxoconcha pulchra* Ishizaki; Yamada and Keyser, p. 205, 206, 207, 209, figs. 3b, 5a, 5b, 6a, 6b, 10^235^.

v. 2011 *Loxoconcha pulchra* Ishizaki; Yamada and Matzke-Karasz, p. 1343-1348, figs. 1, 2, 3A, 3B, 3D, 4-6^236^.

v. 2012 *Loxoconcha pulchra* Ishizaki; Tanaka et al., p. 9, fig. 4H^237^.

v. 2016 *Loxoconcha pulchra* Ishizaki; Matsushima et al., p. 14, fig. 6E^238^.

v. 2017 *Loxoconcha pulchra* Ishizaki; Karanovic et al., p. 42-45, figs. 5-7^239^.

v. 2017 *Loxoconcha pulchra* Ishizaki; Kamiyama et al., p. 125, fig. 3P^240^.

v. 2017 *Loxoconcha pulchra* Ishizaki; Tsukagoshi, p. 16, figs. 4A, 4B^201^.

v. 2018 *Loxoconcha pulchra* Ishizaki; Yamada et al., p. 337, fig. 5.6^166^.

v. 2019 *Loxoconcha pulchra* Ishizaki; Tanaka et al., p. 11, fig. 3.6^203^.

**Type:** Female right valve (Holotype), IGPS 90270.

**Remarks:** Ishizaki^1^ illustrated the internal line drawing of the holotype (pl. 1, fig. 16) and provided optical photograph of the specimen (pl. 7, fig. 20), along with an optical photograph of the female left valve (paratype, pl. 7, fig. 19). Nakao and Tsukagoshi^227^ described the soft parts of this species and presented SEMs of both the male and female. They also noted that this species is primarily distributed within the 21–28 PSU range in the Obitsugawa Estuary, Central Honshu, Japan. Based on the SEMs from Nakao and Tsukagoshi^227^, Ishizaki’s type specimen is identified as female. Karanovic et al.^239^ reported that the Korean specimen appears to have smooth appendages and three setae postero-medially on the third podomere of the antenna. Ishii et al.^153^ demonstrated the ontogenetic change in the number of pore canals. Yamada^232^ provided SEM and TEM images of the hinge and ligament of this species. Yamada and Keyser^235^ illustrated SEM and TEM images of the adductor muscle attachment and discussed the ontogenetical and developmental changes in the adductor muscle (scars) of this species. Yajima^223^ identified this species from the Early Middle Miocene Shukunohora Sandstone, Central Japan; however, it differs by having three carinal ridges running from the anterior to posterior margin via the ventral margin, lacking a postero-ventrally inflated valve, and having an acutely arched anterior margin. Irizuki and Matsubara^224^ figured *Loxoconcha pulchra* from the Early to Middle Miocene Kadonosawa Formation, which differs from the type specimen by having several ridges in the ventral area, a widely arched antero-dorsal margin, and an acutely arched dorsal margin. Tsukagoshi et al. ^225^ discovered this species in a Holocene borehole core from Central Honshu, Japan. However, their specimen was identified as *L. ocellata* based on its carapace outline and lack of inflation in the postero-ventral area. Zheng et al.^32^ reported this species from the South China Sea; however, it differs by having a broadly arched anterior margin, a narrower flat area along the posterior margin, and a longer dorsal margin. Irizuki et al. ^229^ figured *Loxoconcha pulchra* from the Early Miocene Akeyo Formation, Central Japan. This specimen differs from the type specimen by having several ridges in the ventral area, a widely arched antero-dorsal margin, and an acutely arched dorsal margin.

**Occurrence:**

*Recent*: South Korea; Shikoku, Osaka Bay, and Central Honshu, Japan.

*Fossil*: Middle Pleistocene from Kyushu, Osaka Bay, and Central Japan; Holocene from Southwest and Central Honshu, Japan.

**13.** *Loxoconcha tosaensis* Ishizaki, 1968^1^

v*. 1968 *Loxoconcha tosaensis* Ishizaki, p. 31, 32, pl. 2, fig. 3, pl. 7, figs.6-9^1^.

v. 1971 *Loxoconcha tosaensis* Ishizaki; Ishizaki, p. 87, pl. 3, fig. 18^3^.

v. 1975 *Loxoconcha tosaensis* Ishizaki; Ishizaki, p. 54, fig. 2^241^.

v. 1977 *Loxoconcha (Loxoconcha)* *tosaensis* Ishizaki; Hanai et al., p. 63^8^.

non. 1985 *Loxoconcha tosaensis* Ishizaki; Ishizaki and Matoba, pl. 4, figs. 16, 17^13^.

v. 1990 *Loxoconcha tosaensis* Ishizaki; Takayasu et al., pl. 1, fig. 14^212^.

v. 1992 *Loxoconcha tosaensis* Ishizaki; Iwasaki, p. 11, pl. 1, fig. 5^242^.

v. 1993 *Loxoconcha tosaensis* Ishizaki; Kamiya and Nakagawa, p. 133, pl. 6, figs. 6, 7^14^.

non. 1994 *Loxoconcha tosaensis* Ishizaki; Zheng et al., pl. 53, figs. 11, 12^32^.

v. 1998 *Loxoconcha tosaensis* Ishizaki; Yamane, p. 47, pl. 6, fig. 6^35^.

v. 2001 *Loxoconcha tosaensis* Ishizaki; Yasuhara and Irizuki: p. 83, pl. 6, figs. 15-20^151^.

v. 2001 *Loxoconcha tosaensis* Ishizaki; Kamiya et al., 101, fig. 17.11^36^.

v. 2002 *Loxoconcha tosaensis* Ishizaki; Irizuki et al., p. 39, pl. 1, figs. 16, 17^152^.

v. 2002 *Loxoconcha tosaensis* Ishizaki; Yasuhara et al., p. 635, fig. 3.7^228^.

v. 2004 *Loxoconcha tosaensis* Ishizaki; Yasuhara et al., p. 31, fig. 13k, p. 32, fig. 14k, p. 33, fig. 15k^230^.

v. 2005 *Loxoconcha tosaensis* Ishizaki; Irizuki et al., p. 42, fig. 4.14^162^.

v. 2005 *Loxoconcha tosaensis* Ishizaki; Ishida and Takayasu, p. 76, fig. 2.9^216^.

v. 2005 *Loxoconcha tosaensis* Ishizaki; Ishii et al., p. 83-85, 88, 92, 93, fig. 1G, 3, 4, 7-9, tables 1, 2, 4^153^.

v. 2006 *Loxoconcha tosaensis* Ishizaki; Irizuki et al., p. 25, fig. 8.6^39^.

v. 2006 *Loxoconcha tosaensis* Ishizaki; Yasuhara and Seto, p. 106, fig. 4m^243^.

non. 2008 *Loxoconcha (Hanaiconchas) tosaensis* Ishizaki; Hu and Tao, p. 479-480, pl. 22, fig. 16, pl. 39, fig. 21, pl. 150, fig. 12^17^.

v. 2008 *Loxoconcha tosaensis* Ishizaki; Irizuki et al., p. 295, fig. 7^244^.

v. 2009 *Loxoconcha tosaensis* Ishizaki; Irizuki et al., p. 5, fig. 4.10^163^.

v. 2009 *Loxoconcha tosaensis* Ishizaki; Ozawa, p. 238, 242, fig. 4.6^218^.

v. 2010 *Loxoconcha tosaensis* Ishizaki; Irizuki et al., p. 16, fig. 4.14^245^.

v. 2011 *Loxoconcha tosaensis* Ishizaki; Kawano et al., p. 5, fig. 3.11^198^.

v. 2013 *Loxoconcha tosaensis* Ishizaki; Kawano et al., p. 96, fig. 5.8^200^.

v. 2019 *Loxoconcha tosaensis* Ishizaki; Yoo and Karanovic, p. 119-120, fig. 3^167^.

v. 2015 *Loxoconcha tosaensis* Ishizaki; Irizuki et al., p. 154, fig. 4.9^245^.

v. 2015 *Loxoconcha tosaensis* Ishizaki; Irizuki et al., p.470, fig. 3.22^246^.

non. 2018 *Loxoconcha bispinosa* Kajiyama; Irizuki et al., p. 643, fig. 5.1^157^.

non. 2018 *Loxoconcha bispinosa* Kajiyama; Irizuki et al., p. 48, fig. 6^247^.

v. 2019 *Loxoconcha tosaensis* Ishizaki; Tanaka et al., p. 34, fig. 4j^248^.

v. 2019 *Loxoconcha tosaensis* Ishizaki; Tanaka et al., p. 10, fig. 2.12-15^203^.

v. 2020 *Loxoconcha bispinosa* Kajiyama; Sasaki et al., p. 8, fig. 4.5^249^.

v. 2021 *Loxoconcha tosaensis* Ishizaki; Irizuki et al., p. 7, fig. 6.11^250^.

**Type:** Male left valve (Holotype), IGPS 90272.

**Remarks:** Ishizaki^1^ illustrated the internal line drawing of the holotype (pl. 2, fig. 3) and provided an optical photograph of the specimen (pl. 7, fig. 6). This included optical photographs of the male right valve (paratype, pl. 7, fig. 8), as well as the female left valve (paratype, pl. 7, fig. 7) and right valve (paratype, pl.7, fig. 9). Ishii et al.^153^ demonstrated ontogenetic changes in the number of pore canals and noted the fossil record of this species from the Late Pliocene onward. Ozawa^218^ identified a specimen with a developed postero-ventral spine in this species. Yoo and Karanovic^167^ presented SEM images of the male left and right valves, line drawings of the antenna, mandible, maxilla, and male copulatory organ. Specimens figured by Ishizaki et al.^13^ from the Late Pliocene Sasaoka Formation is not identified this species in that it has a sinuate dorsal margin, mid-posterior tubercle and postero-ventral inflation. Zheng et al.^32^ reported this species from the South China Sea; however, it differs from the type species by having finer reticulation, an acutely arched anterior margin, and a widely arched dorsal margin. The specimens figured by Hu and Tao^17^ from Taiwan are not *L. tosaensis*, as they have a long lateral outline, a narrowly arched anterior margin, and finer reticulation. Irizuki et al.^157,247^ identified *L*. *bispinosa* Kajiyama, 1913^169^ from Holocene borehole cores from Hiuchi-nada, Seto Inland Sea, and from Recent surface sediments in Tsushima Island, Japan. However, the type specimen of *L*. *bispinosa* has not been designated (personal communication with E. Kajiyama by N. Ikeya, 1970s), and no specimens have been found, thus, *L. bispinosa* is a nomen dubium. A redescription of *L. bispinosa* is ongoing based on topotypic material originally examined by Kajiyama.

**Occurrence:**

*Recent*: Coast of South Korea; Tsushima Islands, Kyushu, Shikoku, Osaka Bay, Seto Inland Sea, Japan; the Sea of Japan side of Southwest Japan; Northern Honshu, Japan.

*Fossil*: Middle Pleistocene from Kyushu, Southwest, and Central Honshu, Japan; Late Pleistocene from Central Honshu, Japan; Holocene from the Tsushima Straits, Kyushu, Southwest Honshu, Seto Inland Sea, Sea of Japan side of Southwest Honshu, and Northeast Honshu, Japan.

**14.** *Loxoconcha uranouchiensis* Ishizaki, 1968^1^

v*. 1968 *Loxoconcha uranouchiensis* Ishizaki, p. 32, pl. 7, figs. 2, 3^1^.

v. 1969 *Loxoconcha uranouchiensis* Ishizaki; Ishizaki, p. 220, pl. 26, fig. 15^2^.

v. 1971 *Loxoconcha uranouchiensis* Ishizaki; Ishizaki, p. 87, 88, pl. 3, fig. 7^3^.

non.1976 *Loxoconcha uranouchiensis* Ishizaki; Holden, F. 32-33, pl. 4, fig. 12, pl. 14, figs. 9-11^251^.

1977 *Loxoconcha uranouchiensis* Ishizaki; Herrig, p. 1260-1261, pl. 2, figs. 3a, b, 4^5^.

v. 1977 *Loxoconcha (Loxoconcha)* *uranouchiensis* Ishizaki; Hanai et al., p. 63^8^.

v. 1980 *Loxoconcha uranouchiensis* Ishizaki; Okubo, p. 420-422, fig. 15, 19a-f^173^.

1980 *Loxoconcha uranouchiensis* Ishizaki; Hanai et al., p. 190^252^.

non.1981 *Loxoconcha uranouchiensis* Ishizaki; Gou et al., p. 166, pl. 81, figs. 11, 12^221^.

1985 *Loxoconcha uranouchiensis* Ishizaki; Ishizaki and Matoba: pl. 5, fig. 1^13^.

non. 1987 *Loxoconcha uranouchiensis* Ishizaki; Cai and Chen, pl. 2, fig. 2^24^.

non.1987 *Loxoconcha uranouchiensis* Ishizaki; Ikeya et al., p. 63, fig. 1^147^.

non. 1987 *Loxoconcha uranouchiensis* Ishizaki; Tabuki et al., p. 335, pl. 2, fig. 9^252^.

v. 1988 *Loxoconcha laeta* Ishizaki; Ikeya and Shiozaki, p. 139, pl. 4, figs. 4a, 4b^27^.

v. 1988 *Loxoconcha uranouchiensis* Ishizaki; Kamiya, p. 319, 322, 323-326, 328, 329, 331, pl. 1, figs.7-12, text-figs. 5, 7-9, 12, 13, 15, table 2^178^.

v. 1988 *Loxoconcha uranouchiensis* Ishizaki; Kamiya, p. 339, 341, 345, pl. 1, figs. 1-8, text-figs. 1, 3, 4^179^.

v. 1988 *Loxoconcha uranouchiensis* Ishizaki; Kamiya, p. 307, 308, 310-313, 315, 316, pl. 1, figs. 8-11, text-figs. 4, 5.3, 5.4, 7-9, 11, 13^180^.

non. 1988 *Loxoconcha uranouchiensis* Ishizaki; Ruan and Hao, p. 326, pl. 58, figs. 1-4^28^.

v. 1989 *Loxoconcha uranouchiensis* Ishizaki; Kamiya, p. 39-41, 43-45, pl. 1, figs.3, 4, 9-12. 18, 19, pl. 2, figs. 4-9, 11, figures, 1-8^181^.

v. 1989 *Loxoconcha uranouchiensis* Ishizaki; Kamiya, p. 78, 79, 81-84, figs. 3, 4.3, 4.4, 7-9, 11, 12^182^.

v.1990 *Loxoconcha viva* Ishizaki; Ruan, p. 134, pl. 2, fig. 6^72^.

non. 1990 *Loxoconcha uranouchiensis* Ishizaki; Tabuki and Nohara, pl. 2, fig. 9^254^.

v. 1990 *Loxoconcha uranouchiensis* Ishizaki; Takayasu et al., pl. 1, fig. 15^212^.

v. 1991 *Loxoconcha uranouchiensis* Ishizaki; Ikeya and Itoh, p. 139, fig. 18A^149^.

v. 1992 *Loxoconcha uranouchiensis* Ishizaki; Ikeya and Suzuki, p.131, pl. 6, fig. 4^183^.

non.1992 *Loxoconcha uranouchiensis* Ishizaki; Nohara and Oshiro, p.336, figs. 2.1, 2.2^185^.

v. 1992 *Loxoconcha uranouchiensis* Ishizaki; Kamiya, p. 223-233, figs. 2-14, 16^255^.

v. 1992 *Loxoconcha uranouchiensis* Ishizaki; Kamiya and Hazel, p. 160, 162, 164-166, figs. 1-3, 6, pl. 2^184^.

non. 1993 *Loxoconcha uranouchiensis* Ishizaki; Kamiya and Nakagawa, p. 133, pl. 6, fig. 8^14^.

v. 1995 *Loxoconcha uranouchiensis* Ishizaki; Tabuki and Nohara, p. 348, fig. 4.7^256^.

v. 1998 *Loxoconcha uranouchiensis* Ishizaki; Irizuki et al., p. 8, fig. 3.8^150^.

v. 1998 *Loxoconcha uranouchiensis* Ishizaki; Tanaka et al., p. 91, pl. 1, fig. 16^257^.

v. 2000 *Loxoconcha uranouchiensis* Ishizaki; Irizuki and Hosoyama, p. 12, fig. 3.17^226^.

v. 2001 *Loxoconcha uranouchiensis* Ishizaki; Yasuhara and Irizuki: p. 85, pl. 7, figs. 1, 2^151^.

v. 2001 *Loxoconcha* cf. *uranouchiensis* Ishizaki; Yasuhara and Irizuki: p. 85, pl. 7, figs. 3-5^151^.

v. 2001 *Loxoconcha uranouchiensis* Ishizaki; Kamiya et al., 103, fig. 18.7^36^.

v. 2001 *Loxoconcha uranouchiensis* Ishizaki; Nakao et al., p. 133, fig. 5.16^257^.

non. 2001 *Loxoconcha uranouchiensis* Ishizaki; Tabuki, p. 39, fig. 14.6^186^.

v. 2002 *Loxoconcha uranouchiensis* Ishizaki; Irizuki et al., p. 39, fig. 18^152^.

v. 2002 *Loxoconcha uranouchiensis* Ishizaki; Tanaka and Ikeya, p.266, fig.1^188^.

v. 2002 *Loxoconcha uranouchiensis* Ishizaki; Yasuhara et al., p.635, fig.3.8^228^.

v. 2004 *Loxoconcha uranouchiensis* Ishizaki; Horne et al., p. 287, fig. 27^190^.

2004 *Loxoconcha uranouchiensis* Ishizaki; Yamauchi, p. 73^38^.

v. 2004 *Loxoconcha uranouchiensis* Ishizaki; Yasuhara et al., p. 31, fig. 13m, p. 32, fig. 14m, p. 33, fig. 15m^230^.

non. 2004 *Loxoconcha uranouchiensis* Ishizaki; Irizuki, p. 77, pl. 3, fig. 3^37^.

v. 2005 *Loxoconcha uranouchiensis* Ishizaki; Irizuki et al., p. 42, fig. 4.15^162^.

v. 2005 *Loxoconcha uranouchiensis* Ishizaki; Ishii et al., p. 83-85, 87, 88, 92, 93, fig. 1B, 2, 3, 4, 7-9, tables 1, 2, 4^153^.

v. 2005 *Loxoconcha uranouchiensis* Ishizaki; Tanaka, p. 237, 238, figs. 2d, 2e, 3j, 3k, 3l^259^.

2006 *Loxoconcha uranouchiensis* Ishizaki; Irizuki et al., p. 25, fig. 8.7^39^.

2006 *Loxoconcha uranouchiensis* Ishizaki; Schornikov, p.44^16^.

2006 *Loxoconcha uranouchiensis* Ishizaki; Schornikov and Zanina, p.217, table 2^15^.

v. 2007 *Loxoconcha uranouchiensis* Ishizaki; Hou and Gou, pl. 153, fig. 9; pl. 169, figs. 20, 21^106^.

v. 2007 *Loxoconcha uranouchiensis* Ishizaki; Sasaki et al., p. 524, fig. 5.27^154^.

v. 2008 *Loxoconcha uranouchiensis* Ishizaki; Nakao et al., p. 281, fig. 3.F^260^.

non. 2008 *Loxoconcha (Palmoconcha) uranouchiensis* Ishizaki; Hu and Tao, p. 465, pl. 30, figs. 23, 25, pl. 31, figs. 3, 6, pl. 39, fig. 20, pl. 40, figs. 6, 19, pl. 58, fig. 2, pl. 219, figs. 11, 14, 16, pl. 225, figs. 11, 19^17^.

v. 2008 *Loxoconcha uranouchiensis* Ishizaki; Irizuki et al., p. 295, fig. 7^244^.

v. 2008 *Loxoconcha uranouchiensis* Ishizaki; Nakao et al., p. 281, fig. 3.F^260^.

v. 2009 *Loxoconcha uranouchiensis* Ishizaki; Horikoshi et al., p. 154, fig. 5.7^261^.

v. 2012 *Loxoconcha uranouchiensis* Ishizaki; Tanaka et al., p. 12-13, pl. 2, fig. 8^40^.

v. 2012 *Loxoconcha uranouchiensis* Ishizaki; Tanaka et al., p. 9, fig. 4I^237^.

v. 2013 *Loxoconcha uranouchiensis* Ishizaki; Kawano et al., p. 96, fig. 5.9^200^.

v. 2014 *Loxoconcha uranouchiensis* Ishizaki; Schornikov and Zanina, p. 35, pl. 6, figs. 7, 8^156^.

v. 2014 *Loxoconcha uranouchiensis* Ishizaki; Masuma and Yamada, p. 6, fig. 4.2^41^.

non. 2014 *Loxoconcha uranouchiensis* Ishizaki; Tabuki and Nakanishi, p. 264, pl. 1, fig. 7^262^.

v. 2017 *Loxoconcha uranouchiensis* Ishizaki; Tsukagoshi, p. 20, figs. 8.3, 8.4, 9-11^201^.

v. 2018 *Loxoconcha uranouchiensis* Ishizaki; Yamada et al., p. 337, fig. 5.7^166^.

v. 2019 *Loxoconcha uranouchiensis* Ishizaki; Irizuki et al., p. 267, fig. 5.19^168^.

v. 2019 *Loxoconcha uranouchiensis* Ishizaki; Niiyama et al., p. 105, fig. 4.7^133^.

v. 2019 *Loxoconcha uranouchiensis* Ishizaki; Tanaka et al., p. 34, fig. 4k^248^.

v. 2019 *Loxoconcha uranouchiensis* Ishizaki; Tanaka et al., p. 11, fig. 3.7-11^203^.

v. 2021 *Loxoconcha uranouchiensis* Ishizaki; Irizuki et al., p. 7, fig. 6.12^250^.

**Type:** Male left valve (Holotype), IGPS 90276.

**Remarks:** Ishizaki^1^ provided an optical photograph of the specimen (pl. 7, fig. 3) alongside an optical photograph of a female left valve (paratype, pl. 7, fig. 2). Okubo^173^ described the soft parts of this species. Kamiya^178^ mentioned that this species inhabits the sandy bottom of *Zostera* beds, with its population density gradually decreasing from January to July. According to Kamiya^178^, the body length of females increases during low temperatures and decreases during high temperatures; and this size structure is preserved in subfossil assemblages. Moreover, its juvenile ratio is low from spring to summer. Kamiya^179^ noted that the ratio of females and males in this species is equal. Kamiya^180^ observed that the copulation time for this species ranges from several minutes to about 30 minutes. Kamiya^181^ illustrated the shape and distribution of twisted and smooth-type bristles on the shell of this species. Kamiya^255^ identified two forms (large and small) in this species based on shell size and outline, different ornamentation, and copulatory organs. Furthermore, he found that the breeding seasons for the two different forms were nearly the same (autumn to winter) and that interbreeding between the forms did not occur, indicating that speciation was complete. Kamiya and Hazel^184^ observed that the smooth bristles constantly increase throughout each molting stage, while the twisted bristle increase only slightly after the A-4 stages. Ishii et al.^153^ documented the ontogenetic change in the number of pore canals and noted its fossil record from the Late Pliocene onward. Tanaka^259^ provided images of thin sections of this species’ median and lateral eyes. Horden^251^ identified *Loxoconcha uranouchiensis* from the Late Cenozoic drilling core samples at Midway Island; however, the figured specimen differs from the type specimen in having prominent antero-marginal ridges, several short ridges running from the mid-anterior area to the central area, and a lack of a flat postero-ventral area. Gou et al. ^221^ illustrated this species from the Pliocene borehole cores, but it differs in having an antero-ventrally arched anterior margin, a mid-ventrally inflated area, and concentrically arranged reticulation. Cai and Chen^24^ identified this species from the surface sediment of the South China Sea; however, it differs in having an acutely arched anterior margin, a flat posterior area, and concentric ornamentation. Ruan and Hao^28^ illustrated specimens from Late Pleistocene to Holocene borehole cores from the Okinawa Trough, Japan. These differ from this species by having an acutely arched anterior margin, concentrically arranged reticulation, and a protruded postero-ventral carinal ridge. Ikeya et al.^147^ illustrated a specimen that is not *Loxoconcha uranouchiensis* but is *L. viva*, characterized by a short postero-dorsal ridge, a more sinuate dorsal margin, and finer reticulation at the posterior area. *Loxoconcha uranouchiensis* reported by Tabuki et al.^253^ from Sekisei-sho, Southern Ryukyus, Japan, differs from the type specimen by having an acutely arched anterior margin, pitted ornamentation at the postero-dorsal area, and a concentric pattern of surface ornamentation. Tabuki and Nohara^254^ identified this species from Sekisei-sho, Okinawa, Japan; however, it differs in having an acutely arched anterior margin, a fine pit at the postero-dorsal area, and vertically arranged posterior reticulation. Nohara and Oshiro^185^ illustrated this species from Late Pleistocene sediments of the Ikebaru Gravel, Okinawa, Japan; however, it differs by having larger normal pores, V-shaped caudal ridges at the postero-ventral area, and a narrow flat area along the anterior margin. Kamiya and Nakagawa^14^ illustrated this species; however, compared with the type species, their specimen is identified as *Loxoconcha epeterseni*. Irizuki^40^ illustrated this species from the Early Pleistocene Masuda Formation, Tanega-Shima Island, Japan. However, it differs from the type species by having concentrically arranged reticulation, a carinal ridge along the dorsal margin, and lacking carinal ridges running parallel from ventral to posterior margins. All images illustrated by Hu and Tao^17^ as *L. (p.) uranouchiensis* are not identified as this species because these specimens have regular concentric ornamentation, widely arched postero-ventral carinas, and arched posterior margins. Tabuki and Nakanishi^262^ illustrated this species, but it differs by having a straight dorsal margin, no ventral carinal ridge, and no postero-ventral flat area.

**Occurrence:**

*Recent*: Co To Islands, Northern Vietnam; Guangxi, China; Sesoko Island, Okinawa, Japan; Kyushu, Shikoku, Seto Inland Sea, Central Honshu, Sendai Bay, Japan; Sea of Japan side of Southwest Japan; Pacific side of Central Honshu, Northern Honshu, Japan; Peter the Great Bay, Russia.

*Fossil*: Pliocene, Leizhou Peninsula, South China; Middle Pleistocene, Kyushu, Southwest and Central Honshu, Japan; Holocene borehole core from Tsushima Straits, Southwest and Central Honshu, Northeast Honshu, Japan.

**15.** *Loxoconcha viva* Ishizaki, 1968^1^

v*. 1968 *Loxoconcha viva* Ishizaki, p. 33, pl. 7, figs. 12-14^1^.

v. 1971 *Loxoconcha viva* Ishizaki; Ishizaki, p. 88, pl. 3, fig. 20^3^.

v. 1977 *Loxoconcha (Loxoconcha)* *viva* Ishizaki; Hanai et al., p. 63^8^.

v. 1982 *Loxoconcha viva* Ishizaki; Hou et al., p. 208-209, pl. 78, fig. 23^20^.

1982 *Loxoconcha viva* Ishizaki; Hou et al., p. 208-209, pl. 78, figs. 24-26, text-fig. 52^20^.

non.1982 *Loxoconcha viva* Ishizaki; Hou et al., pl. 79, figs. 3-6^20^.

v. 1984 *Loxoconcha viva* Ishizaki; Ishizaki, p. 43, pl. 2, fig. 11^222^.

v.1987 *Loxoconcha uranouchiensis* Ishizaki; Ikeya et al., p. 63, fig. 1^147^.

v.p.1988 *Loxoconcha viva* Ishizaki; Ruan and Hao, p. 326-327, pl. 58, fig. 5^28^.

non.1989 *Loxoconcha viva* Ishizaki; Ruan, pl. 1, fig. 26^161^.

non.1990 *Loxoconcha viva* Ishizaki; Ruan, p. 134, pl. 2, fig. 6^72^.

non. 1991 *Loxoconcha viva* Ishizaki; Ikeya and Itoh, p. 139, fig. 18B^149^.

non. 1992 *Loxoconcha viva* Ishizaki; Ikeya and Suzuki, p. 131, pl. 6, fig. 5^183^.

non. 1996 *Loxoconcha viva* Ishizaki; Kamiya et al., p. 165, figs. 7, 8^263^.

non. 1996 *Loxoconcha viva* Ishizaki; Ozawa, p. 112, pl. 6, fig. 9^33^.

non. 1998 *Loxoconcha viva* Ishizaki; Irizuki et al., p. 8, fig. 3.9^150^.

non. 1998 *Loxoconcha viva* Ishizaki; Yamane, p. 47, pl. 6, fig. 8^35^.

non. 2000 *Loxoconcha viva* Ishizaki; Irizuki and Hosoyama, p. 12, fig. 3.18^226^.

v. 2001 *Loxoconcha viva* Ishizaki; Yasuhara and Irizuki: p. 85, pl. 7, figs. 6-17^151^.

2001 *Loxoconcha viva* Ishizaki; Kamiya et al., 99, fig. 16.7^36^.

v. 2004 *Loxoconcha viva* Ishizaki; Yasuhara et al., p. 31, fig. 13g, p. 32, fig. 14g, p. 33, fig. 15g^230^.

v. 2006 *Loxoconcha tosaensis* Ishizaki; Yasuhara and Seto, p. 106, fig. 4n^243^.

v. 2007 *Loxoconcha viva* Ishizaki; Nakao et al., p. 281, fig. 3G^260^.

v. 2007 *Loxoconcha viva* Ishizaki; Sasaki et al., p. 524, fig. 5.23^154^.

non. 2008 *Loxoconcha (Lochmanoconcha) viva* Ishizaki; Hu and Tao, p. 492, pl. 11, fig. 16, pl. 50, figs. 4, 7, 13, pl. 88, figs. 10, 12, pl. 122, fig. 19, pl. 191, fig. 12^17^.

v. 2008 *Loxoconcha uranouchiensis* Ishizaki; Nakao et al., p. 281, fig. 3.G^260^.

v. 2009 *Loxoconcha viva* Ishizaki; Irizuki et al., p. 5, fig. 4.11^163^.

non. 2009 *Loxoconcha viva* Ishizaki; Tanaka and Nomura, p. 62, fig. 4.15^196^.

non. 2010 *Loxoconcha viva* Ishizaki; Irizuki et al., p. 16, fig. 4.6^245^.

non. 2011 *Loxoconcha viva* Ishizaki; Irizuki et al., p. 2035, fig. 5.^264^

non. 2011 *Loxoconcha viva* Ishizaki; Irizuki et al., p. 43, fig. 4.15^219^.

2012 *Loxoconcha viva* Ishizaki; Tanaka et al., p. 13^40^.

non. 2012 *Loxoconcha viva* Ishizaki; Tanaka et al., p. 117, pl. 1, fig. 12^199^.

v. 2012 *Loxoconcha viva* Ishizaki; Tanaka et al., p. 9, fig. 4J^237^.

non. 2015 *Loxoconcha viva* Ishizaki; Irizuki et al., p. 153, fig. 4.10^245^.

non. 2015 *Loxoconcha viva* Ishizaki; Irizuki et al., p.470, fig. 3.22^265^.

v. 2016 *Loxoconcha viva* Ishizaki; Matsushima et al., p. 14, fig. 6F^238^.

v. 2019 *Loxoconcha viva* Ishizaki; Tanaka et al., p. 34, fig. 4l^248^.

non. 2021 *Loxoconcha viva* Ishizaki; Maehama et al., p. 371, fig. 6.20^266^.

**Type:** Male left valve (Holotype), IGPS 90278.

**Remarks:** Ishizaki^1^ provided optical photograph of the specimen (pl. 7, fig. 13) along with the optical photographs of a female right valve (paratype, pl. 7, fig. 12) and left valve (paratype, pl. 7, fig. 14). Hou et al.^20^ illustrated adult and juvenile of *L. viva* from Quaternary sediments of Jiangsu, China, but some of their specimens differ from the type specimen in having an elongated lateral outline, an acutely arched posterior margin, and a straight ventral margin. Ruan^161^ found this species on the coast of Hubei; however, it differs from the type specimen in having a longer lateral outline, a straight dorsal margin, and a fine pit at the dorsal area. Ikeya and Itoh^149^ found this species in Sendai Bay, Japan; however, they identified it as *L. epeterseni* based on its ornamentation and outline. Ikeya and Suzuki^183^ reported this species from the Sea of Japan side of Southwest Honshu, Japan; however, it differs in having a dorsally arched anterior margin, a widely arched dorsal margin, and an inflated valve. Irizuki et al.^150^ illustrated a specimen that is not *Loxoconcha viva* but *L. epeterseni*, characterized by a short postero-dorsal ridge, a widely arched ventral margin, and an acutely arched anterior margin. The specimen illustrated by Kamiya et al.^263^ and Ozawa^33^ from the Early Pleistocene Omma Formation does not match the type species, as it has a straight ventral margin, no short postero-dorsal ridge, and three mid-posterior ridges. The specimen illustrated by Yamane^35^ from the recent sediment of Hiuchi-nada, Seto Inland Sea, Southwest Japan, is not identified as *L*. *viva* because it lacks a postero-dorsal carinal ridge, has a sinuate ventral margin, and a wider postero-ventral flat area. Irizuki and Hosoyama^226^ reported this species from the Middle Pleistocene Noma Formation, Central Japan; however, their specimen is inconsistent with the type specimen, lacking a sinuate ventral margin and featuring an arched dorsal margin with no sinuation. Hu and Tao^17^ illustrated and identified specimens as this species, but these are not *L. viva*, as they have dorsally arched anterior margins, two prominent carinas from the antero-ventral to the postero-ventral area, and no short carina from the dorsoposterior area to the mid-posterior area. Tanaka and Nomura^196^ illustrated this species from the Late Miocene Maja Formation, Kumejima Island, Okinawa, Japan, but it differs from the type specimen by having two prominent carinal ridges diverging from the central area to the anterior, a broader flat area at the posterior, and a less sinuate ventral margin. Irizuki et al.^245,246^ found this species in Kasado Bay, Yamagushi Prefecture, Southwest Japan; however, it differs by having a shorter and thinner carinal ridge at the postero-dorsal area, several vertical carinal ridges from the posterior margin, and a straight ventral margin. Irizuki et al.^219^ illustrated this species from the Middle Pleistocene borehole core in Saitama Prefecture, Central Japan; however, it differs by having a widely arched ventral margin, a widely arched posterior margin, and a regular concentric reticulation pattern along the ventral area. Tanaka et al.^199^ found this specimen in the Holocene Numa Formation, Central Honshu, Japan; however, it differs by having concentric reticulation, a narrow anterior flat area, and a widely arched dorsal margin. The specimen illustrated by Irizuki et al.^265^ from the Holocene borehole cores in Mutsu Bay, North Japan, differs from the type specimen by having a narrow flat area along the anterior margin, a widely arched ventral margin, and a laterally inflated valve.

**Occurrence:**

*Recent*: Kyushu, Shikoku, Seto Inland Sea, Northern Honshu, Japan.

*Fossil*: Quaternary Jiangsu, China; Holocene borehole core from Okinawa Trough, Seto Inland Sea, Central Honshu, Japan; Holocene Southwest and Central Honshu, Japan.

**16.** *Loxoconcha zamia* Ishizaki, 1968^1^

v*. 1968 *Loxoconcha zamia* Ishizaki, p. 33, 34, pl. 7, figs. 10, 11^1^.

v. 1977 *Loxoconcha (Loxoconcha)* *zamia* Ishizaki; Hanai et al., p. 63^8^.

non. 1988 *Loxoconcha zamia* Ishizaki; Ikeya and Shiozaki, p. 139, pl. 4, fig. 2^27^.

v. 1992 *Loxoconcha zamia* Ishizaki; Ikeya and Suzuki, p. 131, pl. 6, fig. 6^183^.

v. 1993 *Loxoconcha zamia* Ishizaki; Kamiya and Nakagawa, p. 133, pl. 6, fig. 9^14^.

v. 2001 *Loxoconcha zamia* Ishizaki; Kamiya et al., 103, fig. 18.3^36^.

v. 2004 *Loxoconcha zamia* Ishizaki; Irizuki, p. 77, pl. 3, fig. 4^37^.

v. 2005 *Loxoconcha zamia* Ishizaki; Ishii et al., p. 83, 84, 86, 87, 88, 92, 93, figs. 1I, 2, 3, 4, 7-9, tables 1, 2, 4^153^.

v.p. 2008 *Loxoconcha (Argoconcha) zamia* Ishizaki; Hu and Tao, p. 469, pl. 50, figs. 2, 3, 5, 9, pl. 73, figs. 11, 14^17^.

non. 2008 *Loxoconcha (Argoconcha) zamia* Ishizaki; Hu and Tao, pl. 73, fig. 20^17^.

2012 *Loxoconcha zamia* Ishizaki; Tanaka et al., p. 13^40^.

**Type:** Male detached carapace (Holotype), IGPS 90281.

**Remarks:** Ishizaki^1^ provided optical photographs of the left and right valves of the specimen. Ishii et al.^153^ documented the ontogenetic change in the number of pore canals and noted its fossil record from the Late Quaternary onward. The specimen illustrated by Ikeya and Shiozaki^27^ from the Holocene sediment of Central Japan, is not *L. zamia* but *L*. *kattoi*, based on its widely arched anterior margin, sinuate dorsal margin, and irregular postero-dorsal carinal ridge. One specimen (pl. 73, fig. 20) illustrated by Hu and Tao^17^ is not *L. zamia*; it has a sinuate dorsal margin, a tubercle at the antero-dorsal area, and no flat area at the posterior.

**Occurrence:**

*Recent*: Shikoku, Southwest Japan, Sea of Japan side Southwest Honshu, Japan.

*Fossil*: Late Pliocene, Taiwan; Early Pleistocene Northern Ryukyu, Japan; Holocene Southwest Honshu, Japan.

Genus: *Miia* Ishizaki, 1968^1^

Type Species: *Miia uranouchiensis* Ishizaki, 1968^1^

**17.** *Miia uranouchiensis* Ishizaki, 1968^1^

v*. 1968 *Miia uranouchiensis* Ishizaki, p. 35, pl. 2, figs. 5, 6, pl. 6, figs. 21, 22^1^.

v. 1968 *Loxoconcha* sp. B Ishizaki, p. 34, pl. 6, figs. 17, 18^1^.

v. 1977 *Miia uranouchiensis* Ishizaki; Hanai et al., p. 65^8^.

v. 1985 *Miia uranouchiensis* Ishizaki; Ikeya et al., pl. 8, figs. 8, 9^12^.

v. 2001 *Loxoconcha viva* Ishizaki; Yasuhara and Irizuki: p. 87, pl. 8, figs. 4, 5^151^.

**Type:** Female left valve (Holotype), IGPS 90286.

**Remarks:** Ishizaki^1^ provided a line drawing of the holotype specimen (pl. 2, fig. 5), along with an optical photograph of its external view (pl. 6, fig. 22). Additionally, he included a line drawing of the right valve of the paratype specimen (paratype 90287, pl. 2, fig. 6) and its optical photograph (pl. 6, fig. 21). Base on the maximum length of the paratype specimen, we identified this specimen as a male.

**Occurrence:**

*Recent*: Shikoku, Southwest Honshu, Japan.

*Fossil*: Late Pleistocene Central Honshu, Japan.

Genus: *Cytheromorpha* Hirschmann, 1909^267^

Type Species: *Cythere acupunctata* Brady, 1880^268^

**18.** *Cytheromorpha acupunctata* (Brady, 1880) ^268^

v*. 1880 *Cythere acupunctata* Brady, p. 68, pl. 14, fig. 1a-h^268^.

v. 1959 *Cythere acupunctata* Brady; Hanai, p. 428^170^.

v. 1961 *Cytheromorpha acupunctata* (Brady); Hanai, p. 371, text-fig. 12, fig. 2a, b^144^.

v. 1968 *Cytheromorpha acupunctata* (Brady); Ishizaki, p. 35, 36, pl. 7, figs. 17, 18^1^.

non. 1968 *Cytheromorpha japonica* Ishizaki, p. 35, pl. 2, figs. 5, 6, pl. 6, figs. 21, 22^1^.

v. 1969 *Cytheromorpha acupunctata* (Brady); Ishizaki, p. 220, pl. 26, figs. 5, 6^2^.

non. 1969 *Cytheromorpha japonica* Ishizaki; Ishizaki p. 221, pl. 26, fig. 16^2^.

v. 1971 *Cytheromorpha acupunctata* (Brady); Ishizaki, p. 90, pl. 3, figs. 13^3^.

v. 1975 *Cytheromorpha acupunctata* (Brady); Ishizaki, p. 56^241^.

v. 1976 *Cythere acupunctata* Brady; Puri and Hulings, p. 268, pl. 8, fig. 5^269^.

v. 1978 *Cytheromorpha acupunctata* (Brady); Okubo, p. 91-93, figs. 1a-f, 2, 4a-h^270^.

v. 1980 *Cytheromorpha* sp. A; Kim and Park, p. 47, pl. 1, fig. 7^271^.

1980 *Cytheromorpha acupunctata* (Brady); Okubo, p. 395^173^.

non. 1982 *Cytheromorpha japonica* Ishizaki; Hou et al. p. 213, pl. 79, figs.23, 24^20^.

v. 1982 *Cytheromorpha acupunctata* (Brady); Hou et al. p. 213-214, pl. 79, figs. 19-22^20^.

v. 1983 *Cytheromorpha acupunctata* (Brady); Ikeya, p.2, fig. 1.3^272^.

v. 1984 *Cytheromorpha acupunctata* (Brady); Ishizaki, p. 42, pl. 1, fig. 3^222^.

v. 1985 *Cytheromorpha acupunctata* (Brady); Ikeya et al., pl. 8, figs. 7, 10-12^12^.

v. 1985 *Cytheromorpha acupunctata* (Brady); Zhao, pl. 2, fig. 17^160^.

non. 1986 *Cytheromorpha japonica* Ishizaki; Lin and Zhu, pl. 3, fig. 2^273^.

non. 1986 *Cytheromorpha acupunctata* (Brady); Zheng, pl. 2, figs. 23-25^274^.

v.1987 *Cytheromorpha acupunctata* (Brady); Ikeya et al., p. 63, fig. 11^147^.

1987 *Cytheromorpha acupunctata* (Brady); Zheng, p. 199, pl. 6, fig. 15^25^.

v. 1988 *Cytheromorpha acupunctata* (Brady); Ikeya and Kashima, p. 202, fig. 4^275^.

v. 1988 *Cytheromorpha acupunctata* (Brady); Ruan and Hao, p. 328, pl. 55, figs. 34, 35^28^.

v. 1988 *Cytheromorpha acupunctata* (Brady); Wang, et al., p. 103, pl. 1, fig. 26^276^.

v. 1988 *Cytheromorpha japonica* Ishizaki; Wang, et al., p. 103, pl. 1, fig. 27^276^.

v. 1989 *Cytheromorpha acupunctata* (Brady); Ruan, pl. 1, figs. 28, 29^161^.

v. 1990 *Cytheromorpha acupunctata* (Brady); Yajima and Lord, p. 158, pl. 34, figs. 7, 8, text-fig. 70^213^.

v. 1990 *Cytheromorpha acupunctata* (Brady); Lee, p. 363-365, pl. 34, figs. 5.4, 5.5^29^.

v. 1990 *Cytheromorpha acupunctata* (Brady); Takayasu et al., pl. 1, fig. 16^212^.

v. 1991 *Cytheromorpha acupunctata* (Brady); Ikeya and Itoh, p. 135, fig. 14C^149^.

v. 1992 *Cytheromorpha acupunctata* (Brady); Iwasaki, p. 11, pl. 1, fig. 10^242^.

v. 1992 *Cytheromorpha acupunctata* (Brady); Lee and Paik, p. 149, pl. 2, fig. 18^31^.

v. 1993 *Cytheromorpha acupunctata* (Brady); Ikeya, p. 26, figs. 3.1a-3.1c^277^.

v. 1993 *Cytheromorpha acupunctata* (Brady); Ikeya and Shiozaki, p. 17, 29, 30, figs. 1, 13, 14^278^.

v. 1994 *Cytheromorpha acupunctata* (Brady); Tsukagoshi et al., p. 50, 58, fig. 2-29^225^.

v. 1995 *Cytheromorpha acupunctata* (Brady); Ikeya, p. 31, fig. 3^279^.

v. 1996 *Cytheromorpha acupunctata* (Brady); Ozawa, p. 109, pl. 3, fig. 10^33^.

v. 1998 *Cytheromorpha acupunctata* (Brady); Cao, pl. 4, figs. 16, 17^34^.

v. 1998 *Cytheromorpha acupunctata* (Brady); Irizuki et al., p. 8, fig. 3.10^150^.

v. 1998 *Cytheromorpha acupunctata* (Brady); Kim et al., p. 133, pl. 4, fig. 10^280^.

v. 1998 *Cytheromorpha acupunctata* (Brady); Tanaka et al., p. 91, pl. 1, fig. 5^257^.

v. 1998 *Cytheromorpha acupunctata* (Brady); Yamane, p. 43, pl. 4, fig. 6^35^.

v. 2000 *Cytheromorpha acupunctata* (Brady); Irizuki and Hosoyama, p. 12, fig. 3.15^226^.

v. 2001 *Cytherois uranouchiensis* Ishizaki; Yasuhara and Irizuki: p. 81, pl. 5, figs. 1-4^151^.

v. 2002 *Cytheromorpha acupunctata* (Brady); Irizuki et al., p. 39, pl. 1, figs. 19, 20^152^.

v. 2002 *Cytheromorpha acupunctata* (Brady); Nakao and Tsukagoshi, p. 100, 102, fig. 19^227^.

v. 2003 *Cytheromorpha acupunctata* (Brady); Irizuki et al., p. 155, fig. 5^281^.

v. 2002 *Cytheromorpha acupunctata* (Brady); Yasuhara et al., p. 635, fig. 3.13^228^.

v. 2003 *Cytheromorpha acupunctata* (Brady); Yamaguchi, p. 135, fig. 1J^203^.

v. 2003 *Cytheromorpha acupunctata* (Brady); Yasuhara and Kumai, p. 77, pl. 2, fig. 4^282^.

2004 *Cytheromorpha acupunctata* (Brady); Yamauchi, p. 73^38^.

v. 2005 *Cytheromorpha acupunctata* (Brady); Irizuki et al., p. 42, fig. 4.16^162^.

2006 *Cytheromorpha acupunctata* (Brady); Schornikov, p.43^16^.

v. 2006 *Cytheromorpha acupunctata* (Brady); Schornikov and Zanina, p .222, pl. 4, figs. 3, 4^15^.

v. 2006 *Cytheromorpha acupunctata* (Brady); Yasuhara and Seto, p. 106, fig. 4k^243^.

v. 2006 *Cytheromorpha acupunctata* (Brady); Irizuki et al., p. 25, fig. 8.8^39^.

v. 2007 *Cytheromorpha acupunctata* (Brady); Sasaki et al., p. 524, fig. 5.26^154^.

2007 *Cytheromorpha acupunctata* (Brady); Schornikov and Zanina, p. 202^283^.

non. 2008 *Neosinocythere acupunctata* (Brady); Hu and Tao, p. 345, pl. 70, fig. 21, pl. 213, figs. 13, 20^17^.

v. 2008 *Cytheromorpha acupunctata* (Brady); Irizuki et al., p. 294, fig. 6^244^.

v. 2008 *Cytheromorpha acupunctata* (Brady); Nakao et al., p. 281, fig. 3.C^260^.

v. 2008 *Cytheromorpha acupunctata* (Brady); Nakao and Tsukagoshi, p. 275, pl. 7, figs. E-H^234^.

v. 2009 *Cytheromorpha acupunctata* (Brady); Horikoshi et al., p. 154, fig. 5.5^261^.

v. 2009 *Cytheromorpha acupunctata* (Brady); Irizuki et al., p. 5, fig. 4.12^163^.

v. 2010 *Cytheromorpha acupunctata* (Brady); Irizuki et al., p. 16, fig. 4.18^245^.

v. 2011 *Cytheromorpha acupunctata* (Brady); Irizuki et al., p. 2035, fig. 5^264^.

v. 2011 *Cytheromorpha acupunctata* (Brady); Irizuki et al., p. 42, fig. 4.16^219^.

v. 2011 *Cytheromorpha acupunctata* (Brady); Kawano et al., p. 5, fig. 3.5^198^.

v. 2012 *Cytheromorpha acupunctata* (Brady); Tanaka et al., L05406, fig. 1f^284^.

2012 *Cytheromorpha acupunctata* (Brady); Tanaka et al., p. 13^40^.

v. 2013 *Cytheromorpha acupunctata* (Brady); Kawano et al., p. 96, fig. 5.4^200^.

v. 2014 *Cytheromorpha acupunctata* (Brady); Masuma and Yamada, p. 5, fig. 3.18^41^.

v. 2015 *Cytheromorpha acupunctata* (Brady); Irizuki et al., p. 153, fig. 4.11^245^.

v. 2015 *Cytheromorpha acupunctata* (Brady); Irizuki et al., p. 470, fig. 3.24^265^.

v. 2015 *Cytheromorpha acupunctata* (Brady); Yamada et al., p. 57, fig. 3.8^42^.

v. 2016 *Cytheromorpha acupunctata* (Brady); Matsushima et al., p. 14, fig. 6D^238^.

v. 2017 *Cytheromorpha acupunctata* (Brady); Kamiyama et al., p. 125, fig. 3Q^240^.

v. 2018 *Cytheromorpha acupunctata* (Brady); Irizuki et al., p. 643, fig. 5.3^157^.

v. 2018 *Cytheromorpha acupunctata* (Brady); Irizuki et al., p. 47, fig. 5^247^.

v. 2018 *Cytheromorpha acupunctata* (Brady); Yamada et al., p. 337, fig. 5.3^166^.

v. 2019 *Cytheromorpha acupunctata* (Brady); Irizuki et al., p. 267, fig. 5.24^168^.

v. 2019 *Cytheromorpha acupunctata* (Brady); Tanaka et al., p. 34, fig. 4g^248^.

v. 2019 *Cytheromorpha acupunctata* (Brady); Tanaka et al., p. 10, fig. 2.1^203^.

v. 2021 *Cytheromorpha acupunctata* (Brady); Irizuki et al., p. 7, fig. 6.10^250^.

v. 2022 *Cytheromorpha acupunctata* (Brady); Kaneko et al., p. 65, pl. 3, fig. 5^220^.

v. 2024 *Cytheromorpha acupunctata* (Brady); Maekawa, fig. 182.71^285^.

**Type:** Right valve (Lectotype), BM1974.292.

**Remarks:** The specimen named by Ishizaki^1^ as *Cytheromorpha japonica* (male right valve, Holotype, IGPS 90290) is now considered a junior synonym of *Cytheromorpha acupunctata* (Brady, 1880)^268^. Okubo^270^ illustrated the soft parts of this species. Ikeya and Shiozaki^278^ noted that this species adapts to inner bay with a depth of 2 m, coarse sand, salinity levels of 27–29 PSU around Honshu and Shikoku, Japan. Nakao and Tsukagoshi^227^ reported that this species is mainly found in 27 PSU in the Obitsugawa Estuary, Central Honshu, Japan. Based on SEM images from Nakao and Tsukagoshi^227^, Ishizaki’s type specimen is confirmed to be male. Yamaguchi^203^ analyzed 18S rDNA of this species from Tsukumo Bay, Southwest Honshu, Japan. Zheng^274^ discovered this species in Quaternary sediment in Zhejiang, China, but the figured specimen does not belong to the genus *Cytheromorpha*. Hu and Tao^17^ established a new genus, *Neosinocythere*, including *Cytheromorpha acupunctata* (Brady, 1880)^268^ as identified by Ishizaki^1,2^. However, the specimens figured by Hu and Tao^17^ are different species, lacking an evenly arched anterior margin, trapezoidal lateral outline, and a completely reticulated outer surface.

**Occurrence:**

*Recent*: Coast near Shanghai, Hubei, China; Shangdong, North China; Nagdong Estuary, Korea; Kyushu, Tsushima Island, Seto Inland Sea, Shikoku, and Honshu, Japan; Sea of Japan side of Southwest Japan; Northern Honshu, Japan; Peter the Great Bay, Russia.

*Fossil*: Plio-Pleistocene Central Honshu, Japan; Pleistocene Cheju Island, Korea; Middle Pleistocene, Kyushu, Southwest and Central Honshu, Japan; Late Pleistocene Southwest Honshu, Japan; Quaternary sediments from Jiangsu, China; Quaternary borehole cores from Hong Kong and Bohai Sea, China; Holocene of Okinawa Trough, Japan; Holocene borehole cores from Ulleung Basin, Korea; Holocene borehole cores from Tsushima Straits, Kyushu, Seto Inland Sea, Sea of Japan side of Southwest and Central Honshu, and Pacific side of Northeast Honshu, Japan.

Genus: *Nipponocythere* Ishizaki, 1968^1^

Type Species: *Cythere bicarinate* Brady, 1880^268^

**19.** *Nipponocythere bicarinata* (Brady, 1880)^268^

v*. 1880 *Cythere bicarinata* Brady, p. 70, pl. 16, fig. 6a-d^268^.

v. 1959 *Cythere bicarinata* Brady; Hanai, p. 428^170^.

non. 1971 *Nipponocythere asamushiensis* Ishizaki, p. 89, pl. 1, fig. 1, pl. 5, figs. 7, 12, 13, pl. 6, fig. 2, pl. 7, figs. 8, 11^3^.

non. 1976 *Cythere acupunctata* Brady; Puri and Hulings, p. 269, pl. 10, figs. 12, 13^269^.

v. 1980 *Nipponocythere* sp. A Kim and Park, p. 47, pl. 1, figs. 22-25^271^.

non. 1982 *Nipponocythere asamushiensis* Ishizaki; Cai, p. 6, pl. 2, figs. 16, 17^21^.

non. 1982 *Nipponocythere asamushiensis* Ishizaki; Hou et al., p. 212, pl. 79, figs. 16-18^20^.

v. 1982 *Nipponocythere bicarinata* (Brady); Yajima, p. 223, pl. 13, figs. 1-4^286^.

v. 1984 *Nipponocythere bicarinata* (Brady); Ishizaki, p. 42, pl. 1, fig. 10^222^.

v. 1985 *Nipponocythere bicarinata* (Brady); Wang and Zhao, p. 340, pl. 8, fig. 8^287^.

non. 1986 *Nipponocythere bicarinata* (Brady); Lin and Zhu, p. 97, pl. 2, fig. 8^273^.

v.1987 *Nipponocythere bicarinata* (Brady); Ikeya et al., p. 63, figs. 6-10^147^.

v. 1987 *Nipponocythere bicarinata* (Brady); Wang and Zhang, p. 290, pl. 1, fig. 24^146^.

v. 1988 *Nipponocythere bicarinata* (Brady); Wang, et al., p. 103, pl. 1, fig. 23^276^.

non. 1988 *Nipponocythere asamushiensis* Ishizaki; Ruan and Hao, p. 333, pl. 59, fig. 25-27^28^.

non. 1988 *Nipponocythere bicarinata* (Brady); Ruan and Hao, p. 333, pl. 59, fig. 28-31^28^.

non. 1989 *Nipponocythere asamushiensis* Ishizaki; Ruan, pl. 2, fig. 5^161^.

v. 1990 *Nipponocythere bicarinata* (Brady); Yajima and Lord, p. 157, figs. 4.1, 4.2^213^.

v. 1990 *Nipponocythere bicarinata* (Brady); Zhao and Wang, pl. 1, fig. 21^148^.

v. 1991 *Nipponocythere bicarinata* (Brady); Ikeya and Itoh., p. 141, fig. 20B^149^.

v. 1992 *Nipponocythere bicarinata* (Brady); Iwasaki, p. 11, pl. 1, fig. 6^242^.

v. 1998 *Nipponocythere bicarinata* (Brady); Irizuki et al., p. 8, fig. 3-11^150^.

v. 1998 *Nipponocythere bicarinata* (Brady); Kim et al., p. 133, pl. 4, fig. 14^280^.

v. 1998 *Nipponocythere bicarinata* (Brady); Tanaka et al., p. 91, pl. 1, fig. 15^257^.

v. 1998 *Nipponocythere bicarinata* (Brady); Yamane, p. 51, pl. 8, fig. 3^35^.

v. 2001 *Nipponocythere asamushiensis* Ishizaki; Yasuhara and Irizuki, p. 87, pl. 8, figs. 13-20^151^.

v. 2001 *Nipponocythere asamushiensis* Ishizaki; Kamiya et al., 101, fig. 17.12^36^.

v. 2001 *Nipponocythere asamushiensis* Ishizaki; Tabuki, p. 33, fig. 10.15^186^.

v. 2005 *Nipponocythere bicarinata* (Brady); Irizuki et al., p. 42, fig. 4.17^162^.

v. 2006 *Nipponocythere bicarinata* (Brady); Irizuki et al., p. 26, fig. 8.10^39^.

v. 2007 *Nipponocythere bicarinata* (Brady); Sasaki et al., p. 524, fig. 5.24^154^.

v. 2008 *Gouiecythere asamushiensis* (Ishizaki); Hu and Tao, p. 441, pl. 11, fig. 18, pl. 29, fig. 23^17^.

v. 2008 *Nipponocythere bicarinata* (Brady); Nakao et al., p. 281, fig. 3.H^260^.

v. 2009 *Nipponocythere bicarinata* (Brady); Irizuki et al., p. 320, fig. 3.16^288^.

v. 2009 *Nipponocythere bicarinata* (Brady); Irizuki et al., p. 5, fig. 4.13^163^.

v. 2009 *Nipponocythere bicarinata* (Brady); Tanaka and Nomura, p. 62, figs. 4.13^196^.

v. 2009 *Nipponocythere bicarinata* (Brady); Ozawa, p. 238, figs. 4.7, 4.8^218^.

v. 2010 *Nipponocythere bicarinata* (Brady); Irizuki et al., p. 16, fig. 4.16^245^.

v. 2011 *Nipponocythere bicarinata* (Brady); Irizuki et al., p. 2035, fig. 5^264^.

v. 2011 *Nipponocythere bicarinata* (Brady); Irizuki et al., p.42, fig. 4.17^219^.

v. 2012 *Nipponocythere bicarinata* (Brady); Tanaka et al., L05406, fig. 1d^284^.

v. 2012 *Nipponocythere bicarinata* (Brady); Tanaka et al., p. 14, pl. 2, fig. 12^40^.

non. 2012 *Nipponocythere asamushiensis* Ishizki; Tanaka et al., p. 117, pl. 1, fig. 14^199^.

v. 2012 *Nipponocythere bicarinata* Ishizki; Tanaka et al., p. 9, fig. 4K^237^.

v. 2013 *Nipponocythere bicarinata* (Brady); Kawano et al., p. 96, fig. 5.11^200^.

v. 2015 *Nipponocythere bicarinata* (Brady); Irizuki et al., p. 470, fig 3.25^265^.

v. 2018 *Nipponocythere bicarinata* (Brady); Irizuki et al., p. 643, fig. 5.4^157^.

v. 2018 *Nipponocythere bicarinata* (Brady); Irizuki et al., p. 48, fig. 6^247^.

v. 2019 *Nipponocythere bicarinata* (Brady); Hong et al., p. 596, fig. 5^158^.

v. 2019 *Nipponocythere bicarinata* (Brady); Tanaka et al., p. 34, fig. 4m^248^.

v. 2022 *Nipponocythere bicarinata* (Brady); Jöst et al., figs. 1-3^289^.

**Type:** Left valve (Lectotype), BM1974.282; right valve (Lectotype), BM 1974.281.

**Remarks:** The specimen named by Ishizaki^1^ as *Nipponocythere asamushiensis* (male right valve, Holotype, IGPS 91567) is a junior synonym of *Nipponocythere bicarinata* (Brady, 1880)^268^. *Nipponocythere bicarinata* figured by Lin and Zhu^273^ from Laizhou Bay, Bohai Sea, differs from the type specimen in having an anteriorly projecting anterior margin, a lack of a caudal process, and a straight dorsal margin. Ruan and Hao^28^ figured this species from Late Pleistocene and Holocene borehole cores from Okinawa Trough, Japan; however, their specimen differs in having a round lateral outline, a ventrally arched carinal ridge, and the longest point located one-fourth from the venter. Hu and Tao^17^ established a new genus *Gouiecythere*, and included the type specimen of *Nipponocythere asamushiensis* Ishizaki, 1971^3^ as a synonym.

**Occurrence:**

*Recent*: South China Sea; Hong Kong, China; East China Sea; shelf south to central China; Nagdong Estuary, Korea; Tsushima Island, Kyushu, Seto Inland Sea; Southwest and Central Honshu, Japan; Sendai Bay, Northeast Honshu, Japan.

*Fossil*: Late Pliocene of Okinawa, Japan; Late Pliocene and Pliocene of Taiwan; Middle Pleistocene of Kyushu, Southwest and Central Honshu, Japan; Late Pleistocene of Okinawa Trough, Japan; Late Pleistocene of Central Japan; Plio-Pleitocene of Okinawa, Japan; Quaternary borehole cores from the Bohai Sea, China; Holocene borehole cores from Hong Kong, China; Holocene borehole core from the Ulleung Basin, Korea; Holocene borehole core from the Tsushima Straits, Kyushu, Seto Inland Sea, Southwest and Central Honshu, Japan; Holocene sediment from Central Honshu, Japan; Holocene borehole cores from Northern Japan.

Family: Cytheruridae Müller, 1894^290^

Genus: *Angulicytherura* Schornikov and Dolgov, 1995^291^

Type Species: *Angulicytherura urupica* Schornikov and Dolgov, 1995^291^

**20.** *Angulicytherura miii* (Ishizaki, 1969)^2^

v*. 1969 *Tetracytherura miii* Ishizaki, p. 216-217, pl. 24, figs. 1-3, pl. 26, figs. 10, 11^2^.

v. 1971 *Tetracytherura miii* Ishizaki; Ishizaki, p. 79, pl. 2, fig. 17^3^.

v. 1977 *Cytherura* ? *miii* (Ishizaki); Hanai et al., p. 54^8^.

non. 1985 *Cytherura* ? *miii* (Ishizaki); Yajima and Ikeya, p. 606, pl. 1, figs. 3, 6^292^.

v. 1990 *Cytherura miii* Ishizaki; Takayasu et al., pl. 1, fig. 11^212^.

v. 1993 *Cytherura* ? *miii* (Ishizaki); Kamiya and Nakagawa, p. 131, pl. 5, figs. 5, 6^14^.

v. 1996 *Cytherura* *miii* (Ishizaki); Tsukagoshi and Kamiya, p. 361, figs. 13E-13H^293^.

v. 2000 *Angulicytherura* *miii* (Ishizaki); Tsukagoshi and Parker, p. 119, fig. 4C, D^294^.

v. 2002 *Angulicytherura* ? *miii* (Ishizaki); Nakao and Tsukagoshi, p. 91-92, figs. 12, 13A-D^227^.

v. 2002 *Cytherura* *miii* (Ishizaki); Yasuhara et al., p. 635, fig. 3.11^228^.

v. 2003 *Cytherura* *miii* (Ishizaki); Irizuki et al., p. 156, fig. 7^281^.

2004 *Angulicytherura* ? *miii* (Ishizaki); Yamauchi, p. 73^38^.

2006 *Angulicytherura* ? *miii* (Ishizaki); Schornikov, p.38^16^.

2006 *Angulicytherura* ? *miii* (Ishizaki); Schornikov and Zanina, p.217, table 2^15^.

v. 2008 *Angulicytherura* ? *miii* (Ishizaki); Nakao and Tsukagoshi, p. 273, pl. 5, figs. E-H^234^.

v. 2014 *Angulicytherura* *miii* (Ishizaki); Masuma and Yamada, p. 5, fig. 3.2^41^.

v. 2017 *Angulicytherura* *miii* (Ishizaki); Kamiyama et al., p. 125, fig. 3M^240^.

v. 2017 *Angulicytherura* *miii* (Ishizaki); Tsukagoshi, p. 22, fig. 13^201^.

v. 2018 *Angulicytherura* *miii* (Ishizaki); Yamada et al., p. 337, fig. 5.1^166^.

**Type:** Female right valve (Holotype), IGPS 90328.

**Remarks:** Ishizaki^2^ provided an internal line drawing (pl. 24, fig. 2) and an external optical photograph (pl. 26, fig.10) of the holotype. The current reexamination presents the external view of the holotype. Tsukagoshi and Parker^294^ displayed an SEM image of the trunk region of this species. Nakao and Tsukagoshi^227^ described the soft parts and provided SEM images of both male and female valves. They also noted that the primary distribution of this species is within 4 to 23 PSU in the Obitsugawa Estuary, Central Japan. According to the SEM images of Nakao and Tsukagoshi^227^, Ishizaki’s type specimen is identified as female. Yajima and Ikeya^292^ included this species in their figures from drilling core samples of DSDP Leg 87 in the Nankai Trough. However, this species was not correctly identified because the specimen exhibited a postero-dorsally protruded caudal process, a nonbranched inner carinal ridge at two-third the height from the venter, and an acutely arched anterior margin.

**Occurrence:**

*Recent*: Seto Inland Sea, Southwestern, Central, and Northern Honshu, Japan.

*Fossil*: Holocene Southwest Honshu, Japan; Holocene borehole core from the Sea of Japan side of Southwestern Honshu, Japan.

Genus: *Howeina* Hanai, 1957^295^

Type Species: *Howeina camptocytheroidea* Hanai, 1957^295^

**21.** *Howeina* *higashimeyaensis* Ishizaki, 1971^3^

v. 1971 *Howeina higashimeyaensis* Ishizaki, p. 80-81, pl. 7, figs. 1-4^3^.

1982 *Howeina higashimeyaensis* Ishizaki; Yajima, p. 174, table1-5^286^.

non. 1985 *Howeina higashimeyaensis* Ishizaki; Ishizaki and Matoba, pl. 4, fig. 11^13^.

1986 *Howeina higashimeyaensis* Ishizaki; Tabuki, p. 41, table 1^296^.

non. 1986 *Howeina higashimeyaensis* Ishizaki; Cheong et al., p. 48, pl. 3, fig. 7^145^.

non. 1990 *Howeina higashimeyaensis* Ishizaki; Yajima and Lord, p. 157, fig. 4.8^213^.

non. 1993 *Howeina higashimeyaensis* Ishizaki; Ishizaki et al., p. 329, fig. 7D^215^.

non. 1996 *Howeina higashimeyaensis* Ishizaki; Kamiya et al., p. 161, pl. 2, fig. 7^263^.

non. 1996 *Howeina higashimeyaensis* Ishizaki; Ozawa, p. 111, pl. 5, fig. 7^33^.

1996 *Howeina higashimeyaensis* Ishizaki; Ozawa, p. 100, table 3, p. 105, table 6^33^.

1997 *Howeina higashimeyaensis* Ishizaki; Tsukawaki et al., p. 56, table 2^297^.

1999 *Howeina higashimeyaensis* Ishizaki; Schornikov and Sokolenko, p. 216^298^,

2000 *Howeina higashimeyaensis* Ishizaki; Lee et al., p. 467, appendix^299^.

2000 *Howeina higashimeyaensis* Ishizaki; Tsukawaki et al., p. 106, table 5b^299^.

non. 2001 *Howeina higashimeyaensis* Ishizaki; Kamiya et al., 97, fig. 15.19^36^.

non. 2004 *Howeina higashimeyaensis* Ishizaki; Ozawa et al., p. 13, fig. 1.9^300^.

2005 *Howeina higashimeyaensis* Ishizaki; Ozawa and Kamiya, p. 172, table 4, p. 176, table 8^301^.

2005 *Howeina higashimeyaensis* Ishizaki; Ozawa and Kamiya, p. 266, table 6^217^.

non. 2006 *Howeina higashimeyaensis* Ishizaki; Ozawa, fig. 1.9^302^.

2006 *Howeina higashimeyaensis* Ishizaki; Schornikov, p. 39^16^.

2006 *Howeina higashimeyaensis* Ishizaki; Schornikov and Zanina, p .218, table. 2^15^.

non. 2007 *Howeina higashimeyaensis* Ishizaki; Ozawa, fig. 1.5^303^.

non. 2010 *Howeina higashimeyaensis* Ishizaki; Ozawa, p. 33, pl. 3, fig. 2^304^.

non. 2014 *Howeina higashimeyaensis* Ishizaki; Schornikov and Zanina, p. 36, pl. 8, figs. 11, 12^156^.

**Type:** Female right valve (Holotype), IGPS 90350.

**Remarks:** Ishizaki^3^ provided SEM images of the holotype, including external (pl. 7, fig.1) and internal (pl. 7, fig. 4) views. He also illustrated SEM images of the paratype’s left external and internal views. However, these specimens were from the Pliocene Higashimeya Formation, not the Recent Aomori Bay. The extant specimen differs from the fossil specimen in several ways: it has a narrower caudal process, a prominent ridge running parallel to the postero-dorsal margin, a strong ridge extending from the mid-dorsal area to the antero-ventral area, and a straight ventral margin. The fossil record of *Howeina higashimeyaensis* is uncertain, and a taxonomic reassessment of the fossil specimens is necessary. In this paper, we tentatively classify the fossil specimens of *H. higashimeyaensis* as “non*.*” The specimen reported by Ozawa^33^ differs from the holotype in having a twisted carinal ridge extending from the mid-anterior area to the central area, a sinuate ventral margin, and reticulation. Irizuki et al.^229^ identified the Ozawa’s^33^ specimen as *Semicytherura mainensis*.

**Occurrence:**

*Recent*: Northen Honshu, Japan.

*Fossil*: unknown.

Family: Paradoxostomatidae Brady and Norman, 1889^305^

Genus: *Cytherois* Müller, 1884^306^

Type Species: *Cytherois virens* Müller, 1884^306^

**22.** *Cytherois asamusiensis* Ishizaki, 1971^3^

v*. 1971 *Cytherois asamushiensis* Ishizaki, p. 90-91, pl. 1, figs. 6, pl. 3, figs. 12, 15^3^.

v. 1977 *Cytherois asamushiensis* Ishizaki; Hanai et al., p. 74^8^.

v.p. 1990 *Cytherois asamushiensis* Ishizaki; Lee, p. 370-371, pl. 35, fig. 5^29^.

v. 1991 *Cytherois asamushiensis* Ishizaki; Ikeya and Itoh, p. 135, fig. 14B^149^.

v. 1995 *Cytherois asamushiensis* Ishizaki; Ozawa et al., p. 31, pl. 1, fig. 8^204^.

2006 *Paracytheroma asamushiensis* (Ishizaki); Schornikov, p.37^16^.

v. 2014 *Paracytheroma asamushiensis* (Ishizaki); Schornikov and Zanina, p.32, pl. 2, figs. 18, 19^156^.

non 2015 *Paracytheroma asamushiensis* (Ishizaki); Irizuki et al., p. 470, fig. 3.2^265^.

**Type:** Right valve (Holotype), IGPS 91575.

**Remarks:** Ishizaki^3^ provided an internal line drawing (pl. 1, fig. 6) and an external optical photograph (pl.3, fig.12) of the holotype, as well as an optical photograph of the external view of a left valve (IGPS 91576, paratype, pl. 3, fig. 15). The current re-examination presents the external view of the holotype. Irizuki et al. ^265^ identified this species in a Holocene drilling core from Northern Japan. However, their specimen differs from the type specimen by having an anterior-ventrally projecting anterior margin, a broadly arching posterior margin, and a short, straight section on the dorsal margin.

**Occurrence:**

*Recent*: Sendai Bay, Aomori Bay, Northern Honshu, Japan; and Peter the Great Bay, Russia.

*Fossil*: Pleistocene Cheju Island, Korea; and Middle Pleistocene Central Honshu, Japan.

**23.** *Cytherois nakanoumiensis* Ishizaki, 1969^2^

v*. 1969 *Cytherois nakanoumiensis* Ishizaki, p. 221, pl. 26, figs. 3, 4, pl. 24, figs. 7, 8^2^.

v. 1971 *Cytherois nakanoumiensis* Ishizaki; Ishizaki, p. 91, pl. 3, fig. 11^3^.

v. 1977 *Cytherois nakanoumiensis* Ishizaki; Hanai et al., p. 74^8^.

v.p. 1982 *Cytherois uranouchiensis* Ishizaki; Cai, p. 5, pl. 1, fig. 29^21^.

v. 2001 *Cornucoquimba tosaensis* (Ishizaki); Yasuhara and Irizuki, p. 79, pl. 4, figs. 18, 19^151^.

v. 2009 *Cytherois nakanoumiensis* Ishizaki; Irizuki et al., p. 5, fig. 4.14^163^.

v. 2014 *Cytherois nakanoumiensis* Ishizaki; Masuma and Yamada, p. 5, fig. 3.15^41^.

v. 2015 *Cytherois nakanoumiensis* Ishizaki; Irizuki et al., p.470, fig 3.26^265^.

v. 2015 *Cytherois nakanoumiensis* Ishizaki; Yamada et al., p.56, fig 3.6^42^.

**Type:** Female right valve (Holotype), IGPS 87018.

**Remarks:** Ishizaki^2^ provided a line drawing of the inner view (pl. 24, fig. 8) and optical photographs of the external view (pl. 26, fig. 3) of the holotype. He also included a line drawing of the inner view (pl. 24, fig. 7) and an optical photograph of the external view (pl. 26, fig. 4) of a paratype (IGPS 87019). The current re-examination focuses on the holotype specimen.

**Occurrence:**

*Recent*: South China Sea; the Sea of Japan side of Honshu, Northern Honshu, Japan; Seto Inland Sea, Japan.

*Fossil*: Holocene in Southwest Honshu, Japan; Holocene borehole cores from the Sea of Japan side of Southwestern Japan; and Northern Honshu, Japan.

**24.** *Cytherois uranouchiensis* Ishizaki, 1968^1^

v*. 1968 *Cytherois uranouchiensis* Ishizaki, p. 36-37, pl. 2, figs. 12, 13, pl. 8, figs. 7, 8^1^.

v. 1969 *Cytherois uranouchiensis* Ishizaki; Ishizaki, p. 221, pl. 26, figs. 1, 2^2^.

v. 1971 *Cytherois uranouchiensis* Ishizaki; Ishizaki, p. 91, pl. 4, fig. 20^3^.

v. 1977 *Cytherois uranouchiensis* Ishizaki; Hanai et al., p. 74^8^.

non. 1982 *Cytherois uranouchiensis* Ishizaki; Cai, p. 5, pl. 1, fig. 29, 30^21^.

v. 1984 *Cytherois uranouchiensis* Ishizaki; Ishizaki, p. 43, pl. 2, fig. 6^222^.

v. 2001 *Cytherois uranouchiensis* Ishizaki; Yasuhara and Irizuki, p. 79, pl. 4, figs. 20, 21^151^.

v. 2008 *Cytherois uranouchiensis* Ishizaki; Irizuki et al., p. 295, fig. 7^244^.

v. 2009 *Cytherois uranouchiensis* Ishizaki; Irizuki et al., p. 5, fig. 4.15^163^.

v. 2014 *Cytherois nakanoumiensis* Ishizaki; Masuma and Yamada, p. 5, fig. 3.16^41^.

v. 2015 *Cytherois uranouchienss* Ishizaki; Irizuki et al., p. 470, fig. 3.27^265^.

v. 2015 *Cytherois uranouchiensis* Ishizaki; Yamada et al., p. 57, fig. 3.7^42^.

v. 2019 *Cytherois uranouchiensis* Ishizaki; Irizuki et al., p. 267, fig. 5.25^168^.

**Type:** Right valve (Holotype), IGPS 90295.

**Remarks:** Ishizaki^2^ provided a line drawing of the inner view (pl. 2, fig. 12) and optical photographs of the external view (pl. 8, fig. 7) of the holotype. He also included a line drawing of the inner view (pl. 2, fig. 13) and an optical photograph of the external view (pl. 8, fig. 8) of a left valve from the paratype (IGPS 90296). The current re-examination focuses on the holotype specimen, which has a broken ventral margin. Additionally, one figure (pl. 1 fig. 29) of *Cytherois uranouchiensis* reported by Cai^21^ from the South China Sea has been identified as *Cytherois nakanoumiensis*.

**Occurrence:**

*Recent*: Kyushu, Shikoku, Southwest and Northern Japan; Sea of Japan side of Honshu, Seto Inland Sea, Japan.

*Fossil*: Middle Pleistocene borehole cores from Southwest Honshu, Japan; Holocene Southwest Honshu, Japan; Holocene borehole cores from the Sea of Japan side of Honshu, Japan; Northern Honshu, Pacific side of Northeast Honshu, Japan.

Genus: *Paracytherois* Müller, 1894^290^

Type Species: *Bythocythere flexuosa* Brady, 1867^307^

**25.** *Paracytherois mutsuensis* Ishizaki, 1971^3^

v*. 1971 *Paracytherois mutsuensis* Ishizaki, p. 91, pl. 1, figs. 10, 11, pl. 4, figs. 21-23^3^.

v. 1977 *Paracytherois mutsuensis* Ishizaki; Hanai et al., p. 75^8^.

v. 2001 *Paracytherois tosaensis* Ishizaki; Yasuhara and Irizuki, p. 89, pl. 9, fig. 1^151^.

non. 2008 *Cytherois mutsuensis* Ishizaki; Hu and Tao, p. 516, pl. 204, figs. 5, 18^17^.

**Type:** Female carapace (Holotype), IGPS 91579.

**Remarks:** Ishizaki^3^ illustrated the external left lateral view of the holotype with an optical photograph (pl. 4, fig.22), as well as line drawings and optical photographs of the internal view of a right valve (IGPS 91580, paratype, pl. 1, fig. 11, pl. 4, fig. 21) and another right valve (IGPS 91581, paratype, pl. 1, fig. 10, pl. 4, fig. 23). The current reexamination revealed that the holotype had appendages. The specimens depicted by Hu and Tao^17^ from recent collections in Taiwan do not correspond to this species, as they exhibit a highest point positioned posteriorly in the lateral view, rounder posterior and anterior margins, and a straight ventral margin.

**Occurrence:**

*Recent*: Southwest and Northern Honshu of Japan.

*Fossil*: unknown.

**26.** *Paracytherois tosaensis* Ishizaki, 1968^1^

v*. 1968 *Paracytherois tosaensis* Ishizaki, p. 37, pl. 2, figs. 10, 11, pl. 9, figs. 7, 8^1^.

v. 1969 *Paracytherois tosaensis* Ishizaki; Ishizaki, p. 221, pl. 26, fig. 14^2^.

v. 1971 *Paracytherois tosaensis* Ishizaki; Ishizaki, p. 91, pl. 4, fig. 17^3^.

v. 1977 *Paracytherois tosaensis* Ishizaki; Hanai et al., p. 75^8^.

v.p. 1988 *Paracytherois tosaensis* Ishizaki; Ruan and Hao, p. 344, pl. 61, fig. 22^28^.

non. 1988 *Paracytherois tosaensis* Ishizaki; Ruan and Hao, p. 344, pl. 61, fig. 20, 21^28^.

v. 1998 *Paracytherois tosaensis* Ishizaki; Tanaka et al., p. 91, pl. 1, fig. 9^257^.

v. 2003 *Paracytherois tosaensis* Ishizaki; Irizuki et al., p. 13, pl. 1, fig. 10^308^.

v.p. 2008 *Cytherois tosaensis* Ishizaki; Hu and Tao, p. 515-516, pl. 107, fig. 11, pl. 204, fig. 9^17^.

non. 2008 *Cytherois tosaensis* Ishizaki; Hu and Tao, pl. 107, fig. 17, pl. 204, figs. 4, 6, 19, text-fig. 293^17^.

**Type:** Left valve (Holotype), IGPS 90297.

**Remarks:** Ishizaki^2^ provided a line drawing of the inner view (pl. 2, fig. 10) and optical photographs of the external view (pl. 9, fig. 8) of the holotype. He also included a line drawing of the inner view (pl. 2, fig. 11) and an optical photograph of the external view (pl. 9, fig. 7) of a paratype (IGPS 90298). The current re-examination focuses on the holotype specimen, which exhibits a rounded posterior margin. The specimens (pl. 61, figs. 20 and 21) illustrated by Ruan and Hao^28^ from Late Pleistocene to Holocene borehole cores in the Okinawa Trough, Japan, do not correspond to the type specimens, as they feature an antero-ventrally arched anterior margin, a postero-dorsally arched posterior margin, and a rounded lateral outline. Additionally, some specimens identified as this species by Hu and Tao^17^ differ from the type specimen in having a shorter lateral outline, an acutely curved postero-ventral margin, and an arched postero-dorsal margin (pl. 107, fig. 17). Their illustrated specimen (text-fig. 293B) does not belong to the genus *Cytherois* but to *Paradoxostoma* or *Boreostoma*.

**Occurrence:**

*Recent*: Shikoku; the Sea of Japan side of Southwest Honshu; Northern Honshu, Japan.

*Fossil*: Middle Pleistocene in Central Honshu, Japan; Late Pleistocene in Southern Taiwan; Holocene in the Okinawa Trough, Japan.

Family: Xestoleberididae Sars, 1928^309^

Genus: *Xestoleberis* Sars, 1866^5^

Type Species: *Cythere aurantia* Baird, 1838^310^

**27.** *Xestoleberis hanaii* Ishizaki, 1968^1^

v. 1961 *Xestoleberis* sp. Hanai, p. 363, text-fig. 7, fig. 2a, 2b^144^.

v*. 1968 *Xestoleberis hanaii* Ishizaki, p. 41-42, pl. 9, figs. 1, 2^1^.

v. 1971 *Xestoleberis hanaii* Ishizaki; Ishizaki, p. 95, pl. 4, fig. 14^3^.

v. 1974 *Xestoleberis hanaii* Ishizaki; Schornikov, p.184-186, text-fig. 260^311^.

v. 1977 *Xestoleberis hanaii* Ishizaki; Hanai et al., p. 66^8^.

v. 1979 *Xestoleberis hanaii* Ishizaki; Okubo, p. 9-10, fig. 1d-g^312^.

1980 *Xestoleberis hanaii* Ishizaki; Okubo, p. 429^173^.

1982 *Xestoleberis hanaii* Ishizaki; Cai, p. 7, pl. 2, figs. 14, 15^21^.

v. 1984 *Xestoleberis hanaii* Ishizaki; Okubo, p. 19-41, pl. 1, figs. 2-6, tables 1-10^313^.

v. 1985 *Xestoleberis hanaii* Ishizaki; Ikeya et al., pl. 8, figs. 16, 18, 22^12^.

v. 1985 *Xestoleberis hanaii* Ishizaki; Ishizaki and Matoba, pl. 6, fig. 6^13^.

1986 *Xestoleberis hanaii* Ishizaki; Zhao. et al., pl. 2, fig. 10^314^.

v. 1987 *Xestoleberis hanaii* Ishizaki; Wang and Zhang, p. 290, pl. 1, fig. 8^146^.

non. 1987 *Xestoleberis hanaii* Ishizaki; Whatley and Zhao, p. 23, 28-19, pl. 1, figs. 12, 13^315^.

v. 1988 *Xestoleberis hanaii* Ishizaki; Cai, pl. 2, fig. 20^26^.

v. 1988 *Xestoleberis hanaii* Ishizaki; Ikeya and Shiozaki, p. 135, pl. 2, figs. 1a, 1b^27^.

v. 1988 *Xestoleberis hanaii* Ishizaki; Ruan and Hao, p. 371, pl. 68, figs. 19-22^28^.

non. 1988 *Xestoleberis hanaii* Ishizaki; Wang, et al., p. 103, pl. 1, fig. 31^276^.

v. 1988 *Xestoleberis hanaii* Ishizaki; Kamiya, p. 307, text-fig. 4^180^.

v. 1989 *Xestoleberis hanaii* Ishizaki; Kamiya, p. 78, 85, 86, figs. 3, 12, 13.3, 14.5^182^.

v. 1990 *Xestoleberis hanaii* Ishizaki; Lee, p. 366, pl. 34, figs. 10, 11^29^.

v. 1991 *Xestoleberis hanaii* Ishizaki; Abe and Vannier, p. 586, fig. 3^316^.

v. 1991 *Xestoleberis hanaii* Ishizaki; Ikeya and Itoh, p. 145, fig. 24D^149^.

non. 1992 *Xestoleberis hanaii* Ishizaki; Mostafawi, p. 155, pl. 6, fig. 135^74^.

v. 1992 *Xestoleberis hanaii* Ishizaki; Ikeya and Suzuki, p. 137, pl. 9, fig. 6^183^.

1992 *Xestoleberis hanaii* Ishizaki; Yajima, p. 264^317^.

non. 1994 *Xestoleberis hanaii* Ishizaki; Yumoto, p. 640, 642, 645, 647, figs. 1, 4-6^318^.

v. 1994 *Xestoleberis hanaii* Ishizaki; Zheng et al., pl. 60, figs. 11, 14^32^.

1998 *Xestoleberis hanaii* Ishizaki; Cao, pl. 10, figs. 12, 13^34^.

v. 1998 *Xestoleberis hanaii* Ishizaki; Kamiya and Nakagawa, p. 133, pl. 6, fig. 11^14^.

v. 1998 *Xestoleberis hanaii* Ishizaki; Irizuki et al., p. 8, fig. 3.12^150^.

v. 2000 *Xestoleberis hanaii* Ishizaki; Tsukagoshi and Parker, p. 20, fig. 5C, D^294^.

v. 2000 *Xestoleberis hanaii* Ishizaki; Ikeya and Kato, p. 149-159, figs. 1-10, tables 1, 2^319^.

v. 2001 *Xestoleberis hanaii* Ishizaki; Kamiya et al., 93, fig. 13.17^36^.

v. 2001 *Xestoleberis hanaii* Ishizaki; Yasuhara and Irizuki, p. 95, pl. 12, figs. 14^151^.

v. 2001 *Xestoleberis hanaii* Ishizaki; Nakao et al., p. 133, fig. 5.9^258^.

v. 2001 *Xestoleberis hanaii* Ishizaki; Vannier et al., p. 84, figs. 8.4, 8.5^320^.

v. 2002 *Xestoleberis hanaii* Ishizaki; Irizuki et al., p. 39, pl. 1, fig. 21^152^.

v. 2003 *Xestoleberis hanaii* Ishizaki; Irizuki et al., p. 13, pl. 1, fig. 8^308^.

v. 2003 *Xestoleberis hanaii* Ishizaki; Yamaguchi, p. 135, fig. 1M^203^.

non. 2004 *Xestoleberis hanaii* Ishizaki; Irizuki, p. 77, pl. 3, fig. 7^37^.

2004 *Xestoleberis hanaii* Ishizaki; Yamauchi, p. 74^38^.

v. 2005 *Xestoleberis hanaii* Ishizaki; Ishida and Takayasu, p. 77, fig. 2.13^216^.

v. 2005 *Xestoleberis hanaii* Ishizaki; Kondo et al., p. 6-11, 13, figs. 2-8, tables 1, 2, Appendix A^321^.

v. 2005 *Xestoleberis hanaii* Ishizaki; Tanaka, p. 125, fig. 2I^259^.

2006 *Xestoleberis hanaii* Ishizaki; Schornikov, p. 48^16^.

2006 *Xestoleberis hanaii* Ishizaki; Schornikov and Zanina, p.218, table 2^15^.

2007 *Xestoleberis hanaii* Ishizaki; Schornikov and Zanina, p.222^283^.

2007 *Xestoleberis hanaii* Ishizaki; Tanaka, p.222^194^.

2007 *Xestoleberis hanaii* Ishizaki; Yamada, p. 52, figs. 5R, 5S, 7A^231^.

v.p. 2008 *Xestoleberis hanaii* Ishizaki; Hu and Tao, p. 495, pl. 123, fig. 13^17^.

non. 2008 *Xestoleberis hanaii* Ishizaki; Hu and Tao, pl. 132, fig. 13, pl. 152, fig. 11, pl. 155, figs. 15, 17, text-fig. 285^17^.

2008 *Xestoleberis hanaii* Ishizaki; Hu and Tao, pl. 192, figs. 4, 9^17^.

non. 2008 *Xestoleberis hanaii* Ishizaki; Ozawa and Tsukawaki, p. 47, pl. 2, fig. 18^322^.

non. 2008 *Xestoleberis hanaii* Ishizaki; Yamada, p. 42, fig. 1D^233^.

v. 2008 *Xestoleberis hanaii* Ishizaki; Yamada, p. 42, fig. 2^233^.

v. 2009 *Xestoleberis hanaii* Ishizaki; Ozawa, p. 238, fig. 4.21^218^.

non. 2009 *Xestoleberis hanaii* Ishizaki; Tanaka and Nomura, p. 62, fig. 4.27^196^.

v. 2010 *Xestoleberis hanaii* Ishizaki; Kaji and Tsukagoshi, p. 526, 527, figs. 4, 6^323^.

non. 2010 *Xestoleberis hanaii* Ishizaki; Ozawa, p. 35, pl. 4, fig. 21^208^.

v. 2010 *Xestoleberis hanaii* Ishizaki; Ozawa and Domitsu, p. 6, fig. 5.19^155^.

non. 2011 *Xestoleberis hanaii* Ishizaki; Kawano et al., p. 5, fig. 3.15^198^.

non. 2012 *Xestoleberis hanaii* Ishizaki; Tanaka et al., p. 14, pl. 2, fig. 18^40^.

v. 2012 *Xestoleberis hanaii* Ishizaki; Tanaka et al., p. 118, pl. 2, fig. 1^199^.

v. 2013 *Xestoleberis hanaii* Ishizaki; Kawano et al., p. 96, fig. 5.15^200^.

v. 2014 *Xestoleberis hanaii* Ishizaki; Noraswana and Ramlan, p. 614, fig. 4^324^.

v. 2014 *Xestoleberis hanaii* Ishizaki; Masuma and Yamada, p. 6, fig. 4.23^41^.

v. 2017 *Xestoleberis hanaii* Ishizaki; Tsukagoshi, p. 21, fig. 12^201^.

v. 2017 *Xestoleberis hanaii* Ishizaki; Yamada, p. 1571-1575, figs. 1-6^325^.

v. 2018 *Xestoleberis hanaii* Ishizaki; Faiz and Raoh, p. 117, fig. 3.18^326^.

non. 2018 *Xestoleberis hanaii* Ishizaki; Irizuki et al., p. 47, fig. 5^247^.

v. 2018 *Xestoleberis hanaii* Ishizaki; Yamada et al., p. 337, fig. 5.11^166^.

v. 2019 *Xestoleberis hanaii* Ishizaki; Irizuki et al., p. 227, fig. 5.11^168^.

v. 2019 *Xestoleberis* sp. 14 Le and Tsukagoshi, p. 19, fig. 4. 24^202^.

v. 2019 *Xestoleberis hanaii* Ishizaki; Niiyama et al., p. 105, fig. 4.14^133^.

v. 2019 *Xestoleberis hanaii* Ishizaki; Tanaka et al., p. 34, fig. 4v^248^.

v. 2019 *Xestoleberis hanaii* Ishizaki; Tanaka et al., p. 13, figs. 5.3-5, 5.10^203^.

v. 2020 *Xestoleberis hanaii* Ishizaki; Sasaki et al., p. 8, fig. 4.12^249^.

v. 2022 *Xestoleberis hanaii* Ishizaki; Kaneko et al., p. 65, pl. 3, fig. 13^220^.

**Type:** Left valve (Holotype), IGPS 90316.

**Remarks:** Ishizaki^1^ illustrated the external left lateral view of the holotype with an optical photograph (pl. 9, fig. 2) and an optical photograph of the external view of the right valve (IGPS 90317, paratype, pl. 9, fig. 1) The current reexamination presents the external view of the holotype. Schornikov^311^ described the soft parts of this species from the Kuril Islands. Okubo^313^ illustrated several ontogenetic stages of this species and noted seasonal variations in valve sizes: smaller in summer and larger in spring. He observed this species in the bottom and algae of rocky shores, where it is abundant in summer, and grows and dies within one or two months. Okubo^313^ also described the copulatory postures of males and females and noted that the A-2 instar develops in the female carapace. Kamiya^180^ documented this species living on eelgrass and the bottom sediment near the eelgrass. Kamiya^182^ reported its presence on the leaves of *Nemacystus*, *Grateloupia*, and calcareous algae. Tsukagoshi and Parker^294^ provided an SEM image of the trunk region of this species. According to Ikeya and Kato^319^, females lay about 40 eggs and live for approximately 18 weeks. Yamaguchi^203^ analyzed 18S rDNA of this species from Tsukumo Bay, Southwest Honshu, Japan. Kondo et al.^321^ cultured this species at various water temperatures and measured Mg/Ca ratios at different molt stages. Kaji and Tsukagoshi^323^ illustrated ontogenetic changes in the eye, showing that the eye contains pigment and tapetum during the embryo stage. Vannier et al.^320^ provided a thin cross-section of the body of this species. Tanaka^259^ illustrated thin sections of the lateral and median eyes. Yamada^325^ examined intermolt and molt stages of the “Xestoleberis-spot” using SEM and TEM, concluding that this feature helps provide chitin fibers to the exocuticle of the outer lamella. Whatley and Zhao^315^ identified this species from Brady’s specimen, but it differs by having a pointed dorsal margin at mid-length from the anterior end, a broadly arched posterior margin, and a rounded lateral outline. Yumoto^318^ illustrated this species, but it differs from the type specimen by having a widely arched posterior margin, a more posteriorly positioned highest point, and a more sinuate ventral margin. *Xestoleberis hanaii*, as figured by Mostafawi^74^ from the Sunda Shelf, does not match the type specimen, differing in having a higher carapace in lateral view, an acutely arched anterior margin, and a larger posterior cardinal angle. Some specimens illustrated by Hu and Tao^17^ from Taiwan do not belong to this species, as they exhibit a straight dorsal margin of the left valve, a broadly arched posterior margin, and a straight ventral margin (pl. 132, fig. 13, pl. 152, fig. 11, pl. 155, figs. 15, 17). Ozawa and Tsukawaki^322^ depicted a right valve of *X. hanaii*, but it differs by having an acute anterior margin, a postero-dorsally arched posterior margin, and a slender lateral outline. Tanaka and Nomura^196^ illustrated this species from the Late Miocene Maja Formation, Kumejima Island, Okinawa, Japan; however, it differs by having a rounded dorsal margin, a postero-ventrally arched posterior margin, and a weakly sinuate ventral margin. Ozawa^304^ illustrated a juvenile specimen of *X. hanaii* that does not match the juveniles of *X. hanaii* shown by Okubo^313^, as it features a straight ventral margin, a dorsally arched posterior margin, and an acutely arched anterior margin. Wang et al.^276^ illustrated *X. hanaii*, but their specimen differs with a broadly arched anterior margin, the highest point located at mid-length from the anterior end, and a relatively straight ventral margin. Yamada^233^ illustrated various specimens as this species; however, one figure (fig. 1D) does not represent *X. hanaii* due to its widely arched dorsal margin, postero-ventrally arched posterior margin, and straight ventral margin. Irizuki^37^ reported this species from the Early Pleistocene Masuda Formation on Tasnegashima Island, Japan; however, it differs by having the highest point at mid-length from the anterior end, a more broadly arched anterior margin, and evenly curved anterior and posterior dorsal margins. Kawano et al.^198^ illustrated a juvenile specimen of this species; however, it differs with a shorter antero-dorsal margin, a ventrally projected postero-ventral area, and a relatively straight ventral margin. Tanaka et al.^40^ illustrated this species from recent sediment in Suruga Bay, Central Japan; however, it differs by having a widely arched anterior margin, a straight ventral margin, and a postero-ventrally projected posterior margin. Irizuki et al.^247^ reported this species from recent sediment on Tsushima Island, Southwest Japan; however, it differs by having an acute dorsal margin, a broadly arched anterior margin, and a rounded posterior margin. *Xestoleberis* sp. 14, illustrated by Le and Tsukagoshi^202^ from Southern Vietnam, is identified as *X. hanaii* based on its outline.

**Occurrence:**

*Recent*: Palau Bidong; off the coast of the Malay Peninsula; East coast of the Malay Peninsula; Co To Islands, Northern Vietnam; South China Sea; the Northern part of the South China Sea, China; Seto Inland Sea, Shikoku; Sea of Japan side Southwest and Northern Honshu, Japan; Central Honshu, Sendai Bay, Japan; Kuril Islands.

*Fossil*: Pleistocene Cheju Island, Korea; Early Pleistocene Sado Island, Central Japan; Middle Pleistocene Central and North Honshu, Japan; Late Pleistocene in Southern Taiwan; Late Pleistocene to Holocene in the Okinawa Trough, Japan; Holocene borehole cores from Hong Kong, China; Holocene in the Tsushima Straits, Kyushu, Southwest Honshu, Japan; Holocene from the Sea of Japan side of Southwest Honshu, Central, and the Pacific side of Northeast Japan.

Family: Krithidae Mandelstam, 1958^327^

Genus: *Krithe* Brady, Crosskey and Robertson, 1874^328^

Type Species: *Ilyobates praetexta* Sars, 1866^5^

28. *Krithe japonica* Ishizaki, 1971^3^

v*. 1971 *Krithe japonica* Ishizaki, p. 78, pl. 5, fig. 1, pl. 6, figs. 1, 5, pl. 7, fig. 6^3^.

v. 1975 *Krithe japonica* Ishizaki; Ishizaki, p. 56. ^241^

v. 1977 *Krithe japonica* Ishizaki; Hanai et al., p. 26^8^.

v. 1984 *Krithe japonica* Ishizaki; Ishizaki, p. 43, pl. 2, fig. 7^222^.

v. 1990 *Krithe japonica* Ishizaki; Lee, p. 163-164, pl. 7, figs. 1, 2, text-fig. 31^29^.

v. 1991 *Krithe japonica* Ishizaki; Ikeya and Itoh, p. 138, fig. 17C^149^.

non. 1992 *Krithe japonica* Ishizaki; Huh and Paik, p.113, pl. 3, fig. 6^214^.

v. 1992 *Krithe japonica* Ishizaki; Lee and Paik, p. 147, pl. 1, fig. 16^31^.

non. 1997 *Krithe japonica* Ishizaki; Zhao and Whatley, p. 202, fig. 5.10^329^.

v. 2001 *Krithe japonica* Ishizaki; Yasuhara and Irizuki, p. 83, pl. 6, figs. 1-4^151^.

2004 *Krithe japonica* Ishizaki; Irizuki, p. 73, pl. 1, fig. 7^37^.

v. 2004 *Krithe japonica* Ishizaki; Yasuhara et al., p. 32, fig. 14q, p. 33, fig. 15q^230^.

v. 2006 *Krithe japonica* Ishizaki; Yasuhara and Seto, p. 106, fig. 4h^243^.

v. 2009 *Krithe japonica* Ishizaki; Ozawa, p. 239, 240, figs. 4.2, 4.3^218^.

v. 2009 *Krithe japonica* Ishizaki; Irizuki et al., p. 16, fig. 4.1^163^.

v. 2011 *Krithe japonica* Ishizaki; Irizuki et al., p. 2035, fig. 5^264^.

v. 2011 *Krithe japonica* Ishizaki; Irizuki et al., p. 42, fig. 4.1^219^.

v. 2013 *Krithe japonica* Ishizaki; Kawano et al., p. 96, fig. 5.6^200^.

v. 2015 *Krithe japonica* Ishizaki; Irizuki et al., p. 153, fig. 4.1^245^.

v. 2018 *Krithe japonica* Ishizaki; Irizuki et al., p. 643, fig. 5.7^157^.

v. 2018 *Krithe japonica* Ishizaki; Irizuki et al., p. 48, fig. 6^247^.

v. 2021 *Krithe japonica* Ishizaki; Irizuki et al., p. 7, fig. 6.1^250^.

**Type:** Female right valve (Holotype), IGPS 90342.

**Remarks:** Ishizaki^1^ illustrated the holotype, with an SEM image in internal view of the left valve (pl. 6, fig. 1), along with the internal view of the female right valve (IGPS 90343, paratype, pl. 6, fig. 5). He also provided SEM images of the internal view of the male left valve (IGPS 90341, paratype, pl. 5, fig. 1) and the external view of the male right valve (IGPS 90340, paratype, pl. 7, fig. 6). The present reexamination focuses on the external view of the holotype. *Krithe japonica* reported from the Middle Miocene of the lower unit of the Yeonil Group, South Korea, differs from the type species by having a narrowly arched anterior margin and posterior margin, a straight ventral margin, and the maximum height located at mid-length from the anterior end. Zhao and Whatley^329^ illustrated this species from a depth of 1,491 m in the Okinawa Trough; however, it differs from the type specimen in having a widely arched dorsal margin, a convex ventral margin, and a prominent projection of the caudal process.

**Occurrence:**

*Recent*: Tsushima Island; Seto Inland Sea, Japan; Sendai Bay, Northern Honshu, Japan.

*Fossil*: Middle to Late Pleistocene on Cheju Island, Korea; Middle Pleistocene in Central Honshu, Japan; Holocene in the Tsushima Straits, Southwest Honshu, and the Seto Inland Sea, Japan.

Family: Cytherideidae Sars, 1925^330^

Genus: *Perissocytheridea* Stephenson, 1938^331^

Type Species: *Cytheridea* ? *matsoni* Stephenson, 1935^332^

**29.** *Perissocytheridea japonica* Ishizaki, 1968^1^

v*. 1968 *Perissocytheridea japonica* Ishizaki, p. 18, pl. 1, fig. 4, pl. 3, figs. 4, 5^1^.

v. 1968 *Clithrocytheridea* sp. A Ishizaki, p. 18, pl. 3, fig. 12^1^.

v. 1977 *Clithrocytheridea* ? *japonica* (Ishizaki); Hanai et al., p. 24^8^.

non. 1979 *Clithrocytheridea* ? *japonica* (Ishizaki); Okubo, p. 143, fig. 1a-d^333^.

1980 *Clithrocytheridea* ? *japonica* (Ishizaki); Okubo, p. 394^173^.

v. 1982 *Perissocytheridea japonica* Ishizaki; Hou et al., p. 162-163, pl. 73, figs. 9-13, pl. 71, figs. 19, 20, text-fig. 25a, 25b^20^.

v.1983 *Perissocytheridea japonica* Ishizaki; Okubo, p. 403-410, figs. 1-3^334^.

v.1985 *Perissocytheridea japonica* Ishizaki; Zhao, pl. 2, fig. 6^160^.

non.1988 *Perissocytheridea japonica* Ishizaki; Wang, et al., p. 103, pl. 1, fig. 5^276^.

v.1989 *Perissocytheridea japonica* Ishizaki; Ruan, pl. 1, fig. 3^161^.

non.1991 *Perissocytheridea japonica* Ishizaki; Ikeya and Itoh, p. 141, fig. 20D^149^.

v.1993 *Perissocytheridea (Kroemmelbeinella) japonica* Ishizaki; Witte, p. 16, text-fig. 2^76^.

non. 2003 *Perissocytheridea japonica* Ishizaki; Yamaguchi, p. 135, fig. 1U^203^.

2007 *Perissocytheridea japonica* Ishizaki; Yamada, p. 52, figs.7F. 8F^231^.

v. 2008 *Perissocytheridea japonica* Ishizaki; Nakao and Tsukagoshi, p. 269, pl. 1, figs. H-K^234^.

non. 2012 *Perissocytheridea japonica* Ishizaki; Tanaka et al., p. 118, pl. 2, fig. 7^199^.

non. 2014 *Perissocytheridea japonica* Ishizaki; Masuma and Yamada, p. 6, fig. 4.11^41^.

v. 2016 *Perissocytheridea japonica* Ishizaki; Matsushima et al., p. 14, fig. 6G^238^.

**Type:** Detached carapace (Holotype), IGPS 90204.

**Remarks:** Ishizaki^1^ illustrated the line drawing of the internal view of a right valve (pl. 1, fig. 4) and the external left and right lateral views of the holotype (pl. 3, figs. 4, 5), along with an optical photograph of the external view of a right valve (IGPS 90317, paratype, pl. 9, fig. 1) The present reexamination shows the external left and right views and the internal view of the right valve of the holotype. Okubo^334^ provided illustrations of appendages and SEM images of males, females, and juveniles. Based on his SEM images, the type specimen is identified as female. Okubo^334^ also noted that his previously identified *Clithrocytheridea*? *japonica* does not morphologically coincide with the type specimen and proposed *Perissocytheridea inabai* in the same literature. Wang et al.^276^ illustrated this species from borehole cores collected from the Bohai Sea; however, their specimen differs in having an acute anterior margin, strong carinal ridges running parallel to the ventral and dorsal margins, and a dorsally directed caudal process. Ikeya and Itoh^149^ found this species in Sendai Bay, Japan, but it differs from the type specimen with a sinuate ventral margin, a prominent bulb at the postero-ventral area, and a short caudal process. Yamaguchi^203^, Tanaka et al.^199^, and Masuma & Yamada^41^ identified this species from Northern and Central Honshu, Japan, respectively, but their specimens are not *P. japonica* but *P. inabai*.

**Occurrence:**

*Recent*: Coast near Shanghai, China; coast of Hebei, Yellow Sea, China; Shikoku, Japan; Central Honshu, Japan.

*Fossil*: Quaternary in Jiangsu, China; Holocene core from Central Japan.

Family: Hemicytheridae Puri, 1953^335^

Genus: *Aurila* Pokorny, 1955^336^

Type Species: *Cythere convexa* Baird, 1850^337^

**30.** *Aurila cymba* (Brady, 1969)^211^

v* 1869 *Cythere cymba* Brady, p. 157, pl. 16, figs. 1-4^211^.

v. 1880 *Cythere cymba* Brady; Brady, p. 80-81, pl. 20, fig. 5a-f^268^.

v. 1959 *Cythere cymba* Brady; Hanai, p. 428^170^.

v. 1968 *Aurila miii* Ishizaki, p. 22, pl. 1, fig. 9, pl. 4, figs. 1, 2^1^.

v. 1969 *Aurila miii* Ishizaki; Ishizaki, p. 217, pl. 25, fig. 13^2^.

v. 1971 *Aurila miii* Ishizaki; Ishizaki, p. 81, 82, pl. 2, fig. 1^3^.

v. 1976 *Cythere cymba* Brady; Puri and Hulings, pl. 25, figs. 3-6^269^.

v. 1977 *Aurila cymba* (Brady); Hanai et al., p. 42-43^8^.

non. 1980 *Aurila subconvexa* (Kajiyama); Okubo, p. 397-399, figs. 2, 7m, 7n, 9a-d^173^.

v. 1981 *Aurila miii* Ishizaki; Gou et al., p. 161-162, pl. 91, fig. 4^221^.

v. 1982 *Aurila miii* Ishizaki; Cai, p. 7, pl. 2, figs. 23, 24^21^.

v.1982 *Aurila miii* Ishizaki; Hou et al., p. 179-181, pl. 75, figs. 1-4, 5, 6, text-fig. 41^20^.

1982 *Aurila miii* Ishizaki; Hou et al., pl. 75, figs. 7-10, text-fig. 41^20^.

non 1983 *Aurila miii* Ishizaki; Gou et al., p.55-56, pl. 18, figs. 11-16^22^.

v. 1984 *Aurila cymba* (Brady); Ishizaki, p. 42, pl. 1, fig. 7^222^.

non. 1985 *Aurila miii* Ishizaki; Huang, p. 53, pl. 1, fig. 11^338^.

v. 1985 *Aurila cymba* (Brady); Ishizaki and Matoba, pl. 1, fig. 11^13^.

v. 1985 *Aurila cymba* (Brady); Zhao, pl. 2, fig. 4^160^.

v. 1986 *Aurila cymba* (Brady); Lin and Zhu, p. 97, pl. 2, figs. 4, 9^273^.

non. 1986 *Aurila miii* Ishizaki; Zheng, pl. 1, figs. 30, 31^274^.

non. 1987 *Aurila miii* Ishizaki; Zheng, p. 196, pl. 4, figs. 1-9^25^.

v. 1987 *Aurila cymba* (Brady); Wang and Zhang, pl. 2, fig. 9^146^.

v. 1987 *Aurila cymba* (Brady); Whatley and Zhao, p. 28, pl. 2, figs. 9-12^315^.

non. 1988 *Aurila subconvexa* (Kajiyama); Ikeya and Kashima, p. 202, fig. 2.1^275^.

non. 1988 *Aurila uranouchiensis* Ishizaki; Kamiya, p. 307, 308, text-figs. 4, 5.8^180^.

1988 *Aurila cymba* (Brady); Okubo, p. 139, text-fig. 3 (part)^339^.

non. 1988 *Aurila miii* Ishizaki; Ruan and Hao, p. 309, pl. 54, figs. 11-15^28^.

v. 1988 *Aurila cymba* (Brady); Wang, et al., p. 103, pl. 1, fig. 32^276^.

non.1989 *Aurila miii* Ishizaki; Ruan, pl. 1, fig. 14^161^.

non. 1990 *Aurila cymba* (Brady); Gou, p. 27, pl. 2, fig. 21^71^.

v. 1990 *Aurila cymba* (Brady); Lee, p. 225-227, pl. 15, figs. 1-3^29^.

v. 1990 *Aurila cymba* (Brady); Takayasu et al., pl. 1, fig. 9^212^.

non. 1990 *Aurila miii* Ishizaki; Ruan, p. 133, pl. 1, fig. 13^72^.

non. 1990 *Aurila cymba* (Brady); Zhao and Wang, pl. 1, fig. 19^148^.

non. 1992 *Aurila cymba* (Brady); Lee and Paik, p. 151, pl. 3, fig. 4^31^.

non. 1993 *Aurila cymba* (Brady); Ishizaki et al., p. 360, pl. 34, fig. 2^215^.

v. 1994 *Aurila cymba* (Brady); Zheng et al., pl. 45, figs. 7, 8^32^.

non. 1994 *Aurila miii* Ishizaki; Zheng et al., pl. 45, figs.11-13^32^.

v.p. 1998 *Aurila cymba* (Brady); Cao, pl. 1, figs. 16, 18, 21^34^.

non. 1998 *Aurila cymba* (Brady); Cao, pl. 1, figs. 14, 15, 17, 19^34^.

v. 1998 *Aurila cymba* (Brady); Cao, pl. 1, fig. 20^34^.

v. 1998 *Aurila cymba* (Brady); Irizuki et al., p. 7, fig. 2.9^150^.

non. 1998 *Aurila cymba* (Brady); Tanaka et al., p. 91, pl. 1, fig. 13^257^.

v. 1998 *Aurila cymba* (Brady); Yamane, p. 37, pl. 1, figs. 5a-5e^35^.

v. 2000 *Aurila cymba* (Brady); Irizuki and Hosoyama, p. 12, fig. 3.8^226^.

v. 2001 *Aurila cymba* (Brady); Yasuhara and Irizuki, p. 73, pl. 1, figs. 10, 11^151^.

v. 2002 *Aurila cymba* (Brady); Irizuki et al., p. 39, pl. 1, figs. 7, 8^152^.

v. 2003 *Aurila cymba* (Brady); Irizuki et al., p. 13, pl. 1, fig. 3^308^.

2004 *Aurila miii* Ishizaki; Yamauchi, p. 72^38^.

v. 2005 *Aurila cymba* (Brady); Irizuki et al., p. 41, fig. 3.14^162^.

v. 2005 *Aurila cymba* (Brady); Ishida and Takayasu, p. 76, fig. 2.1^216^.

v. 2006 *Aurila cymba* (Brady); Irizuki et al., p. 23, fig. 6.7^39^.

v. 2008 *Aurila miii* Ishizaki; Hu and Tao, pl. 210, fig. 3, pl. 210, figs. 3, 4, pl. 217, fig. 19^17^.

2008 *Aurila miii* Ishizaki; Hu and Tao, p. 205, pl. 2, fig. 16, pl. 217, fig. 17, pl. 218, figs. 1, 4^17^.

non. *Aurila miii* Ishizaki; Hu and Tao, pl. 43, fig. 24, pl. 65, figs. 6, 8, 10-12, pl. 175, figs. 21, 22, pl. 209, fig. 18, pl. 221, figs. 14, 17, 22, pl. 222, figs. 17, 21, text-fig. 122^17^.

v. 2008 *Aurila cymba* (Brady); Irizuki et al., p. 295, fig. 7^244^.

v. 2009 *Aurila cymba* (Brady); Horikoshi et al., p. 92, fig. 5.2^261^.

v. 2011 *Aurila cymba* (Brady); Kawano et al., p. 5, fig. 3.1^198^.

v. 2012 *Aurila cymba* (Brady); Tanaka et al., p. 118, pl. 2, fig. 13^199^.

v. 2015 *Aurila cymba* (Brady); Irizuki et al., p. 153, fig. 4.7^246^.

v. 2019 *Aurila cymba* (Brady); Niiyama et al., p. 104, fig. 3.16^133^.

v. 2019 *Aurila cymba* (Brady); Tanaka et al., p. 34, fig. 4b^248^.

v. 2019 *Aurila cymba* (Brady); Tanaka et al., p. 9, fig. 1.1-4^203^.

v. 2021 *Aurila cymba* (Brady); Maehama et al., p. 371, fig. 6.7^266^.

v. 2021 *Aurila cymba* (Brady); Yamada et al., p. 582, fig. 7.3^340^.

**Type:** Left valve, IGPS 90231.

**Remarks:** Ishizaki^1^ illustrated the internal view (pl. 1, fig. 9) and the external views of the holotype in optical photographs (pl. 4, fig. 2). He also illustrated an optical photograph of the external view of the right valve (IGPS 90232, paratype, pl. 4, fig. 1). Hanai et al.^8^ classified *Aurila miii* as a junior synonym of *Aurila cymba* (Brady, 1869) ^211^. The SEM images of *Aurila subconvexa* (Kajiyama) by Okubo^173^ are identified as *A. cymba* in this study. Okubo^173^ also described the soft parts of this species. Subsequently, Whatley and Zhao^315^ researched Brady’s (1869) ^211^ type specimens and assigned the lectotype and paralectotype of *A. cymba*. From the comparison of the SEM image of *A. cymba* by Whatley and Zhao^315^ and the holotype specimen of *A. miii*, the latter is confirmed as a junior synonym of *A. cymba*. Hou et al.^20^ illustrated adult and juvenile specimens of this species; however, at least two the figured specimens included *Aurila hataii*, based on six carinal ridges radiated from the central area. *Aurila miii* reported by Gou et al.^22^ from the Pliocene strata of the Leizhou Peninsula, South China, is different from the type species in that it has a longer ridge starting from the antero-ventral area to below the posterior cardinal angle via the eye tubercle, lacks developed two reticulations near the antero-ventral margin and has a short ridge on the caudal process. Kamiya^180^ reported that this species lives on the bottom near *Zostera* beds, although he identified it as *Aurila uranouchiensis*. Okubo^339^ illustrated the male copulatory organ of *A*. *cymba*. However, there is no shell information, so identification was not possible. Ruan and Hao^28^ identified this species from the Late Pleistocene to Holocene cores from Okinawa Tough, Japan; however, their specimen differs in having a widely arched anterior margin, a more anteriorly shifted highest position, and a prominent postero-ventral tubercle. Ruan^161^ illustrated this species from the coast of Hubei, China; however, their specimen differs in having three carinal ridges in the postero-dorsal area, a short carinal ridge in the central area, and an undulated long postero-central carinal ridge. *Aurila cymba*, as figured by Gou^71^ from Hainan Island, South China, is actually *Aurila hataii* based on the carinal ridges radiating from the central area. Zhao and Wang^148^ reported this species from the Northern China shelf; however, their specimen differs by having dorsally arched carinal ridges, short posterior carinal ridges, and finer reticulation. Lee and Paik^31^ illustrated this species from the Pleistocene sediments of Cheju Island, Korea; however, it differs with a straight carinal ridge running from the antero-ventral margin to the central area, a short postero-dorsal carinal ridge, and a longer anterior cardinal ridge. The specimen figured by Ishizaki et al.^215^ from the Pleistocene Omma Formation, Central Japan, is not *A. cymba*, as it has a straight carinal ridge posterior to the eye tubercle, a postero-ventrally protruded ala-like structure, and an undulate mid-posterior carinal ridge. Cao^34^ figured several specimens from the Quaternary borehole cores in Hong Kong; however, some differ from the type specimen and are identified as *A. hataii* due to the radially arranged carinal ridges. Tanaka et al. ^257^ found this species in Lake Nakaumi, Shimane, Southwest Japan; however, it is *A. munechikai* based on the lateral outline and ornamentation. Many specimens shown by Hu and Tao^17^ as *A*. *miii* Ishizaki are inconsistent with Ishizaki’s specimen, having finer ornamentation, a longer antero-ventral carina, and a prominent postero-dorsal tubercle.

**Occurrence:**

*Recent*: Co To Islands, Northern Vietnam; South China; Southern Taiwan; South China Sea; Hong Kong, coast near the Yellow Sea, Bohai Sea, China; shelf from south to middle China; Kyushu, Shikoku, Japan; Seto Inland Sea, Central and Northern Honshu, Sea of Japan side of Southwest Japan.

*Fossil*: Pliocene borehole cores from the South China Sea; Middle Pleistocene Kyushu, Central to Northern Honshu, Japan; Late Pleistocene Southern Taiwan; Pleistocene Cheju Island, Korea; Quaternary borehole cores from Hong Kong, Bohai Sea, China; Quaternary Jiangsu, Fujian, Zhejiang, China; Holocene borehole cores from Hong Kong and Bohai Sea, China; Holocene Southwest Honshu, Central Honshu, and Seto Inland Sea, Japan.

**31.** *Aurila hataii* Ishizaki, 1968^1^

v*. 1968 *Aurila hataii* Ishizaki, p. 20-21, pl. 1, figs. 5, 6, pl. 4, figs. 5, 6^1^.

v. 1971 *Aurila* cf *hataii* Ishizaki; Ishizaki, p. 81, pl. 2, figs. 2, 3^3^.

v. 1977 *Aurila hataii* Ishizaki; Hanai et al., p. 43^8^.

v. 1977 *Aurila hataii* Ishizaki; Kamiya and Nakagawa, p. 127, pl. 3, fig. 5^14^.

v. 1980 *Aurila hataii* Ishizaki; Okubo, p. 400-401, figs. 3, 7g-j, 8, 10a-f^173^.

v. 1982 *Aurils munechiiensis* Ishizaki; Cai, p. 7, pl. 2, figs. 19, 20^21^.

v.1982 *Aurila miii* Ishizaki; Hou et al., pl. 75, figs. 5, 6^20^.

v. 1985 *Aurila hataii* Ishizaki; Ikeya et al., pl. 4, figs. 1, 2^12^.

v. 1987 *Aurila uranouchiensis* Ishizaki; Wang and Zhang, p.291, pl. 2, fig. 8^146^.

v. 1988 *Aurila ikeyai* Okubo, Ikeya and Shiozaki, p. 137, pl. 3, figs. 9a, 9b^27^.

v. 1988 *Aurila okayamensis* Okubo, p. 142^339^.

non. 1988 *Aurila hataii* Ishizaki; Okubo, text-figs. 3 (part), 5c, 5d^339^.

v. 1988 *Aurila ikeyai* Okubo, p. 142, text-figs. 1d-i, 5e, 5f^339^.

v. 1990 *Aurila cymba* (Brady); Gou, p. 27, pl. 2, fig. 21^71^.

v. 1990 *Aurila hataii* Ishizaki; Lee, p. 228-229, pl. 13, figs. 7, 10, 11^29^.

v. 1990 *Aurila miii* Ishizaki; Ruan, p. 133, pl. 1, fig. 13^72^.

v. 1991 *Aurila hataii* Ishizaki; Ikeya and Itoh, p. 131, fig. 10D^149^.

v. 1992 *Aurila hataii* Ishizaki; Ikeya and Suzuki, p. 123, pl. 2, fig. 4^183^.

v. 1992 *Aurila hataii* Ishizaki; Lee and Paik, p. 151, pl. 3, fig. 10^31^.

v. 1994 *Aurila hataii* Ishizaki; Zheng et al., pl. 45, fig. 9, 10^32^.

v. 1998 *Aurila cymba* (Brady); Cao, pl. 1, figs. 14, 15, 17, 19^34^.

v. 2001 *Aurila hataii* Ishizaki; Kamiya et al., p.93, fig. 13.1^36^.

v. 2001 *Robustaurila ishizakii* (Okubo); Nakao et al., p. 133, fig. 5.4^258^.

v. 2007 *Aurila hataii* Ishizaki; Tanaka, p. 125, fig. 2D^194^.

v.p. 2008 *Aurila hataii* Ishizaki; Hu and Tao, p. 211, pl. 65, figs. 2, 3, 7, pl. 113, fig. 17, pl. 128, figs. 4, 6, text-fig. 128^17^.

non. 2008 *Aurila hataii* Ishizaki; Hu and Tao, pl. 25, fig. 21, pl. 35, fig. 2, pl. 44, figs. 5-7, 10, pl. 65, fig. 4, pl. 113, figs. 14-16, 19, 20, pl. 128, figs. 2, 3, 5^17^.

non. 2008 *Aurila hataii* Ishizaki; Ozawa and Tsukawaki, p. 45, pl. 1, fig. 9^322^.

2009 *Aurila corniculata* Okubo; Irizuki et al., p. 320, fig. 3.6^288^.

v. 2009 *Aurila hataii* Ishizaki; Irizuki et al., p. 320, fig. 3.7^288^.

v. 2009 *Aurila hataii* Ishizaki; Tanaka and Nomura, p. 61, fig, 3.6^196^.

non. 2010 *Aurila hataii* Ishizaki; Ozawa and Domitsu, p. 4, fig. 3.6^155^.

2012 *Aurila hataii* Ishizaki; Tanaka et al., p. 19^40^.

v. 2019 *Aurila hataii* Ishizaki; Niiyama et al., p. 104, fig. 3.17^133^.

v. 2019 *Aurila hataii* (Brady); Tanaka et al., p. 9, fig. 1.5-8^203^.

non. 2024 *Aurila cymba* (Brady); Maekawa, fig. 182.68^285^.

v. 2024 *Aurila kiritsubo* Yajima; Maekawa, fig. 182.69^285^.

**Type:** Detached carapace (Holotype), IGPS 90299.

**Remarks:** Ishizaki^1^ illustrated the line drawings of the internal view of the right and left valves (pl. 1, figs. 5, 6) along with the external left and right lateral views (pl. 4, figs. 5, 6) of the holotype. Okubo^173^ described the soft parts and illustrated the male copulatory organ of this species. Additionally, he provided SEM images of the male and female and the A-1 stage of this species. Okubo^173^ redescribed his specimen as *Aurila okayamensis*, highlighting differences in the morphology of the male copulatory organ, which had been misidentified as *A. hataii* in Okubo^339^. The SEM images and line drawings of the male copulatory organ provided by Okubo^339^ do not match *A. hataii*, as they lack the two prominent carinal ridges extending from the central area to the postero-dorsal and postero-ventral areas, concentric reticulation, and a ventral carinal ridge. *Aurila ikeyai* Okubo, 1988^339^ is identified as *A. hataii* based on the five carinal ridges radiating from the central area and shell outline. The present reexamination confirms the external left and right views and the internal view of the right valve of the holotype. Several specimens figured by Hu and Tao^17^ are not this species, as they exhibit an overlapped ventral carina at the postero-ventral area, irregular reticulation of the posterior area, and lack of mid-posterior and postero-ventral carinas. The specimen identified as *Aurila corniculata* (Okubo) by Irizuki et al.^288^ is a juvenile specimen of *A. hataii* with the same ridge pattern. Ozawa and Tsukawaki^322^ reported that the specimen found off Hokkaido is the male of *Aurila elongata* Schornikov and Tsareva (1995)^341^ from Moneron Island, Vostok, Russia. *Aurila hataii* identified by Ozawa and Domitsu^155^ is the female of *Aurila elongata* Schornikov and Tsareva (1995)^341^.

**Occurrence:**

*Recent*: Co To Islands, Northern Vietnam; Hainan Island, South China; South China Sea; coastal zone of Guangxi, China; Shikoku, Seto Inland Sea; Sea of Japan side, Southwest Honshu, Japan; central and Northern Honshu, Kunashiri Island, Japan.

*Fossil*: Pliocene of Taiwan; Late Pliocene, Okinawa, Japan; Pleistocene, Taiwan; Early Pleistocene, Central Honshu, Japan; Middle Pleistocene, Kyushu, Japan; Quaternary Hong Kong, China; Middle to Late Pleistocene, Cheju Island, Korea; Late Pleistocene, Southwest and Central Honshu, Japan; Quaternary Jiangsu, China; Holocene borehole cores from Hong Kong, China; Holocene, Southwest Honshu, Japan.

**32.** *Aurila imotoi* Ishizaki, 1968^1^

v*. 1968 *Aurila imotoi* Ishizaki, p. 21-22, pl. 1, figs. 7, 8, pl. 4, figs. 3, 4^1^.

v. 1977 *Aurila imotoi* Ishizaki; Hanai et al., p. 43^8^.

1982 *Aurila imotoi* Ishizaki; Hou et al., p. 181, pl. 75, figs. 13, 14^20^.

non. 1988 *Aurila imotoi* Ishizaki; Ruan and Hao, p. 309, pl. 54, figs. 16, 17^28^.

v. 1992 *Aurila imotoi* Ishizaki; Lee and Paik, p. 151, pl. 3, fig. 10^31^.

v. 2005 *Aurila imotoi* Ishizaki; Tanaka, p. 237, 238, figs. 2a, 3g, 3h, 3i^259^.

v.p. 2008 *Aurila imotoi* Ishizaki; Hu and Tao, pl. 128, fig. 8^17^.

non. 2008 *Aurila imotoi* Ishizaki; Hu and Tao, p. 207-208, pl. 65, fig. 15, text-fig. 125, pl. 113, figs. 10, 11, pl. 196, fig. 23, pl. 221, figs. 8, 23, text-fig. 125^17^.

v. 2008 *Aurila uranouchiensis* Ishizaki; Hu and Tao, pl. 185, figs. 10, 11, 13-16^17^.

v. 2017 *Aurila* sp.; Hong et al., p. 58, fig. 8.3^125^.

non. 2019 *Aurila corniculata* Okubo; Irizuki et al., p. 267, fig. 5.1^168^.

**Type:** Detached carapace (Holotype), IGPS 90230.

**Remarks:** Ishizaki^1^ illustrated the internal view of the left and right valves (pl. 1, figs. 7, 8) and the external right and left lateral views (pl. 4, figs. 3, 4) of the holotype. The present reexamination shows the external left and right views of the holotype. Tanaka^259^ illustrated the thin section of the lateral eye of this species. Ruan and Hao^28^ reported this species from Late Pleistocene cores from Okinawa Tough, Japan; however, their specimen differs by having a straight mid-posterior carinal ridge, a postero-dorsal inflation, and a v-shaped ventral carinal ridge. Many specimens figured by Hu and Tao^17^ are not included in this species because they have a prominent carina at the anterior area, two short antero-ventral carinas, and an angular postero-dorsal projection. Irizuki et al.^168^ identified *Aurila corniculata* from Holocene borehole cores from Matsushima Bay, Northeast Japan, based on surface ornamentation.

**Occurrence:**

*Recent*: Daxy Port, Northeastern Taiwan; Shikoku, Central Honshu, Japan.

*Fossil*: Pleistocene Southern Taiwan; Pleistocene Cheju Island, Korea; Holocene cores from Hong Kong, China; Matsushima Bay, Northeast Honshu, Japan.

**33.** *Aurila munechikai* Ishizaki, 1968^1^

v*. 1968 *Aurila munechikai* Ishizaki, p. 23, pl. 4, figs. 7, 8^1^.

v. 1977 *Aurila munechikai* Ishizaki; Hanai et al., p. 43^8^.

non. 1982 *Aurils munechiiensis* Ishizaki; Cai, p. 7, pl. 2, figs. 19, 20^21^.

v. 1988 *Aurila munechikai* Ishizaki; Kamiya, p. 307, 308, text-figs. 4, 7^180^.

v. 1989 *Aurila munechikai* Ishizaki; Kamiya, p. 78, 79, figs. 3, 4.7^182^.

1988 *Aurila munechikai* Ishizaki; Okubo, p. 139, text-fig. 3 (part)^339^.

v. 1990 *Aurila munechikai* Ishizaki; Lee, p. 230-321, pl. 14, figs. 10, 11^29^.

v. 1993 *Aurila munechikai* Ishizaki; Kamiya and Nakagawa, p. 127, pl. 3, fig. 7^14^.

v. 1998 *Aurila munechikai* Ishizaki; Irizuki et al., p. 7, fig. 2.10^150^.

non. 1998 *Aurila cymba* (Brady); Tanaka et al., p. 91, pl. 1, fig. 13^257^.

v. 2008 *Aurila munechikai* Ishizaki; Hu and Tao, p. 203-204, pl. 44, fig. 1, pl. 65, fig. 13, pl. 102, figs. 7, 17, 23, pl. 127, fig. 12, text-fig. 121^17^.

non. 2008 *Aurila munechikai* Ishizaki; Hu and Tao, pl. 25, fig. 16. pl. 44, figs. 2, 3, 6, pl. 65, fig. 14, pl. 102, fig. 16, 19, pl. 127, figs. 5, 6, 9, pl. 160, fig. 7, pl. 222, figs. 3, 11, 16^17^.

v. 2010 *Aurila munechikai* Ishizaki; Ozawa, p. 89, fig. 18.4^208^.

v. 2017 *Aurila munechikai* Ishizaki; Tsukagoshi, p. 20, fig. 7^201^.

**Type:** Detached carapace (Holotype), IGPS 90233.

**Remarks:** Ishizaki^1^ illustrated the holotype with external right and left lateral views from optical photographs (pl. 4, figs. 7, 8). The present reexamination displays the holotype. Kamiya^180^ showed that this species inhabits eelgrass beds. Okubo^339^ depicted the male copulatory organ of *A*. *munechikai*. However, without shell information, identification could not be confirmed. The specimens (pl. 25, fig. 19; pl. 44, figs. 2, 3, 6; pl. 127, figs. 5, 6, 9; pl. 160, fig. 7; pl. 222, figs. 3, 11, 16) illustrated by Hu and Tao^17^ do not represent *Aurila munechikai* Ishizaki, as they feature a broadly arched anterior margin, a sinuate postero-medial carina, and a postero-dorsal tubercle. Additionally, the specimen (pl. 102, fig. 19) shown by Hu and Tao^17^ is identified as a different species due to its lack of ornamentation, a postero-dorsal tubercle, and a triangular caudal process.

**Occurrence:**

*Recent*: South China Sea; Shikoku, the Sea of Japan side of Southwest Honshu, Japan; Central Honshu, Japan.

*Fossil*: Pliocene from Southern Taiwan; Upper Pliocene from Northeast Japan; Pleistocene from Southern Taiwan and Cheju Island, Korea; Late Pleistocene from Southern Taiwan; Holocene borehole core from Central Japan; Holocene from Southwest Honshu, Japan.

**34.** *Aurila tosaensis* Ishizaki, 1968^1^

v*. 1968 *Aurila tosaensis* Ishizaki, p. 23-24, pl. 4, figs. 16, 17^1^.

v. 1977 *Aurila tosaensis* Ishizaki; Hanai et al., p. 44^8^.

v. 1983 *Aurila uranouchiensis* Ishizaki; Gou et al., p. 54-55, pl. 18, figs. 1-10^22^.

v. 1985 *Aurila tosaensis* Ishizaki; Ikeya et al., pl. 4, fig. 3^12^.

v. 1990 *Aurila tosaensis* Ishizaki; Lee, p. 231-232, pl. 15, figs. 4-6^29^.

v. 1992 *Aurila tosaensis* Ishizaki; Lee and Paik, p. 151, pl. 3, fig. 11^31^.

v. 1992 *Aurila tosaensis* Ishizaki; Ikeya and Suzuki, p. 123, pl. 2, fig. 4^183^.

v. 1998 *Aurila tosaensis* Ishizaki; Yamane, p. 39, pl. 2, fig. 3^35^.

v. 2012 *Aurila tosaensis* Ishizaki; Tanaka et al., p. 19^40^.

v. 2019 *Aurila tosaensis* Ishizaki; Irizuki et al., p. 267, fig. 5.3^168^.

**Type:** Detached carapace (Holotype), IGPS 90234.

**Remarks:** Ishizaki^1^ illustrated the external left and right lateral views of the holotype in optical photographs (pl. 4, figs. 16, 17). The present reexamination confirms the validity of the species and its holotype.

**Occurrence:**

*Recent*: Shikoku, the Sea of Japan side of Southwest Honshu, and Central Honshu, Japan.

*Fossil*: Pliocene from the Leizhou Peninsula; Middle to late Pleistocene from Cheju Island, Korea; Holocene borehole cores from Matsushima Bay, Northeast Honshu, Japan.

**35.** *Aurila uranouchiensis* Ishizaki, 1968^1^

v*. 1968 *Aurila uranouchiensis* Ishizaki, p. 24, pl. 4, figs. 9, 10^1^.

v. 1971 *Aurila uranouchiensis* Ishizaki, p. 82, pl. 2, figs. 5, 11^3^.

v. 1977 *Aurila uranouchiensis* Ishizaki; Hanai et al., p. 44^8^.

non. 1982 *Aurila uranouchiensis* Ishizaki; Hou et al., p. 161, pl. 80, figs. 1, 2^20^.

1982 *Aurila uranouchiensis* Ishizaki; Cai, p. 7, pl. 2, figs. 16, 17^21^.

1982 *Aurila uranouchiensis* Ishizaki; Hou et al., p. 181-182, pl. 75, figs. 11, 12^20^.

non. 1983 *Aurila uranouchiensis* Ishizaki; Gou et al., p.54-55, pl. 18, figs. 1-10^22^.

non. 1987 *Aurila uranouchiensis* Ishizaki; Wang and Zhang, p.291, pl. 2, figs. 6-8^146^.

non. 1988 *Aurila uranouchiensis* Ishizaki; Cai, pl. 2, figs. 3, 4^26^.

non. 1988 *Aurila uranouchiensis* Ishizaki; Kamiya, p. 307, 308, text-figs. 4, 5.8^180^.

1988 *Aurila uranouchiensis* Ishizaki; Okubo, p. 139, text-fig. 3 (part)^339^.

1988 *Aurila uranouchiensis* Ishizaki; Ruan and Hao, p. 309-310, pl. 54, fig. 22^28^.

non. 1989 *Aurila uranouchiensis* Ishizaki; Kamiya, p. 78, 79, figs. 3, 4.8^182^.

non. 1992 *Aurila uranouchiensis* Ishizaki; Ikeya and Suzuki, p. 123, pl. 2, fig. 7^183^.

v. 2004 *Aurila uranouchiensis* Ishizaki; Irizuki, p. 73, pl. 1, fig.16^37^.

v. 2005 *Aurila uranouchiensis* Ishizaki; Irizuki et al., p. 41, fig.3.17^162^.

non. 2008 *Aurila uranouchiensis* Ishizaki; Hu and Tao, pl. 26, fig. 20, pl. 65, fig. 5, pl. 176, figs. 1, 4, pl. 185, figs. 10, 11, 13-16^17^.

v. 2017 *Aurila* sp. A. Tsukagoshi, p. 16, fig. 2M^201^.

non. 2017 *Aurila uranouchiensis* Ishizaki; Tsukagoshi, p. 20, fig. 8^201^.

**Type:** Left valve (Holotype), IGPS 90235.

**Remarks:** Ishizaki^1^ illustrated the external view (pl. 4, figs. 10) of the holotype in an optical photograph and provided an optical photograph of the external view of a right valve (IGPS 90236, paratype, pl. 4, fig. 9). The present reexamination confirms the holotype. *Aurila uranouchiensi*s, as reported by Hou et al.^20^ and Gou et al.^22^ from the Pliocene strata of the Leizhou Peninsula, South China, differs from the type species by having a robust external surface, an undulated postero-dorsal margin, and a narrowly arched anterior margin. Wang and Zhang^146^ described this species from Holocene borehole cores in Hong Kong, China, but it differs in having divided carinal ridges in the anterior area, coarser reticulation, and a prominent carinal ridge extending from the ventral side of the eye tubercle to the mid-anterior area. Okubo^339^ illustrated the male copulatory organ of *A*. *uranouchiensis*; however, without shell information, this could not be identified. Cai^26^ reported this species from the South China Sea shelf, but it differs by having prominent carinal ridges in the anterior area, the highest point located one-fourth from the anterior end, and a sinuate ventral margin. *Aurila uranouchiensis*, as identified by Kamiya^180^, is *Aurila cymba*, characterized by two anterior ridges, one posterior ridge extending from the mid-central area to the postero-ventral area, and a weak dorsal ridge running parallel to the dorsal margin. Ikeya and Suzuki^183^ illustrated this species off Shimane, Southwest Honshu, Japan, but it differs by having radiate carinal ridges from the central area, coarser reticulation, and a broadly arched anterior margin. Several figures (pl. 26, fig. 20; pl. 176, figs. 1, 4) by Hu and Tao^17^ do not depict *Aurila uranouchiensis*, in that it has two anterior carinas, a postero-dorsal tubercle, and a widely arched anterior margin. The specimen (pl. 65, fig. 5) by Hu and Tao^17^ from the Pliocene Maanshan Mudstone Formation in Southern Taiwan, does not represent *Aurila uranouchiensis*; it shows finer secondary reticulation in the anterior area, a long postero-ventral carina, and a more convex dorsal margin.

**Occurrence:**

*Recent*: Shikoku, Southwest and Northern Honshu, Japan.

*Fossil*: Early Pleistocene from Northern Ryukyu Island, Japan; Middle Pleistocene from Southwest Honshu, Japan.

Genus: *Psudoaurila* Ishizaki and Kato, 1976^342^

Type Species: *Pokornyella japonica* Ishizaki, 1968^1^

**36.** *Pseudoaurila japonica* (Ishizaki, 1968)^1^

v*. 1968 *Pokornyella japonica* Ishizaki, p. 25, pl. 1, fig. 10, pl. 5, figs. 5, 6^1^.

v. 1976 *Pseudoaurila japonica* (Ishizaki); Ishizaki and Kato, p.132-133, 137, pl. 2, figs. 2-5^342^.

v. 1977 *Pokornyella* ? *japonica* Ishizaki; Hanai et al., p. 45^8^.

v. 1990 *Pseudoaurila japonica* (Ishizaki); Lee, p. 255-256, pl. 18, figs. 2, 5, 6^29^.

v. 1992 *Pseudoaurila japonica* (Ishizaki); Ikeya and Suzuki, p. 135, pl. 8, fig. 3^183^.

v. 1993 *Pseudoaurila japonica* (Ishizaki); Kamiya and Nakagawa, p. 127, pl. 1, fig. 19^14^.

v. 2004 *Pseudoaurila japonica* (Ishizaki); Irizuki, p. 73, pl. 3, fig. 9^37^.

v. 2006 *Pseudoaurila japonica* (Ishizaki); Irizuki et al., p. 23, fig. 6.10^39^.

non. 2009 *Pseudoaurila japonica* (Ishizaki); Tanaka and Nomura, p. 62, fig. 4.21^196^.

**Type:** Right valve (Holotype), IGPS 90240.

**Remarks:** Ishizaki^1^ provided a line drawing of the internal view (pl. 1, fig. 10) along with an external right lateral view from the optical photograph (pl. 5, fig. 5) of the holotype. Additionally, he included an optical photograph of the left valve (IGPS 90241, paratype, pl. 5, fig. 6). The present reexamination focuses on the external right view of the holotype. Ishizaki and Kato^342^ introduced a new genus, *Pseudoaurila*, with *Aurila japonica* as the type species and illustrated two metatypes (IGPS 91721, 91722) from the Late Pleistocene Furuya Mud in Central Honshu, Japan. Tanaka and Nomura^196^ identified *P. japonica* from the Late Miocene Maja Formation on Kumejima Island, Okinawa, Japan. However, this specimen differs from the type specimen by having a short, prominent caudal ridge extending from the postero-ventral area to the caudal process, a widely arched dorsal cardinal ridge, and a broadly arched anterior margin.

**Occurrence:**

*Recent*: Kyushu, Shikoku, and Southwest Japan; the Sea of Japan side of Southwest Honshu, Japan.

*Fossil*: Early Pleistocene Northern Ryukyu, Japan; Pleistocene Cheju Island, Korea; Late Pleistocene Central Honshu, Japan; Holocene Southwest Honshu, Japan.

Genus: *Caudites* Coryell and Fields, 1937^343^

Type Species: *Caudites medialis* Coryell and Fields, 1937^343^

**37.** *Caudites japonicus* Ishizaki, 1971^3^

v*. 1971 *Caudites japonicus* Ishizaki, p. 82-83, pl. 1, fig. 4, pl. 5, figs. 6, 8, pl. 6, figs. 8, 9^3^.

v. 1977 *Hermanites* ? *japonicus* (Ishizaki); Hanai et al., p. 48^8^.

v. 1979 *Ambostracon japonicus* (Ishizaki); Okubo, p. 146-148, figs. 2a-f, 3^333^.

1980 *Ambostracon japonicus* (Ishizaki); Okubo, p. 412^173^.

v. 1986 *Ambostracon japonicus* (Ishizaki); Cheong et al., p. 46, pl. 2, fig. 12^145^.

v. 1996 *Caudites japonica* Ishizaki; Ozawa, p. 108, pl. 2, fig. 7^33^.

v. 1996 *Caudites* ? *japonica* Ishizaki; Tsukagoshi and Kamiya, p. 358, figs. 10A-10D^293^.

v. 1996 *Caudites* ? *japonicus* Ishizaki; Tsukagoshi and Kamiya, p. 360, fig. 12D^293^.

**Type:** Left valve (Holotype), IGPS 91541.

**Remarks:** Ishizaki^3^ provided a drawing of muscle scars (pl. 1, fig. 4) and an SEM of the internal view (pl. 5, fig. 6), along with an external view from an optical photograph (pl. 6, fig. 9) of the holotype. He also included an optical photograph of the right valve (IGPS 91542, paratype, pl. 6, fig. 8) and an SEM image of the internal view (pl. 5, fig. 8). The present reexamination focuses on the external view of the holotype. Okubo^333^ described the soft parts of this species from the intertidal zone of the Seto Inland Sea, Japan.

**Occurrence:**

*Recent*: Ulleung Basin, Korea; Seto Inland Sea, Northern Honshu, Hokkaido, Japan.

*Fossil*: Plio-Pleistocene Southwest Honshu, Japan.

Genus: *Cornucoquimba* Ohmert, 1968^344^

Type Species: *Cornucoquimba aligera* Ohmert, 1968^344^

**38.** *Cornucoquimba tosaensis* (Ishizaki, 1968)^1^

v*. 1968 *Hermanites tosaensis* Ishizaki, p. 41, pl. 2, fig. 4, pl. 8, figs. 13, 14^1^.

v. 1969 *Hermanites tosaensis* Ishizaki; Ishizaki, p. 222, pl. 26, fig. 19^2^.

v. 1971 *Hermanites tosaensis* Ishizaki; Ishizaki, p. 94-95, pl. 4, fig. 3^3^.

v. 1977 *Cornucoquimba tosaensis* (Ishizaki); Hanai et al., p. 48^8^.

v. 1978 *Cornucoquimba tosaensis* (Ishizaki); Yajima, p. 395-397, pl. 49, figs. 2a-2c, text-fig. 8^345^.

v. 1979 *Cornucoquimba tosaensis* (Ishizaki); Okubo, p. 144-145, fig. 2g-h^333^.

1980 *Cornucoquimba tosaensis* (Ishizaki); Okubo, p. 412^173^.

non. 1981 *Cornucoquimba tosaensis* (Ishizaki); Gou et al., p. 180, pl. 80, figs. 9-11, pl. 90, fig. 11^221^.

non. 1982 *Cornucoquimba tosaensis* (Ishizaki); Cai, pl. 1, figs. 19, 20^21^.

v. 1982 *Hermanites tosaensis* Ishizaki; Hou et al., p. 234-235, pl. 84, figs. 14, 15, text-fig. 72^20^.

v.p. 1983 *Hermanites tosaensis* Ishizaki; Gou et al., p.87, pl. 13, figs. 24-28^22^.

v. 1984 *Cornucoquimba tosaensis* (Ishizaki); Ishizaki, p. 42, pl. 1, fig.4^222^.

v. 1986 *Cornucoquimba tosaensis* (Ishizaki); Cheong et al., p. 46, pl. 2, fig. 14^145^.

v. 1986 *Hermanites tosaensis* Ishizaki; Hou and Zhao, p. 238, pl. 7, figs. 4, 5^346^.

non. 1988 *Hermanites tosaensis* Ishizaki; Ruan and Hao, p. 368, pl. 67, figs. 13-15^28^.

non. 1989 *Hermanites tosaensis* Ishizaki; Gou, pl. 169, fig. 6^347^.

v. 1990 *Cornucoquimba tosaensis* (Ishizaki); Lee, p. 262-264, pl. 20, figs. 5, 6^29^.

1990 *Cornucoquimba tosaensis* (Ishizaki); Ruan, p. 133, pl. 1, fig. 20^72^.

non. 1991 *Cornucoquimba tosaensis* (Ishizaki); Cai, p. 119, pl. 6, fig. 11^30^.

non. 1992 *Cornucoquimba tosaensis* (Ishizaki); Ikeya and Suzuki, p. 125, pl. 3, fig. 9^183^.

v. 1992 *Cornucoquimba tosaensis* (Ishizaki); Lee and Paik, p.151, pl. 3, fig. 16^31^.

v. 1993 *Cornucoquimba tosaensis* (Ishizaki); Kamiya and Nakagawa, p. 127, pl. 3, fig.12^14^.

non. 1994 *Cornucoquimba tosaensis* (Ishizaki); Zheng et al., pl. 50, figs. 1, 2^32^.

v. 1996 *Cornucoquimba tosaensis* (Ishizaki); Ozawa, p. 108, pl. 2, fig.10^33^.

v. 1996 *Cornucoquimba tosaensis* (Ishizaki); Tsukagoshi and Kamiya, p. 359, 360, figs. 11A-11D, 12F^293^.

v. 1998 *Cornucoquimba tosaensis* (Ishizaki); Kim et al., p. 129, pl. 2, fig.10^280^.

v. 1998 *Cornucoquimba tosaensis* (Ishizaki); Yamane, p. 43, pl. 4, fig. 3^35^.

v. 2001 *Cornucoquimba tosaensis* (Ishizaki); Kamiya et al., 95, fig. 14.11^36^.

v. 2001 *Cornucoquimba tosaensis* (Ishizaki); Yasuhara and Irizuki, p. 79, pl. 4, figs. 3, 4^151^.

v. 2003 *Cornucoquimba tosaensis* (Ishizaki); Ozawa, p. 264, fig. 7.4^348^.

v. 2003 *Cornucoquimba tosaensis* (Ishizaki); Yasuhara and Kumai, p. 77, pl. 2, fig.4^282^.

non. 2004 *Cornucoquimba tosaensis* (Ishizaki); Irizuki et al., p. 75, pl. 2, fig. 1^37^.

2004 *Hermanites tosaensis* Ishizaki; Yamauchi, p. 73^38^.

v. 2005 *Cornucoquimba tosaensis* (Ishizaki); Irizuki et al., p. 42, fig.4.1^162^.

non. 2007 *Cornucoquimba tosaensis* (Ishizaki); Irizuki et al., p. 429, fig. 6.7^352^.

v. 2008 *Hermanites tosaensis* Ishizaki; Hu and Tao, p. 254-255, pl. 35, figs. 6, 9, 15, pl. 44, figs, 17, 18, pl. 67, figs. 8, 13, 23, pl. 178, fig. 14, pl. 222, figs. 4, 7, 20, text-fig. 158^17^.

v. 2008 *Cornucoquimba tosaensis* (Ishizaki); Tanaka, p. 276, fig. 2k^349^.

v. 2009 *Cornucoquimba tosaensis* (Ishizaki); Horikoshi et al., p. 154, fig. 5.4^261^.

v. 2009 *Cornucoquimba tosaensis* (Ishizaki); Ozawa, p. 236, fig. 3.19^218^.

v. 2009 *Cornucoquimba tosaensis* (Ishizaki); Tanaka and Nomura, p. 61, fig. 3.17^196^.

v. 2010 *Cornucoquimba tosaensis* (Ishizaki); Irizuki et al., p. 16, fig. 4.13^245^.

v. 2012 *Cornucoquimba tosaensis* (Ishizaki); Tanaka et al., p. 19, pl. 3, fig. 18^40^.

v. 2012 *Cornucoquimba tosaensis* (Ishizaki); Tanaka et al., p. 118, pl. 2, fig. 12^199^.

v. 2014 *Cornucoquimba tosaensis* (Ishizaki); Masuma and Yamada., p. 5, fig. 3.11^41^.

v. 2021 *Cornucoquimba tosaensis* (Ishizaki); Yamada et al., p. 582, fig. 7-4^340^.

non. 2017 *Cornucoquimba tosaensis* (Ishizaki); Tsukagoshi, p. 16, fig. 2C^201^.

**Type:** Left valve (Holotype), IGPS 90313.

**Remarks:** Ishizaki^1^ illustrated a line drawing of the internal view (pl. 2, fig. 4) and an external view of the optical photograph (pl. 8, fig. 14) of the holotype. He also provided an optical photograph of a right valve (IGPS 90314, paratype, pl. 8, fig. 13). The current reexamination presents the external view of the holotype. Gou et al.^221^ depicted this species from Pliocene borehole cores in the South China Sea, but it differs in having a vertical ridge extending from the postero-dorsal to the postero-ventral area, a short carinal ridge in the mid-dorsal area, and a straight carinal ridge along the ventral margin. Cai^21^ also illustrated this species from the South China Sea; however, it differs by featuring a posteriorly projected caudal process, a short carinal ridge running ventrally at the mid-posterior area, and vertical short carinal ridges at the mid-dorsal area. Ruan and Hao^28^ identified this species from Holocene borehole cores in the Okinawa Trough, Japan; however, it differs by having a straight carinal ridge in the antero-median area, a broadly arched posterior margin, and the absence of a carinal ridge running from the postero-dorsal area to the ventral area. Gou^347^ depicted this species from Quaternary borehole core in the East China Sea; however, it differs from the type species by having a V-shaped carinal ridge in the postero-dorsal area, a postero-ventral spine running parallel to the posterior margin, and the absence of a carinal ridge running from the postero-dorsal area to the ventral area. Cai^30^ and Zheng et al.^32^ reported this species from the Nansha Islands in the South China Sea; however, it differs by having two bulbs in the posterior area, the absence of a carinal ridge running from the postero-dorsal area to the ventral area, and a lack of ventral carinal ridges. Ikeya and Suzuki^183^ found this species from off Shimane, Southwest Japan; however, it differs by lacking a carinal ridge running from the postero-dorsal area to the ventral area, a postero-ventral spine, and a ventral carinal ridge running parallel to the ventral margin. Irizuki et al.^350^ depicted *Cornucoquimba tosaensis* from the Middle Pliocene Kuwae Formation, which differs from the type specimen by having a denticulated anterior margin, regular reticulation in the anterior area, and a robust spine in the postero-ventral area. *Cornucoquimba tosaensis*, as depicted by Tsukagoshi^201^, is not this species for the same reasons. *Cornucoquimba tosaensis* illustrated by Kim et al.^280^ from Holocene borehole sediment differs from the type specimen by having a vertical ridge extending from the postero-dorsal to the postero-ventral area, a spine in the mid-dorsal area, and regular reticulation.

**Occurrence:**

*Recent*: Matsu Islands, Northwestern Taiwan; Ulleung Basin, Korea; Tsushima Straits; Sea of Japan; Southwest Honshu, Japan; Shikoku, Seto Inland Sea; the Sea of Japan side of Southwestern Honshu; Central Honshu; Northern Honshu; Hokkaido, Japan.

*Fossil*: Late Miocene Okinawa, Japan; Pliocene Leizhou Peninsula, South China; Pliocene Southern Taiwan; Late Pliocene, Okinawa, Japan; Late Pliocene Northwestern Taiwan; Plio-Pleistocene Central Honshu, Japan; Pleistocene Cheju Island, Korea; Middle Pleistocene of Southwest and Central Honshu, Japan; Holocene Jiangsu, China; Southern Taiwan; Southwest and Central Honshu, Japan.

Genus: *Finmarchinella* Swain, 1963^351^

Type Species: *Cythereis finmarchica* Sars, 1866^5^

**39.** *Finmarchinella uranipponica* Ishizaki, 1969^2^

v*. 1969 *Finmarchinella uranipponica* Ishizaki, p. 217-218, pl. 26, figs. 12, 13, pl. 24, fig. 4^2^.

v. 1971 *Finmarchinella uranipponica* Ishizaki; Ishizaki, p. 83, pl. 3, fig. 3^3^.

v. 1974 *Finmarchinella (Finmarchinella) uranipponica* Ishizaki; Neale, p. 86^352^.

v. 1977 *Finmarchinella (Finmarchinella) uranipponica* Ishizaki; Hanai et al., p. 45-46^8^.

v. 1986 *Finmarchinella (Finmarchinella) uranipponica* Ishizaki; Cheong et al., p. 46, pl. 2, fig. 7^145^.

v. 1990 *Finmarchinella (Finmarchinella) uranipponica* Ishizaki; Lee, p. 256-258, pl. 18, figs. 7-13^29^.

v. 1990 *Finmarchinella (Finmarchinella) uranipponica* Ishizaki; Yajima and Lord, p. 157, fig. 14^213^.

v. 1991 *Finmarchinella uranipponica* Ishizaki; Ikeya and Itoh, p. 137, fig. 16A^149^.

v. 1992 *Finmarchinella (Finmarchinella) uranipponica* Ishizaki; Lee and Paik, p. 151, pl. 3, fig. 14^31^.

v. 1993 *Finmarchinella uranipponica* Ishizaki; Irizuki, p. 189, fig. 2.12^353^.

v. 1993 *Finmarchinella uranipponica* Ishizaki; Kamiya and Nakagawa, p. 127, pl. 3, fig. 11^14^.

v. 1996 *Finmarchinella uranipponica* Ishizaki; Tsukagoshi and Kamiya, p. 347, 355, 360, figs. 1, 9E-9H, 12A^293^.

v. 1998 *Finmarchinella uranipponica* Ishizaki; Tanaka et al., p. 91, pl. 1, fig. 20^257^.

v. 2001 *Finmarchinella uranipponica* Ishizaki; Yasuhara and Irizuki, p. 81, pl. 5, figs. 8, 9^151^.

v. 2001 *Finmarchinella uranipponica* Ishizaki; Kamiya et al., 101, fig. 17.18^36^.

2004 *Finmarchinella uranipponica* Ishizaki; Yamauchi, p. 72^38^.

2006 *Finmarchinella (Finmarchinella) uranipponica* Ishizaki; Schornikov, p.41^16^.

2006 *Finmarchinella (Finmarchinella) uranipponica* Ishizaki; Schornikov and Zanina, p.216, table 2^15^.

v. 2010 *Finmarchinella uranipponica* Ishizaki; Ozawa, p. 31, pl. 2, fig. 12^208^.

v. 2014 *Finmarchinella uranipponica* Ishizaki; Schornikov and Zanina, p. 33, pl. 3, figs. 22, 23^156^.

2014. *Finmarchinella uranipponica* Ishizaki; Goto et al., p. 78, fig. 6.7^164^.

v. 2015 *Finmarchinella uranipponica* Ishizaki; Irizuki et al., p. 470, fig. 3.8^265^.

**Type:** Left valve (Holotype), IGPS 87044.

**Remarks:** Ishizaki^2^ provided a line drawing of the internal view (pl. 24, fig. 4) and an optical photograph of an external view of the holotype (pl. 26, fig. 13) of the holotype. He also included an optical photograph of a juvenile right valve (IGPS 87050, paratype, pl. 26, fig. 12). The current reexamination presents the external view of the holotype. Ishizak designated the holotype as a juvenile specimen because the duplicature has not yet developed. Schornikov and Zanina^156^ illustrated adult female and male specimens from Peter the Great Bay, Russia.

**Occurrence:**

*Recent*: Sea of Japan side of Southwest Honshu, Northern Honshu, Japan; Southwest Honshu, Japan; Ulleung Basin, Korea; Peter the Great Bay, Russia; Sendai Bay, Japan.

*Fossil*: Late Pliocene from the Sea of Japan side of Northwestern Honshu, Japan; Middle to Late Pleistocene from Cheju Island, Korea; Middle Pleistocene from Central Honshu, Japan; Middle Pleistocene from Sado Island, Japan; Late Pleistocene from Northern Honshu, Japan; Holocene from Southwest and Northern Honshu, Japan.

Genus: *Urocythereis* Ruggieri, 1950^354^

Type Species: *Cytherina favosa* Roemer, 1838^355^

**40.** *Urocythereis miii* Ishizaki, 1969^2^

v*. 1969 *Urocythereis miii* Ishizaki, p. 218-219, pl. 25, figs. 11, 12, pl. 24, figs. 5, 6^2^.

v. 1977 *Hemicythere* ? *miii* (Ishizaki); Hanai et al., p. 42^8^.

non. 1981 *Hemicythere* ? *miii* (Ishizaki); Gou et al., p.161, pl.79, figs. 8-11^221^.

v. 1990 *Hemicythere* ? *miii* Ishizaki; Takayasu et al., pl. 1, fig. 10^212^.

**Type:** Right valve (Holotype), IGPS 90332.

**Remarks:** Ishizaki^2^ illustrated the muscle scars in a drawing (pl. 24, fig. 6) and provided an external view in an optical photograph (pl. 25, fig. 11) of the holotype. He also presented a line drawing of the internal view (pl. 24, fig. 5) and an optical photograph of the external view (pl. 25, fig. 12) of the left valve (IGPS 90333). The current reexamination reveals the external view of the holotype. Gou et al.^221^ depicted this species from Early Miocene borehole cores in the South China Sea; however, it differs in having a carinal ridge that extends from the middle of the dorsal margin to below the eye tubercle, a straight dorsal margin, and four radiated carinal ridges at the anterior area.

**Occurrence:**

*Recent*: Sea of Japan side of Southwest Japan.

*Fossil*: unknown.

Family: Trachyleberididae Sylvester-Bradley, 1948^356^

Genus: *Pistocythereis* Gou in Gou et al. (1983)^22^

Type Species: *Echinocythereis bradyi* Ishizaki, 1968^1^

**41.** *Pistocythereis bradyformis* (Ishizaki, 1968)^1^

v. 1913 *Cythereis darwini* Brady; Kajiyama, p. 12, 13, pl. 1, figs. 67-69^169^.

v. 1959 *Cythereis* sp. Hanai, p. 373, fig. 3a, b, text-fig. 14^170^.

v*. 1968 *Echinocythereis bradyformis* Ishizaki, p. 40, pl. 8, fig. 4^1^.

v. 1971 *Echinocythereis bradyformis* Ishizaki; Ishizaki, p. 94, pl. 4, fig. 1^3^.

v. 1977 *Echinocythereis* ? *bradyformis* Ishizaki; Hanai et al., p.51^8^.

v. 1979 *Echinocythereis bradyi* Ishizaki; Okubo, p. 51-52, fig. 7g, h^333^.

non. 1980 *Echinocythereis bradyformis* Ishizaki; Kim and Park, p. 47, pl. 1, fig. 11^271^.

1980 *Ruggieria* sp. A; Kim and Park, p. 47, pl. 1, figs. 26, 27^271^.

1980 *Ruggieria* sp. B; Kim and Park, p. 47, pl. 1, fig. 28^271^.

v.p. 1981 *Echinocythereis bradyformis* Ishizaki; Gou et al., p. 179, pl. 86, fig. 7, pl. 91, fig. 1^221^.

non. 1981 *Echinocythereis bradyformis* Ishizaki; Gou et al., p. 179, pl. 86, fig. 6, 8-10, pl. 90, fig. 10^221^.

v. 1982 *Echinocythereis bradyi* Ishizaki; Cai, p. 6, pl. 2, figs. 4, 5^21^.

non. 1982 *Echinocythereis bradyformis* Ishizaki; Cai, p. 6, pl. 2, figs. 10, 11^21^.

v. 1982 *Wichmannella bradyformis* (Ishizaki); Yajima, p. 201, pl. 12, figs. 14-17, text-fig. 15-6^286^.

v. 1983 *Pistocythereis bradyformis* (Ishizaki); Gou et al., p.95-96, pl. 4, figs. 25-28, pl. 15, figs. 1-6^22^.

v. 1984 *Pistocythereis bradyformis* (Ishizaki); Ishizaki, p. 42, pl. 1, fig. 1, pl. 2, fig. 9^222^.

1984 Gen. et sp. indet.; Ishizaki, p. 43, pl. 2, fig. 3^222^.

v.1985 *Pistocythereis bradyformis* (Ishizaki); Li, pl. 1, fig. 26^65^.

v. 1985 *Echinocythereis* ? *bradyformis* Ishizaki; Wang, et al., pl. 27, fig. 18^357^.

non. 1985 *Echinocythereis* ? *bradyi* Ishizaki; Zhao et al., p. 352, pl. 20, fig. 3^358^.

1985 *Echinocythereis* ? *bradyformis* Ishizaki; Wang, et al., p. 365, pl. 33, figs. 11, 12^359^.

v. 1985 *Echinocythereis* ? *bradyformis* Ishizaki; Wang and Zhao, p. 340, pl. 8, fig. 3^287^.

v. 1985 *Echinocythereis* ? *bradyformis* Ishizaki; Wang and Bian, p. 347, pl. 15, fig. 10^360^.

v. 1985 *Echinocythereis* ? *bradyformis* Ishizaki; Zhao, pl. 2, fig. 5^160^.

v. 1986 *Echinocythereis bradyformis* Ishizaki; Lin and Zhu, pl. 2, fig. 11^273^.

non. 1986 *Wichmannella bradyformis* (Ishizaki); Zhao. et al., pl. 1, fig. 16^314^.

v.1987 *Pistocythereis bradyformis* (Ishizaki); Ikeya et al., p. 64, fig. 7^147^.

v.1987 *Pistocythereis bradyi* (Ishizaki); Zheng, p. 203, pl. 8, figs. 27, 28^25^.

v.1987 *Pistocythereis bradyformis* (Ishizaki); Zheng, p. 204, pl. 8, figs. 7, 8^25^.

v.1987 *Wichmannella bradyi* (Ishizaki); Wang and Zhang, p. 291, pl. 2, fig. 22^146^.

v.1988 *Pistocythereis bradyformis* (Ishizaki); Cai, pl. 2, fig. 14^26^.

v. 1988 *Pistocythereis bradyformis* (Ishizaki); Ikeya and Kashima, p. 202, fig. 6^275^.

v.p. 1988 *Pistocythereis bradyformis* (Ishizaki); Ruan and Hao, p. 363, pl. 65, figs. 15, 19, 20^28^.

non. 1988 *Pistocythereis bradyformis* (Ishizaki); Ruan and Hao, p. 363, pl. 65, figs. 16-18^28^.

v.p. 1989 *Pistocythereis bradyformis* (Ishizaki); Ruan, pl. 2, fig. 17^161^.

non. 1989 *Pistocythereis bradyformis* (Ishizaki); Ruan, pl. 2, fig. 15, 16^161^.

v. 1990 *Pistocythereis bradyformis* (Ishizaki); Ishizaki, p. 214, fig. 1.6^278^.

v. 1990 *Pistocythereis bradyformis* (Ishizaki); Lee, p. 300-301, pl. 25, fig. 9^29^.

v. 1990 *Pistocythereis bradyformis* (Ishizaki); Takayasu et al., p. 145, pl. 1, fig. 6^212^.

v. 1990 *Pistocythereis bradyformis* (Ishizaki); Zhao and Wang, pl. 1, fig. 13^148^.

v. 1992 *Pistocythereis bradyformis* (Ishizaki); Lee and Paik, p. 153, pl. 4, fig. 13^31^.

v. 1992 *Pistocythereis bradyformis* (Ishizaki); Mostafawi, p. 146-147, pl. 4, fig. 76^74^.

v. 1993 *Pistocythereis bradyformis* (Ishizaki); Kamiya and Nakagawa, p. 129, pl. 4, fig. 10^14^.

v. 1994 *Pistocythereis bradyformis* (Ishizaki); Zheng et al., pl. 51, figs. 2, 3^32^.

v. 1996 *Pistocythereis bradyformis* (Ishizaki); Ozawa, p. 114, pl. 8, fig. 3^33^.

non. 1997 *Pistocythereis bradyformis* (Ishizaki); Dewi, p. 72, figs. 187, 188^87^.

v.p. 1998 *Pistocythereis bradyformis* (Ishizaki); Cao, pl. 8, fig. 4^34^.

non. 1998 *Pistocythereis bradyformis* (Ishizaki); Cao, pl. 8, figs. 8-13^34^.

v. 1998 *Pistocythereis bradyformis* (Ishizaki); Cao, pl. 8, figs. 14-16^34^.

v. 1998 *Pistocythereis bradyformis* (Ishizaki); Irizuki et al., p. 8, fig. 3-3^150^.

v. 1998 *Pistocythereis bradyformis* (Ishizaki); Yamane, p. 53, pl. 9, fig. 5^35^.

non.2000 *Wichmannella bradyformis* (Ishizaki); Hu and Tao, p. 29, figs. 30, 31^361^.

v. 2000 *Pistocythereis bradyformis* (Ishizaki); Irizuki and Hosoyama, p. 12, fig. 3.11^226^.

v. 2001 *Pistocythereis bradyformis* (Ishizaki); Yasuhara and Irizuki, p. 89, pl. 9, figs. 5-10^151^.

v. 2001 *Pistocythereis bradyformis* (Ishizaki); Kamiya et al., 99, fig. 16.12^36^.

v. 2003 *Pistocythereis bradyformis* (Ishizaki); Irizuki et al., p. 13, pl. 1, fig. 6^308^.

2004 *Pistocythereis bradyformis* (Ishizaki); Yamauchi, p. 73^38^.

v. 2005 *Pistocythereis bradyformis* (Ishizaki); Irizuki et al., p. 42, fig. 4.5^162^.

v. 2006 *Pistocythereis bradyformis* (Ishizaki); Irizuki et al., p. 24, fig. 7.5^39^.

v. 2006 *Pistocythereis bradyformis* (Ishizaki); Yasuhara and Seto, p. 107, fig. 5a^243^.

v. 2007 *Pistocythereis bradyformis* (Ishizaki); Sasaki et al., p. 524, fig. 5.14^154^.

v. 2008 *Pistocythereis bradyformis* (Ishizaki); Hu and Tao, pl. 19, fig. 1^17^.

2008 *Pistocythereis bradyformis* (Ishizaki); Hu and Tao, pl. 19, fig. 2, 3, 6, 8, 12, pl. 116, figs. 1, 2, 9, 13, pl. 162, figs. 6, 15^17^.

non. 2008 *Pistocythereis bradyformis* (Ishizaki); Hu and Tao, p. 310-311, pl. 162, fig. 19, 21, text-fig. 187^17^.

v. 2009 *Amphileberis nipponica* (Yajima); Horikoshi et al., 154, fig. 5.11^261^.

v. 2009 *Pistocythereis bradyformis* (Ishizaki); Ozawa, p. 238, fig. 4.15^218^.

v. 2010 *Pistocythereis bradyformis* (Ishizaki); Irizuki et al., p. 16, fig. 4.8^245^.

v. 2011 *Pistocythereis bradyformis* (Ishizaki); Irizuki et al., p. 2035, fig. 5^264^.

v. 2011 *Pistocythereis bradyformis* (Ishizaki); Irizuki et al., p. 42, fig. 4.8^219^.

v. 2012 *Pistocythereis bradyformis* (Ishizaki); Tanaka et al., p. 9, fig. 4N^237^.

v. 2014 *Pistocythereis bradyformis* (Ishizaki); Noraswana et al., p. 31, figs. 3.5, 3.6^362^.

v. 2014 *Pistocythereis bradyi* (Ishizaki); Ramlan and Noraswana, p. 442, fig. 4.4^363^.

v. 2014 *Pistocythereis bradyformis* (Ishizaki); Masuma and Yamada, p. 6, fig. 4.12^41^.

v. 2017 *Pistocythereis bradyformis* (Ishizaki); Hong et al., p. 58, fig. 8.37^125^.

v. 2019 *Pistocythereis bradyformis* (Ishizaki); Irizuki et al., p. 267, fig. 5.22^168^.

v. 2019 *Pistocythereis bradyformis* (Ishizaki); Tanaka et al., p. 34, fig. 4p^248^.

v. 2019 *Pistocythereis bradyformis* (Ishizaki); Tanaka et al., p. 11, fig. 3.18-20, p. 12, fig. 4.1, 4.2, p. 13, fig. 6^203^.

v. 2021 *Pistocythereis bradyformis* (Ishizaki); Tan et al., p. 6, fig. 2.8^138^.

v. 2024 *Pistocythereis bradyformis* (Ishizaki); Maekawa, fig. 182.63^285^.

**Type:** Male left valve (Holotype), IGPS 90311.

**Remarks:** Ishizaki^1^ illustrated an optical photograph of the holotype’s external view (pl. 8, fig. 4). The current reexamination confirms the holotype. Some specimens of *Echinocythereis bradyformis* reported by Gou et al. ^221^ from Late Miocene to Pliocene borehole cores in the South China Sea differ from the type specimen in having a narrowly arched anterior margin, a projected posterior margin, and fewer surface reticulations. A specimen illustrated by Zhao et al.^358^ from the South China Sea off Guangdong Province also differs from the type specimen with its narrowly arched anterior margin, projected posterior margin, and fewer surface reticulations. Some specimens identified by Ruan and Hao^28^ from Late Pleistocene borehole cores in the Okinawa Trough are not classified as this species due to their broadly arched anterior margin, posteriorly inclined dorsal margin, and postero-ventrally arched posterior margin. Two specimens illustrated by Ruan^161^ do not belong to this species as they have an acutely curved anterior margin, a straight postero-ventral margin, and fewer anterior denticles. Dewi^87^ reported this species from Holocene borehole cores on Java Island, Indonesia; however, it differs from the type specimen by having radially arranged reticulation, a long carinal ridge along the ventral margin, and an acutely arched posterior margin. Cao^34^ illustrated several specimens of this species; however, the adult specimens are *P. bradyi*. Hu and Tao^361^ reported this species from recent sediments in Singapore; however, it differs by having a long ventral carinal ridge, finer reticulation in the anterior area, and an anteriorly arched anterior margin. The specimens (pl. 162, fig. 19, 21) illustrated by Hu and Tao^17^ are not *P. bradyformis* but *P. bradyi*. Ramlan and Noraswana^363^ identified *P. brady* as *P. bradyformis* based on its bifurcated ventral carinal ridge.

**Occurrence:**

*Recent*: East coast of the Malay Peninsula; Central Vietnam; Sunda Shelf; Pearl River Mouth, Hong Kong; South China Sea; Penghu Islands and Southwestern coast of Taiwan; coast of Hebeiss, Bohai Sea, China; East China Sea, Bohai Sea, China; shelf of central to south China; Kyushu, Shikoku, Seto Inland Sea; Sea of Japan side of Southwest, Central, and Northern Honshu, Japan.

*Fossil*: Pliocene from Leizhou Peninsula; Pliocene from Taiwan; Late Pliocene from Northwestern Taiwan; Plio-Pleistocene from Taiwan; Plio-Pleistocene from Central Honshu, Japan; Middle to Late Pleistocene from Cheju Island, Korea; Middle Pleistocene from Kyushu, Southwest and Central Honshu, Japan; Late Pleistocene from Southwest and Central Honshu, Japan; Pleistocene from Taiwan; Late Pleistocene from Taiwan; Late Pleistocene and Holocene from Okinawa Trough, Japan; Quaternary from Fujiang, China; Quaternary from Hong Kong, China; Holocene from Seto Inland Sea, Southwest, Central, and Northeast Honshu, Japan; Holocene from a borehole core in Hong Kong, China.

**42.** *Pistocythereis bradyi* (Ishizaki, 1968)^1^

v. 1880 *Cythere darwini* Brady; Brady, p. 97, 98, pl. 25, fig. 2a-g^268^.

v. 1912 *Cythere* sp. Müller, p.323^364^.

v*. 1968 *Echinocythereis bradyi* Ishizaki, p. 40, pl. 9, fig. 17^1^.

v. 1969 *Echinocythereis bradyi* Ishizaki; Ishizaki, p. 222, pl. 25, fig. 14^2^.

v. 1971 *Echinocythereis bradyi* Ishizaki; Ishizaki, p. 94, pl. 4, fig. 2^3^.

v. 1977 *Echinocythereis* ? *bradyi* Ishizaki; Hanai et al., p. 51-52^8^.

non. 1979 *Echinocythereis bradyi* Ishizaki; Okubo, p. 51-52, fig. 7g, h^333^.

non. 1981 *Echinocythereis bradyi* Ishizaki; Gou et al., p. 179, pl. 86, figs. 3-5^221^.

non. 1982 *Echinocythereis bradyi* Ishizaki; Cai, p. 6, pl. 2, figs. 4, 5^21^.

non.1982 *Echinocythereis bradyi* Ishizaki; Hou et al., p. 232-233, pl. 84, figs. 1-6^20^.

v. 1982 *Echinocythereis bradyformis* Ishizaki; Cai, p. 6, pl. 2, figs. 10, 11^21^.

1982 *Wichmannella bradyi* (Ishizaki); Yajima, p. 201^286^.

v. 1983 *Pistocythereis bradyi* (Ishizaki); Gou et al., p.94-95, pl. 14, figs. 1-12, pl. 5, fig. 1, text-fig. 31a, b^22^.

1983 *Echinocythereis* ? *bradyi* Ishizaki; Ikeya, p. 2, fig. 1.7^272^.

v. 1984 *Pistocythereis bradyi* (Ishizaki); Ishizaki, p. 42, pl. 1, fig. 2^222^.

v. 1985 *Pistocythereis bradyi* (Ishizaki); Li, pl. 1, fig. 14^65^.

v. 1985 *Wichmannella bradyi* (Ishizaki); Tabuki and Nohara, pl. 2, fig. 16^365^.

v. 1985 *Echinocythereis* ? *bradyi* Ishizaki; Zhao et al., p. 352, pl. 20, fig. 2^358^.

v. 1986 *Echinocythereis* *bradyi* Ishizaki; Lin and Zhu, pl. 3, fig. 4^273^.

non. 1986 *Echinocythereis* *bradyi* Ishizaki; Lin and Zhu, p. 97, pl. 2, fig. 5^273^.

v. 1986 *Wichmannella bradyi* (Ishizaki); Zhao et al., pl. 1, fig. 15^314^.

non.1986 *Pistocythereis bradyi* (Ishizaki); Zheng, pl. 3, figs. 14-19^274^.

v.1987 *Pistocythereis bradyi* (Ishizaki); Ikeya et al., p. 64, fig. 6^147^.

v.1987 *Pistocythereis bradyi* (Ishizaki); Tabuki et al., p. 335, pl. 2, fig. 6^253^.

non.1987 *Pistocythereis bradyi* (Ishizaki); Zheng, p. 203, pl. 8, figs. 27, 28^25^.

non.1987 *Wichmannella bradyi* (Ishizaki); Wang and Zhang, p. 291, pl. 2, fig. 22^146^.

v.1988 *Pistocythereis bradyi* (Ishizaki); Cai, pl. 2, fig. 14^26^.

v. 1988 *Wichmannella bradyi* (Ishizaki); Wang, et al., p. 103, pl. 1, fig. 3^276^.

v.1988 *Pistocythereis bradyi* (Ishizaki); Whatley and Zhao, p. 19, pl. 9, figs. 3-5^67^.

v.1988 *Pistocythereis bradyi* (Ishizaki); Zhao and Whatley, p. 167, pl. 2, fig. 8^68^.

1989 *Pistocythereis bradyi* (Ishizaki); Zhao and Whatley, p. 187^366^.

v. 1990 *Pistocythereis bisanensis* (Ishizaki); Takayasu et al., p. 145, pl. 1, fig. 7^212^.

v. 1990 *Pistocythereis bradyi* (Ishizaki); Ishizaki, p. 214, fig. 1.5^278^.

v. 1990 *Pistocythereis bradyi* (Ishizaki); Tabuki and Nohara, pl. 2, fig. 6^254^.

v. 1991 *Pistocythereis bradyi* (Ishizaki); Cai, p. 111, pl. 4, fig. 12^30^.

v. 1992 *Pistocythereis bradyi* (Ishizaki); Iwasaki, p. 11, pl. 1, fig. 10^242^.

v. 1992 *Pistocythereis bradyi* (Ishizaki); Mostafawi, p. 146, pl. 4, fig. 15^74^.

v. 1994 *Pistocythereis bradyi* (Ishizaki); Zheng et al, pl. 56, figs. 4-6^32^.

non. 1997 *Pistocythereis bradyi* (Ishizaki); Dewi, p. 72, fig. 186^87^.

v. 1998 *Pistocythereis bradyformis* (Ishizaki); Cao, pl. 8, figs. 7, 8-13^34^.

non. 1998 *Pistocythereis bradyformis* (Ishizaki); Cao, pl. 8, figs. 14-16^34^.

v. 1998 *Pistocythereis bradyi* (Ishizaki); Irizuki et al., p. 8, fig. 3.4^150^.

v. 1998 *Pistocythereis bradyformis* (Ishizaki); Tanaka et al., p. 91, pl. 1, fig. 19^257^.

v. 1998 *Pistocythereis bradyi* (Ishizaki); Yamane, p. 53, pl. 9, fig. 6^35^.

non. 2000 *Wichmannella bradyi* (Ishizaki); Hu and Tao, p. 28, figs. 29A, 29B^361^.

v. 2000 *Pistocythereis bradyi* (Ishizaki); Irizuki and Hosoyama, p. 12, fig. 3.12^226^.

v. 2001 *Pistocythereis bradyi* (Ishizaki); Yasuhara and Irizuki, p. 89, pl. 9, figs. 11-16^151^.

v. 2001 *Pistocythereis bradyi* (Ishizaki); Kamiya et al., 103, fig. 18.10^36^.

v. 2001 *Pistocythereis bradyi* (Ishizaki); Tabuki, p. 33, fig. 10.11^186^.

v. 2003 *Pistocythereis bradyi* (Ishizaki); Irizuki et al., p. 13, pl. 1, fig. 7^308^.

v. 2004 *Pistocythereis bradyi* (Ishizaki); Irizuki and Seto, p. 314, fig. 1^367^.

2004 *Pistocythereis bradyi* (Ishizaki); Yamauchi, p. 73^38^.

v. 2004 *Pistocythereis bradyi* (Ishizaki); Yasuhara et al., p. 30, fig. 12c, p. 31, fig. 13c, p. 32, fig. 14c, p. 33, fig. 15c^230^.

v. 2005 *Pistocythereis bradyi* (Ishizaki); Irizuki et al., p. 42, fig. 4.5^162^.

v. 2005 *Pistocythereis bradyi* (Ishizaki); Ishida and Takayasu, p. 76, fig. 2.11^216^.

v. 2006 *Pistocythereis bradyformis* (Ishizaki); Irizuki et al., p. 24, fig. 7.6^39^.

v. 2006 *Pistocythereis bradyformis* (Ishizaki); Yasuhara and Seto, p. 107, fig. 5c^243^.

v. 2007 *Pistocythereis bradyi* (Ishizaki); Nor Faiz et al., p. 147, pl. 2, fig. 6^107^.

2007 *Pistocythereis bradyi* (Ishizaki); Nor Faiz et al., p. 147, pl. 2, fig. 7^107^.

v. 2007 *Pistocythereis bradyi* (Ishizaki); Sasaki et al., p. 524, fig. 5.22^154^.

v. 2008 *Pistocythereis bradyi* (Ishizaki); Hu and Tao, pl. 55, fig. 19, pl. 69, figs. 8, 21, pl. 95, fig. 5, pl. 149, fig. 3, pl. 162, figs. 2, 5,18, pl. 163, fig. 12^17^.

2008 *Pistocythereis bradyi* (Ishizaki); Hu and Tao, pl. 10, figs. 5, 7, 9, 10, 13, 18, pl. 47, fig. 15, pl. 149, figs. 1, 2, pl. 162, fig. 17, pl. 163, figs. 3, 7, pl. 187, fig. 7^17^.

non. 2008 *Pistocythereis bradyi* (Ishizaki); Hu and Tao, p. 311, pl. 21, figs. 1-3, pl. 27, figs. 2-6, pl. 37, fig. 6, pl. 47, figs. 8, 9, 11, pl. 69, fig. 18, pl. 84, figs. 17-20, pl. 85, fig. 1, pl. 90, fig. 19, pl. 94, figs. 8, 9, 13, pl. 95, figs. 1, 3, 4, pl. 105, figs. 3, 5, 6, 8, 9, 10-12, 14-17, pl. 116, figs. 8, 10, 11, pl. 139, figs. 7, 8, 17, 20, 24, 26, pl. 161, figs. 18, 20, 21, pl. 163, figs. 1, 4, 9, pl. 187, figs. 2, 3, 6, 8-11, pl. 199, figs. 1, 2, pl. 210, fig. 5, pl .211, figs. 11, 12, 14, text-fig. 188^17^.

v. 2008 *Pistocythereis bradyi* (Ishizaki); Irizuki et al., p. 296, fig. 8^244^.

v. 2009 *Pistocythereis bradyi* (Ishizaki); Ramlan and Noraswana, p. 17, pl. 3, figs. 1, 2^368^.

v. 2009 *Pistocythereis* sp. Ramlan and Noraswana, p. 17, pl. 3, figs. 3, 4^368^.

v. 2009 *Pistocythereis bradyi* (Ishizaki); Tanaka and Nomura, p. 62, fig. 4.19^196^.

v. 2010 *Pistocythereis bradyi* (Ishizaki); Irizuki et al., p. 16, fig. 4.9^245^.

v. 2010 *Pistocythereis bradyi* (Ishizaki); Ramlan and Noraswana, p. 202, pl. 1, figs. 1, 2^369^.

v. 2011 *Pistocythereis bradyi* (Ishizaki); Irizuki et al, p. 2035, fig. 5^264^.

v. 2011 *Pistocythereis bradyi* (Ishizaki); Tanaka et al, p. 34, fig. 6B^248^.

v. 2012 *Pistocythereis bradyi* (Ishizaki); Tanaka et al, p. 18, pl. 3, fig. 12^40^.

v. 2012 *Pistocythereis bradyi* (Ishizaki); Tanaka et al, p. 118, pl. 12, fig. 11^199^.

v. 2012 *Pistocythereis bradyi* (Ishizaki); Tanaka et al., p. 9, fig. 4O^237^.

v. 2013 *Pistocythereis bradyi* (Ishizaki); Kawano et al, p. 96, fig. 5.12^200^.

non. 2014 *Pistocythereis bradyi* (Ishizaki); Ramlan and Noraswana, p. 442, fig. 4.4^363^.

v. 2015 *Pistocythereis bradyi* (Ishizaki); Yasuhara et al, p. 150, figs. 84T-84W, 85O-85R, table 1^370^.

v. 2015 *Pistocythereis bradyi* (Ishizaki); Yamada et al, p. 56, fig. 3.12^42^.

v. 2015 *Pistocythereis bradyi* (Ishizaki); Irizuki et al., p. 470, fig. 3.14^265^.

v. 2016 *Pistocythereis bradyi* (Ishizaki); Li et al, p. 1477, figs. 2.1a, 2.1b^371^.

v. 2016 *Pistocythereis bradyi* (Ishizaki); Matsushima et al., p. 14, fig. 6H^238^.

v. 2017 *Pistocythereis bradyi* (Ishizaki); Omar et al., p. 18, fig. 2^372^.

v. 2017 *Pistocythereis bradyi* (Ishizaki); Tsukagoshi, p. 15, fig. 2E^201^.

v. 2018 *Pistocythereis bradyi* (Ishizaki); Irizuki et al., p. 643, fig. 5.11^157^.

v. 2019 *Pistocythereis bradyi* (Ishizaki); Hong et al., p. 596, fig. 5^158^.

v. 2019 *Pistocythereis bradyi* (Ishizaki); Irizuki et al., p. 267, fig. 5.21^168^.

non. 2019 *Pistocythereis bradyi* (Ishizaki); Niiyama et al., p. 105, fig. 4.11^133^.

v. 2019 *Pistocythereis bradyi* (Ishizaki); Tanaka et al., p. 34, fig. 4q^248^.

v. 2019 *Pistocythereis bradyi* (Ishizaki); Tanaka et al., p. 12, fig. 4.3-5, p. 13, fig. 7^203^.

v. 2020 *Pistocythereis bradyi* (Ishizaki); Sasaki et al., p. 8, fig. 4.9^249^.

v. 2024 *Pistocythereis bradyformis* (Ishizaki); Maekawa, fig. 182.64^285^.

**Type.** Female left valve (Holotype), IGPS 90312.

**Remarks.** Ishizaki^1^ illustrated an optical photograph of an external view of the holotype (pl. 9, fig. 17). Okubo^333^ combined *Pistocythereis bradyi* and *Pistocythereis bradyformis* as one species, *Pistocythereis bradyi*, and illustrated soft parts and SEM images. However, his SEM images are of *Pistocythereis bradyformis*, identifiable by its irregular reticulation, and a prominent carinal ridge running from the mid-anterior area to the postero-ventral area. The present reexamination confirms the holotype. Gou et al. ^221^ depicted this species from Pliocene borehole cores in the South China Sea, but it differs by having vertically oriented reticulation across the valve surface, a projected posterior margin, and a narrowly arched anterior margin. Hou et al.^20^ illustrated this species from Quaternary sediment in Jiangsu, China; showing similar differences: vertically oriented reticulation, a projected posterior margin, and a narrowly arched anterior margin. In Gou et al.^22^, Gou defined the genus *Pistocythereis*, designated *Echinocythereis bradyi* Ishizaki, 1968^1^ as they type species. This species features a crenulate median groove with a socket at the anterior end, an underdeveloped antero-marginal rim, and either concentrically arranged small round spines or delicate large mesh reticulation on the outer shell surface. The specimen depicted as *E. bradyformis* by Kim and Park^271^ differs from the type specimen with its tapering posterior margin, two parallel ridges from the central to the posterior area, and the absence of an eye tubercle. Kim and Park^271^ also illustrated *Ruggieria* sp. A and *R*. sp. B, which are juvenile specimens of *P. bradyformis*. Gou et al.^22^ mentioned that this genus has been identified in East Asia since the Pliocene. Ishizaki^222^ illustrated a specimen labeled as Gen. et sp. indet., which is the juvenile form of this species. Zheng^274^ found this species in quaternary sediment in Zhejiang, China, but the specimen is not *P. bradyformis*, as it features a long carinal ridge along the anterior to ventral margin, an acutely curved anterior margin, and lacks denticulation along the anterior and posterior margins. Lin and Zhu^273^ found this species in Laizhou Bay, Bohai Sea, but it differs from the type specimen with its acutely arched anterior margin, projected posterior margin, and absence of spines around the anterior and posterior margins. Dewi^87^ reported this species from a Holocene borehole core in Java Island, Indonesia, but it differs from the type specimen for its straight ventral carinal ridge, irregular reticulation in the anterior area, and acutely curved postero-ventral margin. *Pistocythereis bradyformis*, as illustrated by Cao^34^, includes *P. bradyi*. Hu and Tao^361^ depicted this species from recent sediment in Jurong, Singapore; however, it differs from the type specimen by having a straight ventral carinal ridge, a parallelogram lateral outline, and a sinuate ventral margin. Most of the specimens illustrated by Hu and Tao^17^ are not *P. brady* but *P. bradyformis*. The specimen figured by Hu and Tao^17^ (pl. 55, fig. 19) is a juvenile of the genus *Actinocythereis*. Another specimen illustrated by Hu and Tao^17^ (pl. 94, fig. 14) is misidentified and does not belong to the genus *Pistocythereis*. Two specimens (pl. 211, figs. 1, 19), also figured by Hu and Tao^17^, are not members of the genus *Pistocythereis* but are instead identified as *Keijella kloempritensis* (Kingma, 1948)^45^.  *Pistocythereis bradyi*, reported by Niiyama et al.^133^ from recent sediment of the Co To Islands, Northern Vietnam, differs from the type specimen by having a sinuate dorsal margin, a narrowly arched posterior margin, and a lack of two ventral carinal ridges.

**Occurrence:**

*Recent*: West coast of Malaysia; Malacca Straits; Sunda Shelf; Spratly Islands, South China Sea; Northern South China Sea; Hong Kong, China; Sekisei-sho, Okinawa, Japan; Shandong, North China; Kyushu, Shikoku, Japan; Seto Inland Sea, Central and Northern Honshu, Japan; and Sea of Japan side of Southwest Japan.

*Fossil*: Pliocene from Leizhou Peninsula; Late Pliocene from Okinawa, Japan; Plio-Pleistocene from Taiwan and Okinawa; Middle Pleistocene from Southwest and Central Honshu, Japan; Pleistocene from Taiwan and Okinawa; Late Pleistocene from Southwestern Honshu, Japan; Quaternary borehole cores from Hong Kong, the Bohai Sea; Leizhou Peninsula, China; Holocene from Tsushima Straits, Southwest and Central Honshu, Seto Inland Sea, Japan; Holocene from the Sea of Japan side of Southwest Honshu, Central and Northern Honshu, and Pacific side of Northeast Honshu, Japan.

Genus: *Trachyleberis* Brady, 1898^373^

Type Species: *Cythere scabrocuneata* Brady, 1880^268^

**43.** *Trachyleberis niitsumai* Ishizaki, 1971^3^

v*. 1971 *Trachyleberis niitsumai* Ishizaki, p. 93, pl. 1, fig. 5, pl. 4, figs. 15, 18, pl. 5, fig. 3, pl. 6, fig. 10, pl. 7, fig. 9^3^.

v. 1977 *Acanthocythereis* ? *niitsumai* (Ishizaki); Hanai et al., p. 50^8^.

v. 1978 *Trachyleberis* sp. Yajima, p. 398-399, pl. 49, figs. 1a, 1b^345^.

v.p. 1979 *Trachyleberis scabrocuneata* (Brady); Okubo, p.149-151, fig. 7c-e^333^.

non. 1981 *Trachyleberis niitsumai* Ishizaki; Gou et al., p.175, pl. 84, figs. 4-6^221^.

v. 1981 *Costa sinensis* Hu, p. 67-68, pl. 1, figs. 9, 10, text-fig. 5C, 5D^174^.

v. 1982 *Trachyleberis niitsumai* Ishizaki; Cai, p. 7, pl. 2, figs. 38, 39^21^.

v. 1982 *Trachyleberis scabrocuneata* (Brady); Hou et al., p.223-224, pl. 83, figs. 1-5, text-fig. 63^20^.

v.p. 1982 *Trachyleberis niitsumai* Ishizaki; Hou et al., p. 225, pl. 83, figs. 6-11, text-fig. 65^20^.

1982 *Acanthocythereis* *niitsumai* (Ishizaki); Yajima, p. 196^286^.

v. 1983 *Trachyleberis niitsumai* Ishizaki; Gou et al., p.82, pl. 15, fig. 29^22^.

v. 1983 *Trachyleberis costus* Hu, p. 150-151, pl. 1, figs. 17-19^175^.

v. 1983 *Trachyleberis* cf. *costus* Hu, p. 151, pl. 1, figs. 23, 24^175^.

v. 1983 *Trachyleberis scabrocuneata* (Brady); Ikeya, p. 2, figs. 1.1, 1.2^272^.

v. 1984 *Acanthocythereis* *sinensis* (Hu); Hu, p. 97, 98, pl. 7, figs. 3, 5-7, 12, text-fig. 31^176^.

v. 1985 *Trachyleberis scabrocuneata* (Brady); Huang, p. 53, pl. 1, fig. 8^338^.

v. 1985 *Acanthocythereis* *niitsumai* (Ishizaki); Wang, et al., pl. 11, fig. 14^374^.

v. 1985 *Acanthocythereis* *niitsumai* (Ishizaki); Wang and Zhao, pl. 7, fig. 19^287^.

v. 1985 *Acanthocythereis* *niitsumai* (Ishizaki); Zhao et al., pl. 19, fig. 13^358^.

v.p. 1986 *Acanthocythereis* *niitsumai* (Ishizaki); Hu., p.126, 128, pl. 4, figs. 16, 23, 24, 26, 27^23^.

v. 1987 *Trachyleberis niitsumai* Ishizaki; Zheng, p.201, pl. 6, fig. 5^25^.

v.p. 1988 *Trachyleberis niitsumai* Ishizaki; Ruan and Hao, p. 356, pl. 64, figs. 26, 27^28^.

v. 1988 *Acanthocythereis* *niitsumai* (Ishizaki); Wang, et al., p. 103, pl. 1, fig. 4^276^.

v. 1989 *Trachyleberis scabrocuneata* (Brady); Ruan, pl. 2, fig. 7^161^.

v. 1989 *Trachyleberis niitsumai* Ishizaki; Ruan, pl. 2, fig. 8^161^.

v. 1989 *Actinocythereis triangulata* (Guan); Ruan, pl. 2, fig. 9^161^.

v. 1990 *Trachyleberis niitsumai* Ishizaki; Lee, p.272-274, pl. 21, figs. 3-6^29^.

v. 1990 *Acanthocythereis* *niitsumai* (Ishizaki); Zhao and Wang, pl. 2, fig. 12^148^.

v. 1992 *Trachyleberis niitsumai* Ishizaki; Ikeya and Suzuki, p. 137, pl. 9, fig. 2^183^.

v. 1992 *Trachyleberis niitsumai* Ishizaki; Lee and Paik, p.153, pl. 4, fig. 2^31^.

non. 1992 *Trachyleberis niitsumai* Ishizaki; Huh and Paik, p.283, pl. 2, figs. 6, 7^375^.

non. 1992 *Trachyleberis niitsumai* Ishizaki; Huh and Paik, p.111, pl. 2, figs. 6, 7^214^.

v. 1993 *Trachyleberis niitsumai* Ishizaki; Kamiya and Nakagawa, p.129, pl. 4, figs. 5, 6^14^.

v. 1994 *Acanthocythereis* *niitsumai* (Ishizaki); Zheng et al., pl. 45, figs. 4, 5^32^.

v. 1998 *Trachyleberis niitsumai* Ishizaki; Irizuki et al., p.7, fig. 2.12^150^.

v. 1998 *Pistocythereis bradyformis* (Ishizaki); Yamane, p. 59, pl. 12, fig. 5^35^.

v. 2013 *Trachyleberis niitsumai*: Brandão et al., p. 372, 383, figs. 1, 3h, 5h, 5f^376^.

v. 1998 *Trachyleberis niitsumai* Ishizaki; Tanaka et al., p.91, pl. 1, fig. 23^257^.

v. 2002 *Trachyleberis ishizakii* Yasuhara et al.; Yasuhara et al., p. 635, fig. 3.4^228^.

v. 2003 *Trachyleberis niitsumai* Ishizaki; Yasuhara and Kumai, p. 78, pl. 3, fig. 9^282^.

2004 *Acanthocythereis* *niitsumai* (Ishizaki); Yamauchi, p. 73^38^.

v. 2006 *Trachyleberis scabrocuneata* (Brady); Yasuhara and Seto, p. 107, figs. 5e, 5f^243^.

v. 2008 *Trachyleberis niitsumai* Ishizaki; Hu and Tao, p. 264, pl. 26, fig. 21, pl. 46, fig. 13, pl. 69, fig. 3, pl. 104, figs. 8, 18, 20, pl. 115, fig. 12, pl. 161, fig. 16, text-fig. 161^17^.

2008 *Trachyleberis niitsumai* Ishizaki; Hu and Tao, pl. 17, fig. 1, pl. 46, fig. 10, pl. 160, fig. 11^17^.

non. 2008 *Trachyleberis niitsumai* Ishizaki; Hu and Tao, pl.15, fig. 20., pl. 26, figs. 16, 18, pl. 46, fig. 12, pl. 69, fig. 5, pl. 82, figs. 9, 12, 14, pl. 90, fig. 13, pl. 104, figs. 8, 9, 11, 14, pl. 115, figs. 7, 9, 10, pl. 129, fig. 10., pl. 160, figs. 14, 17, pl. 161, figs. 1, 11, 13, 14, pl. 186, figs. 1, 8^17^.

v. 2008 *Trachyleberis niitsumai* Ishizaki; Tanaka, p. 276, fig. 2l^349^.

v. 2009 *Trachyleberis* sp. Horikoshi et al., p. 154, fig. 5.13^264^.

v. 2009 *Trachyleberis ishizakii* Yasuhara et al.; Ozawa, p. 238, fig. 4.20^218^.

v. 2010 *Trachyleberis niitsumai* Ishizaki; Irizuki et al., p. 16, fig. 4.6^245^.

v. 2011 *Trachyleberis* sp. (Brady); Iwatani et al., p. 281, fig. 5.12^377^.

v. 2013 *Trachyleberis niitsumai* Ishizaki; Brandão et al., p. 376, figs. 1, 3h, 5h, 5f^376^.

v. 2014 *Trachyleberis niitsumai* Ishizaki; Masuma and Yamada, p. 6, fig. 4.22^41^.

v. 2015 *Trachyleberis niitsumai* Ishizaki; Irizuki et al., p. 153, fig. 4.5^245^.

v. 2017 *Trachyleberis* sp. Ishizaki; Tsukagoshi, p. 16, fig. 2I^201^.

v. 2018 *Trachyleberis niitsumai* Ishizaki; Irizuki et al., p. 643, fig. 5.12^157^.

v. 2019 *Trachyleberis niitsumai* Ishizaki; Tanaka et al., p. 12, fig. 4.20, p. 13, figs. 5.1, 5.2^203^.

v. 2021 *Trachyleberis niitsumai* Ishizaki Irizuki et al., p. 7, fig. 6.9^250^.

v. 2024 *Trachyleberis niitsumai* Ishizaki; Maekawa, fig. 182.70^285^.

**Type:** Male right valve (Holotype), IGPS 91705).

**Remarks:** Ishizaki^3^ provided an optical photograph of the external view (pl. 6, fig. 10) and an SEM image of the internal view (pl. 7, fig. 9) of the holotype. He also included a drawing of muscle scars of the female left valve, an optical photograph of the external view, and SEM images of the internal views (IGPS 91706, paratype, pl. 1, fig. 5, pl. 4, fig. 18, pl. 5, fig. 3), as well as an SEM image of the internal view of a female left valve (IGPS 91706). Additionally, he showed an optical photograph of a juvenile left valve (IGPS 91707, paratype, pl. 4, fig. 18). Okubo^333^ figured *Trachyleberis scabrocuneata* from the Seto Inland Sea, including *Trachyleberis niitsumai*, and illustrated the soft parts of *T. scabrocuneata*; however, it is difficult to determine if the soft parts are from *T. scabrocuneata* or *T. niitsumai*. The present reexamination confirms the holotype. Gou et al. ^221^ illustrated this species from the Early Miocene borehole cores from the South China Sea; however, it differs by having a sinuate ventral margin, a narrowly arched anterior margin, and a short cardinal ridge running from the eye tubercle. *Costa sinensis*, described by Hu^174^ from the Pleistocene Hengchun Limestone, was later included in *T. niitsumai* by Hu^23^. *Acanthocythereis sinensis* (Hu) was reported from the Plio-Pleistocene Tungshiao Formation, Taiwan, and was included in *T. niitsumai* by Hu^23^. *Trachyleberis costus*, described by Hu^175^ from the Plio-Pleistocene Maanshan Mudstone, was included in *T. niitsumai* by Hu^23^. *Trachyleberis niitsumai*, as figured by Gou et al.^22^, represents the juvenile of this species. *Tracyleberis niitsumai* identified by Huh and Paik^214, 375^ from the Middle Miocene of the lower unit of the Yeonil Group, South Korea, differs from the type species by having an upwardly projected caudal process, a prominent central tubercle, and fewer but more robust spines along the postero-ventral margin. Huh and Whatley^378^ designated their identified *Trachyleberis niitsumai* as *Trachyleneris praeniitsumai* Huh and Whatley^378^. *Trachyleberis ishizakii*, described by Yasuhara et al.^228^ from the Holocene borehole cores, was identified as *T. niitsumai* based on ornamentation. Many specimens (pl.15, fig. 20, pl. 69, fig. 5; pl. 82, figs. 9, 12; pl. 90, fig. 13; pl. 104, figs. 8, 9, 11, 14; pl. 115, figs. 7, 9, 10; pl. 129, fig. 10; pl. 160, figs. 14, 17; pl. 161, figs. 1, 11, 13, 14; pl. 186, figs. 1, 8) figured by Hu and Tao^17^ are not *T. niitsumai* but *T. scabrocuneata*. Ozawa^218^ pointed out that *Trachyleberis ishizakii* Yasuhara et al.^228^ is a juvenile specimen of *T. niitsumai*. *Trachyleberis* sp., as figured by Tsukagoshi^201^, is also a juvenile of this species.

**Occurrence:**

*Recent*: South China Sea; shelf of South China; coast of Hebei, China; Sekisei-sho, Okinawa, Japan; East China Sea, Yellow Sea, China; Tsushima Straits; Southwest and Northern Honshu, Seto Inland Sea, Japan.

*Fossil*: Pliocene from Leizhou Peninsula; Pliocene from Taiwan; Late Pliocene from Taiwan; Plio-Pleistocene from Taiwan; Plio-Pleistocene from Miyazaki, Kyushu, Japan; Pleistocene from Taiwan; Pleistocene from Cheju Island, Korea; Middle Pleistocene from Kyushu and Central Honshu, Japan; Late Pleistocene from Southern Taiwan; Late Pleistocene from Okinawa Trough, Southwest Honshu, Japan; Quaternary Jiangsu, Fujiang, China; Quaternary borehole cores from the Bohai Sea; Holocene from Southwest Honshu, Japan; Quaternary from Central Honshu, Japan; Holocene borehole cores from the Bohai Sea, China; Holocene borehole cores from Kyushu, Central Honshu, and Seto Inland Sea, Japan.

Genus: *Acanthocythereis* Howe, 1963^379^

Type Species: *Acanthocythereis araneosa* Howe, 1963^379^

**44.** *Acanthocythereis mutsuensis* Ishizaki, 1971^3^

v*. 1971 *Acanthocythereis mutsuensis* Ishizaki, p. 93-94, pl. 1, fig. 7, pl. 5, fig. 2, pl. 6, fig. 4^3^.

v. 1977 *Acanthocythereis* ? *mutsuensis* Ishizaki; Hanai et al., p. 50^8^.

non. 1985 *Acanthocythereis mutsuensis* Ishizaki; Wang, et al., p.344, pl. 12, fig. 16^374^.

non. 1990 *Acanthocythereis mutsuensis* Ishizaki; Lee, p.286-287, pl. 24, figs. 3, 4^29^.

non. 1992 *Acanthocythereis mutsuensis* Ishizaki; Huh and Paik, p.283, pl. 2, figs. 8, 9^375^.

non. 1992 *Acanthocythereis mutsuensis* Ishizaki; Huh and Paik, p.111, pl. 2, figs. 8, 9^214^.

non. 1996 *Acanthocythereis mutsuensis* Ishizaki; Ozawa, p.107, pl. 1, fig. 2^33^.

2006 *Acanthocythereis mutsuensis* Ishizaki; Schornikov and Zanina, p.216, table 2^15^.

2006 *Acanthocythereis mutsuensis* Ishizaki; Schornikov, p.47^16^.

non. 2008 *Acanthocythereis mutsuensis* Ishizaki; Hu and Tao, p.272-273, pl. 17, figs. 3-5, pl. 82, fig. 19, pl. 129, fig. 12, pl. 148, fig. 22, pl. 154, fig. 17, text-fig. 171^17^.

2008 *Acanthocythereis mutsuensis* Ishizaki; Hu and Tao, pl. 55, fig. 9^17^.

non. 2014 *Acanthocythereis mutsuensis* Ishizaki; Schornikov and Zanina, p.33, pl. 3, figs. 10, 11^156^.

**Type:** Female left valve (Holotype), IGPS 91708.

**Remarks:** Ishizaki^3^ illustrated the SEM of the holotype’s external view (pl. 5, fig. 2). He also presented a drawing of muscle scars of the left valve (IGPS 91709, paratype, pl. 1, fig. 7) and an SEM of the internal view of a female right valve (IGPS 91718). The present reexamination confirms the holotype. *Acanthocythereis mutsuensis*, reported from the Huanghai Sea (Yellow Sea) by Wang, et al.^374^, do not match the type specimen as it has a sinuate ventral margin, an acutely arched posterior margin, and an evenly arched anterior margin. The specimens illustrated by Lee^29^ from the Pleistocene Sogwipo Formation, Cheju Island, Korea, are not identified as this species because they have a postero-ventrally arched posterior margin, narrowly arched anterior margin, and no postero-ventral spines. Zhao and Wang^148^ reported this species from the shelf off China; however, it differs by having an acutely arched anterior margin, a narrowly arched posterior margin, and a sinuate ventral margin. Huh and Paik^214, 375^ identified *Acanthocythereis mutsuensis* from the Middle Miocene of the lower unit of the Yeonil Group, South Korea, but it differs from the type species with its reticulation, ventrally arched anterior margin, and postero-ventrally protruded posterior margin. Huh and Whatley^378^ designated their identified *Acanthocythereis mutsuensis* as *Acanthocythereis koreana* Huh and Whatley^378^. Ozawa^33^ figured this species; however, it differs from the type species by having a postero-ventrally arched posterior margin, a widely concave ventral margin, and an antero-ventral arched anterior margin. The specimens (pl. 3, figs. 10, 11; pl. 82, fig. 19; pl. 148, fig. 22) identified as *Acanthocythereis mutsuensis* by Hu and Tao^17^ are *Acanthocythereis munechikai* Ishizaki, 1981, from recent sediment of the East China Sea^143^. Two specimens (pl. 129, fig. 12; pl. 154, fig. 17) figured by Hu and Tao^17^ belong to the genus *Trachyleberis*. Schornikov and Zanina^156^ reported this species from Peter the Great Bay, Far East, Russia; however, it differs from the type specimen by having an antero-ventral directed anterior margin, a sinuated ventral margin, and a narrowly arched posterior margin.

**Occurrence:**

*Recent*: Aomori Bay, Northern Honshu, Japan.

*Fossil*: unknown.

Genus: *Pacambocythere* Malz, 1982^380^

Type Species: *Pacambocythere cytherelloidae* Malz, 1982^380^

**45.** *Pacambocythere japonica* (Ishizaki, 1968)^1^

v*. 1968 *Ambocythere japonica* Ishizaki, p. 39-40, pl. 2, fig. 9, pl. 8, figs. 15-17^1^.

non.1976 *Cytherelloidea* ? *japonica* (Ishizaki); Holden, F. 38, pl. 6, fig. 12, pl. 16, fig. 20^251^.

v. 1977 *Ambocythere japonica* Ishizaki; Hanai et al., p. 53^8^.

1982 *Ambocythere japonica* Ishizaki; Yajima, p. 204^286^.

v. 1986 *Pacambocythere japonica* (Ishizaki); Hu, p. 135, 137, pl. 17, figs. 7, 8, 10, 11, text-fig. 6B^23^.

v. 1996 *Ambocythere japonica* Ishizaki; Ozawa, p. 107, pl. 1, fig. 5^33^.

v. 1998 *Ambocythere japonica* Ishizaki; Yamane, p. 37, pl. 1, fig. 1^35^.

v. 2001 *Ambocythere japonica* Ishizaki; Kamiya et al., 93, fig. 14.6^36^.

2012 *Pacambocythere japonica* (Ishizaki); Tanaka et al., p. 17-18^40^.

2012 *Pacambocythere japonica* (Ishizaki); Tanaka et al., p. 117, pl. 2, fig. 9^199^.

v. 2014 *Ambocythere japonica* Ishizaki; Masuma and Yamada, p. 5, fig. 3.1^41^.

**Type:** Right valve (Holotype), IGPS 90307.

**Remarks:** Ishizaki^1^ illustrated a line drawing of the internal view (pl. 2, fig. 9) and an optical photograph of the external view (pl. 8, fig. 17) of the holotype. He also showed optical photographs of the external views of a right valve (IGPS 90308, paratype, pl. 8, fig. 15) and a left valve (IGPS 90309, pl. 8, fig. 16). The present reexamination confirms the holotype. Malz^380^ established a new genus, *Pacambocythere*, based on the internal shell characteristics, which includes a wider fused zone, a narrow strip of inner lamella, and strong teeth. He also included *Ambocythere japonica* Ishizaki^1^ in this genus and pointed out that the genus *Pacambocythere* is distributed only in the Pacific Ocean. We follow the view of Malz^380^. Horden^251^ identified *Cytehrelloidea* ? *japonica*^1^ from the Late Cenozoic drilling core samples at Midway Island; however, the figured specimen differs from the type specimen in that it has a postero-ventral tubercle, a concaved postero-dorsal margin, and an anteriorly directed antero-dorsal sulcus.

**Occurrence:**

*Recent*: Shikoku, Seto Inland Sea, and Sea of Japan side, Central Honshu, Japan.

*Fossil*: Plio-Pleistocene from Southwest Honshu, Japan; Pleistocene from Taiwan; Holocene from Central Honshu, Japan.

Genus: *Doratocsythere* McKenzie, 1967^381^

Type Species: *Doratocythere foveata* McKenzie, 1967^381^

**46.** *Doratocythere tomokoae* (Ishizaki, 1968)^1^

v*. 1968 *Leguminocythereis tomokoae* Ishizaki, p. 26, pl. 1, fig. 11, pl. 5, figs. 1, 2, 17^1^.

v. 1977 *Proteoconcha tomokoae* (Ishizaki); Hanai et al., p. 49^8^.

v.p. 1981 *Campylocythereis tomokoae* (Ishizaki); Gou et al., p.163, pl. 79, fig. 17^221^.

v. 1981 *Basslerites taiwanensis* Hu and Yeh; Hu, p. 69, pl. 1, figs. 5, 8, 11, 12^174^.

non. 1981 *Campylocythereis tomokoae* (Ishizaki); Gou et al., p.163, pl. 79, fig. 18^221^.

v. 1982 *Australimoosella tomokoae* (Ishizaki); Yajima, p. 206, pl. 12, figs. 18, 19, text-fig. 15-4^286^.

vp. 1983 *Campylocythereis tomokoae* (Ishizaki); Gou et al., p.55-56, pl. 12, figs. 6, 9, text-fig. 24^22^.

v. 1983 *Basslerites taiwanensis* Hu and Yeh; Hu, p. 151-152, pl. 1, figs. 13, 14, 16, text-fig. 1^175^.

v. 1984 *Taiwanocythere taiwanensis* (Hu and Yeh); Hu, p. 104, pl. 6, figs. 19-22^176^.

v. 1985 *Moosella tomokoae* (Ishizaki); Ikeya et al., pl. 3, fig. 12^12^.

v. 1985 *Campylocythereis tomokoae* (Ishizaki); Zhao, pl. 2, fig. 12^160^.

v. 1986 *Australimoosella tomokoae* (Ishizaki); Hu, p. 169, pl. 24, figs. 14, 16^23^.

non. 1986 *Campylocythereis tomokoae* (Ishizaki); Zheng, pl. 2, figs. 11-13^274^.

non. 1986 *Campylocythereis tomokoae* (Ishizaki); Lin and Zhu, p. 97, pl. 2, fig. 2^273^.

non. 1988 *Campytocythereis tomokoae* Ishizaki; Wang, et al., p.103, pl. 1, fig. 9^276^.

v.1989 “*Campylocythereis” tomokoae* (Ishizaki); Gou and Gong in Gou, pl. 166, figs. 4, 5^347^.

non.1989 *Campylocythereis tomokoae* (Ishizaki); Ruan, pl. 1, figs. 16, 17^161^.

v. 1993 *Australimoosella tomokoae* (Ishizaki); Kamiya and Nakagawa, p. 129, pl. 4, fig. 4^14^.

v. 1994 *Moosella tomokoae* (Ishizaki); Zheng et al., pl. 51, fig. 17-19^32^.

v. 1998 *Moosella tomokoae* (Ishizaki); Tanaka et al., p. 91, pl. 1, fig. 22^257^.

v. 1998 *Moosella tomokoae* (Ishizaki); Yamane, p. 49, pl. 7, fig. 5^35^.

v. 2001 *Australimoosella tomokoae* (Ishizaki); Kamiya et al., 101, fig. 18.8^36^.

2004 *Moosella tomokoae* (Ishizaki); Yamauchi, p. 73^38^.

v. 2005 *Australimoosella tomokoae* (Ishizaki); Ishida and Takayasu, p.76, fig. 2.2^216^.

2006 *Doratocythere tomokoae* (Ishizaki); Schornikov, p.48^16^.

2006 *Doratocythere tomokoae* (Ishizaki); Schornikov and Zanina, p.216, table 2^15^.

v. 2008 *Australimoosella tomokoae* (Ishizaki); Hu and Tao, p. 347-348, pl. 24, fig. 12, text-fig. 206^17^.

2008 *Australimoosella tomokoae* (Ishizaki); Hu and Tao, pl. 9, fig. 20^17^.

2012 *Moosella tomokoae* (Ishizaki); Tanaka et al., p. 18, pl. 3, fig. 10^40^.

**Type:** Right valve (Holotype), IGPS 90245.

**Remarks:** Ishizaki^1^ illustrated a line drawing of the internal view (pl. 1, fig. 11) and an optical photograph of the external view (pl. 5, fig. 1) of the holotype. He also showed optical photographs of the external views of a left valve (IGPS 90244, paratype, pl. 5, fig. 2) and a juvenile left valve (IGPS 90246, pl. 5, fig. 17). The present reexamination confirms the holotype. Gou et al. ^221^ figured two specimens of this species from the Pliocene borehole cores in the South China Sea; however, one of the specimens (pl. 79, fig. 18) is not identified as *Doratocythere tomokoae* as it has a broadly arched anterior margin, a postero-ventral projection, and no caudal process. *Basslerites taiwanensis* Hu and Yeh, as figured by Hu^174, 175^, are included as synonyms in this species by Hu^23^. Lin and Zhu^273^ reported this species from surface sediment in Laizhou Bay, Bohai Sea; however, it differs from the type specimen by having a sinuate dorsal margin, a broadly arched ventral half of the posterior margin, and a vertical carinal ridge in the posterior area. Wang, et al.^276^ reported this species from Quaternary borehole cores in the Bohai Sea, China, but it differs by having reticulation, a straight carinal ridge running from the mid-anterior to the mid-posterior area, and three carinal ridges along the posterior margin. Zheng^274^ figured this species from Quaternary core samples in Zhejiang, China; however, it differs by having an upwardly pointed posterior margin, a flat postero-ventral area, and a widely arched anterior margin. Ruan^161^ reported this species from the coast of Hebei, but it differs by having a sinuate dorsal margin, a short anterior margin, and a flat area near the ventral margin.

**Occurrence:**

*Recent*: South China Sea, Yellow Sea, China; Shikoku, Southwest and Central Honshu, Japan.

*Fossil*: Pliocene from Leizhou Peninsula; Late Pliocene from Taiwan; Late Pliocene-Early Pleistocene from Taiwan; Pleistocene from Taiwan; Late Pleistocene from Central Japan; Quaternary borehole cores in East China Sea; Holocene from Southwest Honshu, Sea of Japan side in Southwest Honshu, Japan.

Genus: *Sinoleberis* Hu, 1979^172^

Type Species: *Sinoleberis taiwanica* Hu, 1977^382^

**47.** *Sinoleberis tosaensis* (Ishizaki, 1968)^1^

v*. 1968 *Trachyleberis tosaensis* Ishizaki, p. 38-39, pl. 2, figs. 7, 8, pl. 8, figs. 5, 6^1^.

v. 1977 *Trachyleberis* ? *tosaensis* Ishizaki; Hanai et al., p. 49-50^8^.

1982 *Trachyleberis* *tosaensis* Ishizaki; Hou et al., p. 224-225, pl.83, figs. 12-14^20^.

1982 *Trachyleberis* *tosaensis* Ishizaki; Cai., p. 7, pl. 2, fig. 42^21^.

v. 1982 *Sinoleberis tosaensis* (Ishizaki); Malz and Ikeya, p.417-418, pl. 1, figs. 1-3^383^.

v. 1982 *Trachyleberis* ? *tosaensis* Ishizaki; Yajima, p. 194-195, pl. 12, figs. 8, 9, 11, text-fig. 15-3^286^.

v. 1984 *Sinoleberis taiwanica*; Hu, p.129, pl. 7, figs. 22, 24-26^176^.

v. 1986 *Sinoleberis tosaensis* (Ishizaki); Hu, p.129, pl. 24, fig. 10^23^.

non. 1986 *Trachyleberis* *tosaensis* Ishizaki; Lin and Zhu, p. 97, pl. 2, fig. 6^273^.

v. 1990 *Sinoleberis tosaensis* (Ishizaki); Lee, p.291-292, pl. 24, figs. 8, 9^29^.

v. 1992 *Sinoleberis tosaensis* (Ishizaki); Lee and Paik, p.153, pl. 4, fig. 10^31^.

non. 1992 *Sinoleberis tosaensis* (Ishizaki); Huh and Paik, p.283, pl. 2, fig. 11^375^.

non. 1992 *Sinoleberis tosaensis* (Ishizaki); Huh and Paik, p.111, pl. 2, fig. 11^214^.

non. 1993 *Sinoleberis tosaensis* (Ishizaki); Kamiya and Nakagawa, p.129, pl. 4, fig. 9^14^.

v. 1995 *Sinoleberis tosaensis* (Ishizaki); Ozawa et al., p.31, pl. 1, fig. 16^204^.

v. 2001 *Sinoleberis tosaensis* (Ishizaki); Nakao et al., p. 141, fig. 11.5^258^.

v. 2008 *Sinoleberis tosaensis* (Ishizaki); Hu and Tao, p. 278-279, pl. 20, fig. 10, pl. 27, fig. 20, pl. 69, figs. 9, 14, 15, pl. 187, figs. 12-14, 18, pl. 224, figs. 7, 8, 10, 11, 13, text-fig. 175^17^.

2008 *Sinoleberis tosaensis* (Ishizaki); Hu and Tao, pl. 11, figs. 13, 15, pl. 82, fig. 16, pl. 91, fig. 5, pl. 213, fig. 8^17^.

non. 2008 *Sinoleberis tosaensis* (Ishizaki); Hu and Tao, pl. 96, fig. 7, pl. 118, fig. 12, pl. 129, fig. 15^17^.

v. 2009 *Sinoleberis tosaensis* (Ishizaki); Ozawa, p. 238, fig. 4.18^218^.

v. 2012 *Sinoleberis tosaensis* (Ishizaki); Tanaka et al., p. 18, pl. 3, fig. 7^40^.

**Type:** Right valve (Holotype), IGPS 90304.

**Remarks:** Ishizaki^1^ illustrated a line drawing of the internal view (pl. 2, fig. 8) and an optical photograph of the external view (pl. 8, fig. 5) of the holotype. He also provided a line drawing (pl. 2, fig. 7) and an optical photograph of the external view (pl. 8, fig. 6) of a left valve (IGPS 90305, paratype). The present reexamination confirms the holotype. Lin and Zhu^273^ reported this species from Laizhou Bay, Bohai Sea; however, it differs from the type specimen by having an acute anterior margin, lack of spines at the postero-ventral margin, and a straight dorsal margin. *Sinoleberis tosaensis*, identified by Huh and Paik^214, 375^ from the Middle Miocene lower unit of the Yeonil Group, South Korea, differs from the type species in having reticulation, a straight dorsal margin, and an arched posterior margin. Kamiya and Nakagawa^14^ illustrated and identified this species from the Holocene Takahama shell bed in Fukui Prefecture Southwest Honshu, Japan. However, it differs by having a widely arched, flat postero-ventral area, no posterior spines, and a prominent subcentral tubercle. Two specimens (pl. 96, fig. 7; pl. 118, fig. 12; pl. 129, fig. 15) figured by Hu and Tao^17^ are not *S. tosaensis*. These specimens feature undivided anterior marginal carinas, a strong short carina at the postero-dorsal area of the eye tubercle, and numerous short, undulated vertical carinas running from the dorsal area to the central area.

**Occurrence:**

*Recent*: Penghu Islands, Western Taiwan; Northeast Taiwan; Shikoku, Southwest and Central Honshu, Japan.

*Fossil*: Pliocene from Southwest Taiwan; Late Pliocene from Northwestern Taiwan, Central Japan; Plio-Pleistocene from Taiwan; Pleistocene from Taiwan; Pleistocene from Taiwan; Cheju Island, Korea; Middle to Late Pleistocene from Central Honshu, Japan.

**References**

1. Ishizaki, K. Ostracodes from Uranouchi Bay, Kochi Prefecture, Japan. *Sci. Rep. Tohoku Univ., Sendai, Second Ser. (Geol.)* **40**, 1–45 (1968).
2. Ishizaki, K. Ostracodes from Shinjiko and Nakanoumi, Shimane Prefecture, western Honshu, Japan. *Sci. Rep. Tohoku Univ., Sendai, Second Ser. (Geol.)* **41**,197–224 (1969).
3. Ishizaki, K. Ostracodes from Aomori Bay, Aomori Prefecture, northeast Honshu, Japan. *Sci. Rep. Tohoku Univ., Sendai, Second Ser. (Geol.)* **43**, 59–97 (1971).
4. Latreille, P. A. Histoire naturelle, générale et particulière des Crustacés et des Insectes. Hist. Cypris Cytherée 8, 232–254. https://doi.org/10.5962/bhl.title.15764 (1802).
5. Sars, G. O. Oversigt af Norges marine Ostracoder.V*idensk Selsk Christiania Forh* **1865**, 1–130 (1866).
6. Alexander, C. I. Ostracoda of the Cretaceous of North Texas. *Univ. Texas Bull*. **2907**, 1–137 (1929).
7. Jones, T. R. A monograph of the Entomostraca of the Cretaceous Formation of England. *Annu. Vol. Palaeontol. Soc*. **3**, 1–40. https://doi.org/10.5962/bhl.title.46365 (1849).
8. Hanai, T., Ikeya, N., Ishizaki, K., Sekiguchi, Y. & Yajima, M. Checklist of Ostracoda from Japan and its adjacent seas. *Univ. Mus., Univ. Tokyo Bull*. **12**, 1–119 (1977).
9. Yamaguchi, S. & Endo, K. Molecular phylogeny of Ostracoda (Crustacea) inferred from 18S ribosomal DNA sequences: implication for its origin and diversification. *Mar. Biol*. **143**, 23–38 (2003).
10. Maddocks, R. F. Revision of recent Bairdiidae (Ostracoda). *U.S. Natl. Mus. Bull*. **296**, 1–126. https://doi.org/10.5479/si.03629236.295.1 (1969).
11. Hartmann, G. Zur Kenntnis der Ostracoden des Roten Meeres. *Kiel Meeresforsch.* **20**, 3–127 (1964).
12. Ikeya, N., Okubo, I., Kitazato, H. & Ueda, H. Shizuoka (Pleistocene and living Ostracoda, shallow marine, brackish and freshwater). In *Guidebook of Excursions for the 9th International Symposium on Ostracoda* (ed. Organizing Committee 9th International Symposium on Ostracoda) 1–32 (Shizuoka University Press, 1985).
13. Ishizaki, K. & Matoba, Y. Akita Early Pleistocene cold, shallow water Ostracoda. In *Guidebook of Excursions for the 9th International Symposium on Ostracoda* (ed. Organizing Committee 9th International Symposium on Ostracoda) 1–29 (Shizuoka University Press, 1985).
14. Kamiya, T. & Nakagawa, T. Ostracode fossil assemblages in the Holocene shell bed found in Takahama-Cho, Fukui Prefecture, Central Japan. Bull. Fukui Nat. Hist. Mus. **1**, 115–133 (1993).
15. Schornikov E. I. & Zanina, M. A. Class Ostracoda Latreille, 1802. In Far Eastern marine biosphere reserve (ed. Tyurin, A.N.) 211–222, 458–465 (Dalnauka, 2006).
16. Schornikov, E. I. Checklist of the ostracod (Crustacea) fauna of Peter the Great Bay, Sea of Japan. *Zootaxa* **1294**, 29–59 (2006).
17. Hu, C. & Tao, H. Studies on the Ostracod Fauna of Taiwan and Its Adjacent Seas. *Natl. Taiwan Mus. Spec. Publ. Ser.*, **13** (parts 1 and 2), 1–910 (2008).
18. Hornibrook, N. Tertiary and recent marine Ostracoda of New Zealand, their origin affinities and distribution. N.Z. Geol. Surv., Paleontol. Bull. **18**, 1–82 (1952).
19. Schornikov, E. I. Ostracods Bythocytheridae of the Far Eastern Seas. (U.S.S.R. Acad. Sci., Far Eastern Acad. Cent. Inst. Mar. Biol., Publ. House Nauka, 1981).
20. Hou, Y., Chen, T.-C., Yang, H.-R., Ho, J.-D., Zhou, Q.-C. & Tian, M.-Q. Cretaceous-Quaternary ostracode fauna from Jiangsu. (Geological Publishing House, 1982).
21. Cai, H. Distribution of Ostracoda in the northeastern waters of the South China Sea. *Trop. Oceanol.* **1**, 42–57 (1982).
22. Gou, Y., Zheng, S. & Huang, B. Pliocene ostracode fauna of Leizhou Peninsula and Northern Hainan Island, Guangdong province. (Science Press 162, 1983)
23. Hu, C. The ostracodes from the Tungshao Formation (Pleistocene), west coast of Miaoli District, Taiwan. *J. Taiwan Mus.* **39**, 99–173 (1986).
24. Cai, H. & Chen, M. An approach to the distribution and environmental characteristics of microfaunas in surface sediments in the South China Sea. *Acta Micropalaeontol. Sin.* **4**, 73–86 (1987).
25. Zheng, S. Quaternary Ostracoda fauna from coastal deposits along the coast of Fujian. *Mem. Nanjing. Inst. Geol. & Palaeontol., Acad. Sin.* **23**, 189–207 (1987).
26. Cai, H. Ostracoda in sediments from the north continental shelf of South China Sea. *Trop. Oceanol.* 7, 19–27 (1988).
27. Ikeya, N. & Shiozaki, M. Holocene Ostracoda from the Dempukuji-ura Site, Kurihama, Yokosuka City. Annu. Rep. Yokosuka City Cult. Assets. Invest. **16**, 131–140 (1988).
28. Ruan, P. & Hao, Y. 2. Ostracoda. In Quaternary microbiotas in the Okinawa Trough and their geological significance (ed. Research Party of Marine Geology, Ministry of Geology and Mineral Resources, Chinese University of Geosciences) 227–395 (Geological Publishing House, 1988).
29. Lee, E. Pleistocene Ostracoda from the marine sedimentary strata of the Cheju Island, Korea. (Ph D Thesis of Department of Geology Graduate School Korea University,1990).
30. Cai, H. Ostracoda from the Nansha Islands and the adjacent sea areas. In *Quaternary biological groups of the Nansha Islands* (ed. The Multidisciplinary Oceanographic Expedition Team of Academia Sinica to The Nansha Islands) 82–128 (Zhongshan University Publishing House, 1991).
31. Lee, E. & Paik, K. Late Cenozoic ostracod fauna and paleoenvironments of the marine sedimentary strata in the Cheju Island, Korea. *Paleontol. Soc. Korea, Spec. Publ.* **1**, 121–153 (1992).
32. Zheng, S. et al. A comprehensive ecological and palaeoecological study of the sedimentary organisms in the northern and eastern areas of the South China Sea (Hubei Science and Technology Publishing, 1994).
33. Ozawa, H. Ostracode fossils from the late Pliocene to Early Pleistocene Omma Formation in the Hokuriku district, central Japan. *Sci. Rep. Kanazawa Univ.* **41**, 77–115 (1996).
34. Cao, M. Ostracods from Quaternary Hang Hau Formation Lei Yue Mun, Hong Kong. In *Paleontology and stratigraphy in Hong Kong (lower volume)* (eds. Li, Z., et al.) 171–183 (Science Press, 1998).
35. Yamane, K. Recent ostracode assemblages from Hiuchi-nada Bay, Seto Inland Sea of Japan. *Bull. Ehime Prefect. Sci. Mus.* **3**, 19−59 (1998).
36. Kamiya, T., Ozawa, H. *&* Obata, M. Quaternary and Recent marine Ostracoda in Hokuriku district, the Japan Sea coast. In Guidebook of Excursions, 14th International Symposium on Ostracoda (ed. The Organising Committee of ISO 2001) 73–106 (Shizuoka University Press, 2001).
37. Irizuki, T. Fossil Ostracoda from the lower Pleistocene Masuda Formation, Tanega-shima Island, southern Japan. Geosci. Rep. Shimane Univ. **23**, 65–77 (2004).
38. Yamauchi, T. A checklist of published crustacean species from brackish lakes, Shinjiko and Nakaumi, Japan. *Laguna* **11**, 69–86 (2004).
39. Irizuki, T., Takata, H. *&* Ishida, K. Recent Ostracoda from Urauchi Bay, Kamikoshiki-jima Island, Kagoshima Prefecture, southwestern Japan. Laguna **13**, 13–28 (2006).
40. Tanaka, G., Zhou, B., Ikeya, N. *&* Hasegawa, Y. Recent ostracod assemblages from Suruga Bay, Central Japan. Bull. Gunma Mus. Nat. Hist. **16**, 1–30 (2012).
41. Masuma, T. & Yamada, K. Recent ostracode distributions in Kumihama Bay (brackish lake) in the northern part of Kyoto Prefecture, central Japan. *Laguna* **21**, 1–14 (2014).
42. Yamada, K., Masuda, T. & Seto, K. Paleoenvironmental changes in Lake Nakaumi during the last 1,700 years on the basis of ostracode assemblages. *Quat. Res.* **45**, 53–68 (2015).
43. Hu, C. Studies on ostracodes from the Pleistocene Toukoshan Formation in the Miaori area, Taiwan. *Proc. Geol. Soc. China* **20**, 80–107 (1977).
44. Hanai, T. Studies on the Ostracoda from Japan, I. Subfamily Leptocytherinae, new subfamily. *J. Fac. Sci., Univ. Tokyo, Sec. 2* **10**, 431–468 (1957).
45. Kingma, J. T. Contributions to the knowledge of the Young-Caenozoic Ostracoda from the Malayan region. (Kemink, 1948).
46. Howe, H. V. Handbook of ostracod taxonomy. (Louisiana State University Press, 1955).
47. Morkhoven, van F. P. C. M. Post-Paleozoic Ostracoda. Their morphology, taxonomy and economic use. (Elsevier, 1963).
48. Morales, G. A. Ecology, distribution, and taxonomy of recent Ostracoda of the laguna de Terminos, Campeche, Mexico. *Bol. Inst. Geol., Univ. Nac. Autonoma de México* **81**, 1–103 (1966).
49. Guha, D. K. Observation on the Cenozoic and some Mesozoic Ostracoda of India. *Publ. Cent. Adv. Study Geol., Punjab Univ.* **7**, 205–212 (1970).
50. Bate, R. H. The distribution of Recent Ostracoda in the Abu Dhabi Lagoon, Persian Gulf. In *Paléoécologie des Ostracodes Colloque Pau (1970)* (ed. Oertli, H. J.) 239–256 (Bull. Cent. Rech., Pau-SNPA, 1971).
51. Teeter, J. W. Geographic distribution and dispersal of some recent shallow-water marine Ostracoda. Ohio J. Sci. **73**, 46–54 (1973).
52. Hartmann, G. Zur Kenntnis des Eulitorals der afrikanischen Westküste zwischen Angola und Kap der Guten Hoffnung und der afrikanischen Ostküste von Südafrika und Mocambique unter besonderer Berücksichtigung der Polychaeten und Ostracoden. *Mitt. Hambg. Zool. Mus. Inst.* **69**, 229–520 (1974).
53. Jain, S. P. Holocene Ostracoda from the Chilka Lake, Orissa. In *Proceedings of the VI Indian Colloquium on Micropalaeontology and Stratigraphy* (ed. Srinivasan, M. S.) 126–134 (Department of Geology, Banaras Hindu University, 1976).
54. Bonaduce, G., Masoli, M. & Pugliese, N. Ostracoda from the Gulf of Aqaba (Red Sea). *Pubbl. Della Stazione Zool. Napoli* **40**, 372–428 (1976).
55. Paik, K. Regionale Untersuchungen zur Verteilung der Ostracoden im Persischen Golf und im Golf von Oman. *Meteor. Forschungsergebnise, Deutsche Forschungsge-meinschaft., Reihe. C Geol. Geophys., Gebrüder. Boträger.* **28**, 37–76 (1977).
56. Hartmann-Schröder, G. & Hartmann, G. Zur Kenntnis des Eulitorals der australischen Küsten unter besonderer Berücksichtigung der Polychaeten und Ostracoden. Teil 1: Die Ostracoden der Ordnung Podocopida G. W. Müller,1894 der tropisch–subtropischen Westküste Australiens (zwischen Derby im Norden und Penh im Süden). *Mitt. Hambg. Zool. Mus. Inst.* **75**, 63–219 (1978).
57. Jain, S. P. Recent Ostracoda from Mandvi Beach, west coast of India. *Bull. Indian Geologists’ Association* **11**, 89–139 (1978).
58. Annapurna, C. & Rama Sarma, D. V. Occurrence of a new podocopan ostracod *Tanella vasishta* in the Vasishta Godavari Estuary, east coast of India. Curr. Sci. 48, 42–43 (1979).
59. Garbett, E. C. & Maddocks, R. F. Zoogeography of Holocene cytheracean ostracodes in the bays of Texas. *J. Paleontol.* **53**, 841–919 (1979).
60. Keij, A. J. Brief review of the type species of genera from the Kingma collection. In Proceedings of the VII International Symposium on Ostracodes (ed. Krstic, N.) 59–62 (Beograd, 1979).
61. Hartmann, G. Zur Kenntnis des Eulitorals der australischen Küsten unter besonderer Berücksichtigung der Polychaeten und Ostracoden. Teil 5. Die Ostracoden der Ordnung Podocopida G. W. Müller 1894 der warmtemperierten und subtropisch–tropischen Küstenabschnitte der süd–und Südostküste Australiens (Zwischen Ceduna im Westen und Lakes Entrance im Osten). *Mitt. Hambg. Zool. Mus. Inst.* **77**, 111–204 (1980).
62. Hartmann, G. Zur Kenntnis des Eulitorals der australischen Küsten unter besonderer Berücksichtigung der Polychaeten und Ostracoden. Teil 7. Die Ostracoden der Ordnung Podocopida G.W. Müller, 1894 der subtropisch–tropischen Ostküste Australiens (zwischen Eden im Süden und Heron–Island im Norden). *Mitt. Hambg. Zool. Mus. Inst.* **78**, 97–149 (1981).
63. Al-Abdul-Razzaq, S. K., Shublaq, W. & Al-Sheikh, Z. Ostracode distribution and ecology of Sulaibikhat Bay. *Kuwait. Mar. Geol.* **47**, 57–75 (1982).
64. McKenzie, K. G. & Pickett, J. W. Environmental interpretations of late Pleistocene ostracode assemblages from the Richmond River Valley, New South Wales. *Proc. R. Soc. Victoria* **96**, 227–242 (1984).
65. Li, S. Distribution of the ostracod thanatocoenoses in the Pearl River mouth area. *Trop. Oceanol.* **4**, 43–52 (1985).
66. Dias-Brito, D., Moura, J. A. & Wurdig, N. Relationships between ecological models based on ostracods and foraminifers from Sepetiba Bay (Rio de Janeiro–Brazil). In *Evolutionary biology of Ostracoda: Its fundamentals and applications* (eds. Hanai, T., Ikeya, N. & Ishizaki, K.) 467–484 (Kodansha, 1988).
67. Whatley, R. & Zhao, Q. Recent Ostracoda of the Malacca Straits Part II (Continuation). *Rev. Esp. Micropaleontol.* **20**, 5–37 (1988).
68. Zhao, Q. & Whatley, R. Distribution of ostracod assemblages in bottom sediments of the Malacca Straits area. J. Tongji Univ. **16**, 159–168 (1988).
69. Howe, H. V. & McKenzie, K. G. Recent marine Ostracoda (Crustacea) from Darwin and north-western Australia. *Northern Territory Mus. Arts Sci., Monogr. Ser.* **3**, 1–50 (1989).
70. Whatley, R. & Keeler, N. Recent Ostracoda from Reunion Island, southwestern Indian Ocean. *Rev. Micropaléontol.* **3**, 63–84 (1989).
71. Gou, Y. Recent Ostracoda from Hainan Island, South China Sea. *Cour. Forsch. Senckenberg.* **123**, 19–35 (1990).
72. Ruan, P. Distribution of recent Ostracoda from the coastal zone of Gaode and Weizhou Island, Zhuang autonomous region of Guangxi. *Acta Oceanol. Sin.* **9**, 119–134 (1990).
73. Sreenivas, K., Raju, B. N. & Reddi, K. R. Ostracoda in the estuarine sediments, Pulicat Lake Estuary, east coast, India. *J. Geol. Soc. India* **37**, 492–499 (1991).
74. Mostafawi, N. Rezente Ostracoden aus dem mitteren Sunda-Sxhelf, zwischen der Malaiischen Halbinsel und Borneo. *Senckenberg. lethaea* **72**, 129–168 (1992).
75. Jellinek, T. Zur Ökologie und Systematik rezenter Ostracoden aus dem Bereich des ke nianischen Barriere–Riffs. *Senckenberg. lethaea* **73**, 83–225 (1993).
76. Witte, L. Taxonomy and biogeography of West African beach Ostracods. *K. Ned. Akd. Wet., Afd. Nat., Eerste. Reeks.* **39**, 1–84 (1993).
77. Yassini, I., Jones, B. G. & Jones, M. R. Ostracods from the Gulf of Carpentaria, northeastern Australia. *Senckenberg. lethaea* **73**, 375–406 (1993).
78. Coimbra, J. C., Carreño, A. I. & Ferron, F. A. Holocene Podocopida Ostracoda from Sepetiba Bay, Brazil – some dominant taxa. *Pesqui. Geociênc.* **21**, 90–99 (1994).
79. Vaidya, A. S. & Mannikeri, M. S. Faunal affinity and zoogeography of Recent marine Ostracoda from Karwar, west coast of India. Curr. Sci. **67**, 735–738 (1994).
80. Babinot, J. F. & Kouyoumontzakis, G. Associations d'ostracodes d'un environnement récifal envasé: Le lagon de l'ile de Mayotte (Archipel des Comores, Océan Indien occidental). *Geobios* **28**, 17–38 (1995).
81. Bell, K. N., Neil, J. V. & Burn, R. Recent foraminiferal, ostracodal and molluscan changes in a short core from Corner Island, Victoria. *Vic. Naturalist* **112**, 72–78 (1995).
82. Shyam Sunder, V. V., Varma, K. U. & Naidu, T. Y. Recent Ostracoda of the Goguleru Creek, east coast of India. *J. Geol. Soc. India* **45**, 471–481 (1995).
83. Yassini, I. & Jones, B. G. Recent Foraminifera and Ostracoda from estuarine and shelf environments on the southern coast of Australia. (The University of Wollongong Press, 1995).
84. Babinot, J. F. & Degaugue-Michalski, F. Lagoonal to reefal ostracod assemblages from Holocene and Recent deposits, Chesterfield Islands and northern New Caledonia (southwestern Pacific). *Micropaleontology* **42**, 351–362 (1996).
85. Hussain, S. M. & Rajeshwara Rao, N. Faunal affinity, zoogeographic distribution and review of recent Ostracoda from east and west coasts of India. *Bull. Pure Appl. Sci.–Sec. F Geol. Sci.* **15**, 37–50 (1996).
86. Hussain, S., Ragothaman, V. & Mavivannan, V. Distribution of Ostracoda in waters off Tuticorin, southeast coast of India. Indian *J. Mar. Sci.* **25**, 78–80 (1996).
87. Dewi, K. T. Ostracoda from the Java Sea, west of Bawean Island, Indonesia. *Mar. Geol. Inst. Spec. Publ.* **4**, 1–116 (1997).
88. Kumar, V. & Hussain, S. M. A report on Recent Ostracoda from Pitchavaram Mangroves, Tamil Nadu. *Geosci. J.* **18**, 131–139 (1997).
89. Naidu, T. Y., Varma, K. U. & Shyam Sunder, V. V. Diversity and distribution of Recent Ostracoda in the continental shelf sediments off Pentakota and Kalingapatnam, central east coast of India. *J. Geol. Soc. India* **50**, 727–738 (1997).
90. Hussain, S. M. Recent benthic Ostracoda from the Gulf of Mannar, off Tuticorin, southeast coast of India. J. Palaeontol. Soc. India **43**, 1–22 (1998).
91. Al-Jumaily, W. A. K. & Al-Sheikhly, S. S. Palaeozoogeography of shallow marine Ostracoda from Holocene sediments-southern Iraq. *Qatar Univ. Sci. J.* **18**, 215–230 (1999).
92. Carbonel, P., Hoibian, T. & Moyes, J. Écosystèmes et palèoenvironnements de la zone deltaïque de la Mahakam Depuis la fin du Néogène. (Le Sondage Misedor, Editions Technip*,* 1999)
93. Hussain, S. M. & Mohan, S. P. Recent Ostracoda from Adyar River Estuary, Chennai, Tamil nadu. *J. Palaeontol. Soc. India* **45**, 25–31 (2000).
94. Mohan, S. P., Ravi, G., Hussain, S. M. & Rajeshwara Rao, N. Recent Ostracoda from the Bay of Bengal, off Karikkattukuppam (near Chennai), south east coast of India. *J. Palaeontol. Soc. India* **46**, 1–14 (2001).
95. Sridhar, S. G. D., Hussain, S. M., Kumar, V. & Periakali, P. Recent benthic Ostracoda from Palk Bay, off Rameswaram, southeast coast of India. *J. Palaeontol. Soc. India* **47**, 17–39 (2002).
96. Warne, M. T. Palaeo-geomorphological significance of Miocene and Pliocene euryhaline Ostracoda in the Nepean 1 borehole, Port Phillip Basin, SE Australia. Mem. Assoc. Australasian Palaeontol. **27**, 139–148 (2002).
97. Mostafawi, N. Recent Ostracods from the Persian Gulf. *Senckenberg. Maritima* **32**, 51–75 (2003).
98. Helal, S. A. & Abd El-Wahab, M. Recent ostracodes from marine sediments of Safaga Bay, Red Sea, Egypt. *Egypt. J. Paleontol.* **4**, 75–93 (2004).
99. Montenegro, M. E., Pugliese, N. & Sciuto, F. Shallow water ostracods near the Mae Khlong river mouth (NW Gulf of Thailand). *Boll. Soc. Paleontol. Italiana* **43**, 225–234 (2004).
100. Mostafawi, N., Colin, J. P. & Babinot, J. F. An account on taxonomy of ostracodes from recent reefal flat deposits in Bali, Indonesia. *Rev. Micropaléontol.* **48**, 123–140 (2005).
101. Bhandari, A. & Singh, S. D. Recent Ostracoda from the deltaic subenvironment and shallow inner shelf, east coast of India. *J. Geol. Soc. India* **67**, 659–668 (2006).
102. Helal, S. A. & Abd El-Wahab, M. Distribution of podocopid ostracods in mangrove ecosystems along the Egyptian Red Sea Coast. *Crustaceana* **85**, 1669–1696 (2012).
103. Hussain, S. M., Krishnamurthy, R., Suresh Gandhi, M., Ilayaraja, K., Ganesan, P. & Mohan, S. P. Micropalaeontological investigations on tsunamigenic sediments of Andaman Islands. *Curr. Sci.* **91**, 1655–1667 (2006).
104. Pugliese, N., Montenegro, M. E., Sciuto, F. & Chaimanee, N. Environmental monitoring through the shallow marine ostracods of Phetchaburi area (NW Gulf of Thailand). In Proceedings of the second and third Italian meetings on environmental micropaleontology, Spec publication 11 (eds. Coccioni, R. & Marsili, A.) 85–90*(*Grzybowski Foundation, 2006).
105. Gopalakrishna, K., Hussain, S. M., Maheshbilwa, L. & Ayisha, V. A. Recent benthic Ostracoda from the inner-shelf off the Malabar Coast, Kerala, southwest coast of India. *J. Palaeontol. Soc. India* **52**, 59–68 (2007).
106. Hou, Y. & Gou, Y. Fossil Ostracoda of China. Volume 2: Cytheracea and Cytherellidae. (Science Publishing House, 2007)
107. Nor Faiz, N., Omar, R. & Jasin, B. Taburan Ostracod di dalam Sedimen Luar Pantai di Sekitar Pulau Tinggi, Johor. Sains. Malay. **36**, 139–148 (2007).
108. Tanaka, G., Komatsu, T., & Phong, N. D. Recent ostracod assemblages from the northeastern coast of Vietnam and the biogeographical significance of the euryhaline species. *Micropaleontology* **55**, 365–382 (2009).
109. Elumalai, K., Hussain, S. M. & Scott Immanuel Dhas, C. Recent benthic Ostracoda from the sediments of Ennore Creek, Chennai, Tamil Nadu, India. *J. Palaeontol. Soc. India* **55**, 11–22 (2010).
110. Ganesan, P. & Hussain, S. M. Distribution of Recent benthic Ostracoda in Tamiraparani Estuary, Punnaikayal, near Tuticorin, Southeast Coast of India: implications for microenvironments. *Gondwana Geol. Mag.* **25**, 103–114 (2010).
111. Hussain, S. M., Mohan, S. P. & Jonathan, M. P. Ostracoda as an aid in identifying 2004 tsunami sediments: a report from SE coast of India. *Nat. Hazards* **55**, 513–522 (2010).
112. Mostafawi, N., Nabavi, S. M. B. & Moghaddasi, B. Ostracods from the Strait of Hormuz and Gulf of Oman. *Rev. Esp. Micropaleontol.* **42**, 243–265 (2010).
113. Tanaka, G., Komatsu, T., Saito, Y., Nguyen, D. P. & Vu, Q. L. Temporal changes in ostracod assemblages during the past 10,000 years associated with the evolution of the Red River delta system, northeastern Vietnam. *Mar. Micropaleontol.* 81, 77–87 (2011).
114. Mohammed, M. & Keyser, D. Recent ostracods from the tidal flats of the coast of Aden City, Yemen. *Mar. Biodivers.* **42**, 247–280 (2012).
115. Baskar, K., Sridhar, S. G. D. & Hussain, S. M. Distribution of recent benthic Ostracoda off Rameswaram, Palk Strait, Tamil nadu, south east coast of India. *Spec. Publ. Geol. Soc. India* **1**, 195–212 (2013).
116. Fauzielly, L., Irizuki, T. & Sampei, Y. Spatial distribution of recent ostracode assemblages and depositional environments in Jakarta Bay, Indonesia, with relation to environmental factors. *Paleontol. Res.* **16**, 267–281 (2013).
117. Hussain, S. M., Elakkiya, P., Elumalai, K., Jonathan, M. P. & Nagarajan, R. Evidences for extreme wave events in Velanganni Coast, southeast of India. *Bol. Soc. Geol. Mexic.* **65**, 201–205 (2013).
118. Hussain, S. M., Kuleen, E. C., Jisha, K., Elumalai, K. & Ravi, G. Distribution of benthic Ostracoda in surface and subsurface backwater sediments of Ernakulam, Kerala, southwest coast of India: microenvironmental implications. In *Proceedings of XXIII Indian Colloquium on Micropaleontology and Stratigraphy and International Symposium on Global Bioevents in Earth’s History* (eds. Malarkodi, N., Gerta, K., Reddy, A. N. & Jaiprakash, B. C.). 213–224 (Geol. Soc. India, 2013).
119. Iwatani, H., Young, S. M., Irizuki, T., Sanpei, Y. & Ishiga, H. Spatial variations in recent ostracode assemblages and bottom environments in Trincomalee Bay, northeast coast of Sri Lanka. *Micropaleontology* **60**, 509–518 (2014).
120. Yamada, K., Terakura, M. & Tsukawaki, S. The impact on bottom sediments and ostracods in the Khlong Thom River mouth following the 2004 Indian Ocean tsunami. *Paleontol. Res.* **18**, 104–117 (2014).
121. Mahalakshmi, P. & Hussain, S. M. Disrtibution of ostracods from Pazhaverkadu (Pulicat Lagoon) Tamil Nadu, India. In *Lakes and Wetlands* (eds. Vasudevan, S., Ramkumar, T., Singhal, R. K., Rajanikanth, A. & Ramesh, G.) 268–287 (Partridge Publishing, 2015).
122. Mohammed Nishyath, N., Hussain, S. M. & Rajkumar, A. Distribution of Ostracoda in the sediments of the northwestern part of the Bay of Bengal, India – Implications for microenvironment. *J. Palaeontol. Soc. India* **60**, 27–33 (2015).
123. Hussain, S. M., Merin Maria Joy Rajkumar, A., Mohamed Nishath, N. & Fulmali, S. T. Distribution of calcareous microfauna (Foraminifera and Ostracoda) from the beach sands of Kovalam, Thiruvananthapuram, Kerala, southwest coast of India. *J. Palaeontol. Soc. India* **61**, 267–272 (2016).
124. Dewi, K. T., Latuputty, G., Priohandono, Y. A. & Purwanto, C. The respond of microfauna (Ostracoda) to marine environmental condition off Bangka Island, north Sulawesi. *J. Geol. Kelaut.* **15**, 1–9 (2017).
125. Hong, Y., Yasuhara, M., Iwatani, H., Seto, K., Yokoyama, Y., Yoshioka, K. & Mamo, B. Freshwater reservoir construction by damming a marine inlet in Hong Kong: Paleoecological evidence of local community change. *Mar. Micropaleontol.* **132**, 53–59 (2017).
126. Hussain, S. M. An overview of Ostracoda studies from the freshwater, marginal marine and marine ecosystems of Andaman and Nicobar islands and the coasts of India. In *Micropaleontology and Its Applications* (eds. Kathal, P. K., Nigam, R. & Talib, A.) 135–162 (Scientific Publishers, 2017).
127. Noraswana, N. F., Kamal, R. M. & Ramlan, O. Species diversity and distribution of Ostracoda (Crustacea) in Sungai Kubang Badak, Langkawi, Kedah, Malaysia. Malay. Nat. J. **69**, 83–92 (2017).
128. Yousef, E. Distribution and taxonomy of shallow marine ostracods from western coast of the Red Sea Egypt. *Open J. Mar. Sci.* **8**, 51–75 (2018).
129. Wang, H., Zhang, H., Cao, M. & Horne, D. J. Holocene ostracods from the Hang Hau Formation in Lei Yue Mun, Hong Kong, and their palaeoenvironmental implications. Alcheringa **43**, 320–333 (2018).
130. Hussain, S. M., Mahalakshmi, P. & Selvasundaram, S. Distribution of ostracods in the mangrove location of Pulicat Lagoon, Tamil nadu, southeast coast of India. *Asian Acad. Res. J. Multidiscip* **5**, 234–250 (2018).
131. Mishra, R., Hussain, S. M. & Nazeer, M. N. Distribution of ostracoda and foraminifera from sediments of Chilika Lagoon, Odisha, East Coast of India. *J. Palaeontol. Soc. India* **64**, 115–120 (2019).
132. Niiyama, S., Tanaka, G. & Komatsu, T. Recent shallow water ostracod assemblage from the river mouth of the Giang Thanh River, southern Vietnam. *Proc. 2019 CWMD Conf., Kumamoto Univ.*, 694–701 (2019).
133. Niiyama, S., Tanaka, G., Komatsu, T., Doan, H. D., Nguyen, H. B., Trinh, H. T. & Nguyen, M. T. Holocene ostracod assemblages from the Co To Islands, northeastern Vietnam. *J. Micropalaeontol.* **38**, 97–111 (2019).
134. Sridhar, S. G. D., Kumari Deepali Shiva Prabhu, V., Hussain, S. M. & Maniyarasan, S. Distribution of recent benthic Ostracoda, around Pullivasal and Poomarichan Islands, off Rameswaram, Gulf of Mannar, southeast coast of India. *J. Palaeontol. Soc. India* **64**, 27–38 (2019).
135. Rajkumar, A., Hussain, S. M., Mohammed Nishath, N., Dewi, K. T., Sivapriya, V. & Radhakrishnan, K. Recent Ostracod biodiversity from shelf to slope sediments of Gulf of Mannar, India: ecologic and bathymetric implications. *J. Palaeontol. Soc. India* **65**, 1–7 (2020).
136. Forel, M. B. Recent ostracods (Crustacea) from the southwestern coast of Peninsular Thailand (Satun Province), Andaman Sea. *Rev. Micropaléontol.* **72**, 1–14 (2021).
137. Keyser, O. & Mohammed, M. Taxonomy of recent shallow marine Ostracods from Al-Hudeida

City-Yemen. *Mar. Micropaleontol.* **164**, 1–17 (2021).

1. Tan, C. W. J., Gouramanis, C., Pham, T. D., Hoang, D. Q. & Switzer, A. D. Ostracods as pollution indicators in Lap An Lagoon, central Vietnam. Environ. Pollut. **278**, 116762 (2021).
2. Radhakrishnan, K., Hussain, S. M., Sivapriya, V., Rajkumar, A., Nazeer, M. N. & Akram Khan, N. Distribution of recent Ostracoda from the sediments of Gulf of Mannar, Tamil Nadu, southeast coast of India. *J. Palaeontol. Soc. India* **67**, 229–236 (2022).
3. Yousef, E. The impact of some environmental factors on the distribution of the benthic ostracod species from of Safaga Island, Red Sea, Egypt. *Open J. Mar. Sci.* **12**, 83–107 (2022).
4. Sars, G. O. An account of the Crustacea of Norway with short descriptions and figures of all the species, volume 9 (Ostracoda) (Parts 13-14) 209–240 (Bergen Mus., 1926).
5. Fischer, S. Beitrag zur Kenntnis der Ostracoden. Abh. Math.-Phys. Cl. K.-Beyerischen Akad. Wiss. **7**, 635–666 (1855).
6. Ishizaki, K. Ostracoda from the East China Sea. *Sci. Rep. Tohoku Univ. Second Ser.* **51**, 37–65 (1981).
7. Hanai, T. Studies on the Ostracoda from Japan: Hingement. *J. Fac. Sci., Univ. Tokyo, Sec. 2* **13**, 345–377 (1961).
8. Cheong, H., Lee, E., Paik, K. & Chang, S. Recent ostracodes from the southwestern slope of the Ulleung Basin, East Sea, Korea. *J. Paleontol. Soc. Korea* **2**, 38–53 (1986).
9. Wang, Q. *&* Zhang, L. Holocene ostracod fauna and paleoenvironment in the sea region around Hong Kong. Acta Oceanol. Sin. **6**, 281–291 (1987).
10. Ikeya, N., Hasegawa, K. & Kashima, T. 3. Analysis of ostracod fossil – Holocene ostracod assemblage from Kawasaki City*.* In *The geological studies on the alluvial deposits in the Tama and Tsurumi rivers lowland, Kawasaki* (ed. Matsushima, Y.) 51–64 (Comm. Collecting Depos. Kawasaki City Mus., 1987).
11. Zhao, Q. & Wang, P. Modern Ostracoda in shelf seas off China: Zoogeographical zonation. *Oceanol. Limnol. Sin.* **21**, 458–464 (1990).
12. Ikeya, N. & Itoh, H. Recent Ostracoda from the Sendai Bay region, Pacific coast of northeastern Japan. Rep. Fac. Sci., Shizuoka Univ. **25**, 93–145 (1991).
13. Irizuki, T., Fujiwara, O., Fuse, K. & Masuda, F. Paleoenvironmental changes during the last post glacial period on the western coast of the Miura Peninsula, Kanagawa Prefecture, Central Japan: Fossil ostracode fauna and event deposits in bore hole cores. Fossils **64**, 1–22 (1998).
14. Yasuhara, M. & Irizuki, T. Recent Ostracoda from the northeastern part of Osaka Bay, southwestern Japan. *J. Geosci., Osaka City Univ.* 44, 57–95 (2001).
15. Irizuki, T., Kamiya, M. & Ueda, K. Temporal and spatial distribution of fossil ostracode assemblages and sedimentary facies in the Middle Pleistocene Tahara Formation, Atsumi Peninsula, central Japan. Geosci. Rep. Shimane Univ. **21**, 31–39 (2002).
16. Ishii, T., Kamiya, T. & Tsukagoshi, A. Phylogeny and evolution of Loxoconcha (Ostracoda, Crustacea) species around Japan. Hydrobiologia **538**, 81–94 (2005).
17. Sasaki, Y., Irizuki, T., Abe, K., Uchida, J.-I. & Fujiwara, O. Fossil ostracode assemblages from Holocene Tsunami and normal bay deposits along the Tomoe River, Tateyama, Boso Peninsula, central Japan. Quat. Res. **46**, 517–532 (2007).
18. Ozawa, H. & Domitsu, H. Early Pleistocene ostracods from the Hamada Formation in the Shimokita Peninsula, northeastern Japan: the palaeobiogeographic significance of their occurrence for the shallow-water fauna. Paleontol. Res. **14**, 1–18 (2010).
19. Schornikov, E. I. & Zenina, M. A. Ostracods as indicators of conditions and dynamics of water ecosystems (on the example of Peter the Great Bay, Sea of Japan) (Dalnaula, 2014).
20. Irizuki, T., Hirose, K., Ueda, Y., Fujiwara, Y., Ishiga, H. & Seto, K. Ecological shifts due to anthropogenic activities in the coastal seas of the Seto Inland Sea, Japan, since 20th century. Mar. Pollut. Bull. **127**, 637–653 (2018).
21. Hong, Y., Yasuhara, M., Iwatani, H. & Mamo, B. Baseline for ostracod-based northwestern Pacific and Indo-Pacific shallow-marine paleoenvironmental reconstructions: ecological modeling of species distributions. *Biogeosciences* **16**, 585–604 (2019).
22. Noda, H. & Nishikawa, T. eds. Japanese Text of International Code of Zoological Nomenclature. In The International commission on zoological nomenclature. (eds. Ride, W. D. L., Cogger, H. G., Dupuis, C., Kraus, O., Minelli, A., Thompson, F. C. & Tubbs, P. K.) 1–133 (Union of Japanese Societies for Systematic Zoology, Hokkaido University, 2000).
23. Zhao, Q Study of Recent ostracode distribution of the coastal zone of Yellow Sea, East China Sea. *Act. Oceanol. Sin.* **7**, 193–204 (1985).
24. Ruan, P. Distribution of Recent Ostracoda in the littoral zone of Beidaihe, Hebei Province. *Geoscience* **3**, 377–387 (1989).
25. Irizuki, T., Matsubara, T. & Matsumoto, H. Middle Pleistocene Ostracoda from the Takatsukayama ember of the Meimi Formation, Hyogo Prefecture, western Japan: significance of the occurrence of Sinocytheridea impressa. Paleontol. Res. **9**, 37–54 (2005).
26. Irizuki, T., Gotomyo, A., Kawano, S., Yoshioka, K. & Nomura, R. Relationships between ostracode assemblage and environmental factors in the northern part of Harima-nada Bay off Aioi City, Hyogo Prefecture, Southwest Japan. *Geosci. Rep. Shimane Univ.* **28**, 1–9 (2009).
27. Goto, T., Nasuno, S., Irizuki, T., Ohira, H. & Hayashi, H. Fossil track age for MT1 tuff bed and paleoenvironment in the Upper Pliocene Mita Formation, Toyama Prefecture, central Japan. *J. Geol. Soc. Jpn.* **120**, 71–86 (2014).
28. Yamaguchi, T., Terada, T. & Morono, Y. Osmium plasma coating for observation of microfossils, using optical and scanning electron microscopes. *Paleontol. Res.* **20**, 296–301 (2016).
29. Yamada, K., Masuda, T., Seto, K., Uchida, M., Amano, A. & Sampei, Y. Paleoenvironments and relative sea-level changes caused by regional tectonics during the last 4500 years in Kumihama Bay, northern Kyoto Prefecture, central Japan. *Quat. Int.* **471**, 332–344 (2018).
30. Yoo, H. & Karanovic, I. Six species of the ostracod families Loxoconchidae and Cushmanideidae from South Korea. *J. Species Res.* **8**, 116–127 (2019).
31. Irizuki, T., Fujiwara, O., Yoshioka, K., Suzuki, A., Tanaka, Y., Nagao, M., Kawagata, S., Kawano, S. & Nishimura, O. Geochemical and micropaleontological impacts caused by the 2011 Tohoku-oki tsunami in Matsushima Bay, northeastern Japan. Mar. Geol. **407**, 261–274 (2019).
32. Kajiyama, E. The Ostracoda of Misaki, Part 3. *Zool. Mag. Tokyo (Doubutsugaku-zassi)* **25**, 1–16 (1913).
33. Hanai, T. Studies on the Ostracoda from Japan: Historical review with bibliographic index of Japanese Ostracoda. *J. Fac. Sci., Univ. Tokyo, Sec. 2* **11**, 419–439 (1959).
34. Igo, H. & Ikeya, N. Micro-organisms and fossils of the sea bottom. In Form of the organic world (eds. Sakaguchi et al.) 186–209 (Kodansha, 1971).
35. Hu, C. Studies on ostracode faunas from the Hungchun Formation, Hungchun Peninsula, southern Taiwan. Pet. Geol. Taiwan **16**, 59–84 (1979).
36. Okubo, I. Taxonomic studies on recent marine podocopid Ostracoda from the Inland Sea of Seto. *Publ. Seto Mar. Biol. Lab.* **25**, 389–443 (1980).
37. Hu, C. Studies on ostracod faunas from the Hengchun Limestone (Pleistocene), southern Taiwan. *Q. J. Taiwan Mus.* **34**, 61–93 (1981).
38. Hu, C. Ostracods from the Maanshan Mudstone, Hengchun Peninsula, Southern Taiwan. Pet. Geol. Taiwan **19**, 149–178 (1983).
39. Hu, C. New fossil ostracod fauna from Hangchun Peninsula, southern Taiwan. *J. Taiwan Mus.* **37**, 65–130 (1984).
40. Hu. C. & Tao, H. Fossil ostracods from the Fengshan Water Reservoir, Kaoshiung District, Southern Taiwan. *Acta Geol. Taiwanica* **24**, 51–65 (1986).
41. Kamiya, T. Contrasting population ecology of two species of *Loxoconcha* (Ostracoda, Crustacea) in Recent Zostera (eelgrass) beds: adaptive differences between phytal and bottom-dwelling species. *Micropaleontology* **34**, 316–331 (1988).
42. Kamiya, T. Different sex-ratios in two Recent species of *Loxoconcha* (Ostracoda). *Senckenberg. lethaea* **68**, 337–345 (1988).
43. Kamiya, T. Morphological and ethological adaptation of Ostracoda to microhabitats in Zostera beds. In Evolutionary biology of Ostracoda (eds. Hanai, T., Ikeya, N. & Ishizaki, K.) 303–318 (Kodansha, 1988).
44. Kamiya, T. Differences between the sensory organs of phytal and bottom-dwelling Loxoconcha (Ostacoda, Crustacea). J. Micropalaeontol. **8**, 37–47 (1989).
45. Kamiya, T. Functional morphology of Ostracoda in seagrass beds – with special reference to the copulatory behavior. Benthos Res. **35/36**, 75–88 (1989).
46. Ikeya, N. & Suzuki, C. Distribution patterns of modern ostracodes off Shimane Peninsula, southwestern Japan Sea. Rep. Fac. Sci., Shizuoka Univ. **26**, 91–137 (1992).
47. Kamiya, T. & Hazel, J. E. Shared versus derived characters in the pore-system of Loxoconcha (Ostracoda, Crustacea). J. Micropalaeontol. **11**, 159–166 (1992).
48. Nohara, T. & Oshiro, I. Some aquatic and terrestrial animals from brackish-water deposits of Okinawa-Jima, Southern Japan. In Centenary of Japanese micropaleontology (eds. Ishizaki, K. & Saito, T.) 335–337 (Terra Scientific Publishing Company, 1992).
49. Tabuki, R. Plio-Pleistocene and Recent Subtropical Ostracoda in Okinawa. In Guidebook of Excursions, 14th International Symposium on Ostracoda (ed. The Organising Committee of ISO 2001) 21–44 (Shizuoka University Press, 2001).
50. Tanaka, G. Two simple methods for observation of shapes in podocopid ostracod valves. *J. Fossil Res.* **35**, 25–27 (2002).
51. Tanaka, G. & Ikeya, N. Migration and speciation of the *Loxoconcha japonica* species group (Ostracoda) in East Asia. *Paleontol. Res.* **6**, 265–284 (2002).
52. Smith, R. J. & Kamiya, T. The ontogeny of *Loxoconcha japonica* Ishizaki, 1968 (Cytheroidea, Ostracoda, Crustacea). *Hydrobiologia* **490**, 31–52 (2003).
53. Horne, D. J., Smith, R. J., Whittakker, J. E. & Murray, J. W. The first British record and a new species of the superfamily Terrestricytheroidea (Crustacea, Ostracoda): morphology, ontogeny, lifestyle and phylogeny. *Zool. J. Linn. Soc.* **142**, 253–288 (2004).
54. Smith, R. J. & Kamiya, T. The ontogeny of the entocytherid ostracod *Uninocythere occidentalis* (Kozloff and Whitman, 1954) Hart, 1962 (Crustacea). *Hydrobiologia* **538**, 217–229 (2005).
55. Smith, R. J. & Tsukagoshi, A. The chaetotaxy, ontogeny and musculature of the antennule of podocopan ostracods (Crustacea). *J. Zool. Soc. Lond.* **265**, 157–177 (2005).
56. Kamiya, T., Sato, T. & Kumakiri, M. Evolution of the Ostracoda and the Environmantal Changes of the Japan Sea. In: Past, present and future environments of Pan-Japan Sea Region (ed. Hayakawa, K.) 109–125 (Maruzen, 2006).
57. Tanaka, G. Kinematics of adductor muscle and pattern formation of various shell ornamentations in marine and brackish water ostracods. *Paleontol. Res.* **11**, 123–133 (2007).
58. Savatenalinton, S. & Martens, K. On a freshwater species of the genus *Sanyuania* Zhao and Han, 1980 (Crustacea, Ostracoda, Loxoconchidae) from Thailand, with a discussion on morphological evolution of the freshwater Loxoconchidae. *J. Nat. Hist.* **43**, 259–285 (2009).
59. Tanaka, G. & Nomura, S. Late Miocene and Pliocene Ostracoda from the Shimajiri Group, Kume-jima Island, Japan: Biogeographical significance of the timing of the formation of back-arc basin (Okinawa Trough). *Palaeogeogr., Palaeoclimatol., Palaeoecol.* **276**, 56–68 (2009).
60. Morishita, T., Yamaguchi, T., Mashiba, H. & Kamiya, T. A review of the utility of Mg/Ca and Sr/Ca ratios of ostracode valves as a tool for paleoenvironmental reconstructions. *J. Geol. Soc. Jpn.* **116**, 523–543 (2010).
61. Kawano, S., Tsujimoto, A., Ugai, H., Irizuki, T. & Nomura, R. Paleoenvironmental and microfossil assemblages in the Pleistocene Ogushi Formation, Kumamoto Prefecture, Southwest Japan. J. Foss. Res. **44**, 1–10 (2011).
62. Tanaka, G., Kaneko, M., Oishi, M. & Participants of 2011 SPP class in Ota Girls’ High School Ostracods from the Holocene Numa Formation, Chiba Prefecture, central Japan. *Bull. Gunma Mus. Nat. Hist.* **16**, 111–118 (2012).
63. Kawano, S., Irizuki, T. & Nomura, R. Temporal changes of recent ostracode assemblages and bay environment with relation to the opening and extension of the Manzaki-seto Strait in the Tsushima Island, southwest Japan. Earth Sci. (Chikyu Kagaku) **67**, 89–99 (2013).
64. Tsukagoshi, A. Studies on the living organisms for paleontology, part 2: Case studies for evolution and biodiversity of ostracods. Fossils **102**, 15–30 (2017).
65. Le, D. D. & Tsukagoshi, A. First report on two ostracod genera *Loxoconcha* Sars, 1866 (Loxoconchidae) and *Xestoleberis* Sars, 1866 (Xestoleberididae) along the coast of Vietnam. *Acad. J. Biol.* **41**, 15–24 (2019).
66. Tanaka, G., Maekawa, T., Urakawa, R., Komatsu, T. & Henmi, Y. Checklist of ostracods from the Pleistocene Ogushi Formation, Amakusa City, Kumamoto, southwestern Japan. *Bull. Goshoura Cretaceous Mus.* **20**, 7–26 (2019).
67. Ozawa, H., Kamiya, T. & Tsukagoshi, A. Ostracode evidence for the palaoceanographic change of the Middle Pleistocene Jizodo and Yabu formations in the Boso Peninsula, central Japan. *Sci. Rep. Kanazawa Univ.* **40**, 9–37 (1995).
68. Iwatani, H. & Irizuki, T. Geology and fossil ostracode assemblages from the Pliocene Miyazaki Group in the northern part of the Miyazaki Plain, Southwest Japan. *Fossils* **84**, 61–73 (2008).
69. Yamaguchi, S. Morphological evsolution of Cytherocopine ostracods inferred from 18S ribosomal DNA sequences. *J. Crustac. Biol.* **23**, 131–153 (2003).
70. Ozawa, H. & Ishii, T. Taxonomy and sexual dimorphism of a new species of *Loxoconcha* (Podocopida: Ostracoda) from the Pleistocene of the Japan Sea. *Zool. J. Linn. Soc.* **153**, 239–251(2008).
71. Ozawa, H. Extinction of cytheroidean ostracodes (Crustacea) in shallow-water around Japan in relation to environmental fluctuation since the Early Pleistocene. In Species diversity and extinction (eds. Geraldine, H & Tepper, H.) 61–109 (Nova Science Publishers, 2010).
72. Ozawa, H. The history of sexual dimorphism in Ostracoda (Arthropoda, Crustacea) since the Palaeozoic. In Sexual dimorphism (ed. Moriyama, H.) 51–80 (Intech Open, 2013).
73. Benson, R. H. & Coleman, G. L. Recent marine Ostracodes from the eastern Gulf of Mexico. Univ. Kansas Paleontol. Contrib., Artic. 31 Arthropoda **2**, 1–52 (1963).
74. Brady, G. S. Description of Ostracoda. In Les Fonds de la Mer (eds. Folin, L. D. & Périer, L.) 113–176 (Savy, Libraire–Éditeur, 1869).
75. Takayasu, K., Ueda, K. & Ohta, H. Natural history of the Nakano-umi and Shinji lakes – XII. Ostracode thanathocoenoces from the bottom deposits of Lake Nakano-umi and their historical distribution –. Geol. Rep. Shimane Univ. **9**, 129–144 (1990).
76. Yajima, M. & Lord, A. The interpretation of Quaternary environments using Ostracoda: an example from Japan. *Proc. Geologist’s Association* **101**, 153–161 (1990).
77. Huh, M. & Paik, K. Miocene Ostracoda from the Pohang Basin, Korea. Paleontol. Soc. Korea, Spec. Publ. **1**, 101–119 (1992).
78. Ishizaki, K., Irizuki, T. & Sasaki, O.Cobb mountain spike of the Kuroshio Current detected by Ostracoda in the lower Omma Formation (Early Pleistocene), Kanazawa City, central Japan: analysis of depositional environments. In *Ostracoda in the earth and life sciences* (eds. Mckenzie, K. G. & Jones, P. J.) 315–334 (Chapman and Hall, 1993).
79. Ishida, K. & Takayasu, K. Ostracode assemblages of alluvium sediment cores in the western part of Izumo Plain, Shimane Prefecture. Laguna **12**, 73–79 (2005).
80. Ozawa, H. & Kamiya, T. Ecological analysis of benthic ostracods in the northern Japan Sea, based on water properties of modern habitats and late Cenozoic fossil records. *Mar. Micropaleontol.* **55**, 255–276 (2005).
81. Ozawa, H. Middle Pleistocene ostracods from the Naganuma Formation, Sagami group, central Japan: significance of the occurrence for the bay fauna along the Northwest Pacific margin. *Paleontol. Res.* 13, 231–244 (2009).
82. Irizuki, T., Naya, T., Yamaguchi, M. & Mizuno, K. Temporal changes in the paleoenvironment of the inner part of paleo-Tokyo Bay during the middle Pleistocene (MIS 11 and MIS 9): Analysis of fossil ostracode assemblages from the Simosa Group in the Shobu core, Saitama Prefecture, Central Japan. J. Geol. Soc. Jpn. **117**, 35–52 (2011).
83. Kaneko, M., Ishikawa, H., Harashima, M., Nomura, M. & Nakazawa, T. Fossil foraminiferal and ostracode assemblages from the Pleistocene Tokyo Formation in cores GS-SE-1, Kami-Yoga and GS-SE-3, Komazawa, Setagaya, Tokyo, central Japan. Bull. Geol. Surv. Jpn. **73**, 49–65 (2022).
84. Gou, Y., Chen, T., Guan, S., Jiang, Y., Liu, Z., Lai, X., Wu, Q. & Chen, C. Ostracoda. In *Tertiary palaeontology of north continental shelf of South China Sea* (eds. South Sea Branch of Petroleum Corporation of the People’s Republic of China, China National Geological Exploration Corporation Guangzhou Branch, Nanjing Institute of Geology and Palaeontology, Academia Sinica, Institute of Botany, Academia Sinica, Tong-Ji University, Shanghai, China Academy of Geological Sciences, etc.) 138–187 (Guangdong Science and Technology Press, 1981).
85. Ishizaki, K. Detailed survey on ostracods in the drilling no. 56-9 core samples at the Kansai International Airport in Osaka Bay. In *Samples at the Kansai International Airport in Osaka Bay, geological survey of the submarine strata at the Kansai International Airport in Osaka Bay, Central Japan* (ed. Nakaseko, K.) 37–43 (Calamity Science Institute, 1984).
86. Yajima, M. Preliminary notes on the Japanese Miocene Ostracoda. In Evolutionary biology of Ostracoda. (eds. Hanai, T., Ikeya, N. & Ishizaki, K.) 1073–1085 (Kodansha, 1988).
87. Irizuki, T. & Matsubara, T. Vertical changes of depositional environments of the Lower to Middle Miocene Kadonosawa Formation based on analyses of fossil ostracode faunas. J. Geol. Soc. Jpn. **100**, 136–149. (1994).
88. Tsukagoshi, A., Kosugi, M., Kurosawa, K., Matsunaga, M., Momoi, S. & Nakao, Y. Holocene environmental history in the Paleo-Nagareyama Bay, central Japan. Matsudo City Mus. Res. Rep. **2**, 19–61 (1994).
89. Irizuki, T. & Hosoyama, M. Fossil ostracodes (Crustacea) from the Pleistocene Noma Formation, Aichi Prefecture, central Japan. *Bull. Aichi Univ. Educ. (Nat. Sci.)* **49**, 9–15 (2000).
90. Nakao, Y. & Tsukagoshi, A. Brackish-water Ostracoda (Crustacea) from the Obitsu River estuary, central Japan. *Spec. Divers.* **7**, 67–115 (2002).
91. Yasuhara, M., Irizuki, T., Yoshikawa, S. & Nanayama, F. Holocene sea-level changes in Osaka Bay, western Japan: ostracode evidence in a drilling core from the southern Osaka Plain. *J. Geol. Soc. Jpn.* **108**, 633–643 (2002).
92. Irizuki, T., Yamada, K., Maruyama, T. & Ito, H. Paleoecology and taxonomy of Early Miocene Ostracoda and paleoenvironments of the eastern Setouchi Province, central Japan. Micropaleontology **50**, 105–147 (2004).
93. Yasuhara, M., Irizuki, T., Yoshikawa, S., Nanayama, F. & Mitamura, M. Holocene ostracode paleobiogeography in Osaka Bay, southwestern Japan. *Mar. Micropaleontol.* **53**, 11–36 (2004).
94. Yamada, S. Structure and evolution of podocopan ostracod hinges. *Biol. J. Linn. Soc.* **92**, 41–62 (2007).
95. Yamada, S. Ultrastructure of the carapace margin in the Ostracoda (Arthropoda: Crustacea). *Hydrobiologia* **585**, 201–211 (2007).
96. Yamada, S. Review of studies on structure and cuticle formation of ostracod carapace. *Fossils* **83**, 41–58 (2008).
97. Nakao, Y. & Tsukagoshi, A. Seasonal and decadal changes in distribution and abundance of Ostracoda (Crustacea) in the brackish-water environments. *Proc. Inst. Nat. Sci., Nihon Univ.* **43**, 249–275 (2008).
98. Yamada, S. & Keyser, D. Cuticle formation of the adductor muscle attachment in a podocopid ostracod, as an example of a calcified arthropod. *Paleontol. Res.* **13**, 103–118 (2009).
99. Yamada, S. & Matzke-Karasz, R. Skeleto-musculature of the mandible and its function in podocopid ostracodes exemplsified by *Loxoconcha pulchra* (Cytheroidea: Loxoconchidae) and Fabaeformiscandona tyrolensis (Cypridoidea: Candonidae). *J. Morphol.* **272**, 1342–1352 (2011).
100. Tanaka, G., Matsushima, Y. & Maeda, H. Holocene Ostracods from the borehole core at Oppama Park, Yokosuka City, Kanagawa Prefecture, Central Japan: Paleoenvironmental analysis and the discovery of a fossil ostracod with three-dimensionally preserved soft parts. *Paleontol. Res.* **16**, 1–18 (2012).
101. Matsushima, Y., Tanaka, G., Chiba, T., Kudo, Y., Kaneko, M., Ishikawa, H., Nomura, M., Sugihara, S. & Masubuchi,K. Paleorenvironmantal change of the Hirakata-wan Inlet, west of Tokyo Bay, curing the Jomon Transgression. *Bull. Kanagawa Pref. Mus. (Nat. Hist.)* **45**, 1–27 (2016).
102. Karanovic, I., Yoo, H., Tanaka, H. & Tsukagoshi, A. One new species and three records of cytheroid ostracods (Crustacea, Ostracoda) from Korea. J. Species. Res. **6** (Spec. Edition), 38–50 (2017).
103. Karanovic, I., Yoo, H., Tanaka, H. & Tsukagoshi, A. One new species and three records of cytheroid ostracods (Crustacea, Ostracoda) from Korea. J. Species. Res. **6** (Spec. Edition), 38–50 (2017).
104. Ishizaki, K. Analysis of ostracode fauna as an indicator of paleoenvironment. *Mar. Sci. Monthly* **7**, 53–58 (1975).
105. Iwasaki, Y. Ostracod assemblages from the Holocene deposits of Kumamoto, Kyushu. *Mem. Fac. Sci., Kumamoto Univ.* **13**, 1–12 (1992).
106. Yasuhara, M. & Seto, K. Holocene relative sea-level change in Hiroshima Bay, Japan: a semi-quantitative reconstruction based on ostracodes. *Paleontol. Res.* **10**, 99–116 (2006).
107. Irizuki, T., Seto, K. & Nomura, R. The impact of fish farming and Back construction on Ostracoda in Uranouchi Bay on the Pacific coast of southwest Japan – Faunal changes between 1954 and 2002/2005. Paleontol. Res. **12**, 283–302 (2008).
108. Irizuki, T., Ito, H., Yoshioka, K., Kawano, S., Nomura, R., Tanaka, Y. & Sako, M. Recent ostracode assemblages and the marine environment around Kasado Bay in northeastern Suo-nada Bay, Yamaguchi Prefecture, Southwest Japan. Geosci. Rep. Shimane Univ. **29**, 11–20 (2010).
109. Irizuki, T., Ito, H., Sako, M., Toshioka, K., Kawano, S., Nomura, R. & Tanaka, Y. Anthropogenic impacts on meiobenthic Ostracoda (Crustacea) in the moderately polluted Kasado Bay, Seto Island, Seto Inland Sea, Japan, over, the past 70. Mar. Pollut. Bull. **91**, 149–159 (2015).
110. Irizuki, T., Fujiwara, Y., Iwatani, H. & Kawano, S. Recent ostracode assemblages from Shushi Bay, Tsushima Island, southwestern Japan and their ecological and zoogeographical characteristics. Laguna **25**, 39–54 (2018).
111. Tanaka, G., Henmi, Y., Masuda, T., Moriwaki, H., Komatsu, T., Zhou, B., Maekawa, T., Niiyama, S., Nguyen, D. P. & Doan, H. D. Recent ostracod distribution in western Kyushu, Japan, related to the migration of Chinese continental faunal elements. *Mar. Micropaleontol.* **146**, 1–38 (2019).
112. Sasaki, S., Irizuki, T., Urabe, A., Hayashi, H., Seto, K. & Sakai, T. Holocene paleoenvironment and relative sea-level change in Ashibe Port, Iki Island, Nagasaki Prefecture, southwestern Japan. *Laguna* **27**, 1–18 (2020).
113. Irizuki, T., Takahashi, J., Seto, K., Ishiga, H., Fujihara, Y. & Kawano, S. Response of bay ostracod assemblages to Late Holocene sea-level, centennial-scale climate, and human-induced factors in northeast Beppu Bay, Japan. Mar. Micropaleontol. **165**, 1–17 (2021).
114. Holden, J. C. Late Cenozoic Ostracoda from Midway Island drill holes. *U. S. Geol. Surv. Prof. Pap.* **680-F**, 1–41 (1976).
115. Hanai, T., Ikeya, N. & Yajima, M. Checklist of Ostracoda from Southeast Asia. Univ. Mus., Univ. Tokyo Bull. **17**, 1–236 (1980).
116. Tabuki, R., Nakano, T. & Nohara, T. Preliminary report on ostracode fauna from Sekisei-sho area, Yaeyama Islands. *Bull. Coll. Educ., Univ. Ryukyus* **31**, 323–335 (1987).
117. Tabuki, R. & Nohara, T. The Ostracoda of the Sekisei-sho area, Ryukyu islands, Japan: a preliminary report on ostracods from coral reefs in the Ryukyu Islands. In Ostracoda and global events (eds. Whatley, R. & Maybury, C.) 365–373 (Chapman and Hall, 1990).
118. Kamiya, T. Heterochronic dimorphism of Loxoconcha uranouchiensis (Ostracoda) and its implication for speciation. Paleobiology **18**, 221–236 (1992).
119. Tabuki, R. & Nohara, T. Seasonal distribution of intertidal ostracodes on gravels from the moat behind a coral reef off Sesoko Island, Ryukyu Islands, Japan. In Ostracoda and biostratigraphy (ed. Riha, J.) 343–349 (Balkema, 1995).
120. Tanaka, G., Seto, K. & Takayasu, K. The relationship between environments and ostracode assemblages from Miho Bay to Lake Shinji. Laguna **5**, 81–91 (1998).
121. Nakao, Y., Tanaka, G. & Yamada, S. Pleistocene and Living Marine Ostracoda in Shizuoka District, Japan. In Guidebook of Excursions, 14th International Symposium on Ostracoda (ed. The Organising Committee of ISO 2001) 127–147 (Shizuoka University Press, 2001).
122. Tanaka, G. Morphological design and fossil record of the podocopid ostracod naupliar eye. *Hydrobiologia* **538**, 231–242 (2005).
123. Nakao, Y., Nakanishi, T. & Kimura, K. Ostracode fossil assemblages from the latest Pleistocene-Holocene deposits under the southern part of Nakagawa Lowland. *Proc. Inst. Nat. Sci., Nihon Univ.* **43**, 277–286 (2008).
124. Horikoshi, H., Nakao, Y. & Endo, K. Paleoenvironmental changes on ostracode fossil assemblages from the latest Holocene deposits in Misato City, Saitama Prefecture (Central Japan). *Proc. Inst. Nat. Sci., Nihon Univ.* **44**, 149–157 (2009).
125. Tabuki, R. & Nakanishi, A. Benthic ostracod fauna from Man-ko in the southern part of Okinawa Island, Japan. *Bull. Fac. Educ. Univ. Ryukyus* **84**, 253–264 (2014).
126. Kamiya, T., Ozawa, H. & Kitamura, A. Paleo-water mass structure during the deposition of middle part of the Omma Formation based on the change of ostracode assemblage. Hokuriku Geol. Inst. Rep. **5**, 145–165 (1996).
127. Irizuki, T., Takimoto, A., Sako, M., Nomura, R., Kakuno, K., Wanishi, A. & Kawano, S. The influences of various anthopogenic sources of deterioration on meiobenthos (Ostracoda) over the last 100 years in Suo-Nada in the Seto Inland Sea, southwest Japan. Mar. Pollut. Bull. **62**, 2030–2041 (2011).
128. Irizuki, T., Kobe, M., Okushi, K., Kawahata, H. & Kimoto, K. Centennial – to millennial-scale change of Holocene marine environments recorded in Ostracode fauna, northeast Japan. Quat. Res. **84**, 467–480 (2015).
129. Maehama, Y., Kano, K., Oki, K., Irizuki, T. & Hayashi, H. Latest Pleistocene to Holocene calcareous microfossil assemblages from the Shinjima (Moeshima) Island and reconstruction of the paleoenvironments in the inner part of Kagoshima Bay, SW Japan. *J. Geol. Soc. Jpn.* **127**, 363–376.
130. Hirschmann, N. Beitrag zur Kenntnis der Ostracodenfauna des Finnischen Meerbusens. Medd. Soc. Fauna et Flora Fenn. **35**, 282–296 (1909).
131. Brady, G. S. Report on the Ostracoda dredged by H.M.S. Challenger during the Years 1873-1876. Zoology **1**, 1–184 (1880).
132. Puri, H. F. & Hulings, H. C. Designation of lectotypes of some ostracods from the Challenger Expedition. *Bull. Br. Mus. (Nat. Hist.) Zool.* **29**, 251–315 (1976).
133. Okubo, I. Two species of the family Cytheridae (Ostracoda) in the Inland Sea, Japan. *Res. Bull. Okayama Shujitsu Jr. Coll.* **8**, 89–98 (1978).
134. Kim, W. H. & Park, Y.-A. Microbiogenic sediments in the Nagdong Estuary, Korea. *J. Oceanol. Soc. Korea* **15**, 34–48 (1980).
135. Ikeya, N. The ostracodes deposited in the Hancock Museum, Newcastle-upon-Tyne, collected from the Yokohama Harbor and the coast of Misaki. Nat. Hist. Rep. Kanagawa **4**, 1–6 (1983).
136. Lin, H. & Zhu, X. Assemblages of Foraminifera and Ostracoda from surface sediments in the eastern part of the Laizhou Bay. *Prof. Pap. Stratigr. Palaeontol*. **20**, 85–97 (1986).1987
137. Zheng, S. Quaternary ostracods from Zhejiang Province. *Acta Micropalaeontol. Sin.* **3**, 429–434 (1986).
138. Ikeya, N. & Kashima, T. Ostracoda from the Holocene Iri Deposits, Kurihama, Yokosuka City. Annu. Rep. Yokosuka City Cult. Assets. Invest. **16**, 199–203 (1988).
139. Wang, Q., Li, Y., Tian, G. & Lin, F. Quaternary marine Ostracoda on the west coast of the Bohai Sea. Acta Oceanol. Sin. **7**, 94–103 (1988).
140. Ikeya, N. Characteristics of the inner bay ostracodes around the coast of Japan. URBAN KUBOTA **32**, 26–29 (1993).
141. Ikeya, N. & Shiozaki, M. Characteristic of the inner bay ostracodes around the Japanese islands – the use of ostracodes to reconstruct paleoenvironments. Mem. Geol. Soc. Jpn. **39**, 15–32 (1993).
142. Ikeya, N. Ostracoda in sediment cores from Yokohama Port. Annu. Rep. Yokohama Environ. Sci. Res. Inst. **116**, 27–33 (1995).
143. Kim, H., Huh, M. & Han, S. A preliminary report of the Quaternary Ostracoda from the Ulleung Basin in the East Sea, Korea. *J. Paleontol. Soc. Korea* **14**, 115–135 (1998).
144. Irizuki, T., Nakamura, Y., Takayasu, K. & Sakai, S. Faunal changes in Ostracoda (Crustacea) in Lake Nakaumi, southwest Japan, over the last 40 years. Geosci. Rep. Shimane Univ. **22**, 149–160 (2003).
145. Yasuhara, M. & Kumai, H. Fossil ostracodes from the Tako-Shell bed, Shimosa Group and Somei horizontal hollow tomb floor deposits formed in its outcrop in Somei, Tako-machi, Chiba Prefecture, Japan. *Monogr. Association Geol. Collab. Jpn.* **50**, 73–78 (2003).
146. Schornikov, E. I. & Zenina, M. A. Buried ostracods collected at the location of a nuclear submarine accident in the Chazhma Cove (Peter the Great Bay, Sea of Japan). *Russian J. Mar. Biol.* **33**, 200–203 (2007).
147. Tanaka, G., Naruse, H., Yamashita, S. & Arai, K. Ostracodes reveal the bed-origin of tsunami deposits. *Geophys. Res. Lett.* **39**, L05406 (2012).
148. Maekawa, T. Fossils discovered at the Toyotomi-pariod Osaka Castle stone wall excavation site. In Ruin of Osaka Castle 21 (ed. Osaka City Cultural Properties Association) 242–252 (Yoshida CV Press*,* 2024).
149. Yajima, M. Late Pleistocene Ostracoda from the Boso Peninsula, Central Japan. *Univ. Mus., Univ. Tokyo Bull.* **20**, 141–227 (1982).
150. Wang, P. & Zhao, Q. Ostracod distribution in bottom sediments of the East China Sea*.* In Marine micropaleontology of China (eds. Wang, P. et al) 70–92 (China Ocean Press, 1985).
151. Irizuki, T., Taru, H., Taguchi, K. & Matsushima, Y. Paleobiogeographical implications of inner bay Ostracoda during the Late Pleistocene Shimosueyoshi transgression, central Japan, with significance of its migration and disappearance in east Asia. Palaeogeogr. Palaeoclimatol. Palaeoecol. **271**, 316–328 (2009).
152. Jöst, A. B., Hong, Y. & Karanovic, I. First description of soft body parts of *Ambtonia* Malz and *Nipponocythere* Ishizaki (Ostracoda) from Korea with details on the genera’s geographic and paleogeographic distribution. *Mar. Micropaleontol.* **174**, 102029 (2022).
153. Müller, G. W. Die Ostracoden des Golfes von Neapel und der angrenzenden Meeres-Abschnitte. *Fauna und Flora des Golfes von Neapel und der angrenzenden Meeres-Abschnitte* **21**, 1–404 (1894).
154. Schornikov, E. I. & Dolgov, G. V. *Angulicytherura* gen. n. - a new ostracod genus of the family Cytheruridae from Far-Eastern Seas. *Biol. Morya* **21**, 29–36 (1995).
155. Yajima, M. & Ikeya, N. 11 Ostracodes from deep sea drilling project Leg 87. In *Initial reports of DSDP, 87* (eds. Kagami, H., Karig, D. E. & Coulbourn, W. T.) 605–608 (US Government Printing Office, 1985).
156. Tsukagoshi, A. & Kamiya, T. Heterochrony of the ostracod hingement and its significance for taxonomy. Biol. J. Linn. Soc. **57**, 343–370 (1996).
157. Tsukagoshi, A. & Parker, A. R. Trunk segmentation of some podocopine linages in Ostracoda. Hydrobiologia **419**, 15–30 (2000).
158. Hanai, T. Studies on the Ostracoda from Japan. 3 - Subfamily Cytherurinae G.W. Müller (emend. G.O. Sars) and Cytheropterinae, new subfamily. *J. Fac. Sci., Univ. Tokyo, Sec. 2* **11**, 11–36 (1957).
159. Tabuki, R. Plio-Pleistocene Ostracoda from the Tsugaru Basin, North Honshu, Japan. *Bull. Coll. Educ., Univ. Ryukyus* **29**, 27–160 (1986).
160. Tsukawaki, S., Kamiya, T., Kato, M., Nishikawa, M., Okubo, H., Ozawa, H., Shimizu, T., Yamamoto, Y., Omura, A. & Takayama, T. Preliminary Results on the Geological Examinations in a new outcrop at the type locality of the Pleistocene Omma Formation, Kanazawa City, Japan. Bull. Japan Sea Res. Inst. **28**, 45–63 (1997).
161. Schornikov, E. I. & Sokolenko, D. A. Ostracods-Indicators of Near-Bottom Water Masses in the Southern Part of Peter the Great Bay, Sea of Japan. *Russian J. Mar. Biol.* **25**, 215–218 (1999).
162. Lee, E.-H., Huh, M. & Schornikov, E. I. Ostracod fauna from the East Sea coast of Korea and their distribution - preliminary study on Ostracoda as an indicator of water pollution. *J. Geol. Soc. Korea* **36**, 435–472 (2000).
163. Ozawa, H., Kamiya, T., Itoh, H. & Tsukawaki, S. Water temperature, salinity ranges and ecological significance of the three families of Recent cold-water ostracods in and around the Japan Sea. *Paleontol. Res.* **8**, 11–28 (2004).
164. Ozawa, H. & Kamiya, T. The effects of glacio-eustatic sea-level change on Pleistocene cold-water ostracod assemblages from the Japan Sea. *Mar. Micropaleontol.* **54**, 167–189 (2005).
165. Ozawa, H. An overview of the geographical distribution and ecological significance of species in the three families of cryophilic ostracods (Crustacea: Ostracoda) in and around the Japan Sea with special reference to distribution of species in relation to water temperature-salinity ranges. *Taxa, Proc. Japanese Soc. Syst. Zool.* **20**, 26–40 (2006).
166. Ozawa, H. Faunal changes of cryophilic ostracods (Crustacea) in the Japan Sea, in relation to oceanographic environment: an overview. Fossils **82**, 21–28 (2007).
167. Ozawa, H. Preliminary report on the Middle Pleistocene ostracods from the Shichiba Formation on Sado Island in the eastern Japan Sea. Nihon-Kaiiki Kenkyu, Kanazawa Univ. **41**, 15–36 (2010).
168. Brady, G. S. & Norman, A. M. A monograph of the marine and freshwater Ostracoda of the North Atlantic and of Northwestern Europe. Section I: Podocopa. *Sci. Trans. R. Dublin Soc., Ser. 2* **4**, 63–270 (1889).
169. Müller, G. W. Zur naeheren Kenntnis der Cytheriden. Arch. Naturgeschichte. **50**, 1–18 (1884).
170. Brady, G. S. Report on the Ostracoda dredged amongst the Hebrides. *Rep. British Assoc. Adv. Sci.* **36**, 208–211 (1867).
171. Irizuki, T., Masuda, F. & Ikeya, N. Sedimentary facies and Ostracodes of the Middle Pleistocene Hamamatsu Formation from the excavation site of Palaeoloxocon naumanni in Sahama Town, Hamamatsu City, Shizuoka Prefecture. Shizuoka Chigaku **87**, 1–13 (2003).
172. Sars, G. O. An account of the Crustacea of Norway with short descriptions and figures of all the species, volume 9 (Ostracoda)(Parts15-16) 241–277 (Bergen Mus., 1928).
173. Baird, W. The natural history of the British Entomostraca. Mag. Zool. Bot. **2**, 132–144, 400–412 (1838).
174. Schornikov, E. I. On the study of Ostracoda (Crustacea) from the intertidal zone of the Kuril Islands. *Sbornik. Rabot., Inst. Biol. Morya, Dalnevostochnyy Nauchnyy Tsentr., Akademia Nauk USSR* **1**, 137–214 (1974).
175. Okubo, I. Three species of *Xestoleberis* (Ostracoda) from the Inland Sea of Japan. *Proc. Japanese Soc. Syst. Zool.* **16**, 9–17 (1979).
176. Okubo, I. On the life history and size of *Xestoleberis hanaii*. Res. *Bull. Shujitsu Women’s Coll. and Shujitsu Jr. Coll.* **14**, 19–43 (1984).
177. Zhao, Q., Wang, P. & Zhang, Q. Distribution of Ostracoda in the bottom sediments from the northern shelf of South China Sea. Acta Oceanol. Sin. **8**, 590–602 (1986).
178. Whatley, R. & Zhao, Q. A revision of Brady’s 1869 study of the Ostracoda of Hong Kong. *J. Micropalaeontol.* **6**, 21–29 (1987).
179. Abe, K. & Vannier, J. M. C. The role of 5th limbs in mating behaviour of two marine podocopid ostracods, *Bicornucythere bisanensis* (Okubo, 1975) and *Xestoleberis hanaii* Ishizaki, 1968. In *Ostracoda in the earth and life sciences* (eds. McKenzie, K. G. & Jones, P. J.) 581–590 (Balkema, 1991).
180. Yajima, M. Early Miocene Ostracoda from Mizunami, central Japan. *Bull. Mizunami Fossil Mus.* **19**, 247–267 (1992).
181. Yumoto, M. The shell structure of the carapace in *Xestoleberis hanaii* Ishizaki (Crustacea, ostracoda). *Trans. Proc. Palaeontol. Soc. Japan, New Ser.* **176**, 638–649 (1994).
182. Ikeya, N. & Kato, M. The life history and culturing of Xestoleberis hanaii (Crustacea, Ostracoda). Hydrobiologia **419**, 149–159 (2000).
183. Vannier, J., Wang, S. Q. & Coen, M. Leperditicopid arthropods (Ordovician-Late Devonian): Functional morphology and ecological range. J. Paleontol. **75**, 75–95 (2001).
184. Kondo, H., Toyofuku, T. & Ikeya, N. Mg/Ca ratios in the shells of cultured specimens and natural populations of the marine ostracode *Xestoleberis hanaii* (Crustacea). *Hydrobiologia* **225**, 3–13 (2005).
185. Ozawa, H. & Tsukawaki, S. Preliminary report on modern ostracods in surface sediment samples collected during R. V. Tansei-maru Cruise KT04-20 in the southwestern Okhotsk Sea and the northeastern Japan Sea off Hokkaido, north Japan. *Ann. Res. Inst. Japan Sea Reg.* **39**, 31–48 (2008).
186. Kaji, T. & Tsukagoshi, A. Heterochrony and modularity in the degeneration of maxillopodian nauplius eyes. *Biol. J. Linn. Soc.* **99**, 521–529 (2010).
187. Noraswana, N. F. & Ramlan, O. Recent benthic Ostracoda of Pahang River Delta, Pahang Darul Makmur. AIP Conf. Proc. **1614**, 610–615 (2014).
188. Yamada, S. Formation and function of the “*Xestoleberis*-spot” in *Xestoleberis hanaii* (Crustacea: Ostracoda). *J. Morphol.* **278**, 1570–1576 (2017).
189. Faiz, O. R. & Raoh, M. M. Abundance and diversity of benthic ostracod in sediments around Pulau Bidong, Terengganu. *Malays. Appl. Biol.* **47**, 113–118 (2018).
190. Mandelstam, M. J. New ostracode genera and species. *Trudy Vsesoyuznogo Neftyanogo Nauchno-Issledovatelskogo Geologo-RazvedochnogoInstituta (VNIGRI) Novaya Seriya* **115**, 232–299 (1958).
191. Brady, G. S., Crosskey, H. W. & Robertson, D. A monograph of the post-Tertiary Entomostraca of Scotland including species from England and Ireland. *Annu. Vol. Palaeont. Soc.* **28**, 1–232(1874).
192. Zhao, Q. & Whatley, R. Distribution of the ostracod genera *Krithe* and *Parakrithe* in bottom sediments of the East China and Yellow seas. *Mar. Micropaleontol.* **32**, 195–207 (1997).
193. Sars, G. O. Cypridæ (concluded), Cytheridæ (part). In An account of the Crustacea of Norway, 9(IX-X) (ed. Sars, G. O.) 137–176 (Bergen Mus*.*, 1925).
194. Stephenson, M. B. Miocene and Pliocene Ostracoda of the genus Cytheridea from Florida. *J. Paleontol.* **12**, 127–148 (1938).
195. Stephenson, M. B. Some microfossils from the Potamides Matsoni Zone of Louisiana. *Geol. Bull., Dep. Conservation, Louisiana Geol. Surv.* **6**, 187–196 (1935).
196. Okubo, I. Six species of marine Ostracoda from the Inland Sea of Seto. *Res. Bull. Okayama Shujitsu Women’s Coll. and Okayama Shujitsu Jr. Coll.* **9**, 143–157 (1979).
197. Okubo, I. On the genus *Perissocytheridea* Stephenson, 1938. *Res. Bull. Shujitsu Women’s Coll. and Shujitsu Jr. Coll.* **13**, 403–410 (1983).
198. Puri, H. S. The ostracod genus *Hemicythere* and its allies. *Washington Acad. Sci. J.* **43**, 169–179 (1953).
199. Pokorny, V. Contribution to the Morphology and Taxonomy of the Subfamily Hemicytherinae Puri. Acta Univ. Carolinae **2**, 1–36 (1955).
200. Baird, W. The natural history of the British Entomostraca. (Ray Soc, Lond., 1850).
201. Huang, B. Ostracoda from the column samples under surface deposit on the bottom of North Bohai Sea. *J. Oceanogr. Huanghai and Bohai Seas* **3**, 42–51 (1985).
202. Okubo, I. On the Recent *Aurila* species from Japan. In Evolutionary biology of Ostracoda (eds. Hanai, T., Ikeya, N. & Ishizaki, K.) 135–144 (Kodansha, 1988).
203. Yamada, K., Kusunoki, S., R. & Kusumi, A. Paleoenvironmental changes during the Pleistocene in the northern part of the Niitsu Hills, Niigata Prefecture based on fossil ostracod assemblages. *J. Geol. Soc. Jpn.* **127**, 575–591 (2021).
204. Schornikov, E. I. & Tsareva, O. A. New Ostracoda of the genus *Aurila* from the N.W. Pacific. *Mitt. Naturhistrischen Mus. Hamburg Inst.* **92**, 237–253 (1995).
205. Ishizaki, K. & Kato, M. The basin development of the diluvium Furuya Mud Basin, Shizuoka Prefecture, Japan, based on faunal analysis of fossil ostracodes. In *Progress in micropaleontology*, *selected papers in honour of Professor Kiyoshi Asano* (eds. Takayanagi, Y. & Saito, T.) 118–143 (Micropaleontology Press, 1976).
206. Coryell, H. N. & Fields, S. A Gatun Ostracode fauna from Cativa, Panama. *Am. Mus. Novit.* **956**, 1–18 (1937).
207. Ohmert, V. W. Die Coquimbinae, eine neue Unterfamilie der Hemicytheridae (Ostracoda) aus dem Pliozän von Chile. *Mitt. Bayer. Staatssamml. Palaeontol. Hist. Geol.* **8**, 127–165 (1968).
208. Yajima, M. Quaternary Ostracoda from Kisarazu near Tokyo. *Trans. Proc. Palaeontol. Soc. Jpn., New Ser.* **112**, 371–40s9 (1978).
209. Hou, Y. & Zhao, Y.-H. Classification and significance of ornamentations and normal pores in Mesozoic and Cenozoic ostracods from China. *Acta Micropalaeontol. Sin.* **8**, 223–238 (1986).
210. Gou, Y. Ostracoda. In *Cenozoic paleobiota of the continental shelf of the East China Sea (Donghai), Paleozoological Volume* (eds. Research Party of Marine Geology, Ministry of Geology and Mineral Resources, Institute of Geology, Chinese Academy of Geological Sciences) 134–164, 168–172 (Geol. Publ. House, 1989).
211. Ozawa, H. Japan Sea ostracod assemblages in surface sediments: their distribution and relationships to water mass properties. *Paleontol. Res.* **7**, 257–274 (2003).
212. Tanaka, G. Recent benthonic ostracod assemblages as indicators of the Tsushima warm current in the southwestern Sea of Japan. *Hydrobiologia* **598**, 271–284 (2008).
213. Irizuki, T., Kusumoto, M., Ishida, K. & Tanaka, Y. Sea-level changes and water structures between 3.5 and 2.8 Ma in the central part of the Japan Sea Borderland: Analysis of fossil Ostracoda from the Pliocene Kuwae Formation, central Japan. Palaeogeogr. Palaeoclimatol. Palaeoecol. **245**, 421–443 (2007).
214. Swain, F. M. Pleistocene Ostracoda from the Gubik Formation, Arctic Coastal Plain, Alaska. *J. Paleontol.***37**, 798–835 (1963).
215. Neale, J. W. The genus *Finmarchinella* Swain 1963 (Crustacea: Ostracoda) and its species. *Bull. Br. Mus. Nat. Hist. (Zool.)* **27**, 83–93 (1974).
216. Irizuki, T. Morphology and taxonomy of some Japanese Hemicytherin Ostracoda –with particular reference to ontogenetic changes of marginal pores –. Trans. Proc. Palaeontol. Soc. Jpn., New Ser. **170**, 186–211 (1993).
217. Ruggieri, G. Gli Ostracodi delle sabbie gride Quaternarie (Milazziano) di Imola. Giornale di Geologia **21**, 1–57 (1950).
218. Roemer, F. A. Die Cytherinen des Molasse-Gebirges. *N. Jahrb. Mineral., Geogn., Geol. Petrefaktenkunde* **5**, 514–519 (1838).
219. Sylvester-Bradley, P. C. The ostracode genus Cythereis. *J. Paleontol.* **22**, 792–797 (1948).
220. Wang, P., Min, Q., Bian, Y. & Hua, D. Characteristic of foraminiferal and ostracod thanatocoenoses from some Chinese estuaries and their geological significance*.* In Marine micropaleontology of China (eds. Wang P, et al.) 229–255 (China Ocean Press, 1985).
221. Zhao, Q., Wang, P. & Zhang, Q. Ostracoda in bottom sediments of the South China Sea off Guangdong Province, China: their taxonomy and distribution*.* In Marine micropaleontology of China (eds. Wang, P., et al.) 196–217 (China Ocean Press, 1985).
222. Wang, P., Min, Q., Bian, Y. & Cheng, X. On Micropaleontology and Stratigraphy of Quaternary marine transgression in East China*.* In Marine micropaleontology of China (eds. Wang, P., et al.) 265–284 (China Ocean Press, 1985).
223. Wang, P. & Bian, Y. Foraminifera and Ostracoda in bottom sediments of the Bohai Gulf and their bearing on Quaternary paleoenvironments*.* In Marine micropaleontology of China (eds. Wang, P., et al.) 133–150 (China Ocean Press, 1985).
224. Hu, C. & Tao, H. Studies on the ostracod fauna from Jurong, Singapore. *Annu. Rep. Natl. Taiwan Mus.* **43**, 11–47 (2000).
225. Noraswana, N. F., Ramlan, O. & Muhamad Naim, A. M. Distribution of Recent benthic Ostracoda in Pahang River Estuary, east coast peninsular Malaysia. Malay J. Microsc. **10**, 27–33 (2014).
226. Ramlan, O. & Noraswana, N. F. Distribution of Recent Ostracoda in offshore sediment of the South China Sea. *Pertan. J. Sci. Technol.* **22**, 433–444 (2014).
227. Müller, G. W. Crustacea. Ostracoda. Königl. *Preuß. Akad. Wiss. Berlin* **31**, 1–434 (1912).
228. Tabuki, R. & Nohara, T. OKINAWA ISLAND (Plio-Pleistocene tropical and subtropical Ostracoda, and Ostracoda of Recent coral reef). *In* *Guidebook of Excursions for the 9th International Symposium on Ostracoda*. (ed. Organizing Committee 9th International Symposium on Ostracoda) 1–32 (Shizuoka University Press, 1985).
229. Zhao, Q. & Whatley, R. Recent podocopid Ostracoda of the Sedili River and Jason Bay, southeastern Malay Peninsula. *Micropaleontology* **35**, 168–187 (1989).
230. Irizuki, T & Seto, K. Temporal and spatial variations of paleoenvironments of Paleo-Hamana Bay, central Japan, during the Middle Pleistocene – Analyses of fossil ostracode assemblages, and total organic carbon, total nitrogen and total sulfur contents –. J. Geol. Soc. Jpn. **110**, 309–324 (2004).
231. Ramlan, O. & Noraswana, N. F. Distribution of Ostracods in offshore sediment around Pulau Tioman, Pahang. *Malay. App. Biol.* **38**, 11–19 (2009).
232. Ramlan, O. & Noraswana, N. F. Taburan Ostracoda resen di dalam sedimen luar Pantai sekitar Pulau Besar, Johor. *Sains. Malay.* **39**, 199–207 (2010).
233. Yasuhara, M., Hunt, G., Okahashi, H. & Brandão, S. N. Taxonomy of deep-sea trachyleberidid, thaerocytherid, and hemicytherid genera (Ostracoda). *Smithson. Contrib. Paleobiol.* **96**, 1–216 (2015).
234. Li, S., Cui, X., Xu, H., Zhang, Z., Zhao, X., Gao, Z., Liu, Q. & Liu, X. Distribution characteristics of ostracods and evolution of the sedimentary environment of the Late Quaternary in the Laizhou Bay, *Bohai Sea. Quat. Sci.* **36**, 1475–1488 (2016).
235. Omar, R., Faiz, N. N. & Yusoff, M. N. A. Recent benthic Ostracoda in offshore sediment of Pulau Perhentian, Terengganu. *Malay. Appl. Biol.* **46**, 15–19 (2017).
236. Brady, G. S. On new or imperfectly known species of Ostracoda, chiefly from New Zealand. Trans. Zool. Soc. Lond. **14**, 429–452 (1898).
237. Wang, P., Min, Q. & Bian, Y. Distribution of Foraminifera and Ostracoda in bottom sediments of the northwestern part of the South Huanghai (Yellow) Sea and its geological significance*.* In Marine micropaleontology of China (eds. Wang, P., et al) 93–114 (China Ocean Press, 1985).
238. Huh, M. & Paik, K. Miocene Ostracoda from the Seojeongri area, Pohang Basin, Korea. J. Geol. Soc. Korea **28**, 273–283 (1992).
239. Brandão, S. N., Yasuhara, M., Irizuki, T. & Horne, D. J. The ostracod genus Trachyleberis (Crustaca; Ostracoda) and its type species. *Mar. Biodivers.* **43**, 363–405 (2013).
240. Iwatani, H., Irizuki, T. & Goto, T. Temporal changes of Plio-Pleistocene Ostracoda from the Takanabe Formation, Miyazaki Group, Southwest Japan. *Paleontol. Res.* **15**, 269–289 (2011).
241. Huh, M. & Whatley, R. New species of Miocene cytheracean Ostracoda from the Pohang Basin, SE Korea. J. Micropalaeontol. **16**, 31–40 (1997).
242. Howe, H. V. Type Saline Bayou Ostracoda of Louisiana. *Louisiana Geol. Surv., Geol. Bull.* **40**, 1–62 (1963).
243. Malz, H. Plio-/Pleistozäne Buntoniini von SW-Taiwan. *Senckenberg, lethaea* **63**, 377–411 (1982).
244. McKenzie, K. G. Recent Ostracoda from Port Phillip Bay, Victoria. Proc. R. Soc. Victoria **80**, 61–106 (1967).
245. Hu, C. Studies on ostracodes from the Toukoshan Formation (Pleistocene), Miaoli district, Taiwan. *P*et. Geol. Taiwan **14**, 181–217 (1977).
246. Malz, H. & Ikeya, N. On the occurrence of *Sinoleberis* in the Pacific (ostracoda; Pliocene to Recent; Taiwan and Japan). *Senckenberg. lethaea* **63**, 413–427 (1982).
